# Synthesis and in silico screening of a library of β-carboline-containing compounds

**DOI:** 10.3762/bjoc.8.117

**Published:** 2012-07-10

**Authors:** Kay M Brummond, John R Goodell, Matthew G LaPorte, Lirong Wang, Xiang-Qun Xie

**Affiliations:** 1 Department of Chemistry, Chemical Methodologies and Library Development, University of Pittsburgh, Pittsburgh, Pennsylvania 15260, USA; 2 Department of Pharmaceutical Sciences and Computational Chemical Genomics Center, School of Pharmacy; Drug Discovery Institute; Department of Computational Biology; University of Pittsburgh, Pittsburgh, Pennsylvania 15260, USA

**Keywords:** β-carboline, biological activity, chemical diversity, diversity-oriented synthesis, in silico screening

## Abstract

The synthesis of a library of tetrahydro-β-carboline-containing compounds in milligram quantities is described. Among the unique heterocyclic frameworks are twelve tetrahydroindolizinoindoles, six tetrahydrocyclobutanindoloquinolizinones and three tetrahydrocyclopentenoneindolizinoindolones. These compounds were selected from a virtual combinatorial library of 11,478 compounds. Physical chemical properties were calculated and most of them are in accordance with Lipinski’s rules. Virtual docking and ligand-based target evaluations were performed for the β-carboline library compounds and selected synthetic intermediates to assess the therapeutic potential of these small organic molecules. These compounds have been deposited into the NIH Molecular Repository (MLSMR) and may target proteins such as histone deacetylase 4, endothelial nitric oxide synthase, 5-hydroxytryptamine receptor 6 and mitogen-activated protein kinase 1. These in silico screening results aim to add value to the β-carboline library of compounds for those interested in probes of these targets.

## Introduction

Identification of a comprehensive set of small organic molecules capable of selectively modifying the function of biological targets tremendously impacts modern medical research and drug discovery efforts [1]. Currently, this set of small molecules is largely occupied by in-house libraries and commercially available compounds. The NIH Roadmap initiative was established to address a recognized limitation of current compound diversity resulting in the Molecular Libraries Probe Centers Network (MLPCN) which has, since its inception, garnered a library of over 370,000 chemically diverse small molecules in a central molecule repository [2]. This supply of compounds has been made possible by researchers across the disciplines, but largely by synthetic chemists who are preparing compounds with an eye towards biologically relevant targets. Another goal of the NIH Roadmap is the development of enabling methods for the synthesis of these structurally diverse compound libraries; amongst these methods, skeletal diversification strategies have emerged as particularly efficient for maximizing structural diversity [3].

Previous work in the Brummond laboratory has demonstrated that an allene-containing β-carboline provided a good starting point for synthesizing six novel types of hetero-frameworks, all skeletally unique [4]. Moreover, scope and limitation studies contributed to an understanding of chemistries that would possess the robustness necessary for library preparation. Information gained from these experiments was then utilized in the construction of a virtual library of 11,748 compounds. A diversity analysis was performed using B (Burden) C (CAS) UT (Pearlman at the University of Texas) metrics and Tanimoto coefficients (Tc) and this virtual compound library was mapped onto the existing chemical space of the NIH Molecular Libraries Small Molecule Repository (MLSMR) [4]. When considering the physical properties most important to bimolecular interactions, atomic Gasteiger–Hückel charges, polarizabilities, and hydrogen-bond acceptors, these virtual compounds were found to occupy new chemical space when compared to the 327,000 compounds in the MLSMR. A small subset of these compounds was subsequently identified as ones representing a maximally diverse chemical space. The synthesis of a modified subset of this virtual compound library is described within, where modifications were mainly driven by studies of compound stability. Furthermore, a high throughput, in silico screening analysis of this library identified a number of potential biological targets for the compounds.

## Results and Discussion

**Figure 1:**
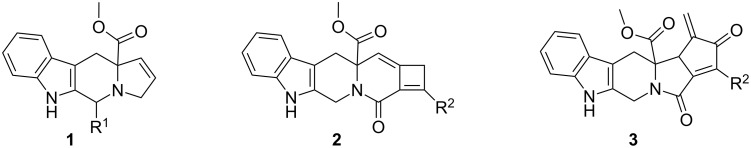
Tetrahydro-β-carboline containing scaffolds **1**–**3**.

Scaffolds **1**, **2** and **3** (Figure 1) were chosen for library preparation based upon favorable Tanimoto coefficient (Tc) scores when compared to the MLSMR, conformational constraints imposed by the β-carboline moiety, and the number of building blocks available for the diversifying elements R^1^ and R^2^.

The syntheses of tetrahydro-β-carbolines **6{1**–**16}** were accomplished in a manner entirely analogous to that reported previously (Table 1, entries 1–16) [4]. For example, the allenic methyl ester of tryptophan **4** was reacted with a number of aldehydes **5{1**–**15}** under acidic conditions to produce the corresponding products in yields ranging from 54–89%. A range of aldehydes were accommodated in the Pictet–Spengler reaction, including formaldehyde (Table 1, entry 1), alkyl aldehydes (Table 1, entries 2 and 3), aryl aldehydes with electron-withdrawing and electron-donating groups (Table 1, entries 4–7, 14 and 15), heteroaromatic aldehydes (Table 1, entries 8–13) and glyoxalates (Table 1, entry 16). Moreover, useful quantities of β-carboline-containing products were obtained (43–100 mg). For entries 2–15, mixtures of two diastereomers were obtained. Since the mixtures could not be readily separated by column chromatography, diastereomeric ratios were determined by ^1^H NMR and were advanced without further purification. Reaction of allene **6** under the silver-nitrate-mediated cyclization conditions afforded the desired fused pyrrolines **1**. However, in the initial phases of this cyclization process, a color change was noted during the purification process. Indeed, when NMR stability studies were performed on the syn- and anti-pyrrolines **1{5}**, decomposition of both diastereomers was evident. Although it was generally difficult to isolate the individual diastereomers, they could be separated by column chromatography. It was found that anti-**1{5}** decomposed more rapidly than syn-**1{5}** during the ^1^H NMR stability studies, when compared to an internal standard. These results combined with previously reported skeletal reorganization processes of functionalized β-carbolines, led to concerns about the long-term storage of these compounds and their inclusion in the MLSMR [5].

**Table 1:** Library of *N*-tosyltetrahydro-β-carbolinepyrrolines **7{1–7}**.

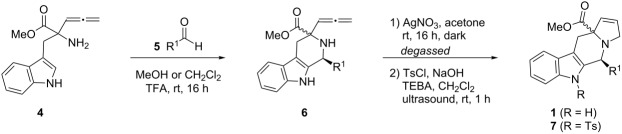						

entry	R^1^		yield of **6**^a^ (%)	anti:syn^b^	yield of **7**^a^ (%)	purity^c^

1	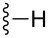	**5{1}**	68 **6{1}**	NA	28 **7{1}**	98%
2	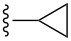	**5{2}**	74 **6{2}**	2.5:1	17 syn-**7{2}**	98%
3	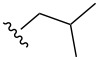	**5{3}**	81 **6{3}**	2.1:1	23 syn-**7{3}**	98%
4	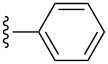	**5{4}**	73 **6{4}**	2.4:1	11 anti-**7{4}** 28 syn-**7{4}**	98% 98%
5	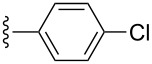	**5{5}**	75 **6{5}**	2.4:1	34 anti-**7{5}** 17 syn-**7{5}**	98% 98%
6	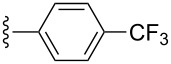	**5{6}**	74 **6{6}**	1.3:1	35 anti-**7{6}** 10 syn-**7{6}**	98% 98%
7	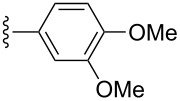	**5{7}**	54 **6{7}**	2.7:1	12 anti-**7{7}** 17 syn-**7{7}**	98% 98%
8	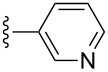	**5{8}**	89 **6{8}**	2.3:1	ND^d^	
9	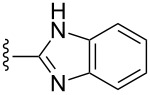	**5{9}**	78 **6{9}**	1.2:1	ND^e^	
10	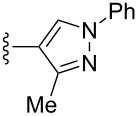	**5{10}**	80 **6{10}**	1:1.1	ND^d^	
11	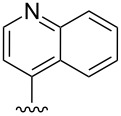	**5{11}**	70 **6{11}**	1.3:1	ND^e^	
12	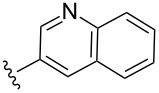	**5{12}**	80 **6{12}**	3.5:1	ND^e^	
13	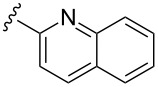	**5{13}**	63 **6{13}**	1:1.4	ND^e^	
14	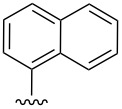	**5{14}**	58 **6{14}**	2.3:1	ND^d^	
15	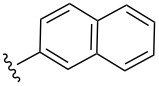	**5{15}**	80 **6{15}**	2.3:1	ND^d^	
16	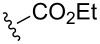	**5{16}**	88 **6{16}**	1.4:1	8 syn-**1{16}**	99%

^a^Isolated yields; ^b^dr determined by ^1^H NMR; ^c^purity established by LCMS/ELS; ^d^compound **6** detected; ^e^compound **6** not detected.

To increase the stability of this class of compounds, a toluenesulfonyl group was added to the indole nitrogen of **1{1**–**7}** to give *N*-tosyl-tetrahydro-β-carbolinepyrroline derivatives **7{1**–**7}**. These tosylated derivatives exhibited improved stability as evidenced by ^1^H NMR (Table 1, entries 1–7, and Supporting Information File 1, S76–S81). Incorporation of the tosyl group also eased the chromatographic separation of the syn- and anti-isomers for entries 2–7, thus compounds **7{2**–**7}** were obtained as single diastereomers. Low to moderate yields for this two-step reaction sequence were attributed to a problematic tosylation due to the sterically hindered nature of the indole nitrogen atom. Moreover, unforeseen limitations were encountered for the heteroaromatic and naphthyl-containing β-carboline intermediates (Table 1, entries 8–15). While in some cases the intermediate pyrrolines **1** were observed (Table 1, entries 8, 10, 14, 15), the corresponding tosylated products were not obtained. The heteroaromatic examples (Table 1, entries 9, 11–13), did not undergo cyclization upon treatment with silver nitrate. For these cases, it was assumed that competing coordination of the heteroatom to the silver ion was an issue; however, attempts were not made to alter the reaction conditions for these substrates. Furthermore, conversion of the naphthalene-containing analogues **1{14}** and **1{15}** to their corresponding tosylates was not successful.

Next, compounds possessing the cyclobutene-fused β-carboline skeleton were assembled from the versatile allenyl intermediate **6**. For this subset of compounds, acylation of the sterically hindered amine of **6{1}** with the ynoic acids **8{1**–**8}**, by using bromo-tris-pyrrolidino-phosponium hexafluorophosphate (PyBroP), provided the requisite allene-yne substrates **9{1,2}**–**9{1,8}**. For the coupling reaction of the ynoic acids with an aryl group on the terminus of the alkyne **8{5–8}**, the desired allene-ynes **9{1,5}**–**9{1,8}** were afforded along with the [2 + 2] cycloadducts **2{1,5}**–**2{1,8}** (Table 2, entries 5–8). Previous studies with related tryptophan-substituted allene-ynes, required much higher temperatures (225 °C versus rt) to give the [2 + 2] cycloadducts, albeit none of these examples possessed two radical stabilizing groups on the alkyne. Because the calculations performed by Tantillo [6] regarding the thermal [2 + 2] cycloaddition reaction suggest that the energy barrier for this reaction should not be effected by the presence of two radical stabilizing groups over one, it was postulated that this cycloaddition process was facilitated by exposure to incident light [7]. Thus, the mixtures of compounds **2** and **9** were reconstituted in CH_2_Cl_2_ and placed in front of two 6 W UV lamps for 16 h at rt to afford the desired cyclobutenes (Method B, Table 2, entries 5–8). During optimization of the conditions for the [2 + 2] cycloaddition reaction (Method A), it was found that reducing the reaction temperature from 225 °C to 160 °C afforded cyclobutene **2{1,3}** in 73% yield (Table 2, entry 3). Similarly, allene-yne **9{1,5}** was subjected to the lower reaction temperature (Method A) to produce cyclobutene **2{1,5}** in 57% yield (Table 2, entry 5).

**Table 2:** Library of tetrahydro-β-carbolinecyclobutenes **2{1,3}**–**2{1,8}**.

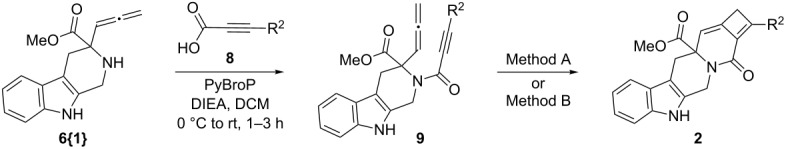						

entry	R^2^		yield of **9**^a^ (%)	method^b^	yield of **2**^a^ (%)	purity^c^

1	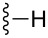	**8{1}**	0 **9{1,1}**	—	—	—
2	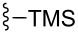	**8{2}**	64 **9{1,2}**	A	ND^d^	—
3	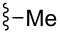	**8{3}**	75^e^ **9{1,3}**	A	73 **2{1,3}**	99%
4	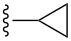	**8{4}**	71 **9{1,4}**	A	57 **2{1,4}**	99%
5	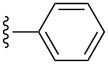	**8{5}**	**9{1,5}** **2{1,5}**	A B	57^f^ **2{1,5}** 40 **2{1,5}**	99%
6	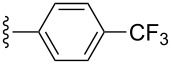	**8{6}**	**9{1,6}** **2{1,6}**	B	41 **2{1,6}**	99%
7	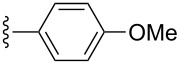	**8{7}**	**9{1,7}** **2{1,7}**	B	30 **2{1,7}**	99%
8	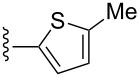	**8{8}**	**9{1,8}**	B	33 **2{1,8}**	99%

^a^Isolated yield; ^b^method A: μW, 160 °C, DMF, 10 min; method B: Placed in front of two 6 W UV lamps (245 nm), CH_2_Cl_2_, rt, 16 h, no stirring; ^c^purity established by LCMS/ELS; ^d^ND = not detected; ^e^μW, 225 °C, DMF, 7 min, (39%); ^f^separated **9{1,5}** (57% yield) from **2{1,5}** (18% yield) after the coupling reaction then submitted **9{1,5}** to method A to give **2{1,5}** in 68% yield. This compound was recombined with the previously isolated **2{1,5}** to afford a combined 57% yield.

For the final library scaffold, a small subset of α-methylenecyclopentenone-containing tetrahydro-β-carbolines was synthesized. These compounds contain a general substructure that has recently been shown to inhibit DNA damage checkpoints [8]. Allene-ynes **9{1,3}–9{1,4}** undergo Pauson–Khand cyclocarbonylation reactions when treated with molybdenum hexacarbonyl in DMSO/toluene solutions (Table 3). Allene-yne **9{1,2}** afforded a mixture of four compounds comprising two diastereomers of **3{1,2}**, the 4-alkylidene cyclopentenone, resulting from the cyclocarbonylation reaction with the distal double of the allene, and a fourth compound, which could not be identified (see spectral data in Supporting Information File 1). Aryl-substituted alkynones **9{1,5}**–**9{1,8}** were not available for the molybdenum-mediated cyclocarbonylation process due to competing [2 + 2] cycloaddition reactions (Table 2).

**Table 3:** Synthesis of α-methylenecyclopentenone library **3{1,3}**–**3{1,4}**.

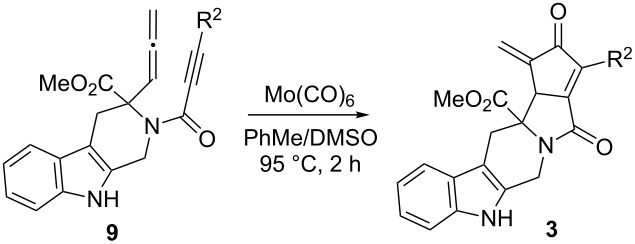				

entry	R^2^		yield of **3**^a^ (%)	purity^b^

1	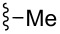	**9{1,3}**	48 **3{1,3}**	99%
2	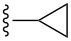	**9{1,4}**	46 **3{1,4}**	99%

^a^Isolated yields; ^b^purity determined by LCMS/ELS.

**Figure 2:**
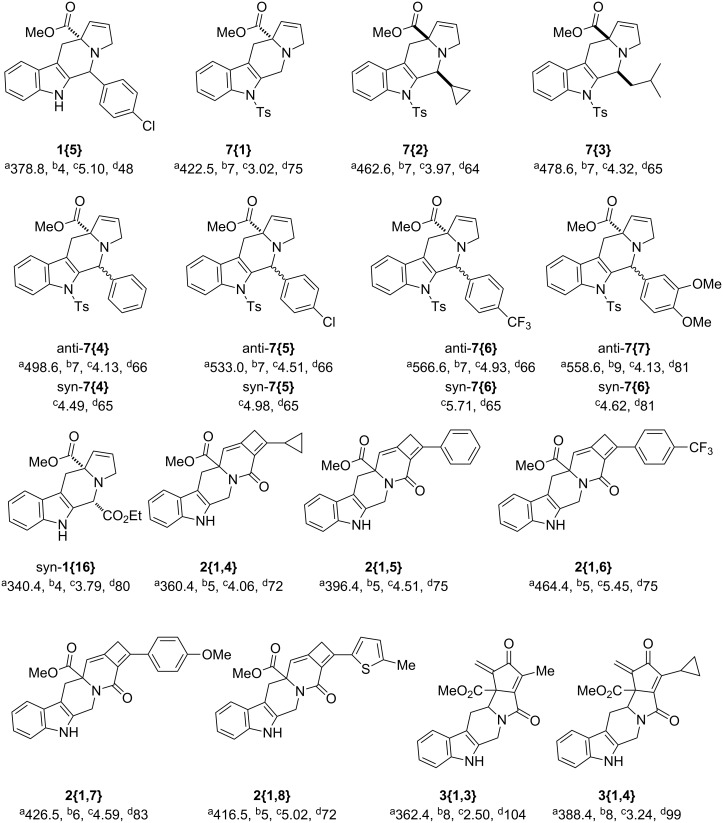
Library of tetrahydro-β-carboline containing compounds **1**–**7** and calculated properties (^a^molecular weight; ^b^hydrogen-bond donor/acceptors; ^c^cLogP; ^d^polar surface area (PSA)).

The majority of these β-carboline-containing products exhibit acceptable calculated physical–chemical properties in accordance with Lipinski’s rule of five (Figure 2) [9–10]. These favorable properties and structural novelty make these valuable candidates for deposition in the MLSCN for biological activity evaluation.

Diversity-oriented synthesis (DOS) has been employed to generate thousands of the organic compounds that have been deposited in the NIH molecular repository for medicinal chemistry research. Deciphering the therapeutic potential of this many compounds is a continuing challenge. By combining chemogenomics databases, such as Protein Data Base (PDB) and ChEMBL, it is possible to map new compounds into existing chemical space and to predict protein targets for new compounds, for which there are two complementary strategies that can be implemented. One is a structure-based docking strategy, in which a query compound is fit into a series of protein binding pockets to identify favorable compound–protein interactions. A second approach is a ligand-based strategy in which the structural similarities between a query compound and a collection of bioactive compounds are identified. In the present study, both of these strategies were used to predict potential targets of the newly synthesized library of β-carboline-containing compounds.

### High-throughput docking studies for protein-target prediction of newly synthesized compounds

Molecular docking studies were performed with the 34 newly synthesized compounds, represented by scaffolds **1**, **2**, **3**, and allenyl precursors **4**, **6{1**–**16}**, **9{1**–**4}** to identify potential protein targets [11]. Protein structures were downloaded from the PDB [12] and the analysis was limited to a selection of the 607 proteins defined as “druggable” targets, in order to reduce computational time [13]. (The complete listing of these proteins and their PDB IDs are provided in Supporting Information File 2). The Surflex-dock module of the Sybyl software was employed for protein preparation and docking of the β-carboline library [14–15]. Water molecules and ligands were removed from the protein structures and the active site of each protein was defined by the corresponding residues around the cocrystallized ligands. In-house algorithms were used to evaluate ligand-docking efficiency, and docking scores were used to assess and rank the protein targets.

**Figure 3:**
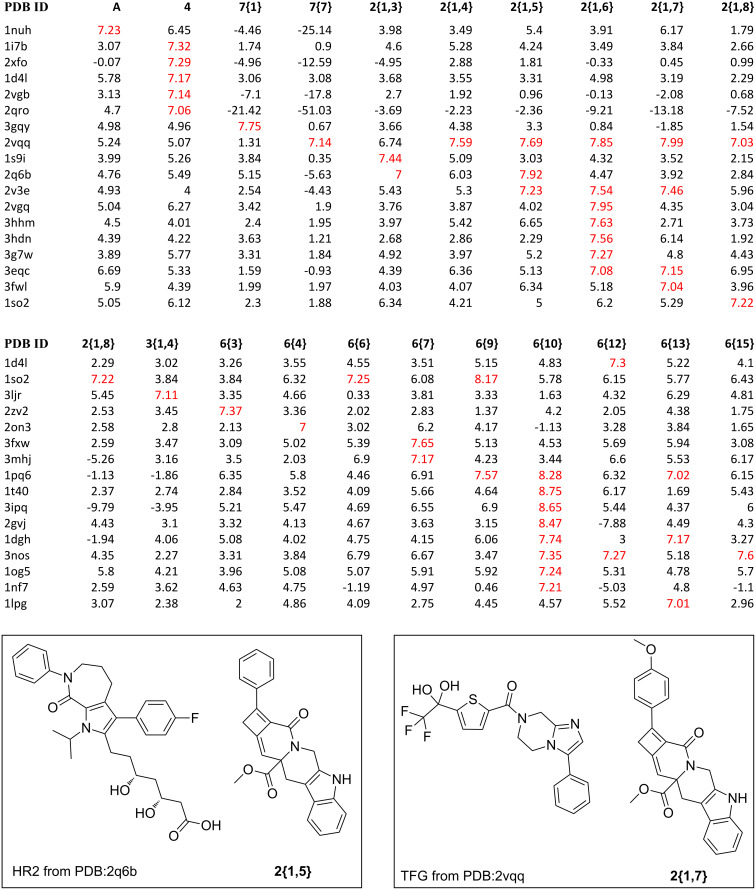
Results of high-throughput docking analysis. Top: A docking-score matrix arranged by compound IDs and PDB IDs; bottom: Structures of known ligands HR2 and TGF and the newly synthesized compounds **2{1,5}** and **2{1,7}**. Docking scores larger than 7.0 are red colored and can be mapped to *K*_d_ values less than 100 nM. The corresponding protein names of PDB IDs and the full docking-score matrix are listed in Supporting Information File 1.

A portion of the protein-scoring matrix is illustrated in Figure 3. Several interesting results emerged from this in silico analysis: (1) Twenty of the new compounds have docking scores greater than 7.0, a number that can be mapped to *K*_d_ values less than 100 nM, for several protein targets; (2) six compounds, **7{7}** and **2{1,4**–**1,8},** are predicted to be ligands for a single protein, human HDAC4 (PDBID:2vqq); (3) compounds **2{1,5}** and **2{1,7}** are predicted to be high-affinity ligands for 3-hydroxy-3-methylglutaryl-coenzyme A, reductase (PDBID:2q6b) and HDAC4, respectively, even though they are structurally different from the corresponding cocrystallized ligands HR2 and TGF; and (4) compounds **2{1,6}** and **6{10}** are predicted to be ligands for a total of 15 protein targets. This high number of potential protein targets may be due to the electronegative trifluoromethyl group on **2{1,6}** and the effect it would have on the α,β-unsaturated amide and the purported bioactivity of the pyrazole group of **6{10}**.

### Ligand-based strategy for target prediction

Ligand-based target prediction algorithms have been developed based upon an established medicinal chemistry principle that structurally similar compounds, with comparable physical properties, should convey related biological properties [16–17]. In this study, structural similarities were calculated between the compounds of the β-carboline library and the bioactive compounds in the well-annotated database, ChEMBL version 13, the largest publicly available compound-target database, containing 1,143,682 distinct compounds, 8,845 targets and 6,933,068 bioactivity entries from 44,682 publications and PubChem bioassays [18–19]. The Openbabel FP2 fingerprint was used as a descriptor to assess similarities between molecules [20]. Tanimoto coefficients were calculated between the compounds of the β-carboline library and the ChEMBL database, and only β-carboline compounds with a Tc greater than 0.60 were considered for bioactivity analysis. A lower Tc threshold was used to identify a larger number of bioactivity targets. Table 4 lists the most promising bioactive targets for the newly synthesized β-carbolines together with the structurally similar lead compounds in ChEMBL along with their reported potency and literature citations. Several interesting results emerge from the comparison study performed, including a number of targets that the compounds should be screened against, such as C–C chemokine receptor type 3, gamma-aminobutyric acid receptor subunit gamma-2, breast adenocarcinoma cells, 5-hydroxytryptamine receptor 6, angiotensin-converting enzyme, and DNA polymerase iota. Moreover, nine of the twelve compounds are represented by allene precursors, ones that were not originally considered in the diversity analysis.

**Table 4:** Potential targets of β-carbolines based upon bioactivity data in ChEMBL.

target	CHEMBL compound	bioactivity type and reference	our compound	similarity score

C–C chemokine receptor type 3	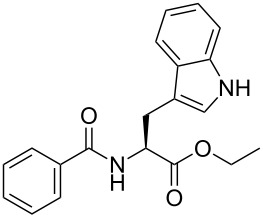 CHEMBL33838	IC_50_ = 325 nM [21]	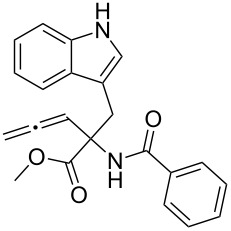 **A**	0.79
Leishmania donovani	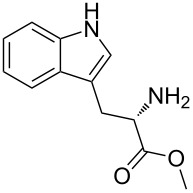 CHEMBL55830	IC_50_ = 1.42 µM [22]	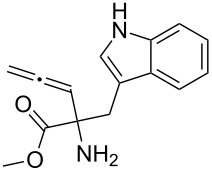 **4**	0.80
Acanthocheilonema viteae	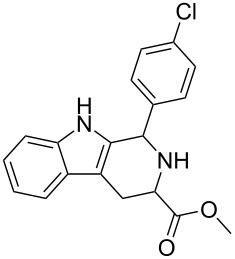 CHEMBL44573	Activity = 94% [23]	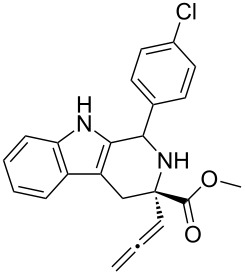 **6{5}**	0.86
Gamma-aminobutyric acid receptor subunit gamma-2	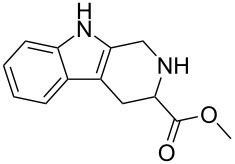 CHEMBL358326	IC_50_ = 250 nM [24]	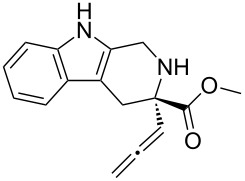 **6{1}**	0.84
5-Hydroxytryptamine receptor 6	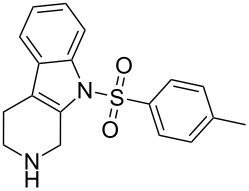 CHEMBL370935	*K*_i_ = 271.3 nM [25]	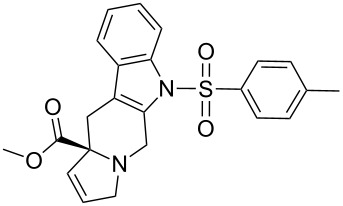 **7{1}**	0.69
3-Hydroxyacyl-CoA dehydrogenase type-2	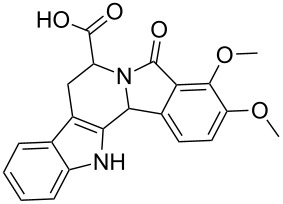 CHEMBL1382101	Potency = 31.6 µM PubChem AID:893	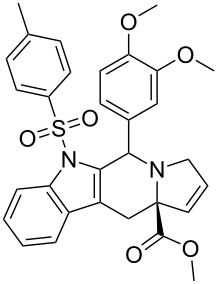 **7{7}**	0.64
Benzodiazepine receptors	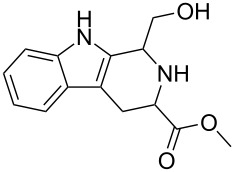 CHEMBL11901	*K*_i_ = 510 nM [26]	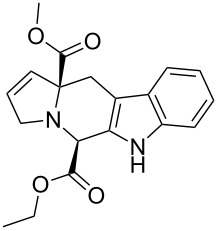 **1{16}**	0.72
Angiotensin-converting enzyme	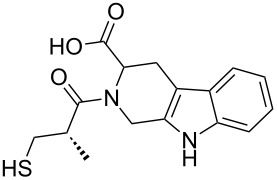 CHEMBL148616	IC_50_ = 500 nM [27]	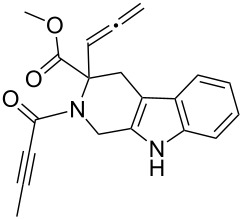 **9{1,3}**	0.71
Antithrombotic potency	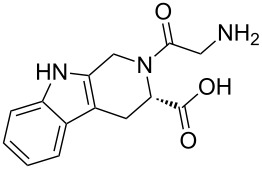 CHEMBL1089460	IC_50_ = 8.56 nM [28]	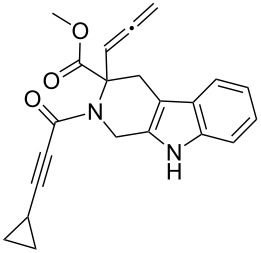 **9{1,4}**	0.69
Mitogen-activated protein kinase 1	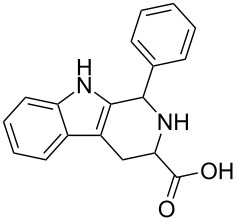 CHEMBL44295	Potency = 0.794 µM PubChem AID:995	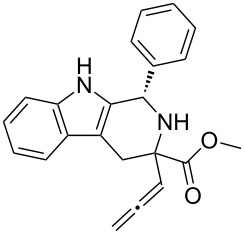 **6{4}**	0.82
Breast adenocarcinoma cells	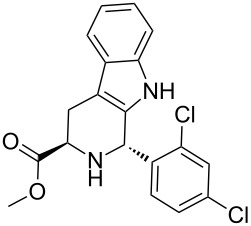 CHEMBL1650665	Inhibition = 78% [29]	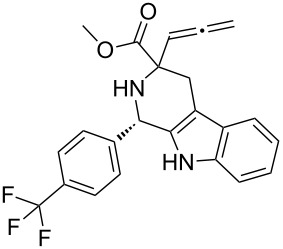 **6{6}**	0.79
DNA polymerase iota	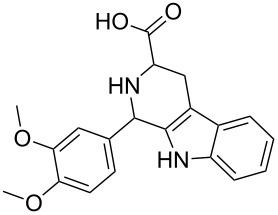 CHEMBL1360719	Potency = 1.78 µM PubChem AID:588590	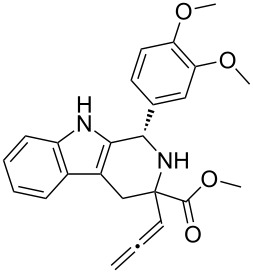 **6{7}**	0.85

## Conclusion

A library of 34 β-carboline-containing compounds was synthesized utilizing a skeletal diversification strategy. High-throughput docking and ligand-based protocols were implemented to predict potential biological targets of the newly synthesized β-carbolines. The docking approach uses a structure-based technology to predict preferred interactions between compounds and protein targets, whereas the ligand-based method uses ligand similarity coefficients to identify potential biological targets. The complementary nature of these two protocols is evidenced by the fact that there was no overlap in the predicted biological targets. Furthermore, the in silico screening of these compounds is intended to add value to the library, by directing them to appropriate biological assays. Such strategies can also be used to explore the mechanisms of a biologically active compound in bioassays whose molecular target is as of yet unidentified.

## Supporting Information

File 1 Experimental procedures and spectral data for compounds **1{5}**, **1{16}**, **2{1,3**–**8}**, **3{1,2**–**4}**, **4**, **6{1**–**10}**, **6{12**–**13}**, **6{15}**, **7{1**–**7}**, **9{1,2**–**4}**.

File 2 The complete listing of the proteins and their PDB IDs (Targets Docking Score Matrix).
