# Supplementary material for: Synthesis and in silico screening of a library of β-carboline-containing compounds
Source: Beilstein J Org Chem. 2012 Jul 10;8:1048–58. doi: 10.3762/bjoc.8.117 (PMC3458722; doi:10.3762/bjoc.8.117)
Supplement: File 1 — Experimental procedures and spectral data for compounds 1{5}, 1{16}, 2{1,3–8}, 3{1,2–4}, 4, 6{1–10}, 6{12–13}, 6{15}, 7{1–7}, 9{1,2–4}. [file Beilstein_J_Org_Chem-08-1048-s001.pdf]

Supporting Information

for

Synthesis and in silico screening of a library of β-carboline-containing compounds

Kay M. Brummond[1]*, John R. Goodell[1], Matthew G. LaPorte[1]*, Lirong Wang[2] and Xiang-Qun Xie[2]*

Address: [1]Department of Chemistry, Chemical Methodologies and Library Development, University of Pittsburgh, Pittsburgh, Pennsylvania 15260 and [2]Department of Pharmaceutical Sciences and Computational Chemical Genomics Center, School of Pharmacy; Drug Discovery Institute; Department of Computational Biology; University of Pittsburgh, Pittsburgh, Pennsylvania 15260

Email: Kay M. Brummond - kbrummon@pitt.edu, Matthew G. LaPorte - mgl12@pitt.edu, Xiang-Qun Xie - xix15@pitt.edu

*Corresponding author

Experimental procedures and spectral data for compounds **1{5}**, **1{16}**, **2{1,3–8}**, **3{1,2–4}**, **4**, **6{1–10}**, **6{12–13}**, **6{15}**, **7{1–7}**, **9{1,2–4}**.

Table of Contents

General S2

Preparation of **1{5}**, **1{16}**, **2{1,3–8}**, **3{1,2–4}**, **4**, **6{1–10}**, **6{12–13}**, **6{15}**, **7{1–7}**, **9{1,2–4}** S2

Spectral data S23

General. All reactions were performed under an argon atmosphere and all glassware was dried in an oven at 140 °C for 24 h prior to use. THF, CH_2Cl_2 and toluene were purified using an alumina column filtration system. DMF, MeOH, acetone and CH_3CN were dried over activated 3 or 4 Å molecular sieves. Et_3N was distilled from CaH_2 and stored over KOH. Reactions were monitored by TLC analysis (precoated silica gel 60 F254 plates, 250 μm layer thickness) and visualization was accomplished with a 254 nm UV light and by staining with a Vaughn's reagent (4.8 g of $(NH_4)_6Mo_7O_{24}\cdot 4H_2O$ and 0.2 g of $Ce(SO_4)_2$ in 100 mL of a 3.5 N H_2SO_4 solution) or a $KMnO_4$ solution (1.5 g of $KMnO_4$ and 1.5 g of K_2CO_3 in 100 mL of a 0.1% NaOH solution). Flash chromatography or preparative TLC on SiO_2 was used to purify the crude reaction mixtures. $^{1}H/^{13}C$ NMR spectra were recorded on either a Bruker Avance 300/75 MHz or Bruker Avance 400/100 MHz instrument. Chemical shifts were reported in parts per million with the residual solvent peak used as an internal standard. Chemical shifts were tabulated as follows: Chemical shift, multiplicity (s = singlet, d = doublet, t = triplet, q = quartet, m = multiplet, ovrlp = overlapping, app = apparent), coupling constants, and integration. Microwave reactions were performed using a Biotage Initiator in glass microwave vials (cap sealed) with continuous magnetic stirring and an external surface temperature sensor. Mass spectra were obtained on a Micromass Autospec double focusing instrument. Infrared spectra were measured on a Smiths Detection IdentifyIR FTIR spectrometer (ATR). Melting points (uncorrected) were determined using a Mel-Temp instrument.

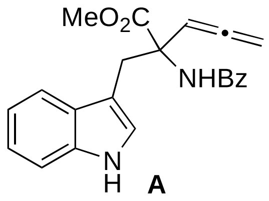

A

(±)-Methyl 2-((1*H*-indol-3-yl)methyl)-2-benzamidopenta-3,4-dienoate (A) [S1]

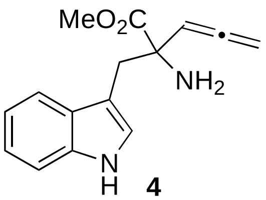

4

(±)-Methyl 2-((1*H*-indol-3-yl)methyl)-2-aminopenta-3,4-dienoate (4) [S1]

To a solution of benzamide **A** (3.60 g, 9.93 mmol) in CH_2Cl_2 (180 mL) was added Et_3OBF_4 (3.77 g, 19.9 mmol) in CH_2Cl_2 (14 mL). The mixture was stirred at rt overnight. The reaction was concentrated in vacuo and diluted with THF (8 mL) and 5% acetic acid (aq) (70 mL) and stirred for 4 h. The reaction was quenched with the addition of saturated K_2CO_3 and stirred for 10 min. The resulting mixture was diluted with water and extracted with EtOAc (3 x 30 mL). The combined organic layers were washed with brine, dried over $MgSO_4$, filtered, and concentrated in vacuo. The resulting residue was purified by chromatography (SiO_2, ISCO-Rf, 50–80% EtOAc:hexane) to afford amine **4** (1.20 g, 47%) as a viscous brown oil. R_f 0.23 (hexane:EtOAc, 1:4), ^{1}H NMR (300 MHz, $CDCl_3$) δ 8.30 (br s, 1 H), 7.67 (d, J = 7.8 Hz, 1 H), 7.35 (d, J = 8.1 Hz, 1 H), 7.29–7.08 (m, 3 H), 5.63 (t, J = 6.6 Hz, 1 H), 4.97 (d, J = 6.6 Hz, 2 H), 3.71 (s, 3 H), 3.47 (A of an ABq, J = 14.1 Hz, 1 H), 3.15 (B of an ABq, J = 14.1 Hz, 1 H), 1.91 (br s, 2 H); ^{13}C NMR (100 MHz, $CDCl_3$) δ 206.4, 175.5, 135.9, 127.9, 123.7, 121.8, 119.4, 119.0, 111.1, 109.7, 96.3, 79.4, 61.0, 52.3, 35.6.

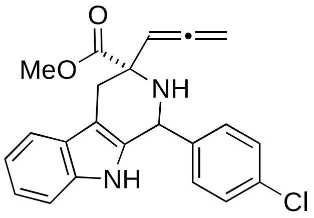

6{5}

(±)-(3*S*)-Methyl 1-(4-chlorophenyl)-3-(propa-1,2-dien-1-yl)-2,3,4,9-tetrahydro-1*H*-pyrido [3,4-*b*]indole-3-carboxylate (6{5}). To a solution of amine **4** (0.100 g, 0.390 mmol) in MeOH (4 mL) was added activated 4 Å molecular sieves (185 mg, 470 mg/mmol), 4-chlorobenzaldehyde (0.055 g, 0.390 mmol), and trifluoroacetic acid (0.029 mL, 0.390 mmol). After 3 h, the reaction was filtered through Celite and the filtrate was treated with saturated $NaHCO_3$ (~1 mL) and stirred for 5 min. The resulting solution was extracted with CH_2Cl_2 (3 x

5 mL). The organic fractions were combined, washed with brine, dried over $MgSO_4$, filtered, and concentrated in vacuo. The resulting residue was purified by flash chromatography (SiO_2, 10–20% EtOAc:hexane) to afford **6{5}** (77.5 mg, 52%, dr 2:1 by ^{1}H NMR) as an off-white amorphous solid, which turned yellow upon storage. R_f 0.49 (hexane:EtOAc, 4:1); ^{1}H NMR (400 MHz, $CDCl_3$) δ 7.58–7.53 (m, 1.5 H), 7.41–7.29 (m, 7.5 H), 7.25–7.18 (m, 1.5 H), 7.18–7.09 (m, 3 H), 5.51–5.46 (m, 1 H), 5.42 (t, *J* = 1.9 Hz, 0.5 H)*, 5.35 (t, *J* = 6.7 Hz, 0.5 H)*, 5.23 (t, *J* = 2.0 Hz, 1 H), 5.01 (d, *J* = 6.0 Hz, 1 H)*, 4.91 (dd, *J* = 11.3, 6.5 Hz, 1 H), 4.73 (dd, *J* = 11.3, 6.5 Hz, 1 H), 3.83 (s, 3 H), 3.67–3.61 (m, 2 H)*, 3.30–3.24 (m, 2 H), 3.04 (dd, *J* = 15.2, 2.6 Hz, 0.5 H)*, 2.80 (br s, 1 H). * Denotes *syn*-product.

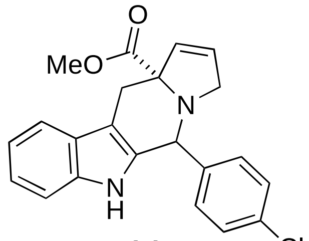

1{5}

(±)-Methyl 5-(4-chlorophenyl)-5,6,11,11a-tetrahydro-3*H*-indolizino[6,7-*b*]indole-11a-carboxylate (1{5}). To a solution of allene **6{5}** (70 mg, 0.185 mmol, as a 2:1 mixture of diastereomers) in acetone (3.6 mL) in a septum-sealed 10 mL reaction vial wrapped in aluminum foil, was added silver nitrate (6.3 mg, 0.037 mmol, 20 mol %, weighed and transferred under low light). The reaction mixture was protected from light and stirred for 18 h. The reaction mixture was diluted with CH_2Cl_2 and washed with saturated $NaHCO_3$ (2 x). The combined aqueous layers were back-extracted with CH_2Cl_2 (2 x) and the combined organic layers dried over $MgSO_4$, filtered, and concentrated in vacuo. The residue was purified using flash chromatography (SiO_2, hexane:acetone, 20:1) to afford **1{5}-*syn*** (13.4 mg, 19%) and **1{5}-*anti*** (16.4 mg, 23%) as amorphous white residues, which turned yellow during concentration. **1{5}-*syn***: R_f 0.1 (hexane:acetone, 20:1); ^{1}H NMR (300 MHz, $CDCl_3$) δ 7.58–7.55 (m, 1 H), 7.43–7.31 (m, 5 H), 7.22–7.16 (m, 1 H), 7.15–7.12 (m, 2 H), 6.14–6.11 (m, 1 H), 6.04–6.01 (m, 1 H), 5.50 (s, 1 H), 3.82–3.69 (m, 2 H), 3.57 (s, 3 H), 3.47 (d, *J* = 13.2 Hz, 1 H), 3.01 (dd, *J* = 14.3, 2.8 Hz, 1 H); LCMS *m*/*z*: 379.4 (t_R = 10.13 min); **1{5}-*anti***: R_f 0.07 (hexane:acetone, 20:1); ^{1}H NMR

(300 MHz, $CDCl_3$) δ 7.60–7.54 (m, 3 H), 7.44–7.34 (m, 3 H), 7.28–7.22 (m, 1 H), 7.15–7.12 (m, 2 H), 6.04–5.96 (m, 2 H), 5.04 (s, 1 H), 4.12 (d, J = 13.9 Hz, 1 H), 3.87–3.77 (m, 2 H), 3.47 (s, 3 H), 2.79 (dd, J = 14.7, 1.5 Hz, 1 H).

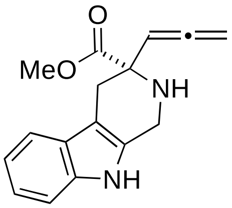

6{1}

(±)-Methyl 3-(propa-1,2-dien-1-yl)-2,3,4,9-tetrahydro-1*H*-pyrido[3,4-*b*]indole-3-carboxylate (6{1}). To a solution of amine **4** (0.763 g, 2.98 mmol) in MeOH (18 mL) was added formaldehyde 37% (0.268 mL, 3.57 mmol), and trifluoroacetic acid (0.22 mL, 2.98 mmol). The reaction mixture was stirred at rt. After 16 h, the reaction mixture was quenched with saturated $NaHCO_3$ (~12 mL) and stirred for 5 min. The resulting solution was extracted with CH_2Cl_2 (3 x 10 mL). The combined organic fractions were washed with brine, dried over $MgSO_4$, filtered, and concentrated in vacuo. The resulting residue was purified by chromatography (SiO_2, ISCO-Rf, 50–80%, EtOAc:hexane) to afford **6{1}** (545 mg, 68%) as a yellow/tan amorphous oil/foam. R_f 0.23 (hexane:EtOAc, 1:4); ^{1}H NMR (300 MHz, $CDCl_3$) δ 7.90 (s, 1 H), 7.52 (d, J = 6.9 Hz, 1 H), 7.29–7.19 (m, 1 H), 7.17–7.12 (m, 2 H), 5.40 (dd, J = 6.6, 13.2 Hz, 1 H), 4.96 (dd, J = 6.9, 11.4 Hz, 1 H), 4.88 (dd, J = 6.4, 11.1, 1 H), 4.16 (A of an ABq, J = 15.6 Hz, 1 H), 4.10 (B of an ABq, J = 15.6 Hz, 1 H), 3.73 (s, 3 H), 3.38 (A of an ABq, J = 15.3 Hz, 1 H), 3.04 (B of an ABq, J = 15.6 Hz, 1 H), 2.51 (br s, 1 H); ^{13}C NMR (100 MHz, $CDCl_3$) δ 207.2, 173.9, 136.2, 130.9, 127.2, 121.4, 119.2, 117.8, 110.7, 106.6, 94.0, 79.3, 60.6, 52.5, 39.8, 29.7.

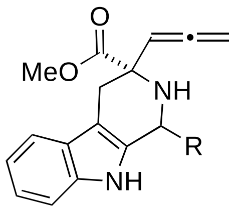

6{2-16}

General procedure for (±)-allenes 6{2–16}. To a solution of amine **4** (0.076 g, 0.297 mmol) in CH_2Cl_2 (2 mL) was added aldehyde (0.322 mmol, 1.1 equiv), and trifluoroacetic acid (0.024 mL, 0.322 mmol, 1.1 equiv). The reaction mixture was stirred 2 h at rt. The reaction was quenched with saturated $NaHCO_3$ (~1 mL) and stirred for 5 min. The resulting solution was extracted with CH_2Cl_2 (3 x 5 mL). The combined organic fractions were washed with brine, dried over $MgSO_4$, filtered, and concentrated in vacuo. The resulting residue was purified by chromatography (SiO_2, ISCO-Rf, variable EtOAc:hexane gradients) to afford allenes. Products were characterized by ^{1}H NMR (see spectral data) to determine dr then used directly in next reaction step.

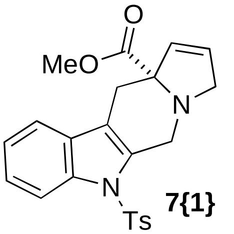

7{1}

General procedure 7{1–7}: (±)-Methyl 6-tosyl-5,6,11,11a-tetrahydro-3*H*-indolizino[6,7-*b*]indole-11a-carboxylate (7{1}). To a solution of allene **6{1}** (43 mg, 0.16 mmol) in acetone (2 mL) in a septum-sealed reaction vial wrapped in aluminum foil, was added silver nitrate (5.0 mg, 0.032 mmol, 20 mol %, weighed and transferred under low light). The reaction was then degassed by bubbling argon for 15 min. The reaction mixture was protected from light and stirred at rt for 18 h. The reaction mixture was diluted with CH_2Cl_2, washed with saturated $NaHCO_3$, brine, dried over MgSO4, filtered, and concentrated in vacuo. The resulting residue was passed through a short column (SiO_2, EtOAc:hex, 1:1) to afford the crude cyclized product, which was used directly in the next step [S2]. To a dry 1 dram vial was added dissolved carboline (0.162 mmol) in CH_2Cl_2 (0.25 mL) followed by addition of crushed NaOH (10 mg, 0.26 mmol), TEBA (4 mg, 0.016 mmol), and *p*-toluene sulfonyl chloride (37 mg, 0.19 mmol). The reaction

mixture was sonicated at rt for 1 h. The reaction was filtered through a short silica plug, washed with hexane:EtOAc (1:1), and concentrated in vacuo. The resulting residue was purified by flash chromatography (SiO_2, hexane:EtOAc, 4:1–1:1) followed by preparative TLC (EtOAc:hexane, 1:1)) to afford tosylated carboline **7{1}** (19.4 mg, 28%) as a pale yellow amorphous foam/oil. R_f 0.85 (hexane:EtOAc, 1:2); IR (neat): 1724, 1450, 1364, 1215, 1167, 1152, 1122, 1088, 973, 755, 745, 703, 660 cm^{-1}; ^{1}H NMR (400 MHz, $CDCl_3$) δ 8.09 (d, *J* = 8.0 Hz, 1 H), 7.68 (d, *J* = 8.0 Hz, 2 H), 7.40 (d, *J* = 8.0 Hz, 1 H), 7.32–7.26 (m, 1 H), 7.25–7.23 (m, 1 H), 7.19 (d, *J* = 8.4 Hz, 2 H), 6.22 (dd, *J* = 2.0, 6.0, 1 H), 5.99 (d, *J* = 6.0, 1 H), 4.62 (A of an ABq, *J* = 16.4 Hz, 1 H), 4.45 (B of an ABq, *J* = 16.8 Hz, 1 H), 4.04 (A of an ABq, *J* = 13.2 Hz, 1 H), 3.93 (B of an ABq, *J* = 12.8 Hz, 1 H), 3.53 (ovlp s, 3 H), 3.55 (ovlp A of an ABq, *J* = 15.2 Hz, 1 H), 2.74 (B of an ABq, *J* = 15.2 Hz, 1 H), 2.34 (s, 3 H); ^{13}C NMR (100 MHz, $CDCl_3$) δ 173.9, 144.7, 136.4, 135.5, 133.6, 132.0, 131.2, 130.0, 129.8, 129.7, 126.5, 126.4, 124.3, 123.3, 118.3, 116.4, 114.2, 71.8, 56.9, 51.7, 45.4, 31.6, 29.5, 22.6, 21.5, 14.1; HRMS (TOF-ES^+) (*m*/*z*): $[M + H]^+$ calcd for $C_{23}H_{23}N_2O_4S$, 423.1379; found, 423.1378.

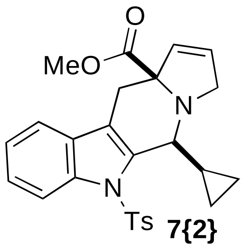

7{2}

(±)-Methyl 5-cyclopropyl-6-tosyl-5,6,11,11a-tetrahydro-3*H*-indolizino[6,7-*b*]indole-11a-carboxylate (7{2})). Followed general procedure. The resulting residue was purified by flash chromatography (SiO_2, hexane:EtOAc, 4:1–1:1) followed by prep TLC (EtOAc:hexane, 1:1) to afford **7{2}- *syn*** (17.2 mg, 0.037 mmol, 17%) as a yellow/tan amorphous oil/foam. R_f 0.5 (hexane:EtOAc, 1:1); ^{1}H NMR (400 MHz, $CDCl_3$) δ 8.16 (d, *J* = 8.4 Hz, 1 H), 7.65 (d, *J* = 8.4 Hz, 1 H), 7.42 (d, *J* = 7.6 Hz, 1 H), 7.33–7.28 (m, 2 H), 7.18 (d, *J* = 8.0 Hz, 2 H), 5.68 (s, 2 H), 4.48 (d, *J* = 7.2 Hz, 1 H), 3.96 (A of an ABq, *J* = 14.0 Hz, 1 H), 3.82–3.81 (m, 4 H), 3.49 (A of an ABq, *J* = 16.8 Hz, 1 H), 3.10 (B of an ABq, *J* = 14.0 Hz, 1 H), 2.99 (B of an ABq, *J* = 16.8 Hz, 1 H), 2.34 (s, 3 H), 1.31–0.88 (m, 5 H), 0.59–0.54 (m, 3 H), 0.40–0.38 (m, 1 H);

^{13}C NMR (100 MHz, $CDCl_3$) δ 175.7, 144.8, 136.5, 136.4, 136.1, 133.6, 129.7, 127.7, 126.4, 124.5, 123.6, 118.2, 117.6, 115.0, 71.7, 61.1, 59.3, 52.9, 27.6, 21.5, 16.4, 4.0, 3.7; IR (neat): 1724, 1448, 1366, 1213, 1167, 1146, 1088, 1053, 1023, 744, 704, 665 cm^{-1}; HRMS (TOF-ES^+) (*m*/*z*): $[M + H]^+$calcd for $C_{26}H_{27}N_2O_4S$, 463.1692; found, 463.1699.

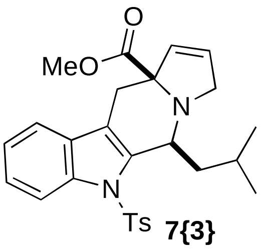

7{3}

(±)-Methyl 5-isobutyl-6-tosyl-5,6,11,11a-tetrahydro-3*H*-indolizino[6,7-*b*]indole-11a-carboxylate (7{3}). Followed general procedure. The resulting residue was purified by flash chromatography (SiO_2, hexane:EtOAc, 4:1–1:1) followed by prep. TLC (EtOAc:hexane, 1:1) to afford **7{3}-*syn*** (25 mg, 0.053 mmol, 23%) as a yellow/tan amorphous oil/foam. R_f 0.33 (hexane:EtOAc, 4:1); ^1H NMR (400 MHz, $CDCl_3$) δ 8.18 (d, *J* = 8.0 Hz, 1 H), 7.64 (d, *J* = 8.4 Hz, 2 H), 7.40–7.34 (m, 1 H), 7.30 (td, *J* = 1.4, 7.7 Hz, 1 H), 7.27–7.21 (m, 1 H), 7.19 (d, *J* = 8.4 Hz, 2 H), 5.79–5.76 (m, 1 H), 5.75–5.63 (m, 1 H), 4.62 (dd, *J* = 10.8, 3.5 Hz, 1 H), 4.04 (A of an ABq, *J* = 13.6 Hz, 1 H), 3.79 (s, 3 H), 3.50 (A of an ABq, *J* = 16.4 Hz, 1 H), 3.32 (B of an ABq, *J* = 14.0 Hz, 1 H), 2.83 (B of an ABq, *J* = 16.4 Hz, 1 H), 2.35 (s, 3 H), 2.21–2.09 (m, 1 H), 1.57–1.51 (m, 2 H), 1.13 (d, *J* = 6.8 Hz, 3 H), 0.99 (d, *J* = 6.8 Hz, 3 H); ^{13}C NMR (100 MHz, $CDCl_3$) δ 175.5, 144.7, 138.2, 136.7, 135.9, 133.7, 129.9, 129.7, 127.6, 126.3, 124.3, 123.5, 118.1, 116.4, 114.9, 70.4, 61.9, 55.0, 52.7, 43.9, 26.7, 24.8, 23.7, 21.5, 20.9; IR (neat): 1726, 1450, 1364, 1213, 1169, 1148, 1088, 745, 665 cm^{-1}; HRMS (TOF-ES^+) (*m*/*z*): $[M + H]^+$ calcd for $C_{27}H_{31}N_2O_4S$, 479.2005; found, 479.2004.

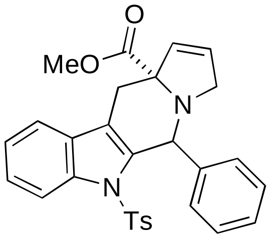

7{4}

(±)-Methyl 5-phenyl-6-tosyl-5,6,11,11a-tetrahydro-3*H*-indolizino[6,7-*b*]indole-11a-carboxylate (7{4}). Followed general procedure. The resulting residue was purified by chromatography (SiO_2, ISCO-Rf, 10–30% EtOAc:hex) to afford **7{4}-*syn*** (33 mg, 28%) and **7{4}-*anti*** (13 mg, 11%) both as yellow amorphous foams.

7{4}-*syn:* R_f 0.31 (hexane:EtOAc, 4:1); mp 186–188 °C (decomp); IR (neat): 1737, 1450, 1370, 1185, 1172, 1120, 744, 701, 662 cm^{-1}; ^{1}H NMR (400 MHz, $CDCl_3$) δ 7.98–7.96 (m, 1 H), 7.48–7.45 (m, 1 H), 7.39 (d, *J* = 8.4 Hz, 2 H), 7.29–7.26 (m, 7 H), 7.06 (d, *J* = 8.0 Hz, 2 H), 6.10 (d, *J* = 6.0 Hz, 1 H), 5.95–5.94 (m, 1 H), 5.90 (s, 1 H), 3.84 (A of an ABq, *J* = 13.2 Hz, 1 H), 3.61 (A of an ABdq, *J* = 15.2, 2.0 Hz, 1 H), 3.57 (s, 3 H), 3.29 (B of an ABq, *J* = 13.2 Hz, 1 H), 2.99 (B of an ABdq, *J* = 14.8, 2.4 Hz, 1 H), 2.29 (s, 3 H); ^{13}C NMR (100 MHz, $CDCl_3$) δ 174.3, 144.0, 141.9, 137.9, 137.7, 134.9, 132.7, 130.3, 129.9, 129.4, 129.2, 127.9, 127.3, 126.8, 124.7, 123.6, 120.3, 118.6, 115.3, 73.0, 60.5, 54.7, 51.7, 30.7, 21.5; HRMS (TOF-ES$^+$) (*m*/*z*): [M + H]$^+$ calcd for $C_{29}H_{27}N_2O_4S$, 499.1692; found, 499.1688.

7{4}-*anti:* R_f 0.17 (hexane:EtOAc, 4:1); mp 166–168 °C (decomp); IR (neat): 1724, 1450, 1372, 1210, 1169, 1150, 1120, 1088, 1059, 977, 745, 703, 667 cm^{-1}; ^{1}H NMR (400 MHz, $CDCl_3$) δ 8.21 (d, *J* = 8.0 Hz, 1 H), 7.52 (d, *J* = 7.2 Hz, 1 H), 7.41–7.37 (m, 3 H), 7.37–7.30 (m, 1 H), 7.20–7.17 (m, 3 H), 7.06–7.01 (m, 4 H), 5.99 (s, 1 H), 5.92 (dt, *J* = 6.0, 1.6 Hz, 1 H), 5.76–5.74 (m, 1 H), 4.31 (A of an ABq, *J* = 13.2 Hz, 1 H), 3.77 (B of an ABq, *J* = 13.6 Hz, 1 H), 3.61 (A of an ABq, *J* = 16.8 Hz, 1 H), 3.12 (s, 3 H), 2.91 (B of an ABq, *J* = 16.8 Hz, 1 H), 2.31 (s, 3 H); ^{13}C NMR (100 MHz, $CDCl_3$) δ 174.7, 144.5, 139.6, 136.4, 135.6, 134.1, 133.9, 129.4, 129.3, 128.5, 128.2, 127.9, 127.1, 126.6, 124.7, 123.4, 118.6, 118.2, 114.6, 69.7, 60.3, 58.9, 52.2, 27.2, 21.5; HRMS (TOF-ES$^+$) (*m*/*z*): [M + H]$^+$ calcd for $C_{29}H_{27}N_2O_4S$, 499.1692; found, 499.1693.

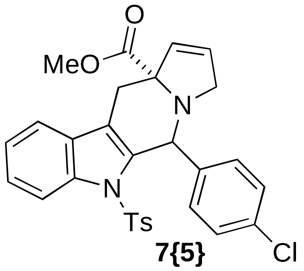

7{5}

(±)-Methyl 5-(4-chlorophenyl)-6-tosyl-5,6,11,11a-tetrahydro-3*H*-indolizino[6,7-*b*]indole-11a-carboxylate (7{5}). Followed general procedure. The resulting residue was purified by chromatography (SiO_2, ISCO-Rf, 10–30%, hexane:EtOAc) followed by preparative TLC (EtOAc:hexane, 1:1) to afford **7{5}-*syn*** (21 mg, 17%) and **7{5}-*anti*** (42 mg, 34%) both as yellowish foams.

7{5}-*syn:* R_f 0.36 (hexane:EtOAc, 4:1); mp 197–199 °C (decomp); IR (neat): 1728, 1448, 1368, 1211, 1169, 1118, 1085, 1014, 971, 813, 742, 667 cm^{-1}; ^1H NMR (400 MHz, $CDCl_3$) δ 8.00 (d, *J* = 7.6 Hz, 1 H), 7.48–7.46 (m, 1 H), 7.39 (d, *J* = 8.0 Hz, 2 H), 7.29 (d, *J* = 1.2 Hz, 3 H), 7.19 (br s, 3 H), 7.07 (d, *J* = 8.0 Hz, 2 H), 6.10 (d, *J* = 6.0 Hz, 1 H), 5.94 (d, *J* = 6.0 Hz, 1 H), 5.88 (s, 1 H), 3.80 (A of an ABq, *J* = 13.2 Hz, 1 H), 3.64–3.62 (m, 1 H), 3.57 (s, 3 H), 3.27 (B of an ABq, *J* = 13.2 Hz, 1 H), 2.97 (B of an ABdq, *J* = 16.0, 2.0 Hz, 1 H), 2.31 (s, 3 H); ^{13}C NMR (100 MHz, $CDCl_3$) δ 174.1, 144.2, 140.3, 137.8, 137.4, 134.9, 133.0, 132.6, 131.5, 130.8, 130.4, 130.3, 129.7, 129.2, 128.0, 126.5, 124.9, 123.7, 120.3, 118.7, 115.4, 73.0, 59.8, 54.6, 51.8, 30.6, 21.5; HRMS (TOF-ES$^+$) (*m*/*z*): $[M + H]^+$ calcd for $C_{29}H_{26}N_2O_4SCl$, 533.1302; found, 533.1292.

7{5}-*anti:* R_f 0.24 (hexane:EtOAc, 4:1); mp 125–127 °C (decomp); IR (neat): 1722, 1362, 1208, 1169, 1150, 1087, 1014, 809, 745, 662 cm^{-1}; ^1H NMR (400 MHz, $CDCl_3$) δ 8.24 (d, *J* = 8.4 Hz, 1 H), 7.53 (d, *J* = 7.2 Hz, 1 H), 7.52–7.39 (m, 3 H), 7.37–7.29 (m, 1 H), 7.11–7.09 (m, 2 H), 7.05 (d, *J* = 8.0 Hz, 2 H), 6.93 (d, *J* = 8.4 Hz, 2 H), 5.94–5.92 (m, 2 H), 5.74 (dt, *J* = 6.0, 1.9 Hz, 1 H), 4.30 (A of an ABtq, *J* = 13.6, 2.0 Hz, 1 H), 3.78 (B of an ABtq, *J* = 13.6, 2.0 Hz, 1 H), 3.59 (A of an ABq, *J* = 16.4 Hz, 1 H), 3.16 (s, 3 H), 2.88 (B of an ABq, *J* = 16.8 Hz, 1 H), 2.34 (s, 3 H); ^{13}C NMR (100 MHz, $CDCl_3$) δ 174.5, 144.7, 138.3, 136.6, 135.7, 133.8, 133.4, 132.8, 129.8, 129.5, 129.2, 128.4, 128.3, 127.9, 126.3, 124.9, 123.5, 118.7, 118.3, 114.6, 69.5, 60.1, 58.1, 52.1, 27.1, 21.5; HRMS (TOF-ES$^+$) (*m*/*z*): $[M + H]^+$ calcd for $C_{29}H_{26}N_2O_4SCl$, 533.1302; found, 533.1288.

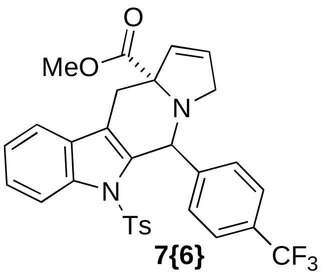

7{6}

(±)-Methyl 6-tosyl-5-(4-(trifluoromethyl)phenyl)-5,6,11,11a-tetrahydro-3*H*-indolizino[6,7-*b*]indole-11a-carboxylate (7{6}). Followed general procedure. The resulting residue was purified by chromatography (SiO_2, ISCO-Rf, 10–20% hexane:EtOAc) to afford **7{6}-*syn*** followed by preparative TLC (EtOAc:hexane, 1:1) (12 mg, 10%) and **7{6}-*anti*** (43.5 mg, 35%) both as yellowish solids.

7{6}-*syn*: R_f 0.28 (hexane:EtOAc, 4:1); mp 191–192 °C (decomp); IR (neat): 1731, 1374, 1323, 1170, 1116, 1066, 1018, 829, 744, 665 cm^{-1}; ^1H NMR (400 MHz, $CDCl_3$) δ 8.02–7.99 (m, 1 H), 7.49–7.48 (m, 3 H), 7.39 (d, *J* = 8.4 Hz, 3 H), 7.33–7.29 (m, 3 H), 7.06 (d, *J* = 8.4 Hz, 2 H), 6.11–6.09 (m, 1 H), 5.98–5.97 (m, 2 H), 3.82 (A of an ABq, *J* = 13.2 Hz, 1 H), 3.62 (A of an ABdq, *J* = 15.2, 2.0 Hz, 1 H), 3.58 (s, 3 H), 3.23 (B of an ABq, *J* = 12.8 Hz, 1 H), 2.97 (B of an ABdq, *J* = 14.8, 2.4 Hz, 1 H), 2.30 (s, 3 H); ^{13}C NMR (100 MHz, $CDCl_3$) δ 174.0, 145.8, 144.3, 137.8, 136.9, 134.9, 132.5, 130.3, 129.8, 129.7, 129.6, 129.2, 126.5, 125.1, 124.9, 124.8, 123.8, 120.5, 118.7, 115.4, 73.0, 60.1, 54.6, 51.8, 30.6, 21.4; HRMS (TOF-ES$^+$) (*m*/*z*): $[M + H]^+$ calcd for $C_{30}H_{26}N_2O_4SF_3$, 567.1565; found, 567.1563.

7{6}-*anti*: R_f 0.21 (hexane:EtOAc, 4:1); mp 148–150 °C (decomp); IR (neat): 1720, 1321, 1210, 1163, 1124, 1090, 1064, 808, 745, 673, 654 cm^{-1}; ^1H NMR (400 MHz, $CDCl_3$) δ 8.26 (d, *J* = 8.4 Hz, 1 H), 7.55 (d, *J* = 7.2 Hz, 1 H), 7.45–7.39 (m, 1 H), 7.38–7.34 (m, 5 H), 7.10 (d, *J* = 8.4 Hz, 2 H), 7.02 (d, *J* = 8.4 Hz, 2 H), 5.99 (s, 1 H), 5.95 (dt, *J* = 2.0, 6.4 Hz, 1 H), 5.78 (dt, *J* = 6.0, 2.0 Hz, 1 H), 4.32 (A of an ABtq, *J* = 13.2, 1.6 Hz, 1 H), 3.85 (B of an ABdq, *J* = 13.2, 2.0 Hz, 1 H), 3.61 (A of an ABq, *J* = 16.8 Hz, 1 H), 3.05 (s, 3 H), 2.89 (B of an ABq, *J* = 16.8 Hz, 1 H), 2.31 (s, 3 H); ^{13}C NMR (100 MHz, $CDCl_3$) δ 174.3, 144.7, 143.8, 136.8, 135.7, 133.7, 132.7, 129.5, 129.3, 129.1, 128.6, 128.2, 126.2, 125.1, 124.7, 124.6, 123.6, 118.7, 118.6,

114.7, 69.3, 60.2, 58.3, 52.0, 27.1, 21.4; HRMS (TOF-ES$^+$) (*m*/*z*): $[M + H]^+$ calcd for $C_{30}H_{26}N_2O_4SF_3$, 567.1565; found, 567.1558.

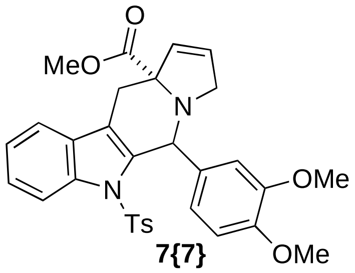

7{7}

(±)-Methyl 5-(3,4-dimethoxyphenyl)-6-tosyl-5,6,11,11a-tetrahydro-3*H*-indolizino[6,7-*b*]indole-11a-carboxylate (7{7}). Followed general procedure. The resulting residue was purified by chromatography (SiO_2, ISCO-Rf, 10–60% EtOAc:hexane) followed by preparative TLC (EtOAc:hexane, 1:1) to afford **7{7}-*syn*** (17 mg, 17%) and **7{7}-*anti*** (12 mg, 12%) both as yellowish residues.

7{7}-*syn*: R_f 0.58 (hexane:EtOAc, 1:1); IR (neat): 1722, 1511, 1448, 1372, 1264, 1234, 1169, 1139, 1088, 1025, 978, 811, 745, 665 cm^{-1}; ^{1}H NMR (400 MHz, $CDCl_3$) δ 8.02–7.99 (m, 1 H), 7.48–7.46 (m, 1 H), 7.37 (d, *J* = 8.4 Hz, 2 H), 7.30–7.27 (m, 3 H), 7.04 (d, *J* = 8.4 Hz, 2 H), 6.86 (br s, 1 H), 6.72 (d, *J* = 8.0 Hz, 1 H), 6.10 (app d, *J* = 6.0 Hz, 1 H), 5.95–5.90 (m, 1 H), 5.83 (s, 1 H), 3.87 (s, 3 H), 3.79–3.77 (m, 4 H), 3.62 (d, *J* = 2.0 Hz, 1 H), 3.59 (s, 3 H), 3.30 (A of an ABq, *J* = 13.2 Hz, 1 H), 2.96 (B of an ABq, *J* = 14.8 Hz, 1 H) 2.30 (s, 3 H); ^{13}C NMR (100 MHz, $CDCl_3$) δ 174.4, 148.2, 148.1, 143.9, 138.1, 137.7, 135.2, 134.3, 132.7, 130.3, 129.8, 129.0, 126.5, 124.7, 123.5, 119.7, 118.6, 115.3, 110.4, 73.0, 60.1, 55.8, 55.7, 54.8, 51.7, 30.7, 21.4; HRMS (TOF-ES$^+$) (*m*/*z*): $[M + H]^+$ calcd for $C_{31}H_{31}N_2O_6S$, 559.1903; found, 559.1900.

7{7}-*anti:* R_f 0.28 (hexane:EtOAc, 1:1); mp 148–150 °C (decomp); IR (neat): 1726, 1511, 1450, 1368, 1254, 1219, 1170, 1027, 809, 745, 660 cm^{-1}; ^{1}H NMR (400 MHz, $CDCl_3$) δ 8.23 (d, *J* = 8.4 Hz, 1 H), 7.52 (d, *J* = 7.2 Hz, 1 H), 7.42–7.29 (m, 4 H), 7.01 (d, *J* = 8.4 Hz, 2 H), 6.79–6.68 (m, 1 H), 6.58 (d, *J* = 8.4 Hz, 1 H), 6.36 (dd, *J* = 8.0, 1.8 Hz, 1 H), 5.92–5.90 (m, 2 H), 5.74–5.73 (m, 1 H), 4.24 (br s, 1 H), 3.85 (s, 3 H), 3.77 (s, 3 H), 3.76–3.70 (m, 1 H), 3.58 (A of an ABq, *J* = 16.4 Hz, 1 H), 3.21 (s, 3 H), 2.91 (B of an ABq, *J* = 16.4 Hz, 1 H), 2.31 (s, 3 H); ^{13}C NMR

(100 MHz, $CDCl_3$) δ 148.6, 148.1, 144.5, 136.4, 135.7, 133.9, 129.3, 128.3, 126.6, 124.7, 123.4, 120.5, 118.6, 117.8, 114.6, 111.9, 110.2, 60.2, 58.7, 55.8, 55.6, 52.4, 27.2, 21.5; HRMS (TOF-ES$^+$) (*m/z*): $[M + H]^+$ calcd for $C_{31}H_{31}N_2O_6S$, 559.1903; found, 559.1900.

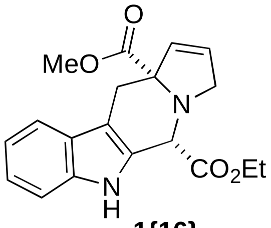

1{16}

(±)-(5*S*,11a*S*)-5-Ethyl 11a-methyl 5,6,11,11a-tetrahydro-3*H*-indolizino[6,7-*b*]indole-5,11a-dicarboxylate (1{16}). To a solution of allene **6{16}** (235 mg, 0.693 mmol, dr = 1.4/1) in acetone (8 mL) in a septum-sealed reaction vial wrapped in aluminum foil, was added silver nitrate (23 mg, 0.14 mmol, 20 mol %, weighed and transferred under low light). The reaction mixture was degassed with bubbling argon (15 min) and protected from light for 18 h. The reaction mixture was diluted with CH_2Cl_2 and washed with saturated $NaHCO_3$ (2 x). The combined organic layers were washed with brine, dried over $MgSO_4$, filtered, and concentrated in vacuo. The resulting residue was purified using chromatography (SiO_2, ISCO-Rf, 10–30% EtOAc/hexane) to afford **1{16}-*syn*** (18 mg, 8%) as a yellow foamy residue. R_f 0.68 (hexane:EtOAc, 1:1); mp 180–182 °C; IR (neat): 3344, 1750, 1705, 1318, 1200, 1163, 1016, 749 cm^{-1}; ^1H NMR (400 MHz, $CDCl_3$) δ 8.32 (br s, 1 H), 7.55 (d, *J* = 8.0 Hz, 1 H), 7.35 (d, *J* = 8.0 Hz, 1 H), 7.19 (td, *J* = 7.6, 1.1 Hz, 1 H), 7.16–7.12 (m, 1 H), 6.22 (dt, *J* = 6.0, 1.2 Hz, 1 H), 6.01 (dt, *J* = 6.0, 1.6 Hz, 1 H), 5.38 (s, 1 H), 4.41-4.31 (m, 2 H), 4.24 (A of an ABq, *J* = 13.2 Hz, 1 H), 3.86 (B of an ABq, *J* = 12.8 Hz, 1 H), 3.63 (A of an ABdq, *J* = 14.4, 1.6 Hz, 1 H), 3.53 (s, 3 H), 2.98 (B of an ABdq, *J* = 14.8, 2.8 Hz, 1 H), 1.39 (t, *J* = 7.2 Hz, 3 H); ^{13}C NMR (100 MHz, $CDCl_3$) δ 174.1, 170.4, 136.2, 132.1, 130.7, 129.0, 127.0, 122.1, 119.4, 118.4, 110.9, 109.0, 74.5, 61.7, 58.4, 54.8, 51.8, 28.8, 14.4; HRMS (TOF-ES$^+$) (*m/z*): $[M + H]^+$ calcd for $C_{19}H_{21}N_2O_4$, 341.1501; found, 341.1505.

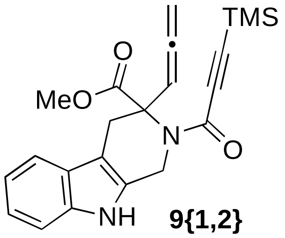

9{1,2}

(±)-Methyl 3-(propa-1,2-dien-1-yl)-2-(3-(trimethylsilyl)propioloyl)-2,3,4,9-tetrahydro-1*H*-pyrido[3,4-*b*]indole-3-carboxylate (9{1,2}). To a solution of 3-(trimethylsilyl)propiolic acid (159 mg, 0.112), amino ester **6{1}** (200 mg, 0.745 mmol), and PyBroP (276 mg, 0.112 mmol) in CH_2Cl_2 (2.0 mL) was added DIEA (519 µL, 2.98 mmol) at 0 °C. The ice bath was removed after 1–2 min and stirring continued at rt for 1 h. The mixture was poured into EtOAc (30 mL) and washed with 5% $NaHSO_4$, saturated $NaHCO_3$, brine, dried over $MgSO_4$, filtered, and concentrated in vacuo. The resulting residue was purified by chromatography (SiO_2, ISCO-Rf, 10–20% EtOAc:hexane) to afford amide **9{1,2}** (187 mg, 64%) as an amorphous residue. R_f 0.7 (hexane:EtOAc, 1:1); IR (neat): 3388, 1735, 1618, 1392, 1249, 1200, 841, 738 cm^{-1}; ^{1}H NMR (300 MHz, $CDCl_3$) δ 8.08 (s, 1 H), 7.49 (d, *J* = 7.8 Hz, 1 H), 7.35 (d, *J* = 7.8 Hz, 1 H), 7.28–7.17 (m, 1 H), 7.16–7.08 (m, 1 H), 5.46 (t, *J* = 6.6 Hz, 1 H), 5.35 (d, *J* = 16.2 Hz, 1 H), 4.81–4.71 (m, 2 H), 4.54 (B of an ABdq, *J* = 6.6, 11.4 Hz, 1 H), 3.83 (s, 3 H), 3.46 (A of an ABq, *J* = 15.9 Hz, 1 H), 3.22 (B of an ABq, *J* = 15.6 Hz, 1 H), 0.27–0.22 (m, 9 H); ^{13}C NMR (100 MHz, $CDCl_3$) δ 208.3, 171.0, 155.0, 136.3, 128.5, 127.0, 122.1, 119.7, 118.1, 110.9, 108.0, 99.0, 95.6, 90.6, 79.5, 62.6, 52.7, 44.2, 26.7, -0.7; HRMS (TOF-ES$^+$) (*m*/*z*): $[M + H]^+$ calcd for $C_{22}H_{25}N_2O_3Si$, 393.1634; found, 393.1639.

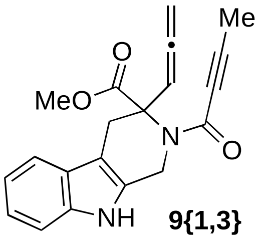

9{1,3}

(±)-Methyl 2-(but-2-ynoyl)-3-(propa-1,2-dien-1-yl)-2,3,4,9-tetrahydro-1*H*-pyrido[3,4-*b*] indole-3-carboxylate (9{1,3}). To a solution of but-2-ynoic acid (70 mg, 0.84), amino ester **6{1}** (150 mg, 0.559 mmol), and PyBroP (207 mg, 0.839 mmol) in CH_2Cl_2 (2.0 mL) was added DIEA

(389 µL, 2.24 mmol) at 0 °C. The ice bath was removed after 1–2 min and stirring continued at rt for 1 h. The mixture was poured into EtOAc (30 mL) and washed with 5% $NaHSO_4$, saturated $NaHCO_3$, brine, dried over $MgSO_4$, filtered, and concentrated in vacuo. The resulting residue was purified by chromatography (SiO_2, ISCO-Rf, 20–80% EtOAc:hexane) to afford **9{1,3}** (140 mg, 75%) as an amorphous residue and bis-allene (19 mg, 10%). R_f 0.24 (hexane:EtOAc, 1:1) (bis-allene, R_f 0.16); IR (neat): 3387, 2233,1731, 1612, 1390, 1372, 1251, 1183, 1042, 852, 740 cm^{-1}; ^{1}H NMR (300 MHz, $CDCl_3$) δ 8.37 (s, 1 H), 7.48 (d, J = 7.5 Hz, 1 H), 7.35 (d, J = 8.1Hz, 1 H), 7.21–7.14 (m, 1 H), 7.13–7.09 (m, 1 H), 5.47 (t, J = 6.3 Hz, 1 H), 5.35 (d, J = 16.5 Hz, 1 H), 4.80–4.56 (m, 2 H), 4.52 (B of an ABdq, J = 11.7, 6.6 Hz, 1 H), 3.82 (s, 3 H), 3.46 (A of an ABq, J = 15.6 Hz, 1 H), 3.22 (B of an ABq, J = 15.3 Hz, 1 H), 1.99 (s, 3 H); ^{13}C NMR (100 MHz, $CDCl_3$) δ 208.2, 171.3, 155.9, 136.4, 128.8, 126.9, 121.9, 119.5, 117.9, 111.0, 107.5, 90.8, 90.7, 79.4, 73.3, 62.6, 52.7, 44.2, 31.5, 26.9, 22.6, 14.1, 4.0; HRMS (TOF-ES$^+$) (m/z): $[M + H]^+$ calcd for $C_{20}H_{19}N_2O_3$, 335.1396; found, 335.1403.

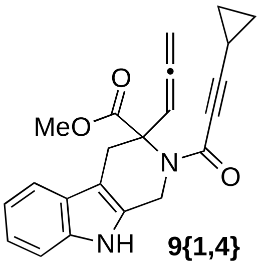

9{1,4}

(±)-Methyl 2-(3-cyclopropylpropioloyl)-3-(propa-1,2-dien-1-yl)-2,3,4,9-tetrahydro-1*H*-pyrido[3,4-*b*]indole-3-carboxylate (9{1,4}). To a solution of 3-cyclopropylpropiolic acid (0.123 g, 1.12 mmol), amino ester **6{1}** (0.200 g, 0.745 mmol), and PyBroP (0.276 g, 1.12 mmol) in CH_2Cl_2 (4.0 mL) was added DIEA (519 µL, 2.98 mmol) at 0 °C. The ice bath was removed after 1–2 min and stirring continued at rt for 3 h. The mixture was poured into EtOAc (30 mL) and washed with 5% $NaHSO_4$, saturated $NaHCO_3$, brine, dried over $MgSO_4$, filtered, and concentrated in vacuo. The resulting residue was purified using chromatography (SiO_2, ISCO-Rf, 20–50% EtOAc:hexane) to afford **9{1,4}** (191 mg, 71%) as an amorphous residue. R_f 0.39 (hexane:EtOAc, 1:1); IR (neat): 3373, 2216, 1731, 1610, 1390, 1331, 1252, 1178, 1042, 854, 738 cm^{-1}; ^{1}H NMR (300 MHz, $CDCl_3$) δ 8.26 (s, 1 H), 7.48 (d, J = 7.5 Hz, 1 H), 7.34 (d, J = 7.8 Hz,

1 H), 7.19–7.14 (m, 1 H), 7.13–7.11 (m, 1 H), 5.44 (t, *J* = 6.6 Hz, 1 H), 5.32 (d, *J* = 16.2 Hz, 1 H), 4.80–4.68 (m, 2 H), 4.51 (B of an ABdq, *J* = 11.4, 6.6 Hz, 1 H), 3.82 (s, 3 H), 3.43 (A of an ABq, *J* = 15.8 Hz, 1 H), 3.23 (B of an ABq, *J* = 15.9 Hz, 1 H), 1.38–1.28 (m, 2 H), 0.94–0.82 (m, 4 H); ^{13}C NMR (100 MHz, $CDCl_3$) δ 208.2, 171.3, 155.9, 136.3, 128.8, 127.0, 121.9, 119.6, 118.0, 110.9, 107.7, 98.3, 90.8, 79.4, 69.2, 62.5, 52.7, 44.2, 31.6, 26.8, 22.6, 14.1, 9.2, -0.4; HRMS (TOF-ES$^+$) (*m*/*z*): $[M + H]^+$ calcd for $C_{22}H_{21}N_2O_3$, 361.1552; found, 361.1556.

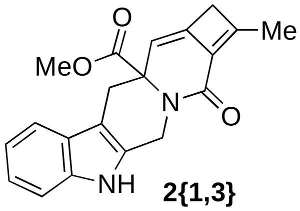

2{1,3}

(±)-Methyl 2-methyl-3-oxo-1,3,5,6,11,11a-hexahydrocyclobuta[*b*]indolo[2,3-*g*]quinolizine-11a-carboxylate (2{1,3}). Method A: To an oven-dried microwave vial (2–5 mL) was added **9{1,3}** (55 mg, 0.16 mmol) in DMF (1 mL). The reaction mixture was subjected to microwave irradiation at 160 °C for 7 min. The crude product was purified by chromatography (SiO_2, ISCO-Rf, 20–50% EtOAc:hexane) to afford the **2{1,3}** (40 mg, 73%) as a off-white/clear amorphous residue. R_f 0.39 (hexane:EtOAc, 1:1); mp 258–260 °C (decomp); IR (neat): 3338, 1731, 1664, 1621, 1381, 1329, 1256, 1195, 1165, 1046, 744 cm^{-1}; ^{1}H NMR (400 MHz, d_6-DMSO) δ 10.92 (s, 1 H), 7.43 (d, *J* = 7.6 Hz, 1 H), 7.32 (d, *J* = 8.0 Hz, 1 H), 7.09–7.01 (m, 1 H), 7.01–6.97 (m, 1 H), 5.40 (s, 1 H), 5.32 (A of an ABq, *J* = 17.6 Hz, 1 H), 4.22 (B of an ABq, *J* = 17.6 Hz, 1 H), 3.73 (A of an ABq, *J* = 15.6 Hz, 1 H), 3.54 (s, 3 H), 3.22 (br s., 2 H), 2.92 (B of an ABq, *J* = 15.6 Hz, 1 H), 2.18 (s, 3 H); ^{13}C NMR (100 MHz, d_6-DMSO) δ 171.3, 158.0, 154.0, 137.4, 136.8, 131.3, 130.2, 126.5, 121.6, 119.2, 118.2, 111.6, 107.0, 104.9, 70.7, 53.6, 32.9, 16.8; HRMS (TOF-ES$^+$) (*m*/*z*): $[M + H]^+$ calcd for $C_{20}H_{19}N_2O_3$, 335.1396; found, 335.1397.

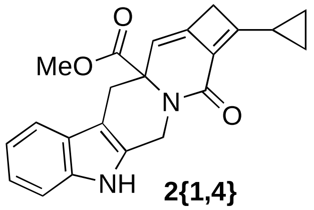

2{1,4}

(±)-Methyl 2-cyclopropyl-3-oxo-1,3,5,6,11,11a-hexahydrocyclobuta[*b*]indolo[2,3-*g*]quinolizine-11a-carboxylate (2{1,4}). Method A: To an oven-dried microwave vial (2–5 mL) was added **9{1,4}** (50 mg, 0.14 mmol) in DMF (1 mL). The reaction mixture was subjected to microwave irradiation at 160 °C for 7 min. The crude product was purified by chromatography (SiO_2, ISCO-Rf, 30–50% EtOAc:hexane) to afford the **2{1,4}** (28 mg, 57%) as an off-white solid. R_f 0.47 (hexane:EtOAc, 1:1); mp 239–240 °C (decomp); IR (neat): 3271, 1728, 1648, 1612, 1333, 1202, 1169, 1042, 745, 660 cm^{-1}; ^1H NMR (400 MHz, $CDCl_3$) δ 9.09 (s, 1 H), 7.52 (d, J = 7.6 Hz, 1 H), 7.38 (d, J = 8.0 Hz, 1 H), 7.20–7.16 (m, 1 H), 7.15–7.11 (m, 1 H), 5.59 (A of an ABq, J = 17.2 Hz, 1 H), 5.28 (s, 1 H), 4.55 (B of an ABq, J = 17.2 Hz, 1 H), 3.82 (A of an ABq, J = 16.4 Hz, 1 H), 3.59 (ovlp B of an ABq, J = 16.3 Hz, 1 H), 3.63 (ovlp s, 3 H), 3.11–3.05 (m, 2 H), 2.09–1.99 (m, 1 H), 1.18–1.12 (m, 4 H); ^{13}C NMR (100 MHz, $CDCl_3$) δ 171.3, 160.4, 158.5, 136.7, 136.4, 129.6, 128.2, 126.4, 121.6, 119.2, 117.9, 111.0, 105.5, 105.5, 70.8, 53.0, 39.7, 37.1, 33.0, 12.9, 10.1, 10.0; HRMS (TOF-ES$^+$) (m/z): $[M + H]^+$ calcd for $C_{22}H_{21}N_2O_3$, 361.1552; found, 361.1544.

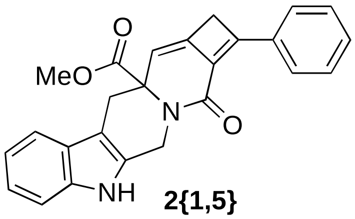

2{1,5}

(±)-Methyl 3-oxo-2-phenyl-1,3,5,6,11,11a-hexahydrocyclobuta[*b*]indolo[2,3-*g*]quinolizine-11a-carboxylate (2{1,5}).

Method A: To an oven-dried microwave vial (2–5 mL) was added **9{1,5}** (76 mg, 0.19 mmol) in DMF (1 mL). The reaction mixture was subjected to microwave irradiation at 160 °C for 7 min. The crude product was purified by chromatography (SiO_2, ISCO-Rf, DCM:acetone, 39:1) to afford **2{1,5}** as an off-white solid.

Method B: To a solution of 3-phenylpropiolic acid (73.5 mg, 0.503 mmol), amino ester **6{1}** (90 mg, 0.335 mmol), and PyBroP (124 mg, 0.503 mmol) in CH_2Cl_2 (1.0 mL) was added DIEA (234 µL, 1.34 mmol) under stirring at 0 °C. The ice bath was removed after 1–2 min and stirring continued at rt for 1 h. The mixture was poured into EtOAc (15 mL) and washed with 5% $NaHSO_4$, saturated $NaHCO_3$ and brine, then dried over $MgSO_4$, filtered, and concentrated in vacuo. The resulting residue was filtered through a short plug of silica (eluted with EtOAc:hexane, 1:1) to isolate a mixture of allene-yne **9{1,5}** and cyclobutene **2{1,5}** (91 mg combined). The mixture was resuspended in CH_2Cl_2 (4 mL) and placed in front of two 6 W (λ = 254 nm) lamps for 24 h without stirring at rt. The reaction mixture was concentrated and purified using flash chromatography (SiO_2, CH_2Cl_2:acetone, 39:1) to afford the cyclobutene **2{1,5}** (53.3 mg, 40%) as a white solid. R_f 0.28 (CH_2Cl_2:acetone, 39:1); mp 291–293 °C (decomp); IR (neat): 3237, 1735, 1646, 1605, 1333, 1264, 1239, 1197, 1157, 1308, 757, 734. 686 cm^{-1}; ^{1}H NMR (400 MHz, d_6-DMSO) δ 10.99 (s, 1 H), 8.06 (d, J = 6.8 Hz, 2 H), 7.53–7.45 (m, 4 H), 7.33 (d, J = 8.0 Hz, 1 H), 7.09 (t, J = 7.2 Hz, 1 H), 7.03–6.99 (m, 1 H), 5.68 (s, 1 H), 5.43 (A of an ABq, J = 18.0 Hz, 1 H), 4.35 (B of an ABq, J = 18.0 Hz, 1 H), 3.79 (A of an ABq, J = 15.6 Hz, 1 H), 3.60 (d, J = 4.0 Hz, 2 H), 3.56 (s, 3 H), 3.00 (B of an ABq, J = 15.2 Hz, 1 H); ^{13}C NMR (100 MHz, d_6-DMSO) δ 171.1, 158.1, 149.9, 137.2, 136.8, 132.7, 131.1, 130.1, 129.2, 129.0, 128.6, 126.5, 121.6, 119.2, 118.2, 111.6, 110.0, 104.8, 71.2, 53.7, 39.9, 36.9, 32.8; HRMS (TOF-ES$^+$) (m/z): $[M + H]^+$ calcd for $C_{25}H_{21}N_2O_3$, 397.1552; found, 397.1587.

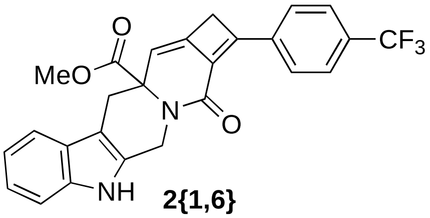

2{1,6}

(±)-Methyl 3-oxo-2-(4-(trifluoromethyl)phenyl)-1,3,5,6,11,11a-hexahydrocyclo-buta[*b*]indolo [2,3-*g*]quinolizine-11a-carboxylate (2{1,6}). Method B: To a solution of 3-(4-(trifluoromethyl)phenyl)propiolic acid (108 mg, 0.503 mmol), amino ester **6{1}** (90 mg, 0.335 mmol), and PyBroP (124 mg, 0.503 mmol) in CH_2Cl_2 (1.0 mL) was added DIEA (234 µL, 1.34 mmol) at 0 °C. The ice bath was removed after 1–2 min and the reaction mixture was stirred at rt for 1 h. The mixture was poured into EtOAc (15 mL) and washed with 5% $NaHSO_4$,

saturated $NaHCO_3$, and brine, then dried over $MgSO_4$, filtered, and concentrated in vacuo. The resulting residue was filtered through a short plug of silica (eluted with EtOAc:hexane, 1:1) to isolate a mixture of allene-yne **9{1,6}** and cyclobutene **2{1,6}** (105 mg combined). The mixture was resuspended in CH_2Cl_2 (4 mL) and placed in front of two 6 W (λ = 254 nm) lamps for 24 h without stirring at rt. The reaction was concentrated and purified using flash chromatography (SiO_2, CH_2Cl_2:acetone, 39:1) to afford the cyclobutene **2{1,6}** (64.4 mg, 41%) as a yellow solid. R_f 0.43 (CH_2Cl_2:acetone, 39:1); mp 284–286 °C (decomp); IR (neat): 3284, 1735, 1646, 1605, 1320, 1200, 1163, 1122, 1113, 1064, 829, 740 cm^{-1}; ^{1}H NMR (400 MHz, d_6-DMSO) δ 11.00 (s, 1 H), 8.23 (d, J = 8.0 Hz, 2 H), 7.87 (d, J = 8.0 Hz, 2 H), 7.46 (d, J = 8.0 Hz, 1 H), 7.33 (d, J = 8.0 Hz, 1 H), 7.11–7.07 (m, 1 H), 7.03–6.99 (m, 1 H), 5.80 (s, 1 H), 5.45 (A of an ABq, J = 18.0 Hz, 1 H), 4.36 (B of an ABq, J = 17.6 Hz, 1 H), 3.79 (A of an ABq, J = 15.2 Hz, 1 H), 3.67 (d, J = 2.4 Hz, 2 H), 3.57 (s, 3 H), 3.02 (B of an ABq, J = 15.6 Hz, 1 H); ^{13}C NMR (100 MHz, d_6-DMSO) δ 170.8, 157.8, 147.9, 140.0, 136.8, 136.2, 130.9, 130.0, 129.5, 126.4, 126.2, 126.1, 121.7, 119.2, 118.2, 111.8, 111.7, 104.8, 71.4, 53.8, 37.0, 32.7; HRMS (TOF-ES$^+$) (m/z): $[M + H]^+$ calcd for $C_{26}H_{20}N_2O_3F_3$, 465.1426; found, 465.1436.

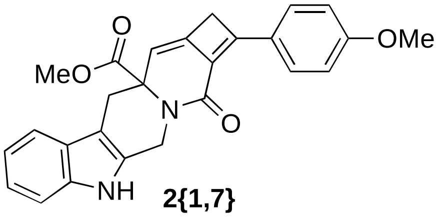

2{1,7}

(±)-Methyl 2-(4-methoxyphenyl)-3-oxo-1,3,5,6,11,11a-hexahydrocyclobuta[*b*]indolo[2,3-*g*]quinolizine-11a-carboxylate (2{1,7}). Method B: To a solution of 3-(4-methoxyphenyl)propiolic acid (89 mg, 0.503 mmol), amino ester **6{1}** (90 mg, 0.335 mmol), and PyBroP (124 mg, 0.503 mmol) in CH_2Cl_2 (1.0 mL) was added DIEA (234 µL, 1.34 mmol) at 0 °C. The ice bath was removed after 1–2 min and the reaction mixture was stirred at rt for 2 h. The mixture was poured into EtOAc (15 mL) and washed with 5% $NaHSO_4$, saturated $NaHCO_3$ and brine, then dried over $MgSO_4$, filtered, and concentrated in vacuo. The resulting residue was filtered through a short plug of silica (eluted with EtOAc:hexane, 1:1) to isolate a mixture of allene-yne **9{1,7}** and cyclobutene **2{1,7}** (69 mg combined). The mixture was resuspended in CH_2Cl_2 (4 mL) and placed in front of two 6 W (λ = 254 nm) lamps for 24 h without stirring at rt.

The reaction was concentrated and purified using flash chromatography (SiO_2, CH_2Cl_2:acetone, 39:1) to afford the cyclobutene **2{1,7}** (43.6 mg, 30%) as a white solid. R_f 0.20 (CH_2Cl_2:acetone, 39:1); mp 296–298 °C (decomp); IR (neat): 3256, 1733, 1646, 1599, 1508, 1331, 1305, 1252, 1170, 1025, 829, 738 cm^{-1}; ^1H NMR (400 MHz, d_6-DMSO) δ 10.98 (s, 1 H), 8.01 (d, J = 8.8 Hz, 2 H), 7.45 (d, J = 7.6 Hz, 1 H), 7.32 (d, J = 8.0 Hz, 1 H), 7.10–7.06 (m, 3 H), 6.99–6.98 (m, 1 H), 5.57 (s, 1 H), 5.41 (A of an ABq, J = 17.6 Hz, 1 H), 4.32 (B of an ABq, J = 18.0 Hz, 1 H), 3.85 (s, 3 H), 3.78 (A of an ABq, J = 15.2 Hz, 1 H), 3.57–3.52 (m, 5 H), 2.99 (B of an ABq, J = 15.2 Hz, 1 H); ^{13}C NMR (100 MHz, d_6-DMSO) δ 171.2, 161.7, 158.4, 150.0, 137.3, 136.8, 131.1, 130.2, 126.5, 126.0, 125.7, 121.6, 119.2, 118.2, 114.8, 111.6, 108.5, 104.9, 71.1, 55.9, 53.6, 36.8, 32.9; HRMS (TOF-ES$^+$) (m/z): $[M + H]^+$ calcd for $C_{26}H_{23}N_2O_4$, 427.1658; found, 427.1620.

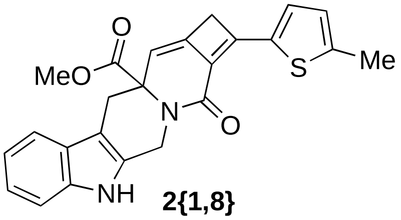

2{1,8}

(±)-Methyl 2-(5-methylthiophen-2-yl)-3-oxo-1,3,5,6,11,11a-hexahydrocyclobuta[*b*]indolo [2,3-*g*]quinolizine-11a-carboxylate (2{1,8}). Method B: To a solution of 3-(5-methylthiophen-2-yl)propiolic acid (83.6 mg, 0.503 mmol), amino ester **6{1}** (90 mg, 0.335 mmol), and PyBroP (124 mg, 0.503 mmol) in CH_2Cl_2 (1.0 mL) was added DIEA (234 uL, 1.34 mmol) while stirring at 0 °C. The ice bath was removed after 1–2 min and the reaction mixture was stirred at rt for 3 h. The mixture was poured into EtOAc (15 mL) and washed with 5% $NaHSO_4$, saturated $NaHCO_3$ and brine, then dried over $MgSO_4$, filtered, and concentrated in vacuo. The resulting residue was filtered through a short plug of silica (eluted with EtOAc:hexane, 1:1) to isolate a mixture of allene-yne **9{1,8}** and cyclobutene **2{1,8}** (62 mg combined). The semipurified product was placed in front of two 6 W (λ = 254 nm) lamps for 24 h without stirring at rt. The reaction was concentrated and purified using flash chromatography (SiO_2, CH_2Cl_2:acetone, 39:1) to afford the cyclobutene **2{1,8}** (45.6 mg, 0.109 mmol, 33%) as a yellow solid. R_f 0.24 (CH_2Cl_2:acetone, 39:1); mp 297–298 °C (decomp); ^1H NMR (400 MHz, d_6-DMSO) δ 10.97 (s, 1 H), 7.48–7.44 (m, 2 H), 7.31 (d, J = 8.0 Hz, 1 H), 7.06 (t, J = 7.2 Hz, 1 H), 7.02–6.94 (m, 1 H), 6.93 (d, J =

2.8 Hz, 1 H), 5.57 (s, 1 H), 5.38 (A of an ABq, J = 17.6 Hz, 1 H), 4.28 (B of an ABq, J = 18.0 Hz, 1 H), 3.74 (A of an ABq, J = 15.5 Hz, 1 H), 3.58–3.56 (m, 5 H), 2.98 (B of an ABq, J = 15.6 Hz, 1 H), 2.52 (s, 3 H); ^{13}C NMR (100 MHz, d_6-DMSO) δ 171.1, 157.9, 147.2, 141.3, 137.3, 136.8, 133.9, 132.1, 130.2, 127.7, 126.5, 124.2, 121.6, 119.2, 118.2, 111.6, 109.1, 104.8, 71.3, 53.7, 38.0, 32.9, 15.9; IR (neat): 3247, 1731, 1642, 1599, 1331, 1264, 1195, 1159, 1040, 734 cm^{-1}; HRMS (TOF-ES$^+$) (m/z): [M + H]$^+$ calcd for $C_{24}H_{21}N_2O_3S$, 417.1271; found, 417.1273.

(±)-Methyl 3-methyl-1-methylene-2,4-dioxo-1,2,4,6,7,12,12a,12b-octahydrocyclo-penta[1,2]indolizino[6,7-*b*]indole-12a-carboxylate (3{1,3}). To an oven dried 1 dram tall sealable vial was added $Mo(CO)_6$ (83 mg, 0.31 mmol). The vial was evacuated under vacuum and filled with argon (3 x). A solution of allene-amide **9{1,3}** (70 mg, 0.21 mmol) in dry toluene (4 mL) was added by syringe followed by the addition of dry DMSO (125 µL, 1.77 mmol). The solution was placed in a preheated oil bath at 95 °C for 2 h. After completion the solution was passed through a pipette containing silica. The filtrate was concentrated and the crude material was purified by flash chromatography (SiO_2, hexane:EtOAc, 2:1) to afford **3{1,3}** (36.8 mg, 0.101 mmol, 48%) as a 1:1 mixture of two diastereomers as an amorphous residue. R_f 0.11 (hexane:EtOAc, 2:1); ^{1}H NMR (400 MHz, $CDCl_3$) δ 8.65 (s, 0.5 H), 8.52 (s, 0.5 H), 7.55 (d, J = 7.5 Hz, 0.5 H), 7.42 (d, J = 7.6 Hz, 0.5 H), 7.33 (d, J = 6.0 Hz, 0.5 H), 7.31 (d, J = 8.0 Hz, 0.5 H), 7.29–7.16 (m, 1.5 H), 7.13–7.11 (m, 0.5 H), 6.49 (d, J = 2.0 Hz, 0.5 H), 6.36 (d, J = 2.0 Hz, 0.5 H), 5.97 (d, J = 1.0 Hz, 0.5 H), 5.76 (d, J = 1.2 Hz, 0.5 H), 5.32 (dd, J = 16.6, 1.3 Hz, 0.5 H), 5.01 (dd, J = 17.2, 1.4 Hz, 0.5 H), 4.66 (d, J = 16.6 Hz, 0.5 H), 4.38 (d, J = 17.3 Hz, 0.5 H), 4.16 (d, J = 7.2 Hz, 0.5 H), 4.00–3.98 (m, 1 H), 3.78 (s, 1.5 H), 3.44 (s, 1.5 H), 3.32 (d, J = 15.6 Hz, 0.5 H), 3.09 (d, J = 15.1 Hz, 0.5 H), 2.37 (d, J = 15.6 Hz, 0.5 H), 2.19 (d, J = 3.2 Hz, 1.5 H), 2.15 (d, J = 2.8 Hz, 1.5 H); ^{13}C NMR (100 MHz, $CDCl_3$) δ 196.0, 195.5, 171.7, 168.9, 165.6, 164.1, 154.9, 153.9, 143.0, 141.8, 140.4, 139.8, 136.7, 136.4, 128.9, 127.6, 126.5, 126.1, 122.3, 122.2, 121.1, 120.9, 119.8, 119.7, 118.0, 117.9, 111.1, 111.0, 105.9, 105.1, 69.2, 66.7, 60.4, 53.3, 53.0, 52.1, 48.3, 38.8, 38.0, 30.8, 25.0, 21.0, 14.1, 9.1, 9.0; IR (neat): 3332,

1733, 1698, 1387, 1370, 1331, 1232, 1198, 1042, 744 cm^{-1}; HRMS (TOF-ES^+) (*m*/*z*): $[M + H]^+$ calcd for $C_{21}H_{19}N_2O_4$, 363.1345; found, 363.1341.

(±)-Methyl 3-cyclopropyl-1-methylene-2,4-dioxo-1,2,4,6,7,12,12a,12b-octahydrocyclo-penta[1,2]indolizino[6,7-*b*]indole-12a-carboxylate (3{1,4}). To an oven-dried tall sealable vial was added $Mo(CO)_6$ (142 mg, 0.54 mmol). The vial was evacuated under vacuum and filled with Argon (3 x). A solution of allene-amide **9{1,4}** (130 mg, 0.361 mmol) in dry toluene (4 mL) was added by syringe followed by the addition of dry DMSO (218 µL, 3.1 mmol). The solution was placed in a preheated oil bath at 95 °C for 2 h. After completion the solution was passed through a pipette containing silica. The filtrate was concentrated and the crude material was purified by flash chromatography (SiO_2, hexane:EtOAc, 2:1) to afford **3{1,4}** (63 mg, 0.162 mmol, 46%) as a 1:1 mixture of two diastereomers as an amorphous residue. R_f 0.52 (hexane:EtOAc, 1:1); ^{1}H NMR (400 MHz, $CDCl_3$) δ 8.89 (s, 0.5 H), 8.66 (s, 0.5 H), 7.55 (d, *J* = 7.5 Hz, 0.5 H), 7.50 (d, *J* = 7.8 Hz, 0.5 H), 7.42 (d, *J* = 8.0 Hz, 0.5 H), 7.35–7.29 (m, 1 H), 7.22–7.11 (m, 1.5 H), 6.42 (d, *J* = 2.0 Hz, 0.5 H), 6.30 (d, *J* = 2.0 Hz, 0.5 H), 5.94 (d, *J* = 1.0 Hz, 0.5 H), 5.73 (d, *J* = 1.6 Hz, 0.5 H), 5.31 (dd, *J* = 13.2, 1.3 Hz, 0.5 H), 5.01 (dd, *J* = 13.3, 1.4 Hz, 0.5 H), 4.66 (d, *J* = 16.6 Hz, 0.5 H), 4.40 (d, *J* = 17.3 Hz, 0.5 H), 4.10 (d, *J* = 14.8 Hz, 0.5 H), 3.94 (d, *J* = 7.2 Hz, 1 H), 3.77 (s, 1.5 H), 3.46 (s, 1.5 H), 3.29 (d, *J* = 15.3 Hz, 0.5 H), 3.07 (d, *J* = 14.8 Hz, 0.5 H), 2.24 (d, *J* = 8.8 Hz, 0.5 H), 1.62–1.60 (m, 1 H), 1.53–1.39 (m, 1 H), 1.15–1.08 (m, 1 H), 1.06–1.02 (m, 1 H), 1.01–0.99 (m, 1 H); ^{13}C NMR (100 MHz, $CDCl_3$) δ 194.6, 194.4, 171.8, 169.0, 164.3, 151.3, 150.6, 148.7, 147.9, 140.4, 140.0, 136.9, 136.8, 136.4, 129.1, 127.8, 126.5, 126.1, 122.2, 122.1, 120.7, 119.7, 119.6, 118.0, 117.8, 111.1, 111.0, 105.7, 105.5, 105.1, 69.0, 66.7, 53.2, 52.9, 52.1, 48.1, 39.1, 38.1, 30.7, 25.0, 9.5, 9.2, 8.9, 8.5, 8.0, 7.7; IR (neat): 3353, 1735, 1694, 1661, 1353, 1331, 1200, 973, 952, 742 cm^{-1}; HRMS (TOF-ES^+) (*m*/*z*): $[M + H]^+$ calcd for $C_{23}H_{21}N_2O_4$, 389.1501; found, 389.1518.

References

[S1] Painter, T. O.; Wang, L.; Majumder, S.; Xie, X.-Q.; Brummond, K. M. *ACS Comb. Sci.* **2011**, *13* 166–174.

[S2] Ran, J.-Q.; Huang, N.; Xu, H.; Yang, L.-M.; Lv, M.; Zheng, Y.-T. *Bioorg. Med. Chem. Lett.* **2010**, *20*, 3534–3536.

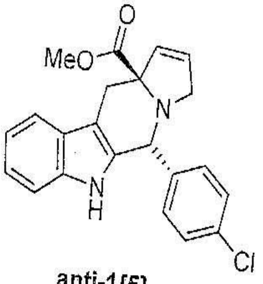

anti-1{5}

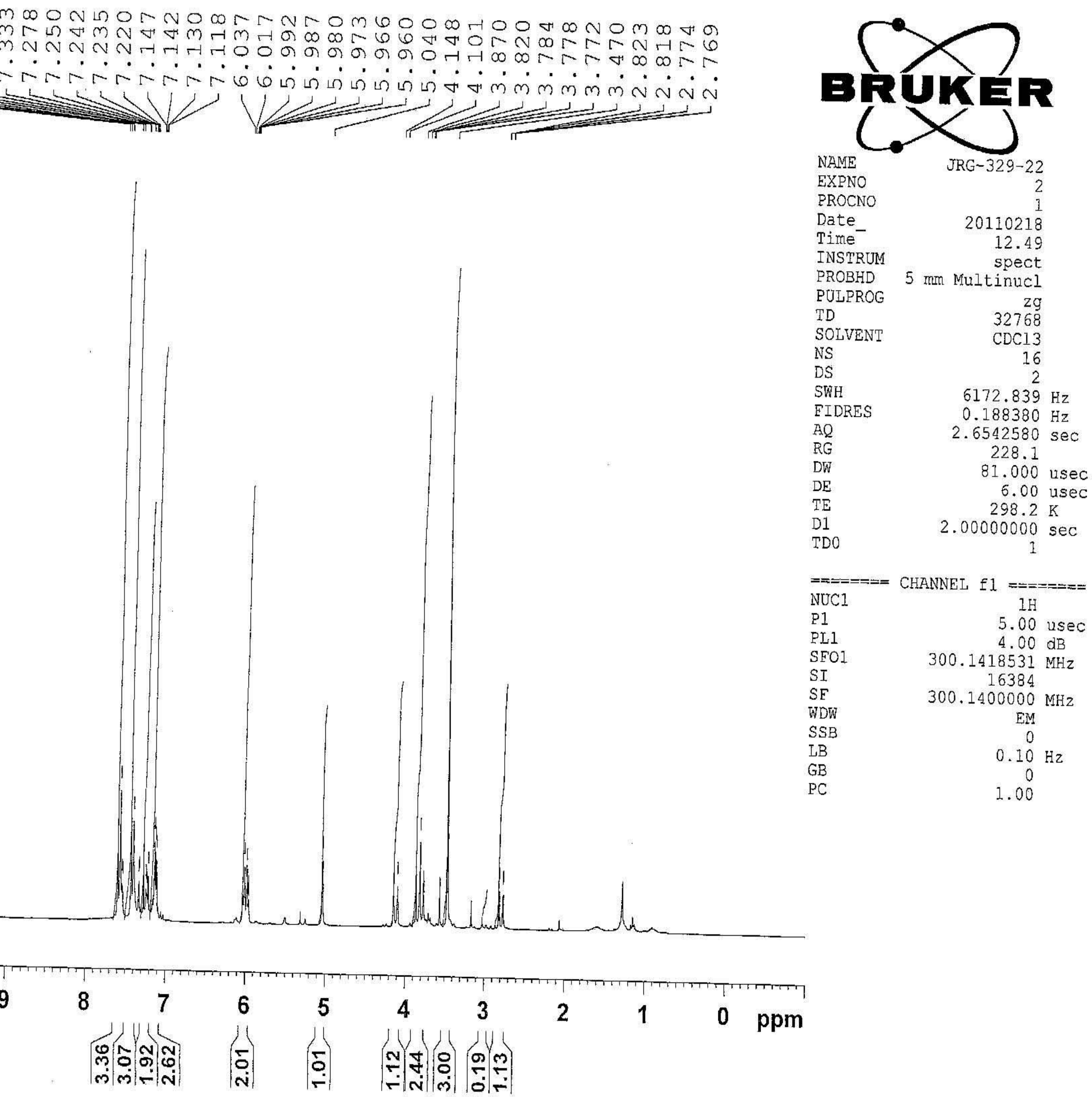

7.601 7.572 7.553 7.540 7.435 7.407 7.339 7.333 7.278 7.250 7.242 7.235 7.220 7.147 7.142 7.130 7.118 6.037 6.017 5.992 5.987 5.980 5.973 5.966 5.960 5.040 4.148 4.101 3.870 3.820 3.784 3.778 3.772 3.470 2.823 2.818 2.774 2.769
BRUKER
NAME JRG-329-22
EXPNO 2
PROCNO 1
Date_ 20110218
Time_ 12.49
INSTRUM spect
PROBHD 5 mm Multinucl
PULPROG zg
TD 32768
SOLVENT CDCl3
NS 16
DS 2
SWH 6172.839 Hz
FIDRES 0.188380 Hz
AQ 2.6542580 sec
RG 228.1
DW 81.000 usec
DE 6.00 usec
TE 298.2 K
D1 2.00000000 sec
TD0 1
======== CHANNEL f1 ========
NUC1 1H
P1 5.00 usec
PL1 4.00 dB
SFO1 300.1418531 MHz
SI 16384
SF 300.1400000 MHz
WDW EM
SSB 0
LB 0.10 Hz
GB 0
PC 1.00
11 10 9 8 7 6 5 4 3 2 1 0 ppm
3.36 3.07 1.92 2.62 2.01 1.01 1.12 2.44 3.00 0.19 1.13

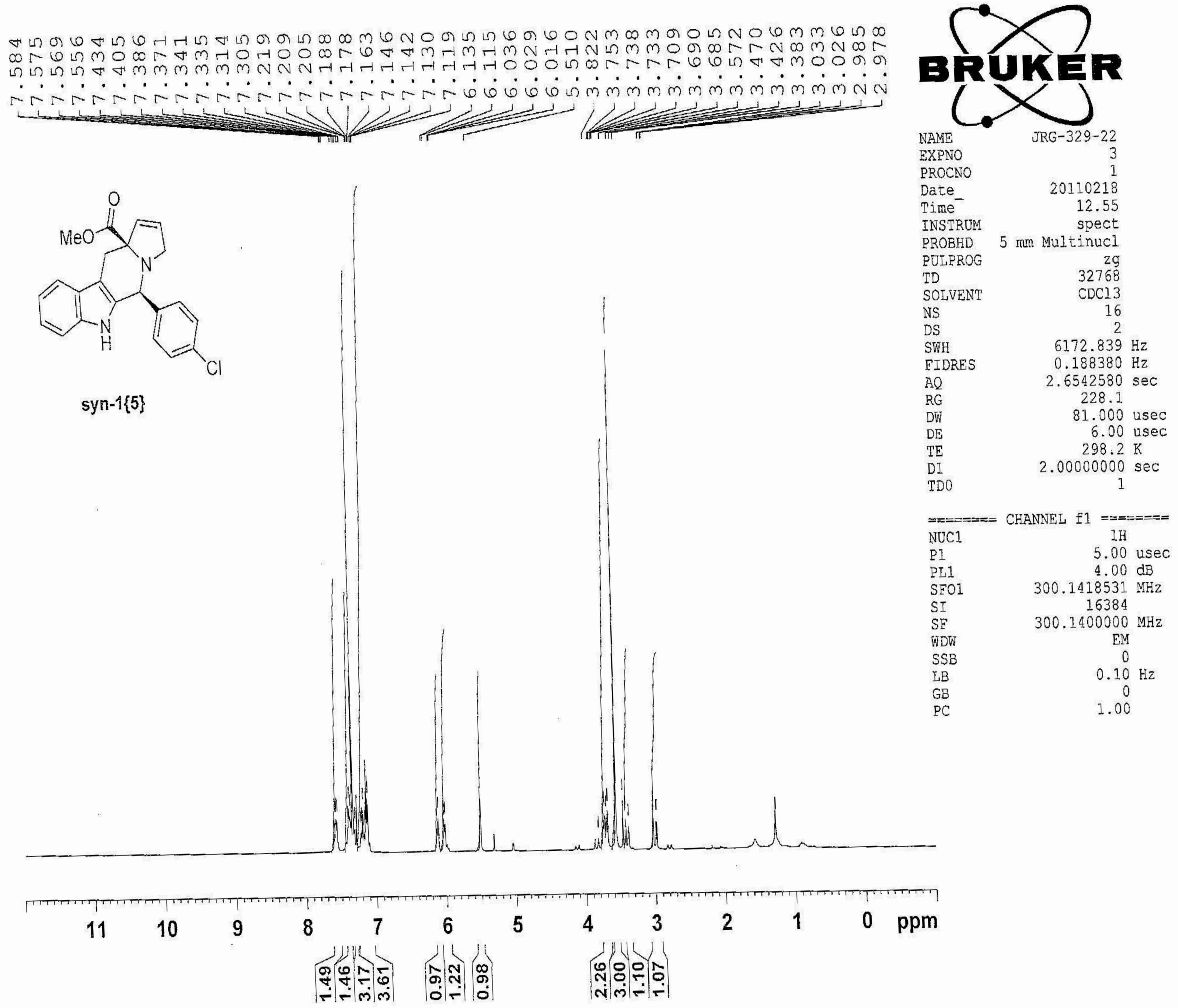
7.584
7.575
7.569
7.556
7.434
7.405
7.386
7.371
7.341
7.335
7.314
7.305
7.219
7.209
7.205
7.188
7.178
7.163
7.146
7.142
7.130
7.119
6.135
6.115
6.036
6.029
6.016
5.510
3.822
3.753
3.738
3.733
3.709
3.690
3.685
3.572
3.470
3.426
3.383
3.033
3.026
2.985
2.978
BRUKER
NAME JRG-329-22
EXPNO 3
PROCNO 1
Date_ 20110218
Time_ 12.55
INSTRUM spect
PROBHD 5 mm Multinucl
PULPROG zg
TD 32768
SOLVENT CDCl3
NS 16
DS 2
SWH 6172.839 Hz
FIDRES 0.188380 Hz
AQ 2.6542580 sec
RG 228.1
DW 81.000 usec
DE 6.00 usec
TE 298.2 K
D1 2.00000000 sec
TD0 1
======== CHANNEL f1 ========
NUC1 1H
P1 5.00 usec
PL1 4.00 dB
SFO1 300.1418531 MHz
SI 16384
SF 300.1400000 MHz
WDW EM
SSB 0
LB 0.10 Hz
GB 0
PC 1.00
O
MeO
N
N
H
Cl
syn-1{5}
11
10
9
8
7
6
5
4
3
2
1
0
ppm
1.49
1.46
3.17
3.61
0.97
1.22
0.98
2.26
3.00
1.10
1.07

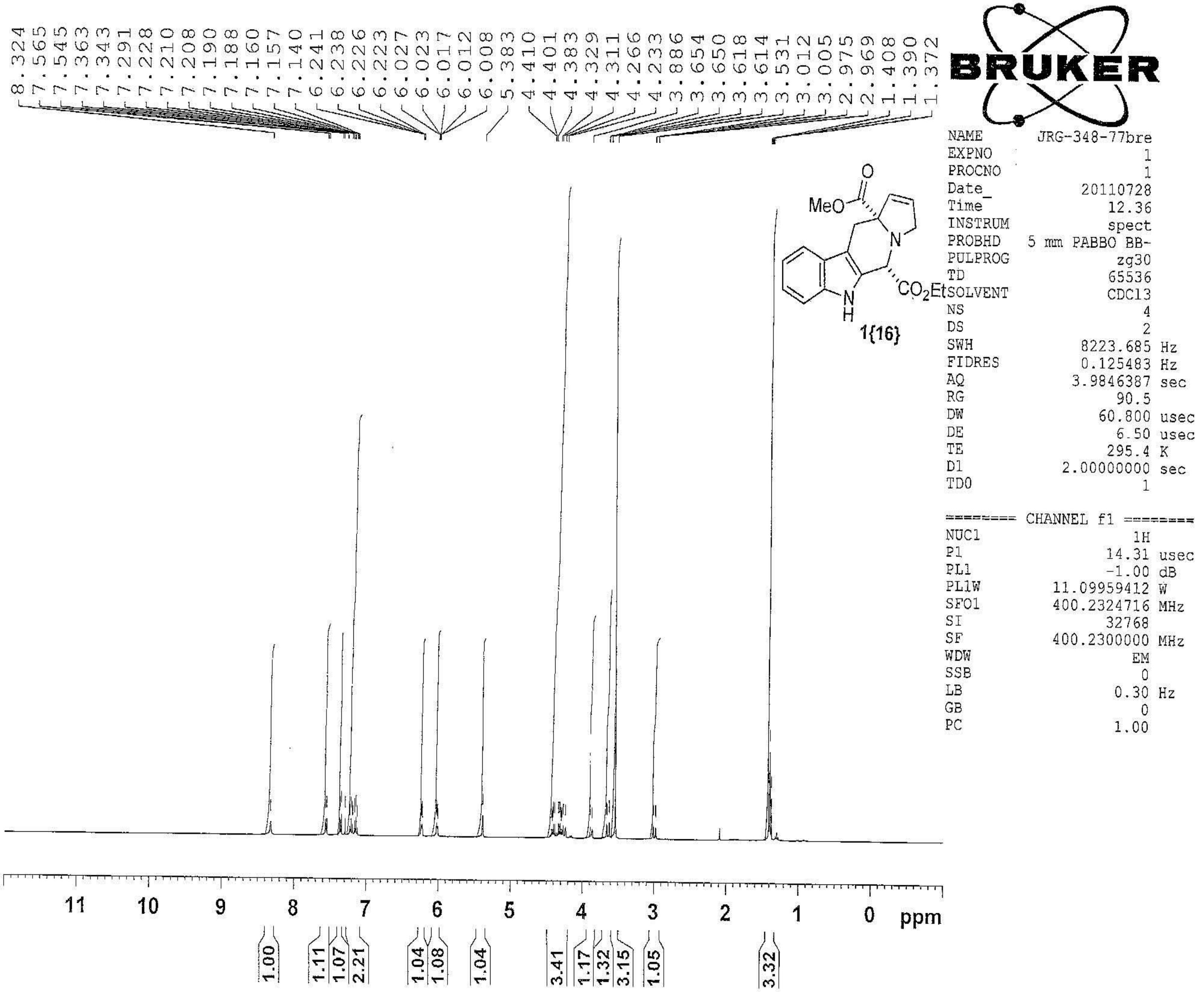
8.324 7.565 7.545 7.363 7.343 7.291 7.228 7.210 7.208 7.190 7.188 7.160 7.157 7.140 6.241 6.238 6.226 6.223 6.027 6.023 6.017 6.012 6.008 5.383 4.410 4.401 4.383 4.329 4.311 4.266 4.233 3.886 3.654 3.650 3.618 3.614 3.531 3.012 3.005 2.975 2.969 1.408 1.390 1.372
BRUKER
MeO
O
N
CO2Et
N
H
1{16}
NAME JRG-348-77bre
EXPNO 1
PROCNO 1
Date_ 20110728
Time 12.36
INSTRUM spect
PROBHD 5 mm PABBO BB-
PULPROG zg30
TD 65536
SOLVENT CDCl3
NS 4
DS 2
SWH 8223.685 Hz
FIDRES 0.125483 Hz
AQ 3.9846387 sec
RG 90.5
DW 60.800 usec
DE 6.50 usec
TE 295.4 K
D1 2.00000000 sec
TD0 1
======== CHANNEL f1 ========
NUC1 1H
P1 14.31 usec
PL1 -1.00 dB
PL1W 11.09959412 W
SFO1 400.2324716 MHz
SI 32768
SF 400.2300000 MHz
WDW EM
SSB 0
LB 0.30 Hz
GB 0
PC 1.00
11 10 9 8 7 6 5 4 3 2 1 0 ppm
1.00 1.11 1.07 2.21 1.04 1.08 1.04 3.41 1.17 1.32 3.15 1.05 3.32

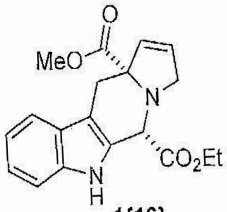

1{16}

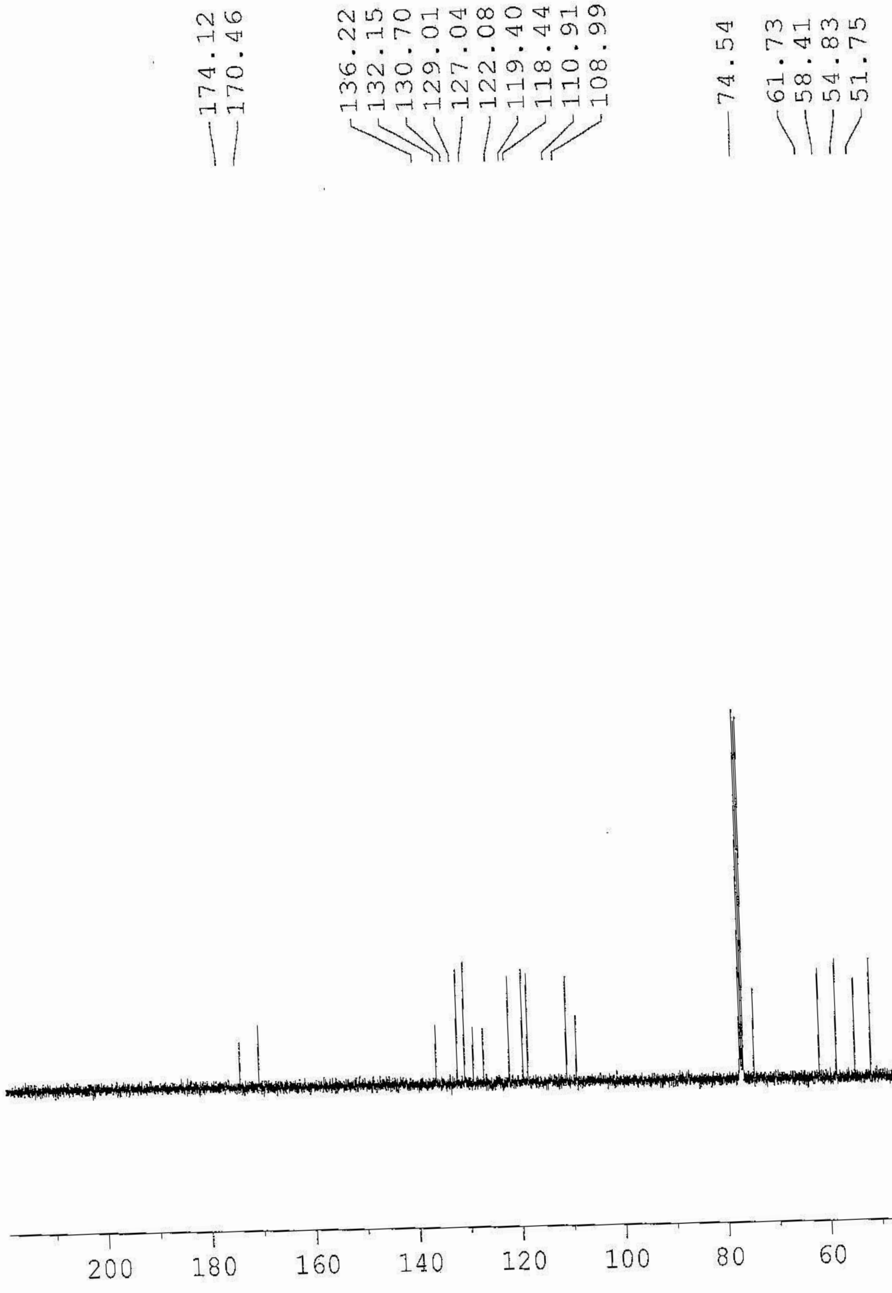

NAME	JRG-348-77bre
EXPNO	2
PROCNO	1
Date_	20110728
Time	12.46
INSTRUM	spect
PROBHD	5 mm PABBO BB-
PULPROG	zgpg30
TD	65536
SOLVENT	CDCl3
NS	75
DS	4
SWH	24038.461 Hz
FIDRES	0.366798 Hz
AQ	1.3631988 sec
RG	203
DW	20.800 usec
DE	6.50 usec
TE	295.8 K
D1	3.00000000 sec
D11	0.03000000 sec
TD0	1

======== CHANNEL f1 ========

NUC1	13C
P1	10.00 usec
PL1	-1.59 dB
PL1W	51.07626343 W
SFO1	100.6479773 MHz

======== CHANNEL f2 ========

CPDPRG2	waltz16
NUC2	1H
PCPD2	75.00 usec
PL2	-1.00 dB
PL12	13.39 dB
PL13	20.00 dB
PL2W	11.09959412 W
PL12W	0.40393090 W
PL13W	0.08816721 W
SFO2	400.2316009 MHz
SI	32768
SF	100.6379214 MHz
WDW	EM
SSB	0
LB	1.00 Hz
GB	0
PC	1.40

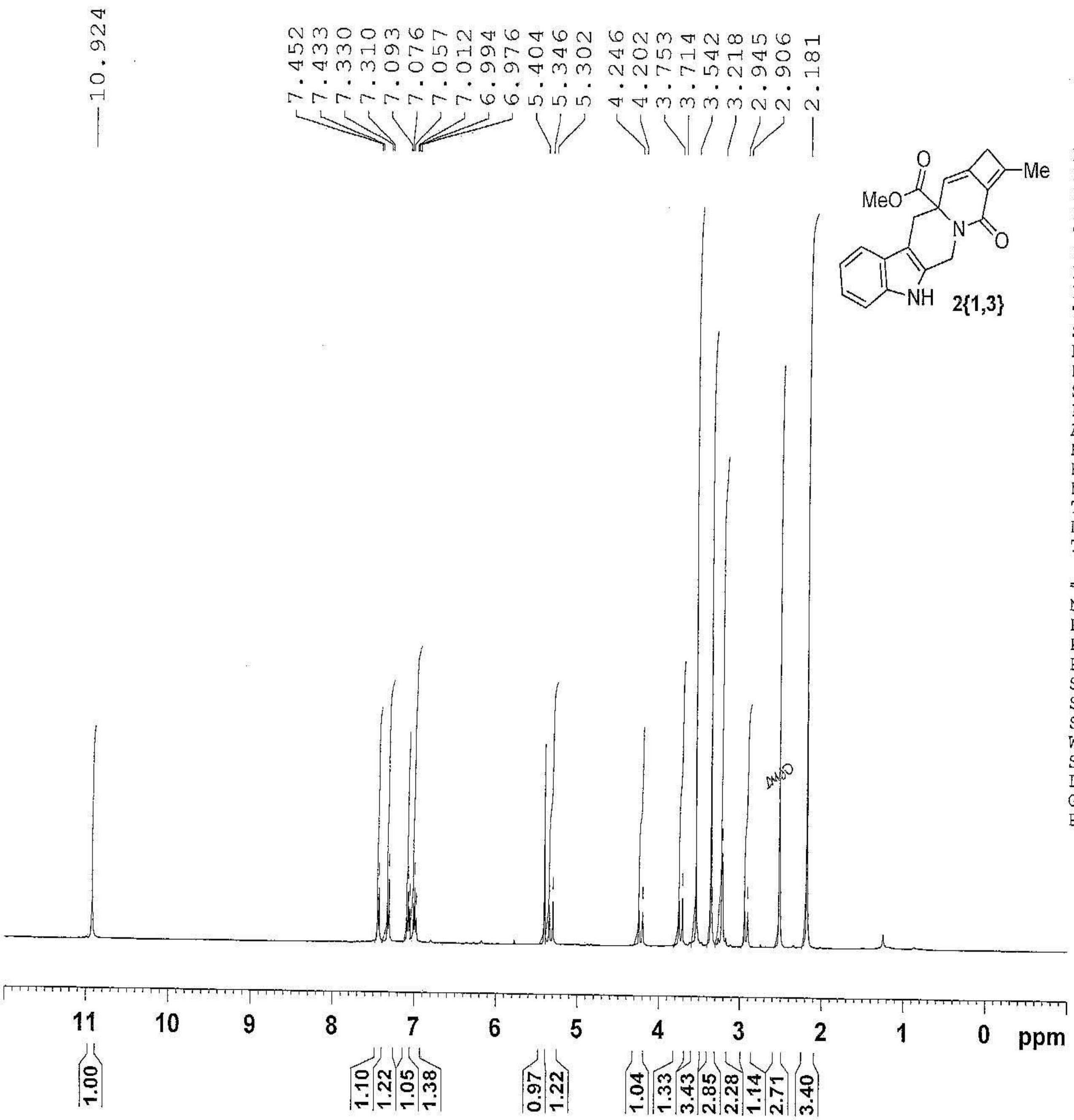

2{1,3}

NAME	JRG-348-35
EXPNO	1
PROCNO	1
Date_	20110629
Time	9.18
INSTRUM	spect
PROBHD	5 mm PABBO BB-
PULPROG	zg30
TD	65536
SOLVENT	DMSO
NS	4
DS	2
SWH	8223.685 Hz
FIDRES	0.125483 Hz
AQ	3.9846387 sec
RG	144
DW	60.800 usec
DE	6.50 usec
TE	294.9 K
D1	2.00000000 sec
TD0	1

======== CHANNEL f1 ========

NUC1	1H
P1	14.31 usec
PL1	-1.00 dB
PL1W	11.09959412 W
SFO1	400.2324716 MHz
SI	32768
SF	400.2300000 MHz
WDW	EM
SSB	0
LB	0.30 Hz
GB	0
PC	1.00

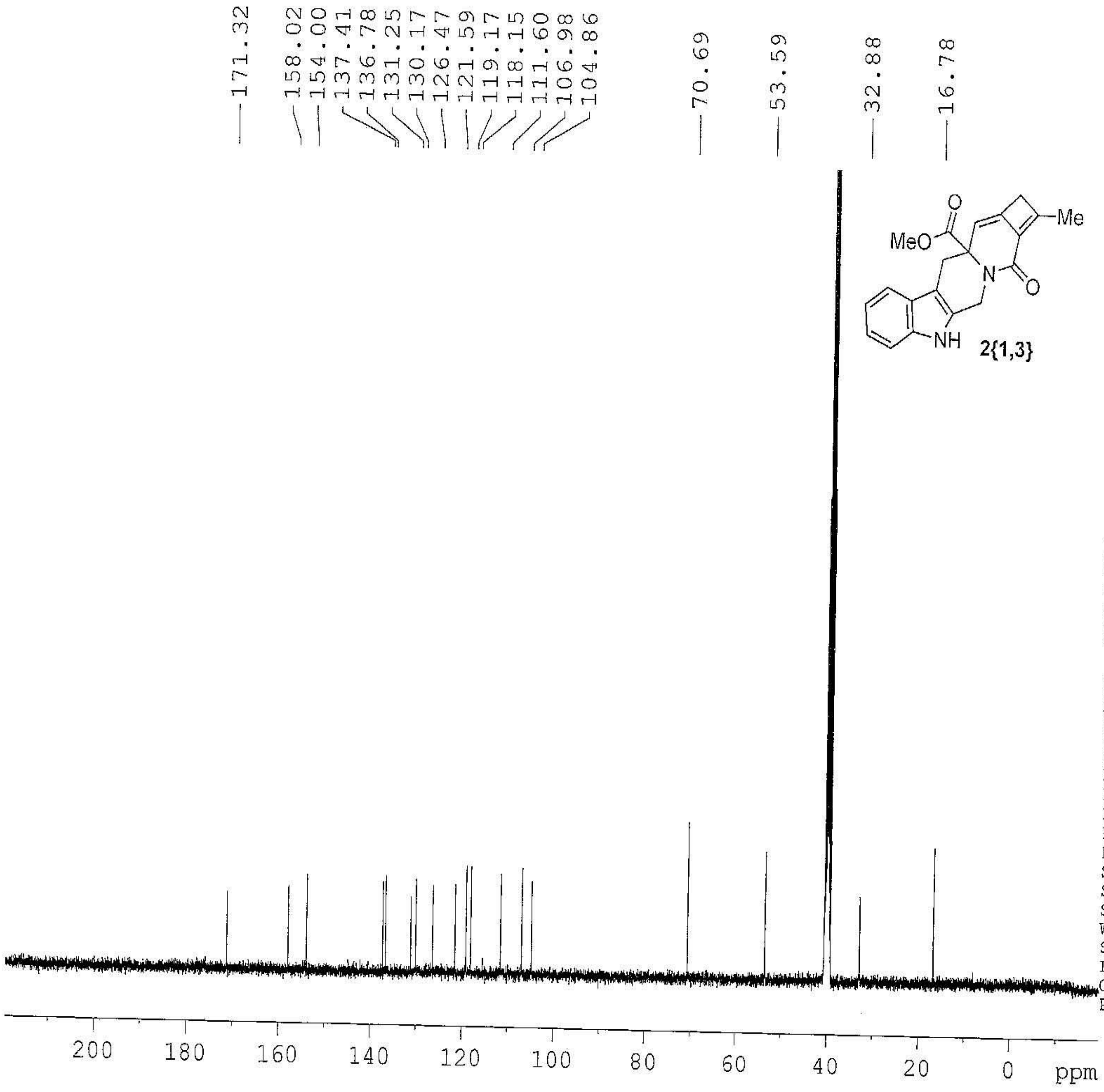

2{1,3}

BRUKER

NAME	JRG-348-35
EXPNO	2
PROCNO	1
Date_	20110629
Time_	9.22
INSTRUM	spect
PROBHD	5 mm PABBO BB-
PULPROG	zgpg30
TD	65536
SOLVENT	DMSO
NS	1200
DS	4
SWH	24038.461 Hz
FIDRES	0.366798 Hz
AQ	1.3631988 sec
RG	203
DW	20.800 usec
DE	6.50 usec
TE	295.2 K
D1	3.00000000 sec
D11	0.03000000 sec
TD0	1

======== CHANNEL f1 ========

NUC1	13C
P1	10.00 usec
PL1	-1.59 dB
PL1W	51.07626343 W
SFO1	100.6479773 MHz

======== CHANNEL f2 ========

CPDPRG2	waltz16
NUC2	1H
PCPD2	75.00 usec
PL2	-1.00 dB
PL12	13.39 dB
PL13	20.00 dB
PL2W	11.09959412 W
PL12W	0.40393090 W
PL13W	0.08816721 W
SFO2	400.2316009 MHz
SI	32768
SF	100.6379140 MHz
WDW	EM
SSB	0
LB	1.00 Hz
GB	0
PC	1.40

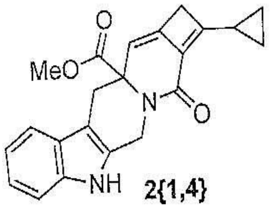

2{1,4}

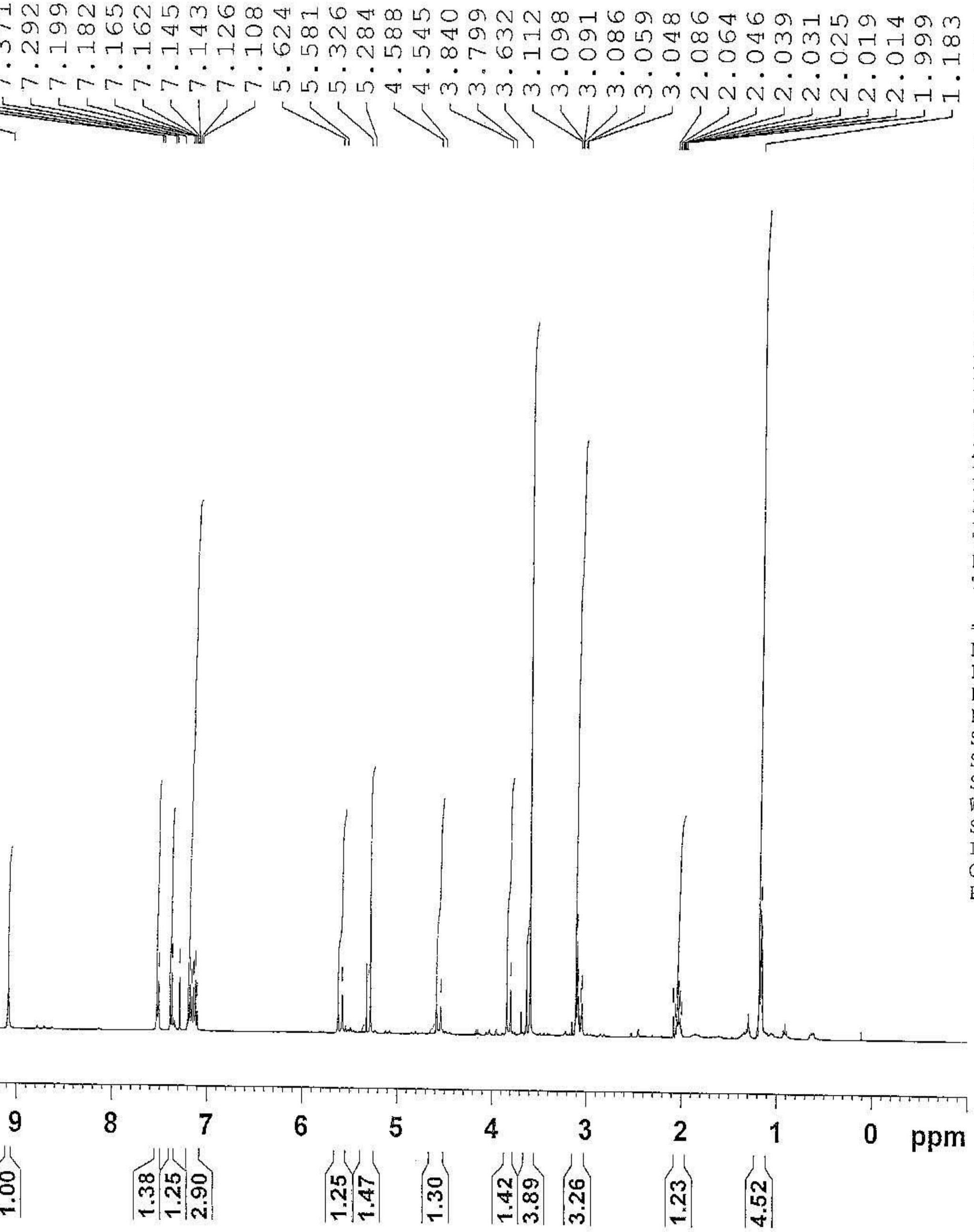

BRUKER

NAME	JRG-348-70re
EXPNO	1
PROCNO	1
Date_	20110726
Time	23.18
INSTRUM	spect
PROBHD	5 mm PABBO BB-
PULPROG	zg30
TD	65536
SOLVENT	CDCl3
NS	6
DS	2
SWH	8223.685 Hz
FIDRES	0.125483 Hz
AQ	3.9846387 sec
RG	101
DW	60.800 usec
DE	6.50 usec
TE	295.2 K
D1	2.00000000 sec
TD0	1

======== CHANNEL f1 ========

NUC1	1H
P1	14.31 usec
PL1	-1.00 dB
PL1W	11.09959412 W
SFO1	400.2324716 MHz
SI	32768
SF	400.2300000 MHz
WDW	EM
SSB	0
LB	0.30 Hz
GB	0
PC	1.00

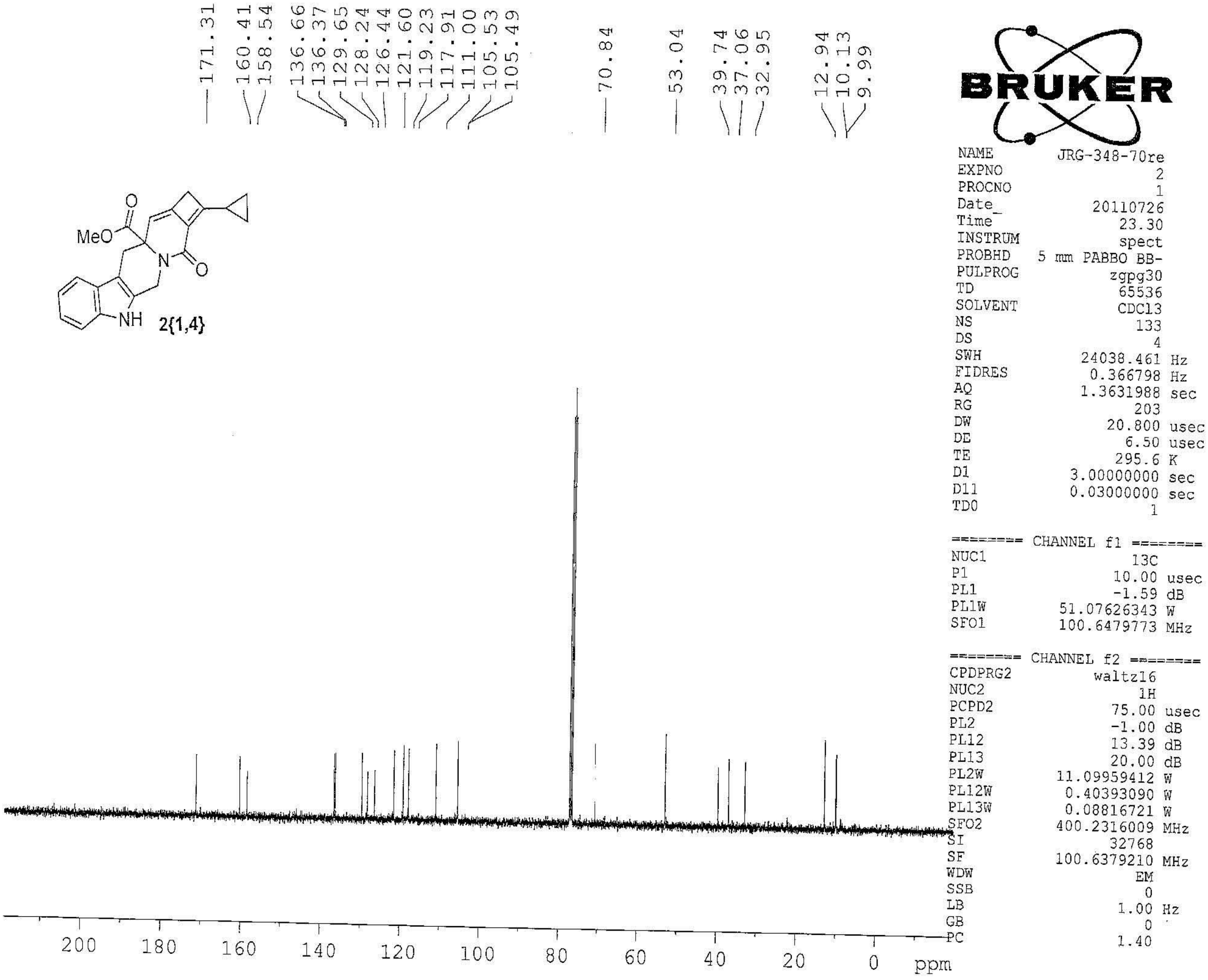
171.31
160.41
158.54
136.66
136.37
129.65
128.24
126.44
121.60
119.23
117.91
111.00
105.53
105.49
70.84
53.04
39.74
37.06
32.95
12.94
10.13
9.99
MeO
O
N
NH
2{1,4}
BRUKER
NAME JRG-348-70re
EXPNO 2
PROCNO 1
Date_ 20110726
Time 23.30
INSTRUM spect
PROBHD 5 mm PABBO BB-
PULPROG zgpg30
TD 65536
SOLVENT CDCl3
NS 133
DS 4
SWH 24038.461 Hz
FIDRES 0.366798 Hz
AQ 1.3631988 sec
RG 203
DW 20.800 usec
DE 6.50 usec
TE 295.6 K
D1 3.00000000 sec
D11 0.03000000 sec
TD0 1
======== CHANNEL f1 ========
NUC1 13C
P1 10.00 usec
PL1 -1.59 dB
PL1W 51.07626343 W
SFO1 100.6479773 MHz
======== CHANNEL f2 ========
CPDPRG2 waltz16
NUC2 1H
PCPD2 75.00 usec
PL2 -1.00 dB
PL12 13.39 dB
PL13 20.00 dB
PL2W 11.09959412 W
PL12W 0.40393090 W
PL13W 0.08816721 W
SFO2 400.2316009 MHz
SI 32768
SF 100.6379210 MHz
WDW EM
SSB 0
LB 1.00 Hz
GB 0
PC 1.40
200
180
160
140
120
100
80
60
40
20
0
ppm

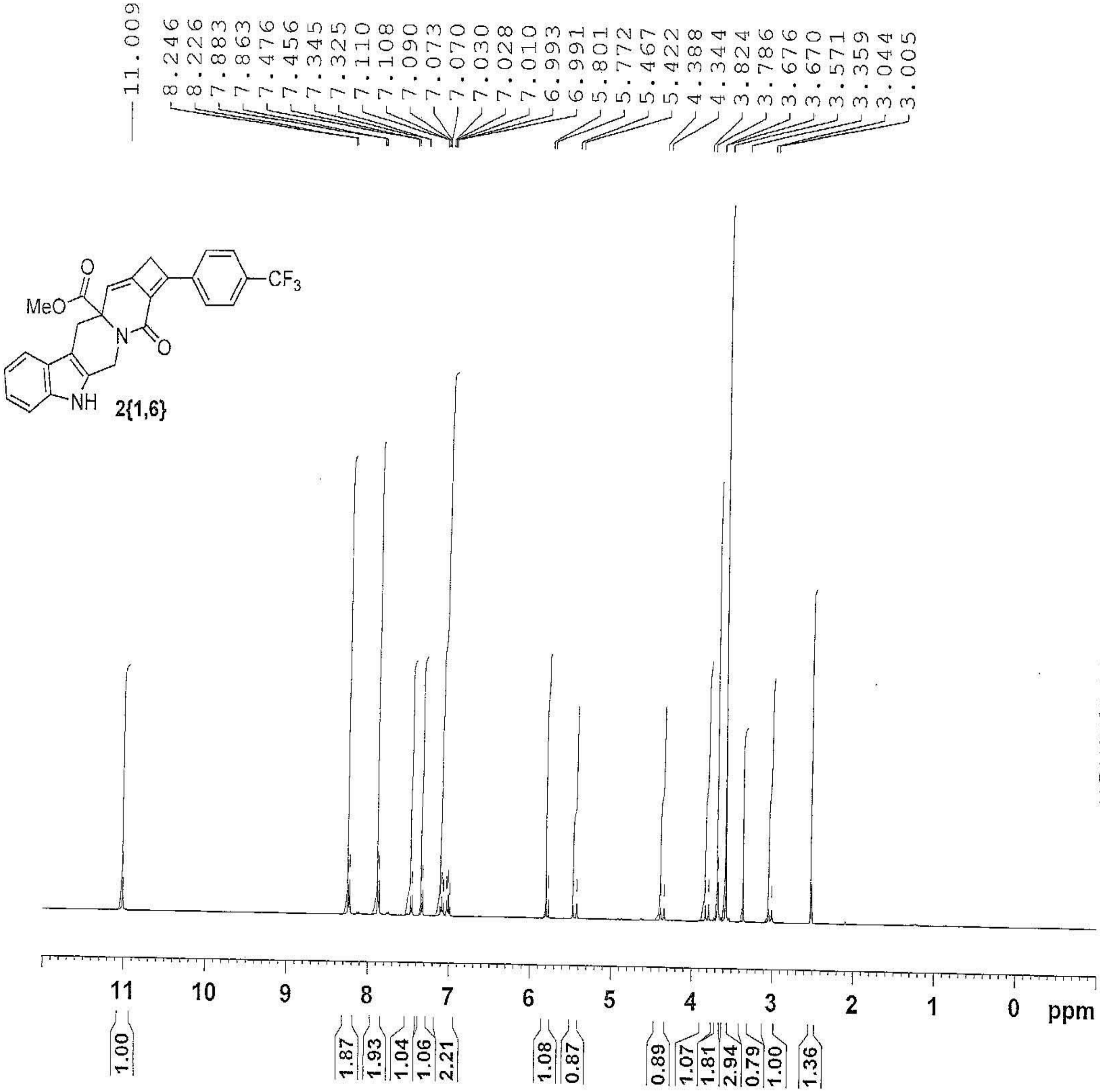

BRUKER

NAME	JRG-348-30_DMSO
EXPNO	1
PROCNO	1
Date_	20110628
Time	9.39
INSTRUM	spect
PROBHD	5 mm PABBO BB-
PULPROG	zg30
TD	65536
SOLVENT	DMSO
NS	3
DS	2
SWH	8223.685 Hz
FIDRES	0.125483 Hz
AQ	3.9846387 sec
RG	128
DW	60.800 usec
DE	6.50 usec
TE	295.3 K
D1	2.00000000 sec
TD0	1

======== CHANNEL f1 ========

NUC1	1H
P1	14.31 usec
PL1	-1.00 dB
PL1W	11.09959412 W
SFO1	400.2324716 MHz
SI	32768
SF	400.2300000 MHz
WDW	EM
SSB	0
LB	0.30 Hz
GB	0
PC	1.00

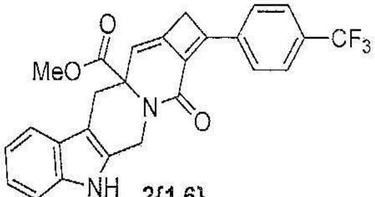

2{1,6}

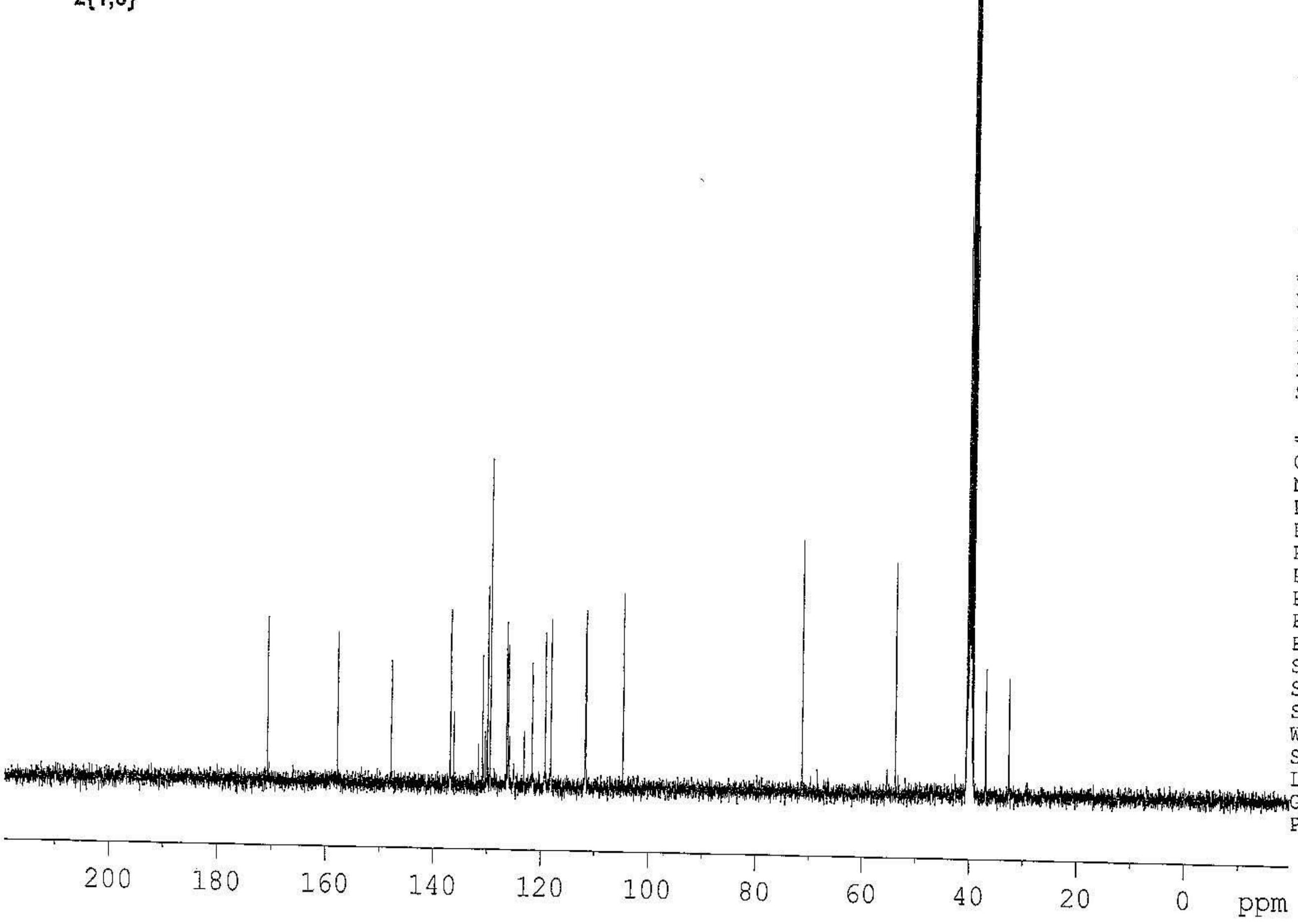

BRUKER

```
NAME       JRG-348-30_DMSO
EXPNO                    3
PROCNO                   1
Date_             20110628
Time                 13.00
INSTRUM              spect
PROBHD    5 mm PABBO BB-
PULPROG            zgpg30
TD                  65536
SOLVENT              DMSO
NS                    700
DS                      4
SWH             24038.461 Hz
FIDRES           0.366798 Hz
AQ              1.3631988 sec
RG                    203
DW                 20.800 usec
DE                   6.50 usec
TE                  295.8 K
D1             3.00000000 sec
D11            0.03000000 sec
TD0                     1

======== CHANNEL f1 ========
NUC1                  13C
P1                  10.00 usec
PL1                 -1.59 dB
PL1W          51.07626343 W
SFO1          100.6479773 MHz

======== CHANNEL f2 ========
CPDPRG2           waltz16
NUC2                   1H
PCPD2               75.00 usec
PL2                 -1.00 dB
PL12                13.39 dB
PL13                20.00 dB
PL2W          11.09959412 W
PL12W          0.40393090 W
PL13W          0.08816721 W
SFO2          400.2316009 MHz
SI                  32768
SF            100.6379140 MHz
WDW                    EM
SSB                     0
LB                   1.00 Hz
GB                      0
PC                   1.40
```

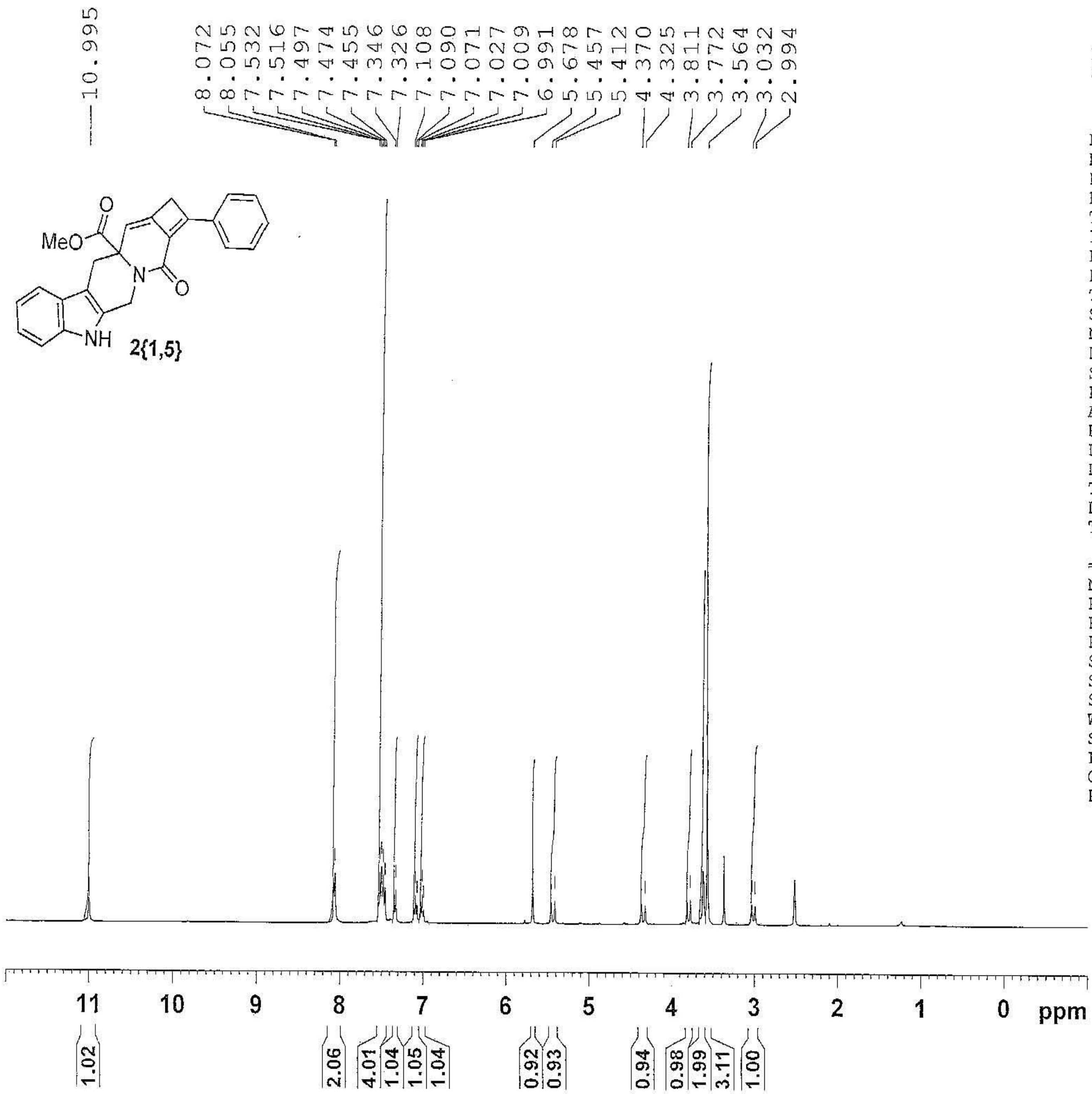

2{1,5}

BRUKER

NAME	JRG-348-27_DMSO
EXPNO	1
PROCNO	1
Date_	20110627
Time	15.18
INSTRUM	spect
PROBHD	5 mm PABBO BB-
PULPROG	zg30
TD	65536
SOLVENT	DMSO
NS	4
DS	2
SWH	8223.685 Hz
FIDRES	0.125483 Hz
AQ	3.9846387 sec
RG	101
DW	60.800 usec
DE	6.50 usec
TE	294.8 K
D1	2.00000000 sec
TD0	1

======== CHANNEL f1 ========

NUC1	1H
P1	14.31 usec
PL1	-1.00 dB
PL1W	11.09959412 W
SFO1	400.2324716 MHz
SI	32768
SF	400.2300000 MHz
WDW	EM
SSB	0
LB	0.30 Hz
GB	0
PC	1.00

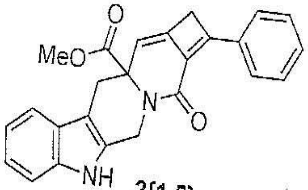
2{1,5}

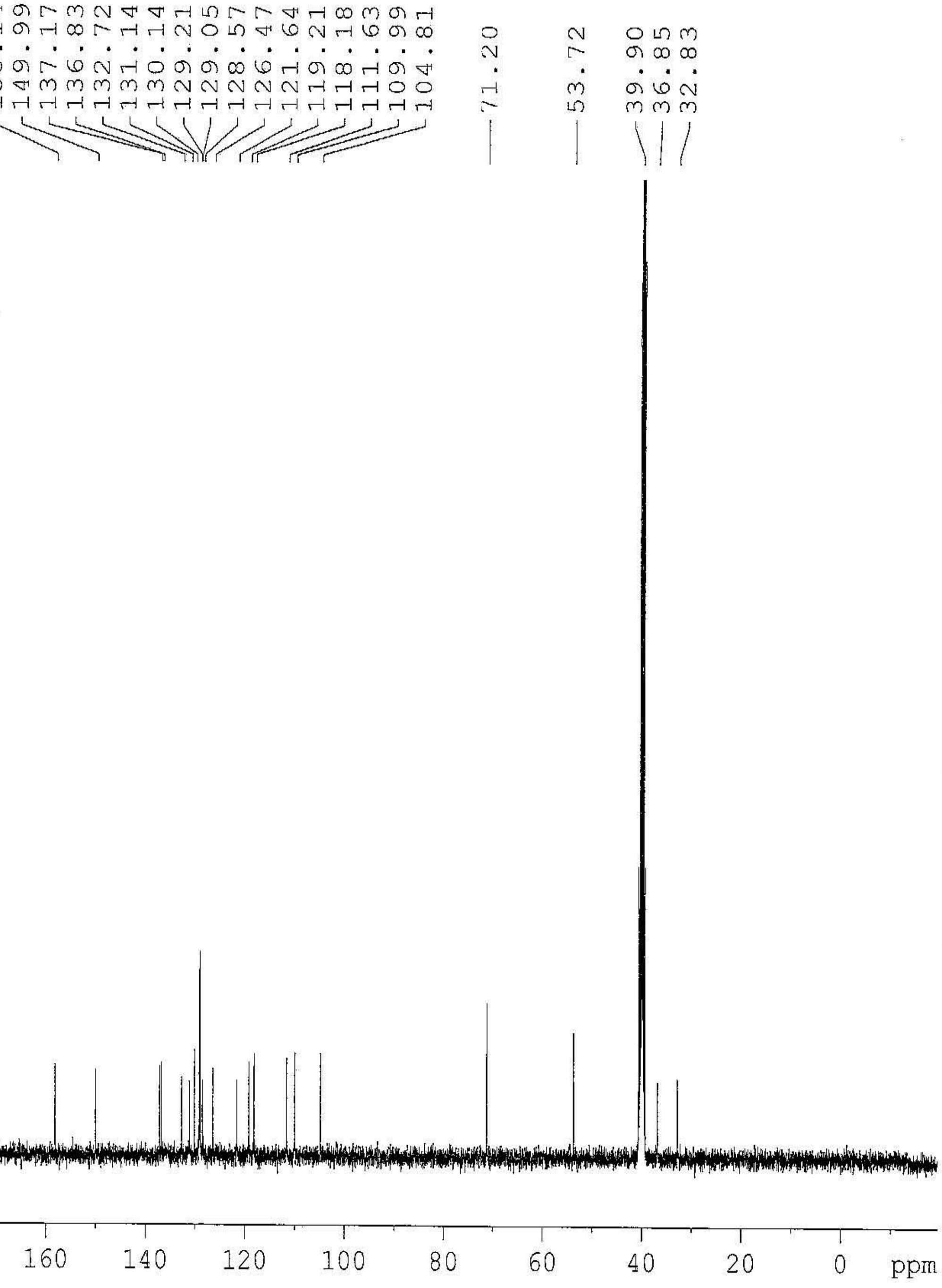

BRUKER

NAME	JRG-348-27_DMSO
EXPNO	2
PROCNO	1
Date_	20110627
Time_	15.30
INSTRUM	spect
PROBHD	5 mm PABBO BB-
PULPROG	zgpg30
TD	65536
SOLVENT	DMSO
NS	200
DS	4
SWH	24038.461 Hz
FIDRES	0.366798 Hz
AQ	1.3631988 sec
RG	203
DW	20.800 usec
DE	6.50 usec
TE	295.5 K
D1	3.00000000 sec
D11	0.03000000 sec
TD0	1

======== CHANNEL f1 ========

NUC1	13C
P1	10.00 usec
PL1	-1.59 dB
PL1W	51.07626343 W
SFO1	100.6479773 MHz

======== CHANNEL f2 ========

CPDPRG2	waltz16
NUC2	1H
PCPD2	75.00 usec
PL2	-1.00 dB
PL12	13.39 dB
PL13	20.00 dB
PL2W	11.09959412 W
PL12W	0.40393090 W
PL13W	0.08816721 W
SFO2	400.2316009 MHz
SI	32768
SF	100.6379140 MHz
WDW	EM
SSB	0
LB	1.00 Hz
GB	0
PC	1.40

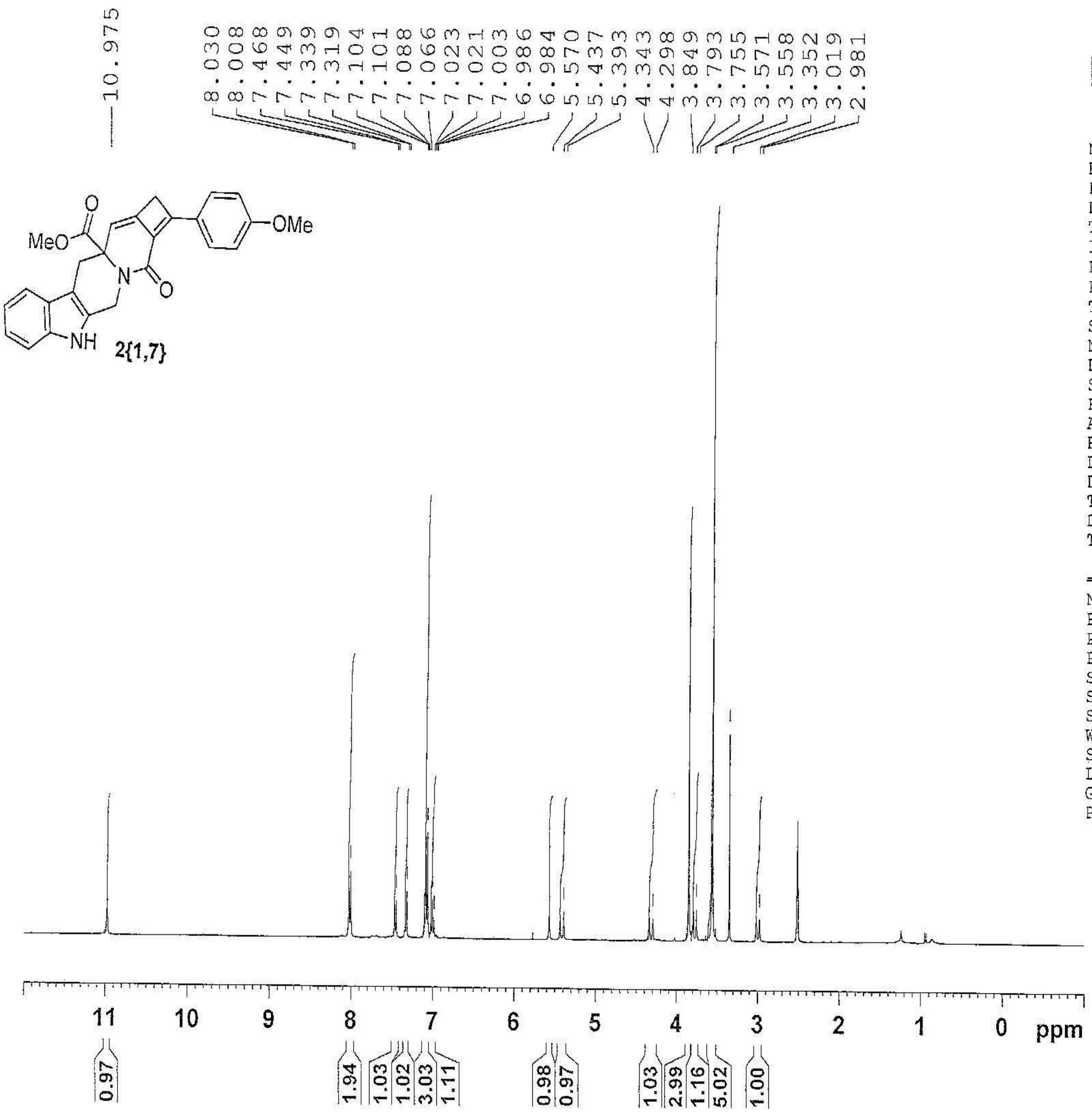

NAME	JRG-348-31_DMSO	
EXPNO	1	
PROCNO	1	
Date_	20110628	
Time	9.11	
INSTRUM	spect	
PROBHD	5 mm PABBO BB-	
PULPROG	zg30	
TD	65536	
SOLVENT	DMSO	
NS	5	
DS	2	
SWH	8223.685	Hz
FIDRES	0.125483	Hz
AQ	3.9846387	sec
RG	144	
DW	60.800	usec
DE	6.50	usec
TE	295.2	K
D1	2.00000000	sec
TD0	1	

======== CHANNEL f1 ========

NUC1	1H	
P1	14.31	usec
PL1	-1.00	dB
PL1W	11.09959412	W
SFO1	400.2324716	MHz
SI	32768	
SF	400.2300000	MHz
WDW	EM	
SSB	0	
LB	0.30	Hz
GB	0	
PC	1.00	

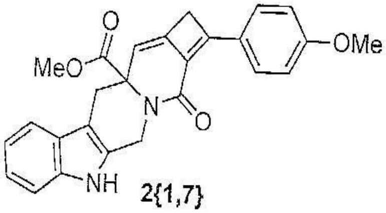

2{1,7}

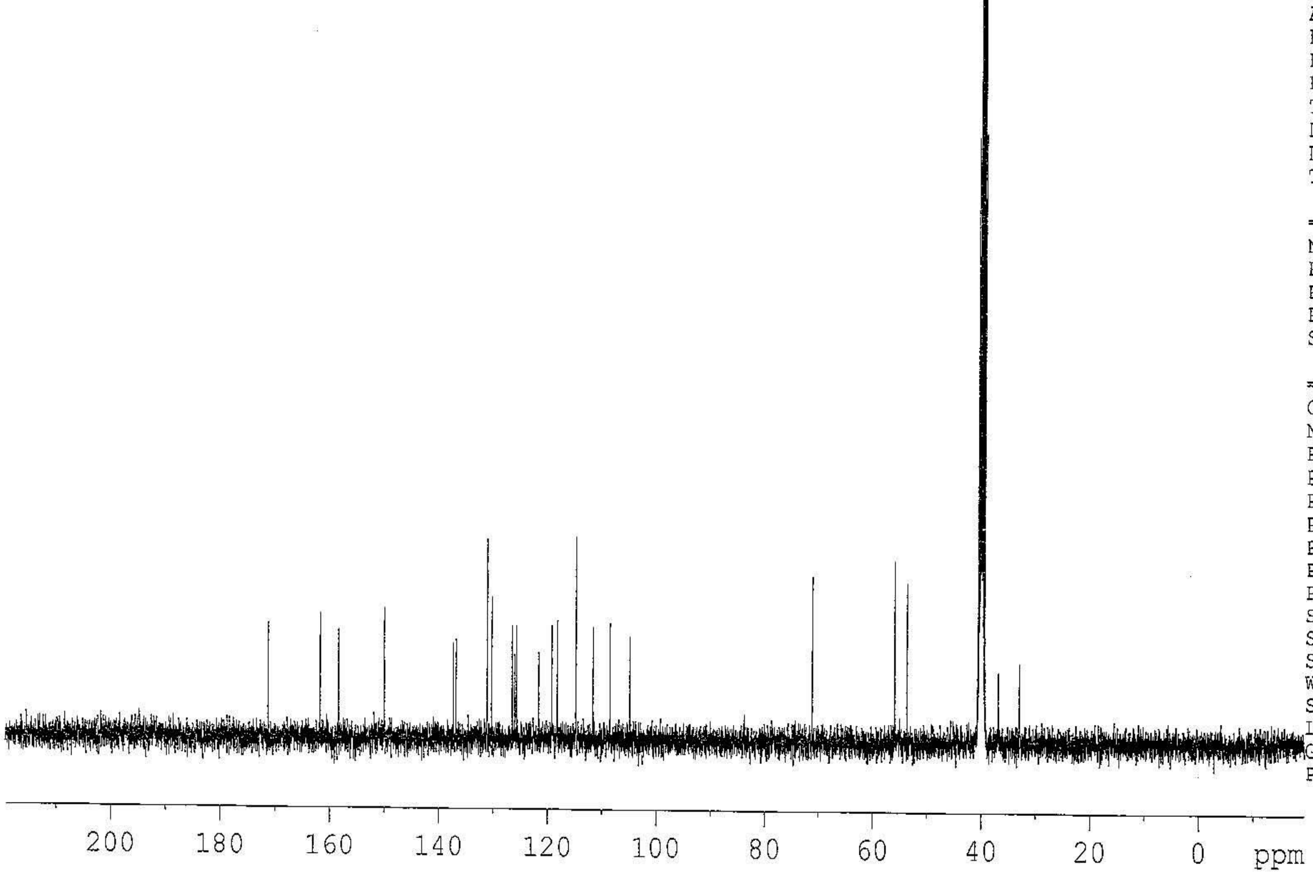

BRUKER

NAME	JRG-348-31_DMSO
EXPNO	2
PROCNO	1
Date_	20110628
Time_	9.35
INSTRUM	spect
PROBHD	5 mm PABBO BB-
PULPROG	zgpg30
TD	65536
SOLVENT	DMSO
NS	300
DS	4
SWH	24038.461 Hz
FIDRES	0.366798 Hz
AQ	1.3631988 sec
RG	203
DW	20.800 usec
DE	6.50 usec
TE	295.8 K
D1	3.00000000 sec
D11	0.03000000 sec
TD0	1

======== CHANNEL f1 ========

NUC1	13C
P1	10.00 usec
PL1	-1.59 dB
PL1W	51.07626343 W
SFO1	100.6479773 MHz

======== CHANNEL f2 ========

CPDPRG2	waltz16
NUC2	1H
PCPD2	75.00 usec
PL2	-1.00 dB
PL12	13.39 dB
PL13	20.00 dB
PL2W	11.09959412 W
PL12W	0.40393090 W
PL13W	0.08816721 W
SFO2	400.2316009 MHz
SI	32768
SF	100.6379140 MHz
WDW	EM
SSB	0
LB	1.00 Hz
GB	0
PC	1.40

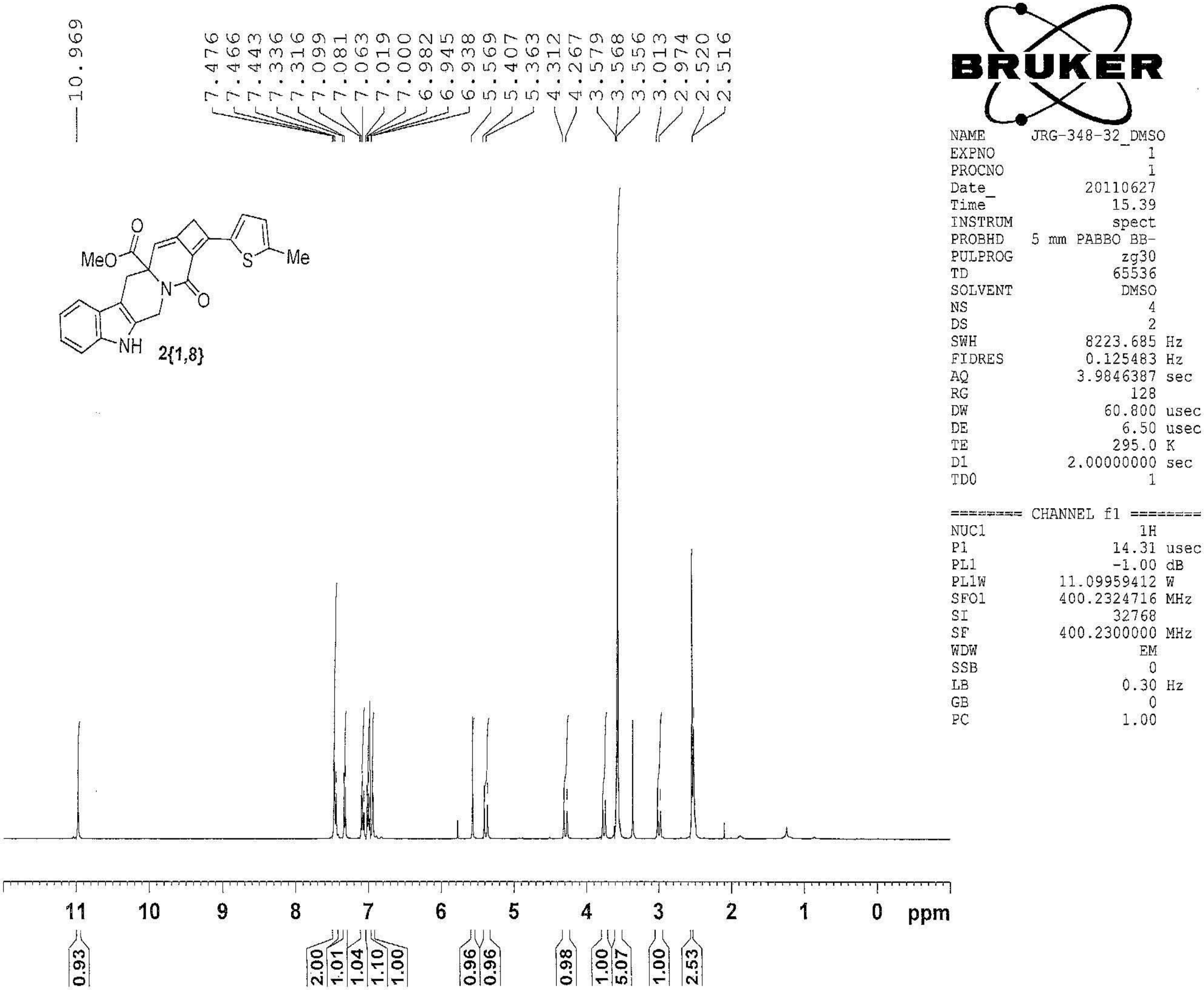
2{1,8}
10.969
7.476
7.466
7.443
7.336
7.316
7.099
7.081
7.063
7.019
7.000
6.982
6.945
6.938
5.569
5.407
5.363
4.312
4.267
3.579
3.568
3.556
3.013
2.974
2.520
2.516
BRUKER
NAME JRG-348-32_DMSO
EXPNO 1
PROCNO 1
Date_ 20110627
Time 15.39
INSTRUM spect
PROBHD 5 mm PABBO BB-
PULPROG zg30
TD 65536
SOLVENT DMSO
NS 4
DS 2
SWH 8223.685 Hz
FIDRES 0.125483 Hz
AQ 3.9846387 sec
RG 128
DW 60.800 usec
DE 6.50 usec
TE 295.0 K
D1 2.00000000 sec
TD0 1
======== CHANNEL f1 ========
NUC1 1H
P1 14.31 usec
PL1 -1.00 dB
PL1W 11.09959412 W
SFO1 400.2324716 MHz
SI 32768
SF 400.2300000 MHz
WDW EM
SSB 0
LB 0.30 Hz
GB 0
PC 1.00
11
10
9
8
7
6
5
4
3
2
1
0
ppm
0.93
2.00
1.01
1.04
1.10
1.00
0.96
0.96
0.98
1.00
5.07
1.00
2.53

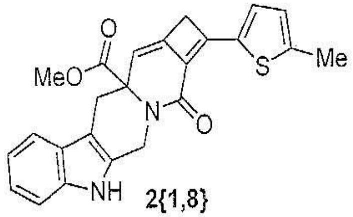

2{1,8}

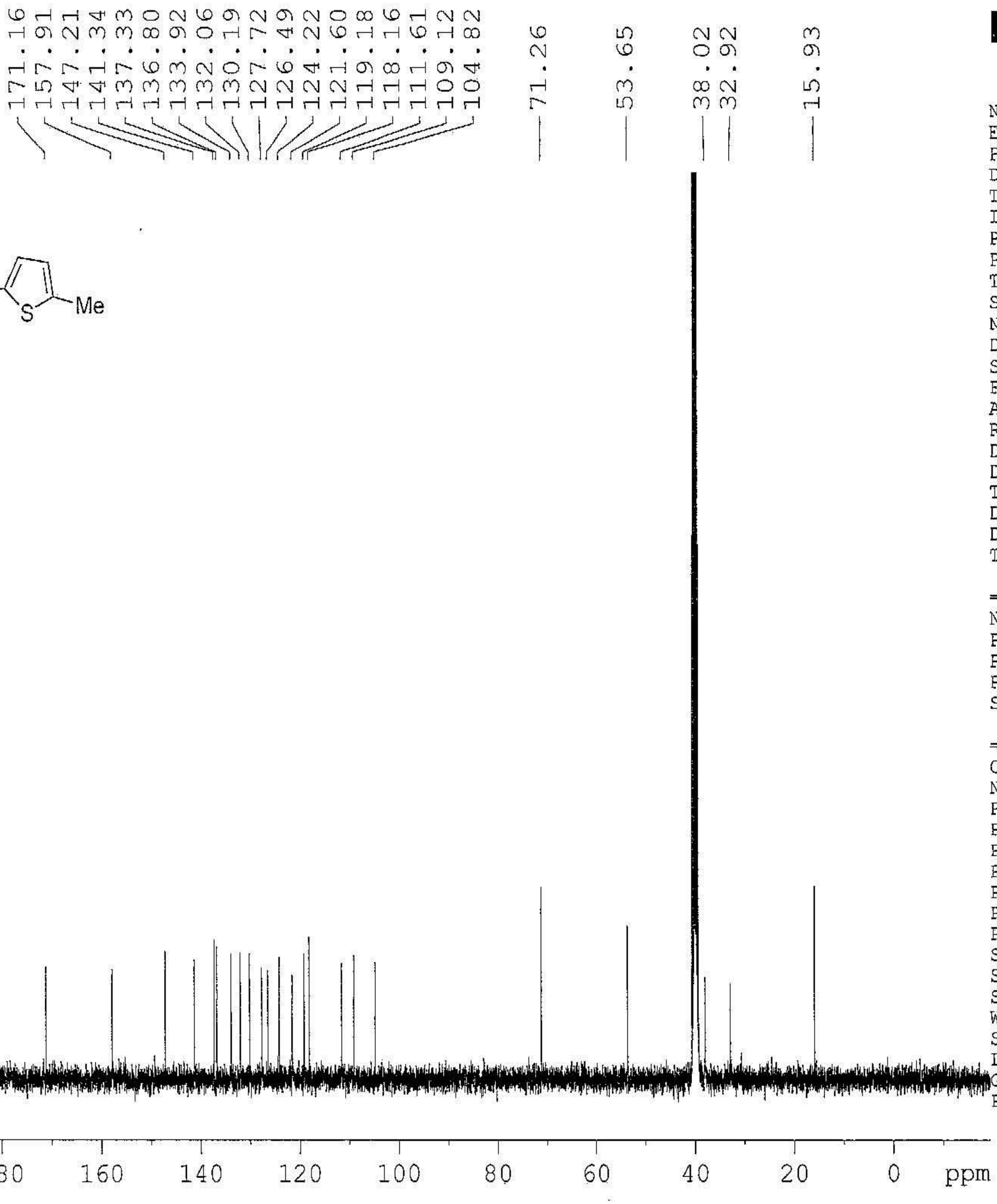

NAME	JRG-348-32_DMSO
EXPNO	2
PROCNO	1
Date_	20110627
Time_	16.03
INSTRUM	spect
PROBHD	5 mm PABBO BB-
PULPROG	zgpg30
TD	65536
SOLVENT	DMSO
NS	299
DS	4
SWH	24038.461 Hz
FIDRES	0.366798 Hz
AQ	1.3631988 sec
RG	203
DW	20.800 usec
DE	6.50 usec
TE	295.6 K
D1	3.00000000 sec
D11	0.03000000 sec
TD0	1

======== CHANNEL f1 ========

NUC1	13C
P1	10.00 usec
PL1	-1.59 dB
PL1W	51.07626343 W
SFO1	100.6479773 MHz

======== CHANNEL f2 ========

CPDPRG2	waltz16
NUC2	1H
PCPD2	75.00 usec
PL2	-1.00 dB
PL12	13.39 dB
PL13	20.00 dB
PL2W	11.09959412 W
PL12W	0.40393090 W
PL13W	0.08816721 W
SFO2	400.2316009 MHz
SI	32768
SF	100.6379140 MHz
WDW	EM
SSB	0
LB	1.00 Hz
GB	0
PC	1.40

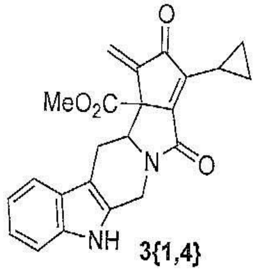

3{1,4}

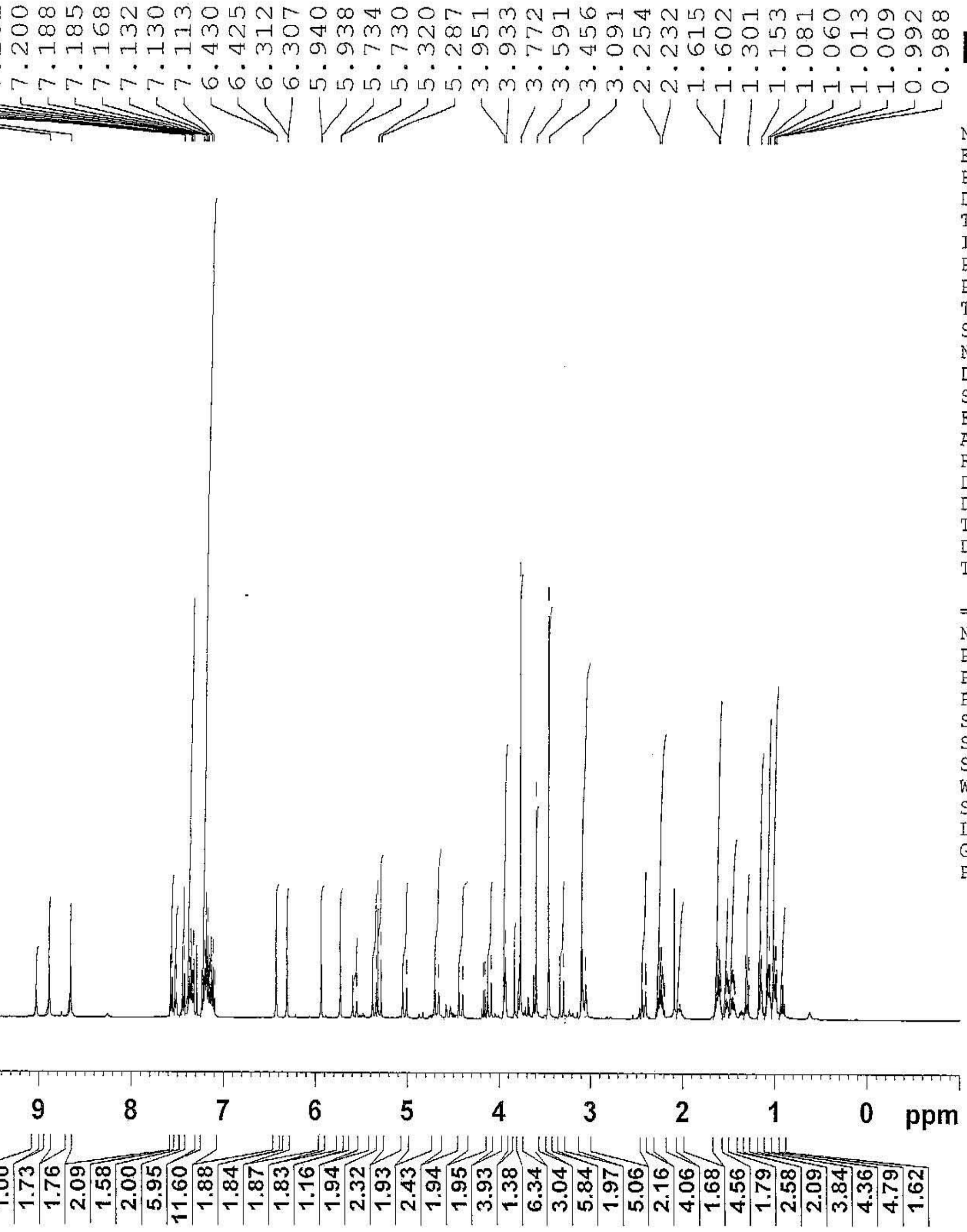

BRUKER

NAME	JRG-348-74re
EXPNO	1
PROCNO	1
Date_	20110726
Time_	23.36
INSTRUM	spect
PROBHD	5 mm PABBO BB-
PULPROG	zg30
TD	65536
SOLVENT	CDCl3
NS	6
DS	2
SWH	8223.685 Hz
FIDRES	0.125483 Hz
AQ	3.9846387 sec
RG	64
DW	60.800 usec
DE	6.50 usec
TE	295.3 K
D1	2.00000000 sec
TD0	1

======== CHANNEL f1 ========

NUC1	1H
P1	14.31 usec
PL1	-1.00 dB
PL1W	11.09959412 W
SFO1	400.2324716 MHz
SI	32768
SF	400.2300000 MHz
WDW	EM
SSB	0
LB	0.30 Hz
GB	0
PC	1.00

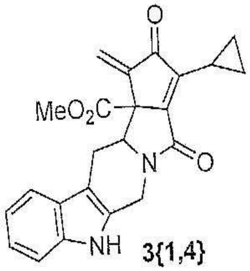

3{1,4}

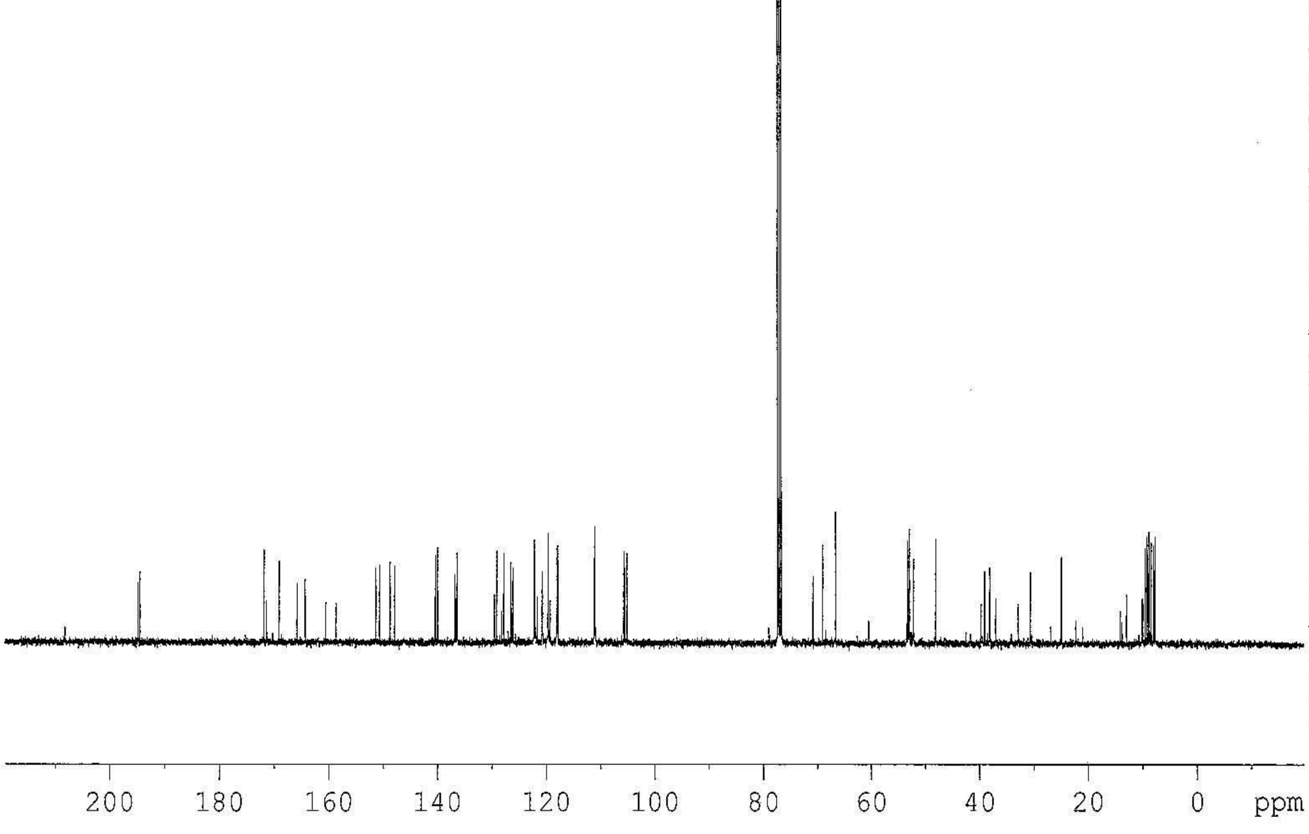

BRUKER

NAME	JRG-348-74re	
EXPNO	2	
PROCNO	1	
Date_	20110727	
Time_	0.15	
INSTRUM	spect	
PROBHD	5 mm PABBO BB-	
PULPROG	zgpg30	
TD	65536	
SOLVENT	CDCl3	
NS	511	
DS	4	
SWH	24038.461	Hz
FIDRES	0.366798	Hz
AQ	1.3631988	sec
RG	203	
DW	20.800	usec
DE	6.50	usec
TE	295.6	K
D1	3.00000000	sec
D11	0.03000000	sec
TD0	1	

======== CHANNEL f1 ========

NUC1	13C	
P1	10.00	usec
PL1	-1.59	dB
PL1W	51.07626343	W
SFO1	100.6479773	MHz

======== CHANNEL f2 ========

CPDPRG2	waltz16	
NUC2	1H	
PCPD2	75.00	usec
PL2	-1.00	dB
PL12	13.39	dB
PL13	20.00	dB
PL2W	11.09959412	W
PL12W	0.40393090	W
PL13W	0.08816721	W
SFO2	400.2316009	MHz
SI	32768	
SF	100.6379251	MHz
WDW	EM	
SSB	0	
LB	1.00	Hz
GB	0	
PC	1.40	

JRG-348-75re_p
CDCl3,   1H

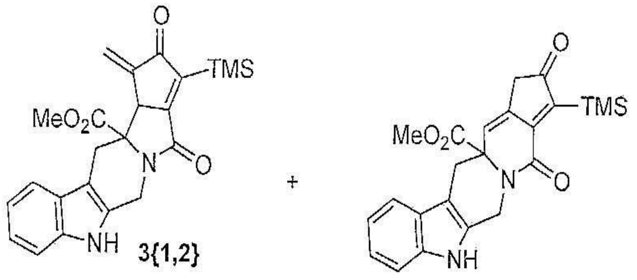

3{1,2}

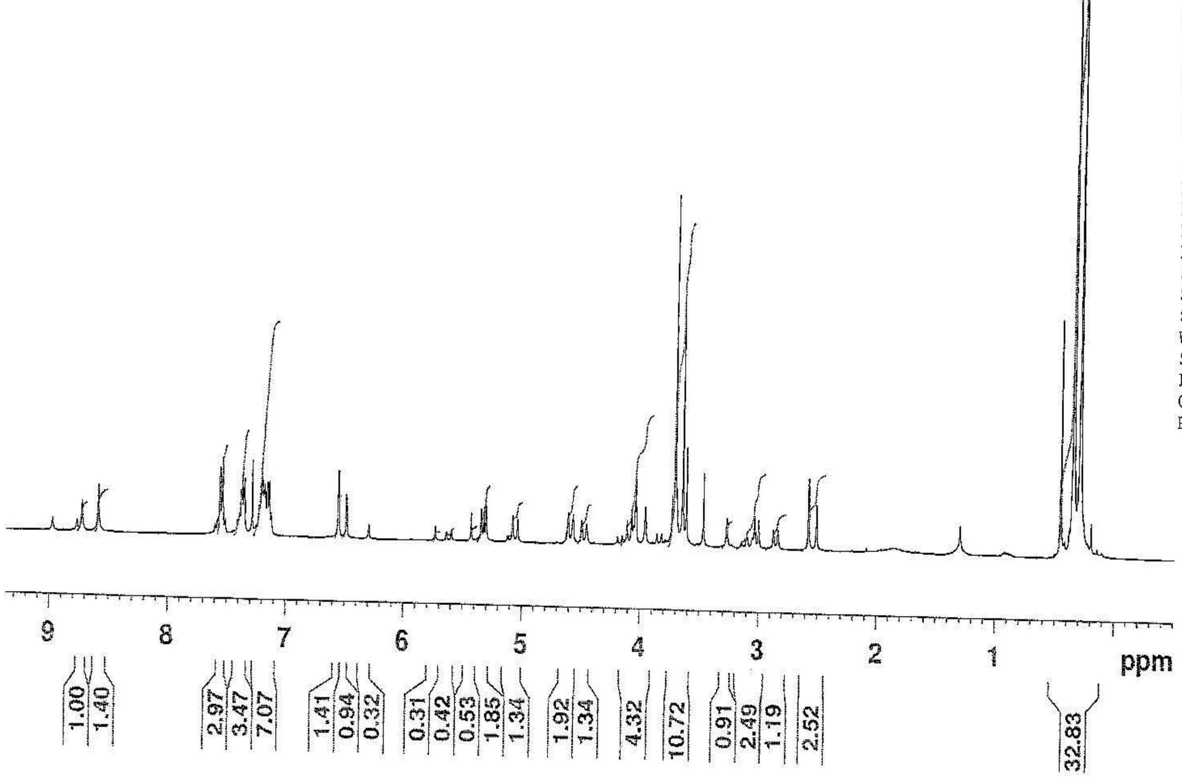

NAME	JRG-348-75re
EXPNO	1
PROCNO	1
Date_	20110726
Time	22.06
INSTRUM	spect
PROBHD	5 mm PABBO BB-
PULPROG	zg30
TD	65536
SOLVENT	CDCl3
NS	8
DS	2
SWH	8223.685 Hz
FIDRES	0.125483 Hz
AQ	3.9846387 sec
RG	80.6
DW	60.800 usec
DE	6.50 usec
TE	294.8 K
D1	2.00000000 sec
TD0	1

======== CHANNEL f1 ========

NUC1	1H
P1	14.31 usec
PL1	-1.00 dB
PL1W	11.09959412 W
SFO1	400.2324716 MHz
SI	32768
SF	400.2300000 MHz
WDW	EM
SSB	0
LB	0.30 Hz
GB	0
PC	1.00

JRG-348-75re_C
CDCl3 13C

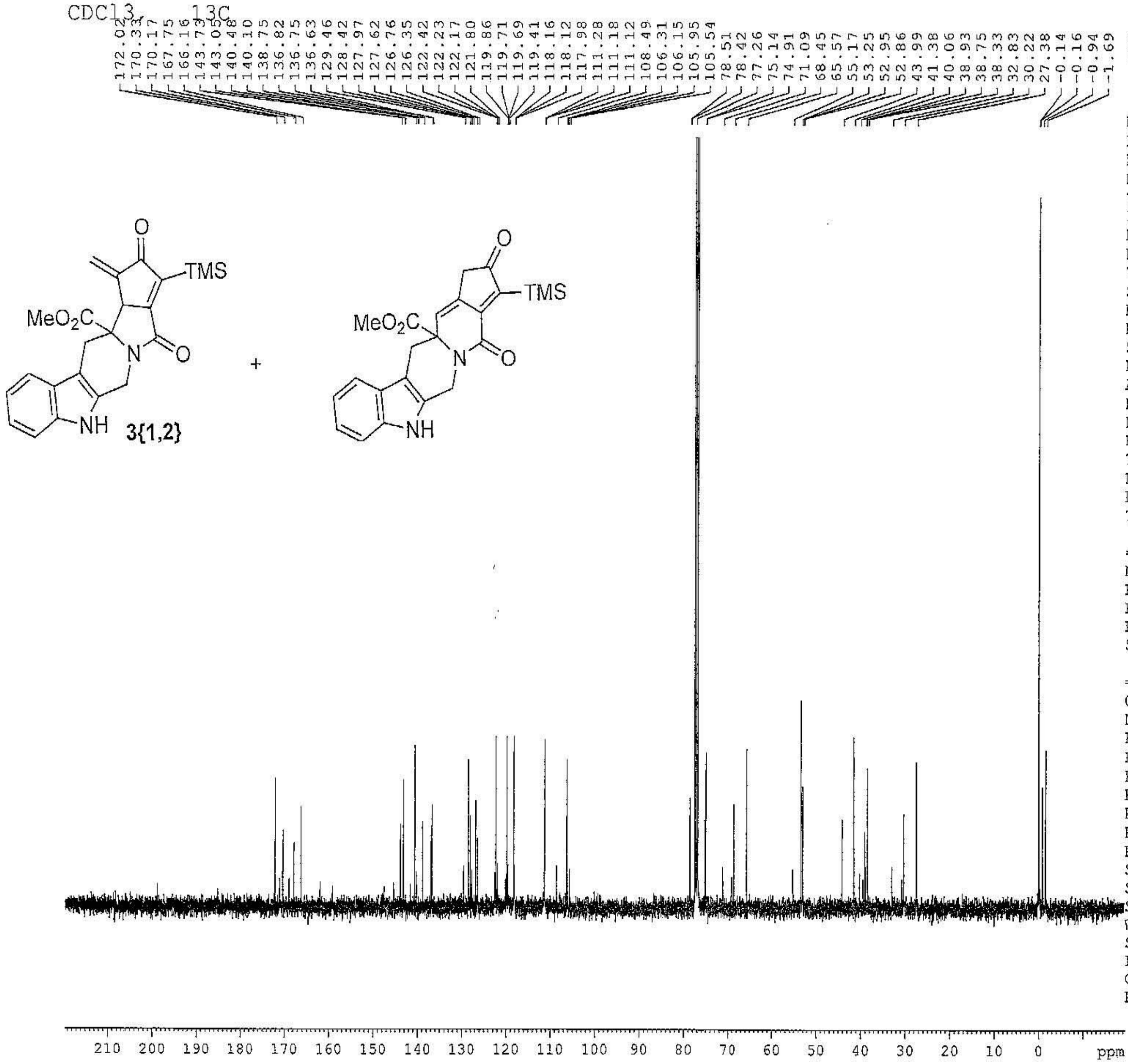

NAME	JRG-348-75re	
EXPNO	2	
PROCNO	1	
Date_	20110726	
Time	22.39	
INSTRUM	spect	
PROBHD	5 mm PABBO BB-	
PULPROG	zgpg30	
TD	65536	
SOLVENT	CDCl3	
NS	507	
DS	4	
SWH	24038.461	Hz
FIDRES	0.366798	Hz
AQ	1.3631988	sec
RG	203	
DW	20.800	usec
DE	6.50	usec
TE	295.6	K
D1	3.00000000	sec
D11	0.03000000	sec
TD0	1	

======== CHANNEL f1 ========

NUC1	13C	
P1	10.00	usec
PL1	-1.59	dB
PL1W	51.07626343	W
SFO1	100.6479773	MHz

======== CHANNEL f2 ========

CPDPRG2	waltz16	
NUC2	1H	
PCPD2	75.00	usec
PL2	-1.00	dB
PL12	13.39	dB
PL13	20.00	dB
PL2W	11.09959412	W
PL12W	0.40393090	W
PL13W	0.08816721	W
SFO2	400.2316009	MHz
SI	32768	
SF	100.6379140	MHz
WDW	EM	
SSB	0	
LB	1.00	Hz
GB	0	
PC	1.40	

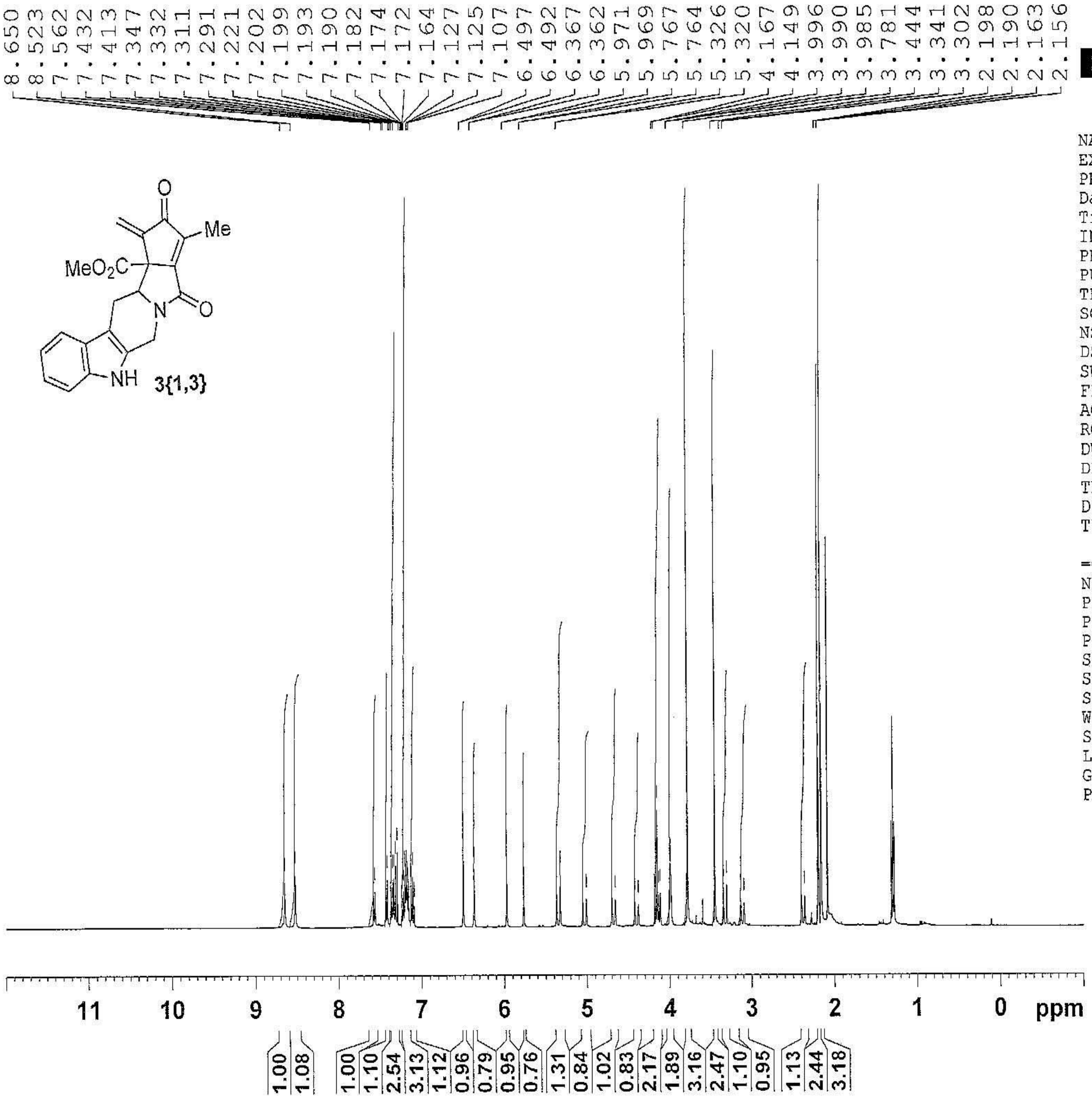

BRUKER

NAME	JRG-348-37re
EXPNO	1
PROCNO	1
Date_	20110726
Time	22.52
INSTRUM	spect
PROBHD	5 mm PABBO BB-
PULPROG	zg30
TD	65536
SOLVENT	CDCl3
NS	8
DS	2
SWH	8223.685 Hz
FIDRES	0.125483 Hz
AQ	3.9846387 sec
RG	80.6
DW	60.800 usec
DE	6.50 usec
TE	295.1 K
D1	2.00000000 sec
TD0	1

======== CHANNEL f1 ========

NUC1	1H
P1	14.31 usec
PL1	-1.00 dB
PL1W	11.09959412 W
SFO1	400.2324716 MHz
SI	32768
SF	400.2300000 MHz
WDW	EM
SSB	0
LB	0.30 Hz
GB	0
PC	1.00

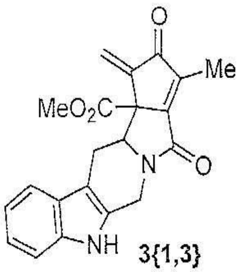
3{1,3}

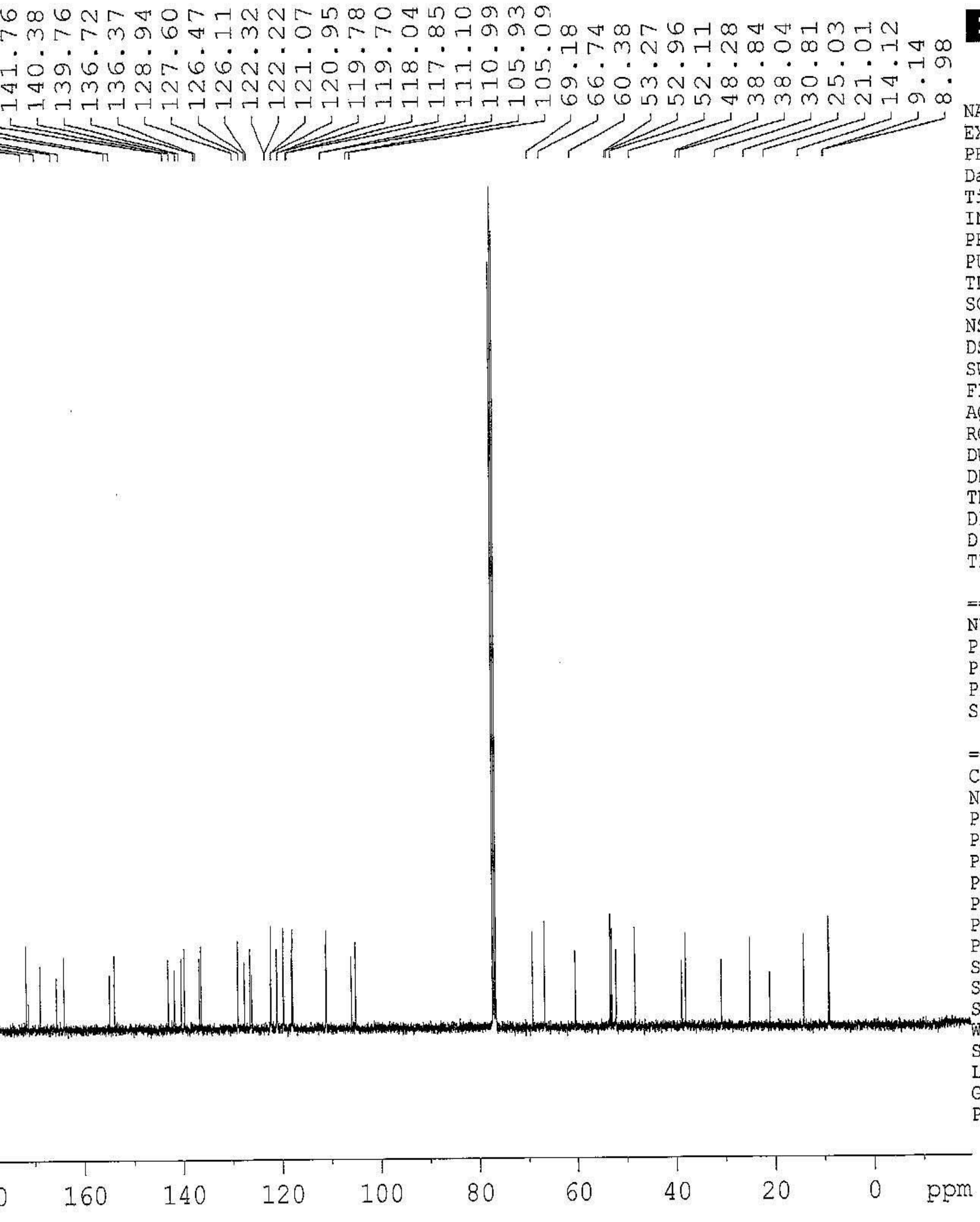

BRUKER

NAME	JRG-348-37re	
EXPNO	2	
PROCNO	1	
Date_	20110726	
Time_	23.13	
INSTRUM	spect	
PROBHD	5 mm PABBO BB-	
PULPROG	zgpg30	
TD	65536	
SOLVENT	CDCl3	
NS	252	
DS	4	
SWH	24038.461	Hz
FIDRES	0.366798	Hz
AQ	1.3631988	sec
RG	203	
DW	20.800	usec
DE	6.50	usec
TE	295.6	K
D1	3.00000000	sec
D11	0.03000000	sec
TD0	1	

======== CHANNEL f1 ========

NUC1	13C	
P1	10.00	usec
PL1	-1.59	dB
PL1W	51.07626343	W
SFO1	100.6479773	MHz

======== CHANNEL f2 ========

CPDPRG2	waltz16	
NUC2	1H	
PCPD2	75.00	usec
PL2	-1.00	dB
PL12	13.39	dB
PL13	20.00	dB
PL2W	11.09959412	W
PL12W	0.40393090	W
PL13W	0.08816721	W
SFO2	400.2316009	MHz
SI	32768	
SF	100.6379241	MHz
WDW	EM	
SSB	0	
LB	1.00	Hz
GB	0	
PC	1.40	

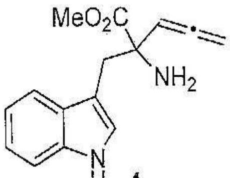

4

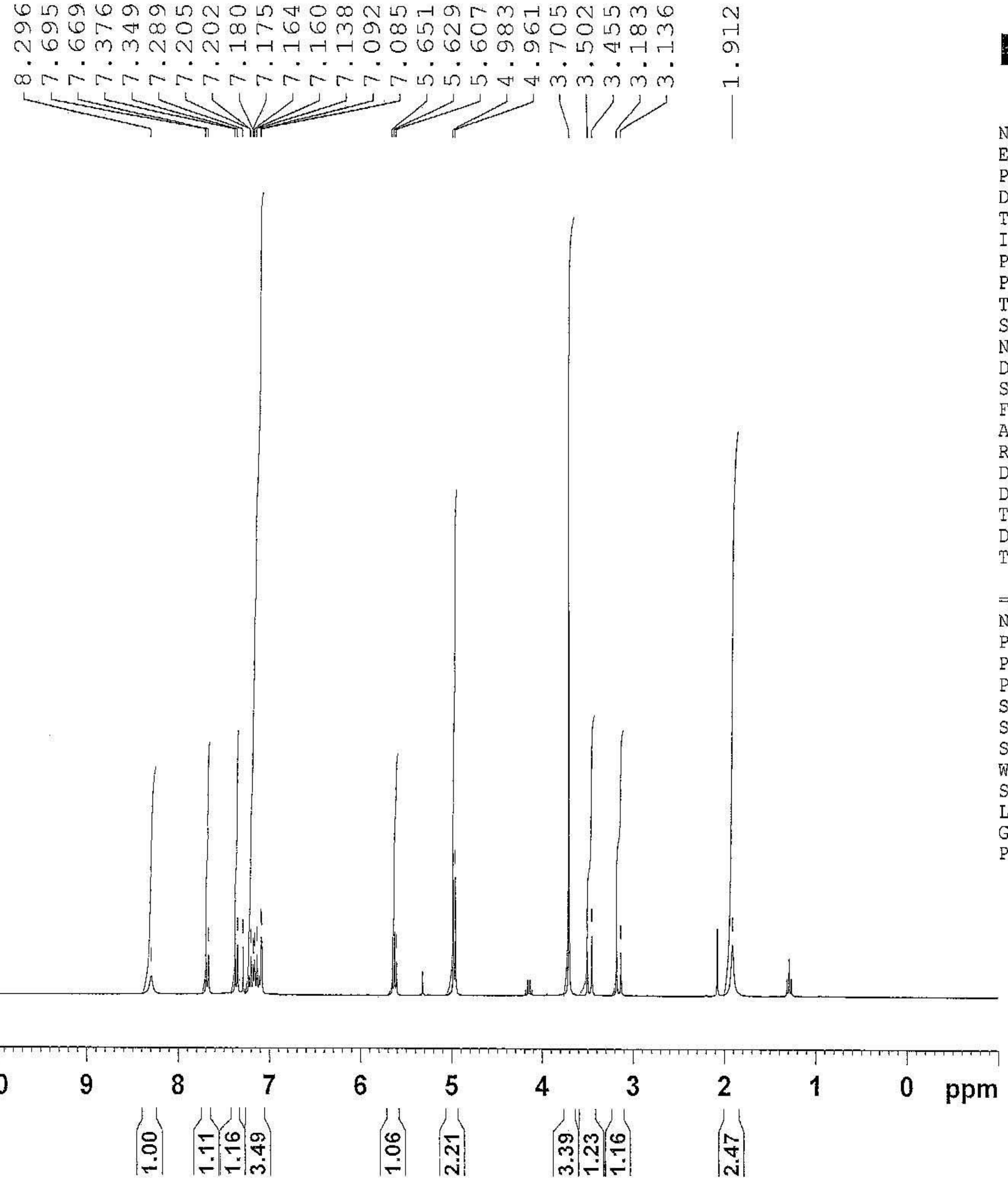

NAME	JRG-348-21
EXPNO	1
PROCNO	1
Date_	20110609
Time	16.57
INSTRUM	spect
PROBHD	5 mm Multinucl
PULPROG	zg30
TD	32768
SOLVENT	CDCl3
NS	16
DS	2
SWH	6188.119 Hz
FIDRES	0.188846 Hz
AQ	2.6477044 sec
RG	128
DW	80.800 usec
DE	6.50 usec
TE	297.4 K
D1	1.00000000 sec
TD0	1

======== CHANNEL f1 ========

NUC1	1H
P1	13.20 usec
PL1	-2.00 dB
PL1W	18.20942116 W
SFO1	300.2318540 MHz
SI	32768
SF	300.2300000 MHz
WDW	EM
SSB	0
LB	0.10 Hz
GB	0
PC	1.00

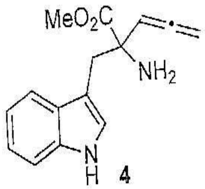

4

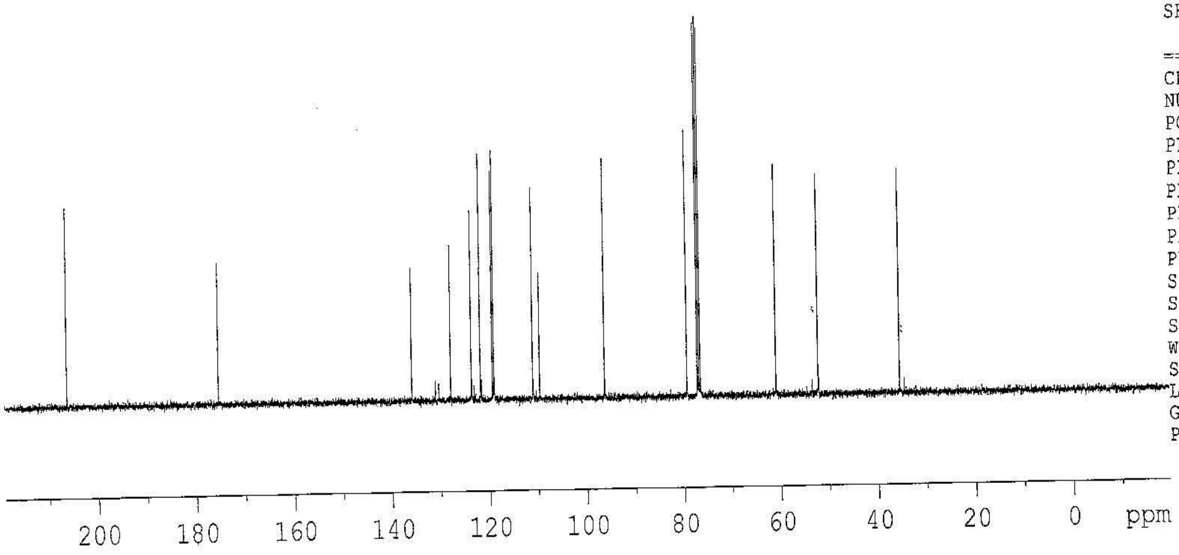

BRUKER

NAME	JRG-348-21_carbon
EXPNO	1
PROCNO	1
Date_	20110715
Time	9.23
INSTRUM	spect
PROBHD	5 mm PABBO BB-
PULPROG	zgpg30
TD	65536
SOLVENT	CDCl3
NS	128
DS	4
SWH	24038.461 Hz
FIDRES	0.366798 Hz
AQ	1.3631988 sec
RG	203
DW	20.800 usec
DE	6.50 usec
TE	295.7 K
D1	3.00000000 sec
D11	0.03000000 sec
TD0	1

======== CHANNEL f1 ========

NUC1	13C
P1	10.00 usec
PL1	-1.59 dB
PL1W	51.07626343 W
SFO1	100.6479773 MHz

======== CHANNEL f2 ========

CPDPRG2	waltz16
NUC2	1H
PCPD2	75.00 usec
PL2	-1.00 dB
PL12	13.39 dB
PL13	20.00 dB
PL2W	11.09959412 W
PL12W	0.40393090 W
PL13W	0.08816721 W
SFO2	400.2316009 MHz
SI	32768
SF	100.6379297 MHz
WDW	EM
SSB	0
LB	1.00 Hz
GB	0
PC	1.40

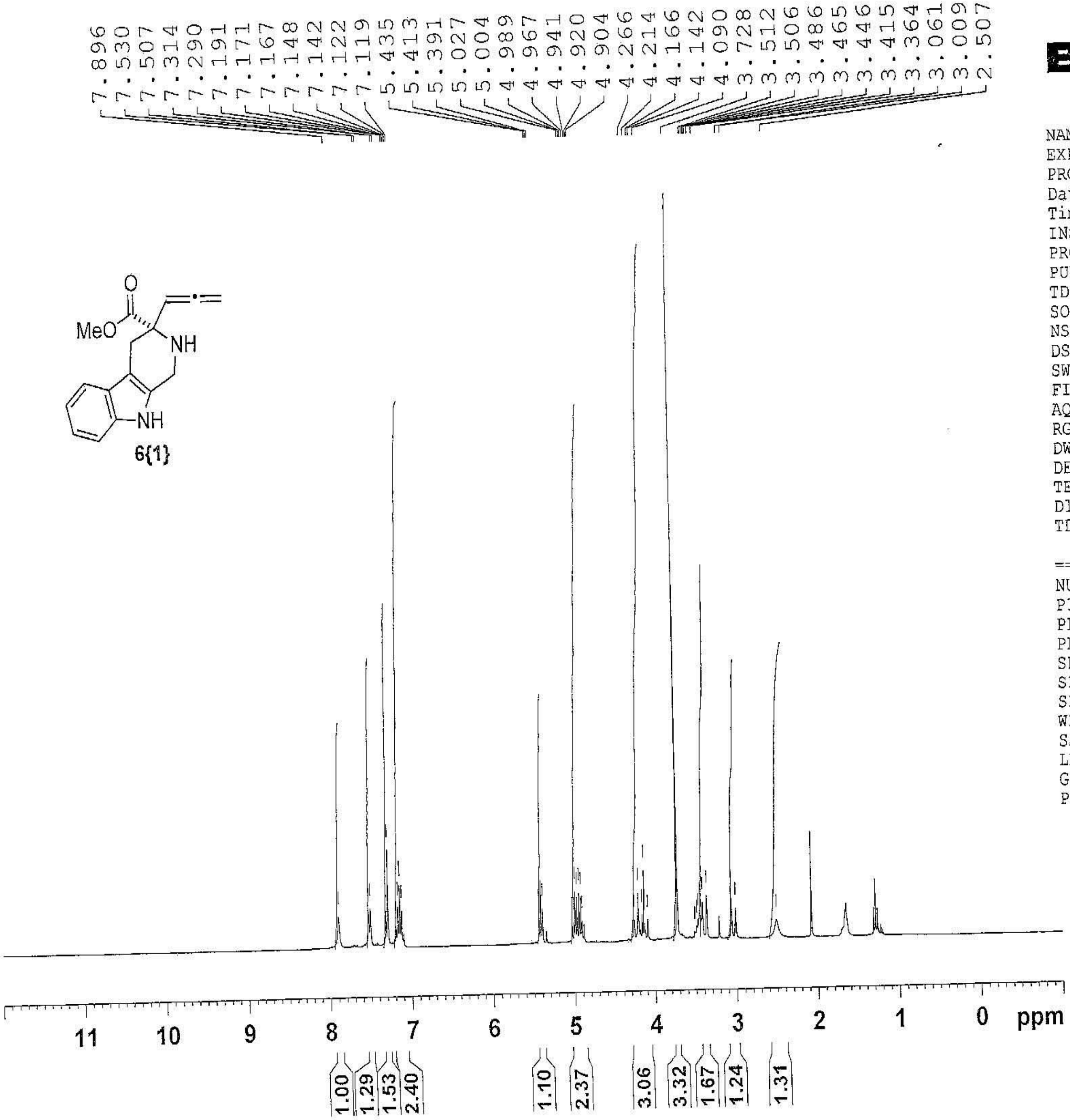

6{1}

BRUKER

NAME	JRG-329-81
EXPNO	1
PROCNO	1
Date_	20110429
Time	10.36
INSTRUM	spect
PROBHD	5 mm Multinucl
PULPROG	zg30
TD	32768
SOLVENT	CDCl3
NS	16
DS	2
SWH	6188.119 Hz
FIDRES	0.188846 Hz
AQ	2.6477044 sec
RG	128
DW	80.800 usec
DE	6.50 usec
TE	294.2 K
D1	1.00000000 sec
TD0	1

======== CHANNEL f1 ========

NUC1	1H
P1	13.20 usec
PL1	-2.00 dB
PL1W	18.20942116 W
SFO1	300.2318540 MHz
SI	32768
SF	300.2300000 MHz
WDW	EM
SSB	0
LB	0.10 Hz
GB	0
PC	1.00

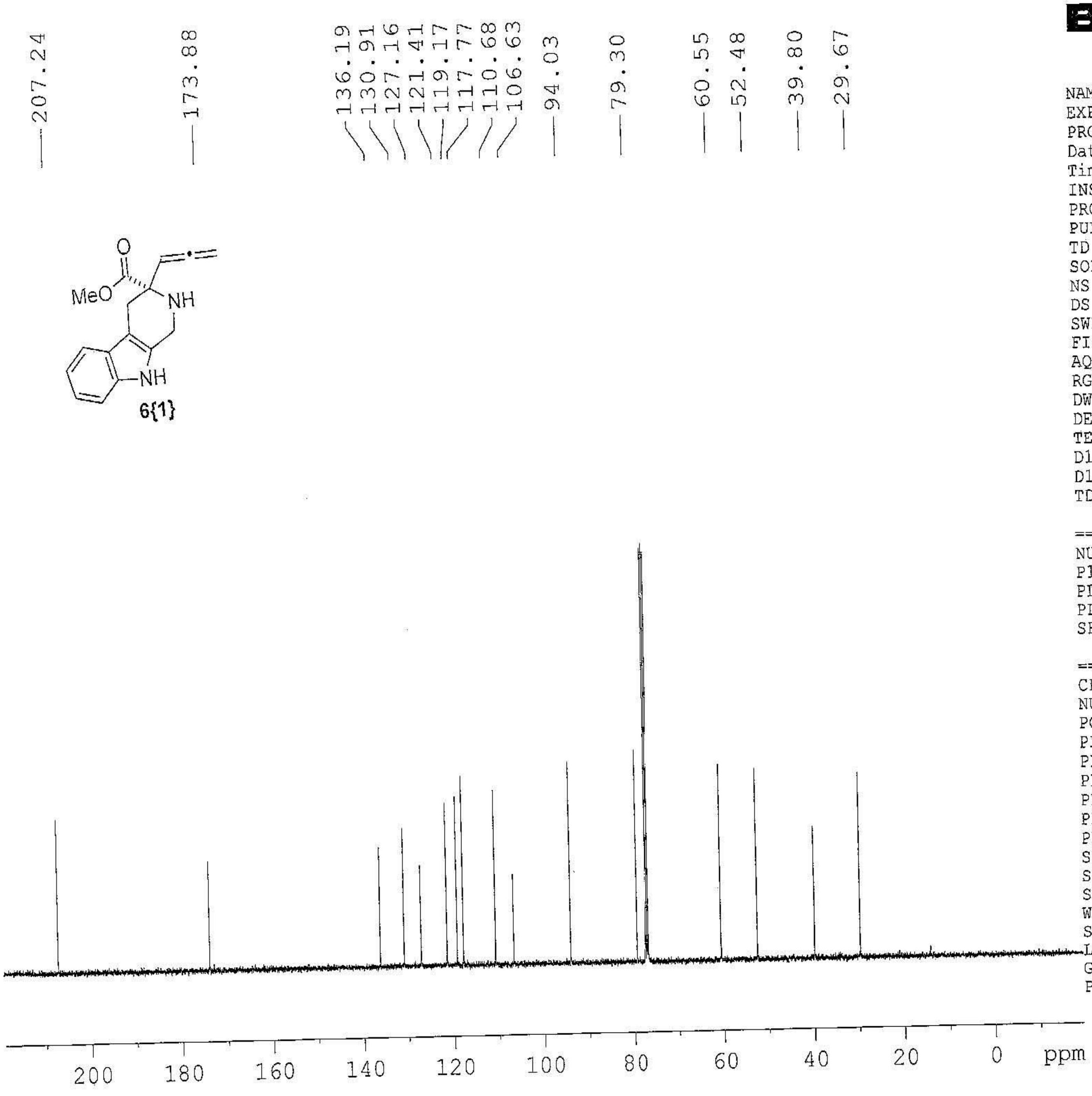

BRUKER

NAME	JRG-348-25_carbon
EXPNO	1
PROCNO	1
Date_	20110715
Time	9.48
INSTRUM	spect
PROBHD	5 mm PABBO BB-
PULPROG	zgpg30
TD	65536
SOLVENT	CDCl3
NS	248
DS	4
SWH	24038.461 Hz
FIDRES	0.366798 Hz
AQ	1.3631988 sec
RG	203
DW	20.800 usec
DE	6.50 usec
TE	295.8 K
D1	3.00000000 sec
D11	0.03000000 sec
TD0	1

======== CHANNEL f1 ========

NUC1	13C
P1	10.00 usec
PL1	-1.59 dB
PL1W	51.07626343 W
SFO1	100.6479773 MHz

======== CHANNEL f2 ========

CPDPRG2	waltz16
NUC2	1H
PCPD2	75.00 usec
PL2	-1.00 dB
PL12	13.39 dB
PL13	20.00 dB
PL2W	11.09959412 W
PL12W	0.40393090 W
PL13W	0.08816721 W
SFO2	400.2316009 MHz
SI	32768
SF	100.6379253 MHz
WDW	EM
SSB	0
LB	1.00 Hz
GB	0
PC	1.40

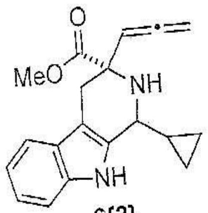

6{2}

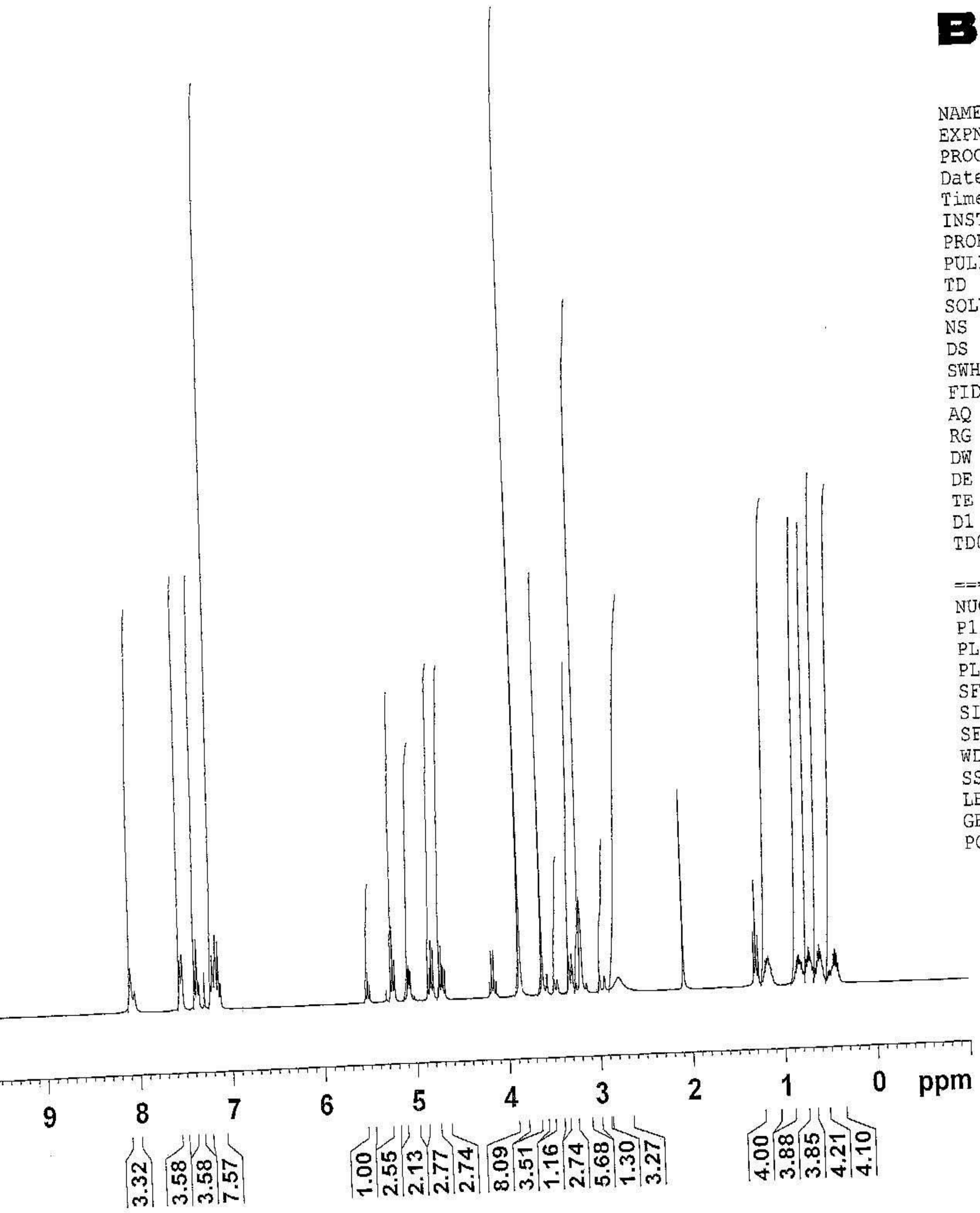

NAME	JRG-329-79
EXPNO	1
PROCNO	1
Date_	20110429
Time_	10.47
INSTRUM	spect
PROBHD	5 mm Multinucl
PULPROG	zg30
TD	32768
SOLVENT	CDCl3
NS	16
DS	2
SWH	6188.119 Hz
FIDRES	0.188846 Hz
AQ	2.6477044 sec
RG	57
DW	80.800 usec
DE	6.50 usec
TE	294.4 K
D1	1.00000000 sec
TD0	1

======== CHANNEL f1 ========

NUC1	1H
P1	13.20 usec
PL1	-2.00 dB
PL1W	18.20942116 W
SFO1	300.2318540 MHz
SI	32768
SF	300.2300000 MHz
WDW	EM
SSB	0
LB	0.10 Hz
GB	0
PC	1.00

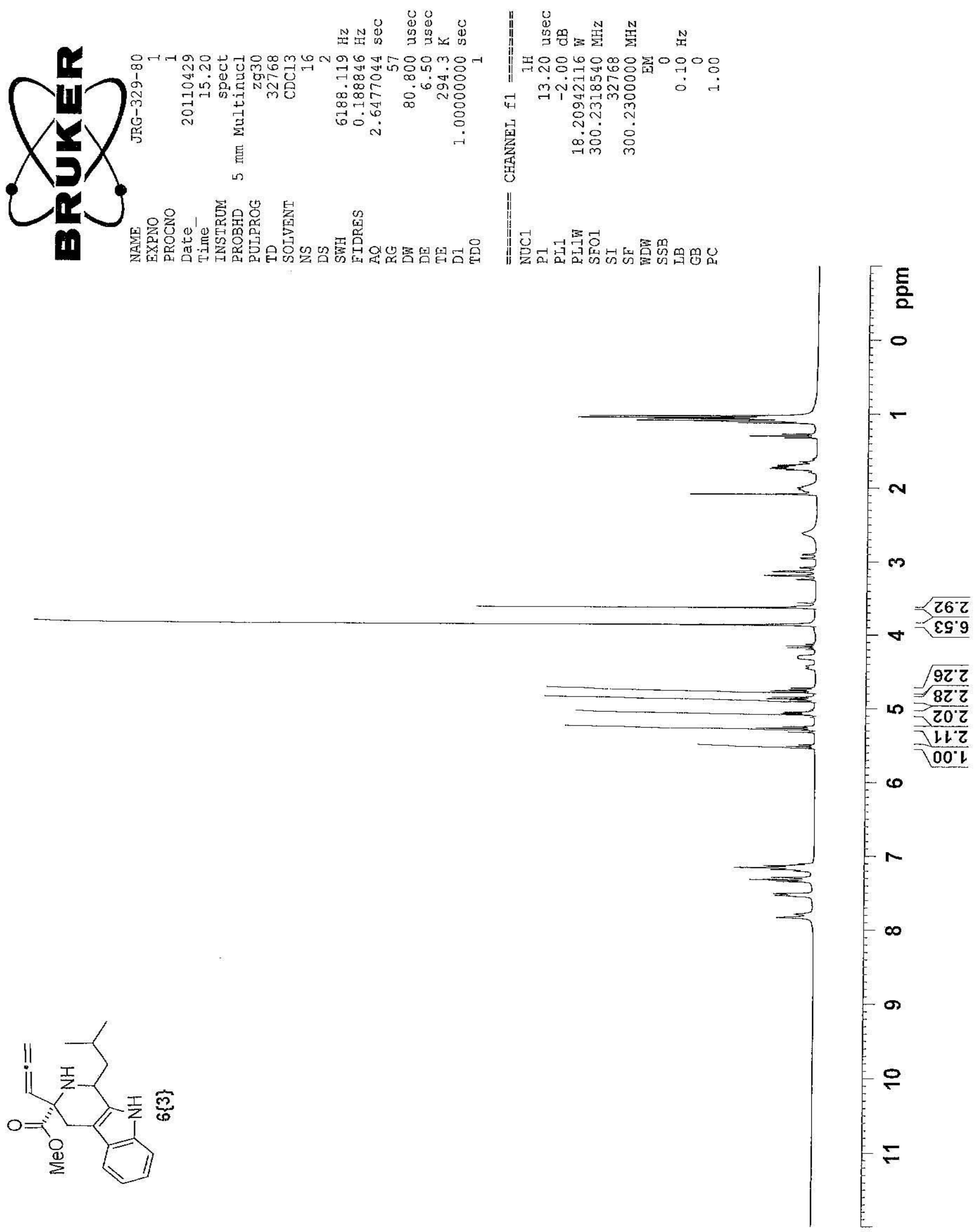
BRUKER
NAME JRG-329-80
EXPNO 1
PROCNO 1
Date_ 20110429
Time_ 15.20
INSTRUM spect
PROBHD 5 mm Multinucl
PULPROG zg30
TD 32768
SOLVENT CDCl3
NS 16
DS 2
SWH 6188.119 Hz
FIDRES 0.188846 Hz
AQ 2.6477044 sec
RG 57
DW 80.800 usec
DE 6.50 usec
TE 294.3 K
D1 1.00000000 sec
TD0 1
======== CHANNEL f1 ========
NUC1 1H
P1 13.20 usec
PL1 -2.00 dB
PL1W 18.20942116 W
SFO1 300.2318540 MHz
SI 32768
SF 300.2300000 MHz
WDW EM
SSB 0
LB 0.10 Hz
GB 0
PC 1.00
ppm
2.92
6.53
2.26
2.28
2.02
2.11
1.00
MeO
O
NH
NH
6{3}

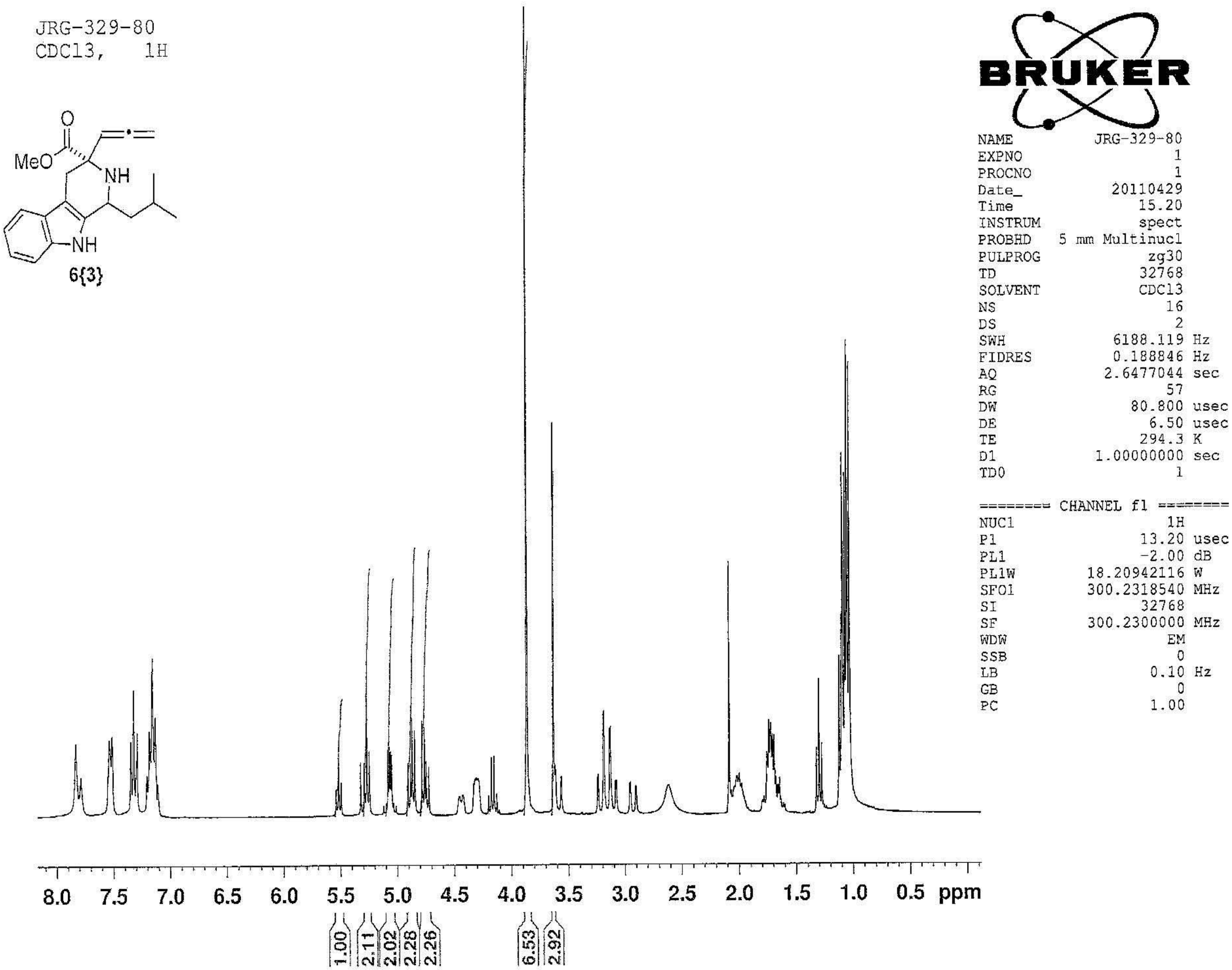
JRG-329-80
CDCl3, 1H
MeO
O
NH
NH
6{3}
BRUKER
NAME JRG-329-80
EXPNO 1
PROCNO 1
Date_ 20110429
Time 15.20
INSTRUM spect
PROBHD 5 mm Multinucl
PULPROG zg30
TD 32768
SOLVENT CDCl3
NS 16
DS 2
SWH 6188.119 Hz
FIDRES 0.188846 Hz
AQ 2.6477044 sec
RG 57
DW 80.800 usec
DE 6.50 usec
TE 294.3 K
D1 1.00000000 sec
TD0 1
======== CHANNEL f1 ========
NUC1 1H
P1 13.20 usec
PL1 -2.00 dB
PL1W 18.20942116 W
SFO1 300.2318540 MHz
SI 32768
SF 300.2300000 MHz
WDW EM
SSB 0
LB 0.10 Hz
GB 0
PC 1.00
8.0 7.5 7.0 6.5 6.0 5.5 5.0 4.5 4.0 3.5 3.0 2.5 2.0 1.5 1.0 0.5 ppm
1.00
2.11
2.02
2.28
2.26
6.53
2.92

JRG-348-40
CDCl3, 1H

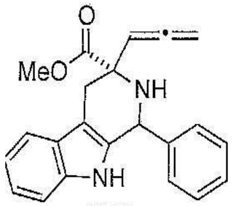

6{4}

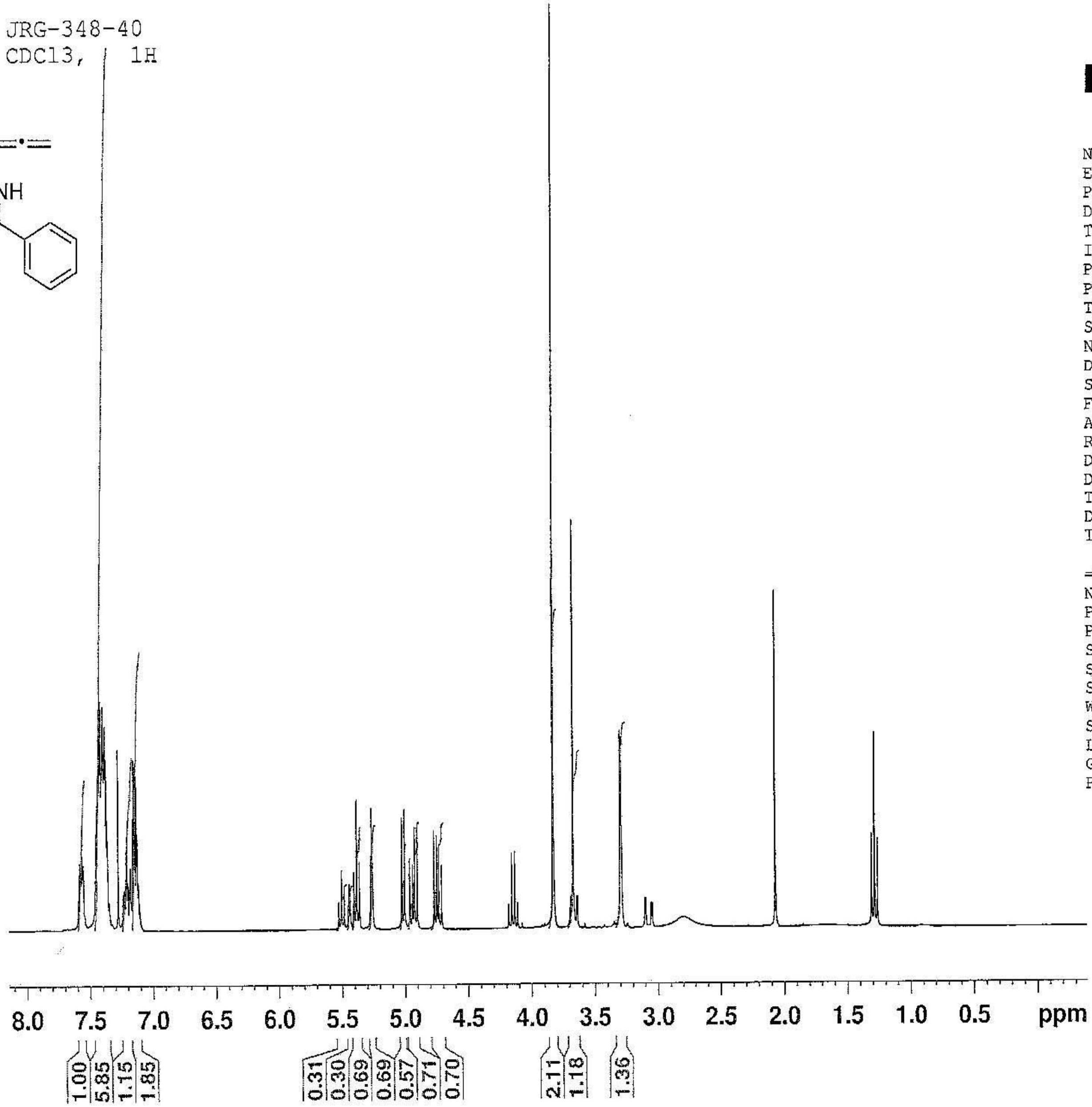

NAME	JRG-348-40
EXPNO	1
PROCNO	1
Date_	20110629
Time	8.41
INSTRUM	spect
PROBHD	5 mm Dual 13C/
PULPROG	zg30
TD	32768
SOLVENT	CDCl3
NS	8
DS	2
SWH	6172.839 Hz
FIDRES	0.188380 Hz
AQ	2.6542580 sec
RG	322.5
DW	81.000 usec
DE	6.50 usec
TE	294.2 K
D1	1.00000000 sec
TD0	1

======== CHANNEL f1 ========

NUC1	1H
P1	6.20 usec
PL1	4.00 dB
SFO1	300.1418535 MHz
SI	32768
SF	300.1400000 MHz
WDW	EM
SSB	0
LB	0.30 Hz
GB	0
PC	1.00

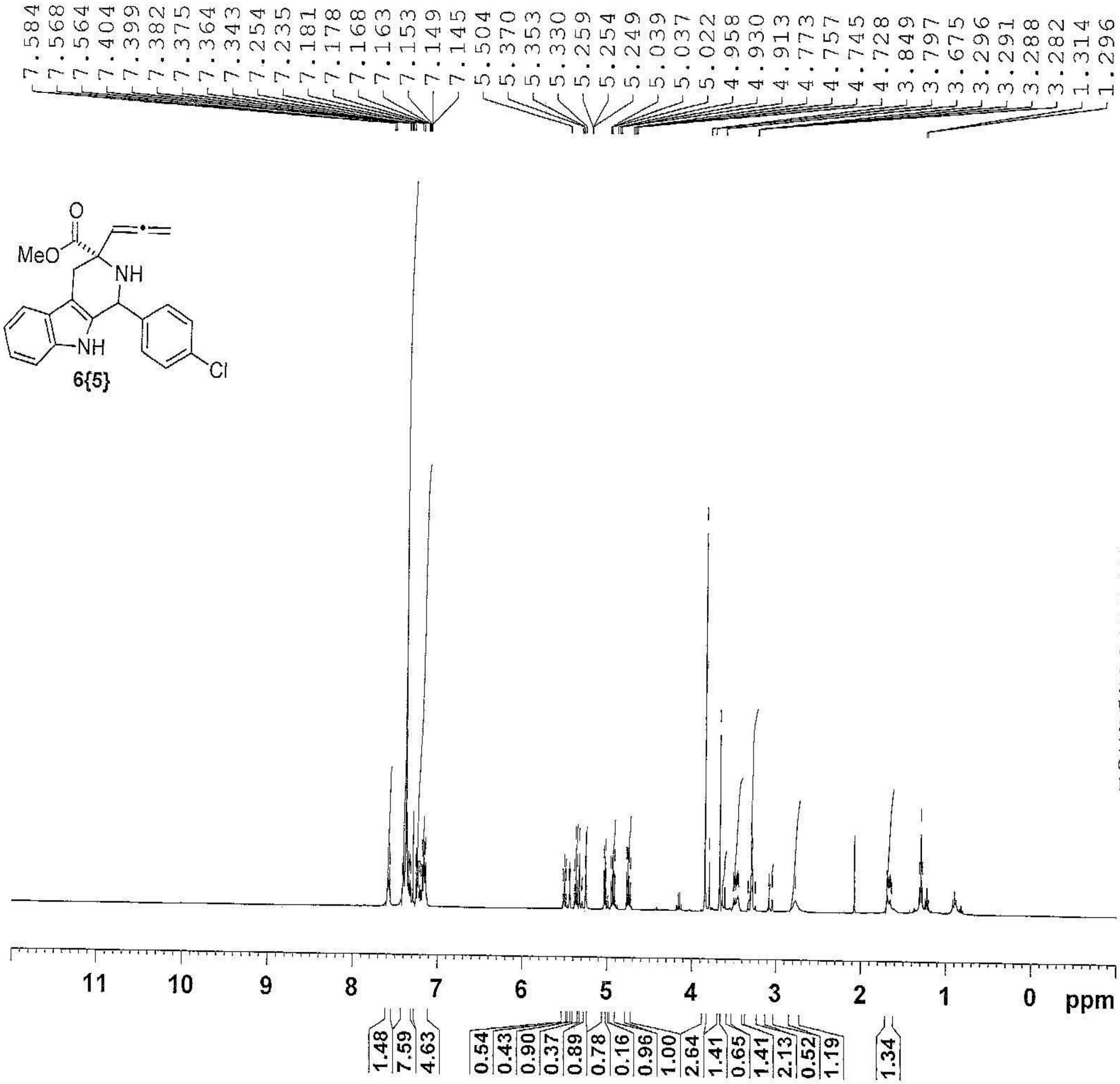

6{5}

NAME	JRG-329-16
EXPNO	1
PROCNO	1
Date_	20110203
Time	16.28
INSTRUM	spect
PROBHD	5 mm PABBO BB-
PULPROG	zg30
TD	65536
SOLVENT	CDCl3
NS	10
DS	2
SWH	8223.685 Hz
FIDRES	0.125483 Hz
AQ	3.9846387 sec
RG	114
DW	60.800 usec
DE	6.50 usec
TE	295.5 K
D1	2.00000000 sec
TD0	1

======== CHANNEL f1 ========

NUC1	1H
P1	14.31 usec
PL1	-1.00 dB
PL1W	11.09959412 W
SFO1	400.2324716 MHz
SI	32768
SF	400.2300000 MHz
WDW	EM
SSB	0
LB	0.30 Hz
GB	0
PC	1.00

JRG-329-87
CDCl3,   1H

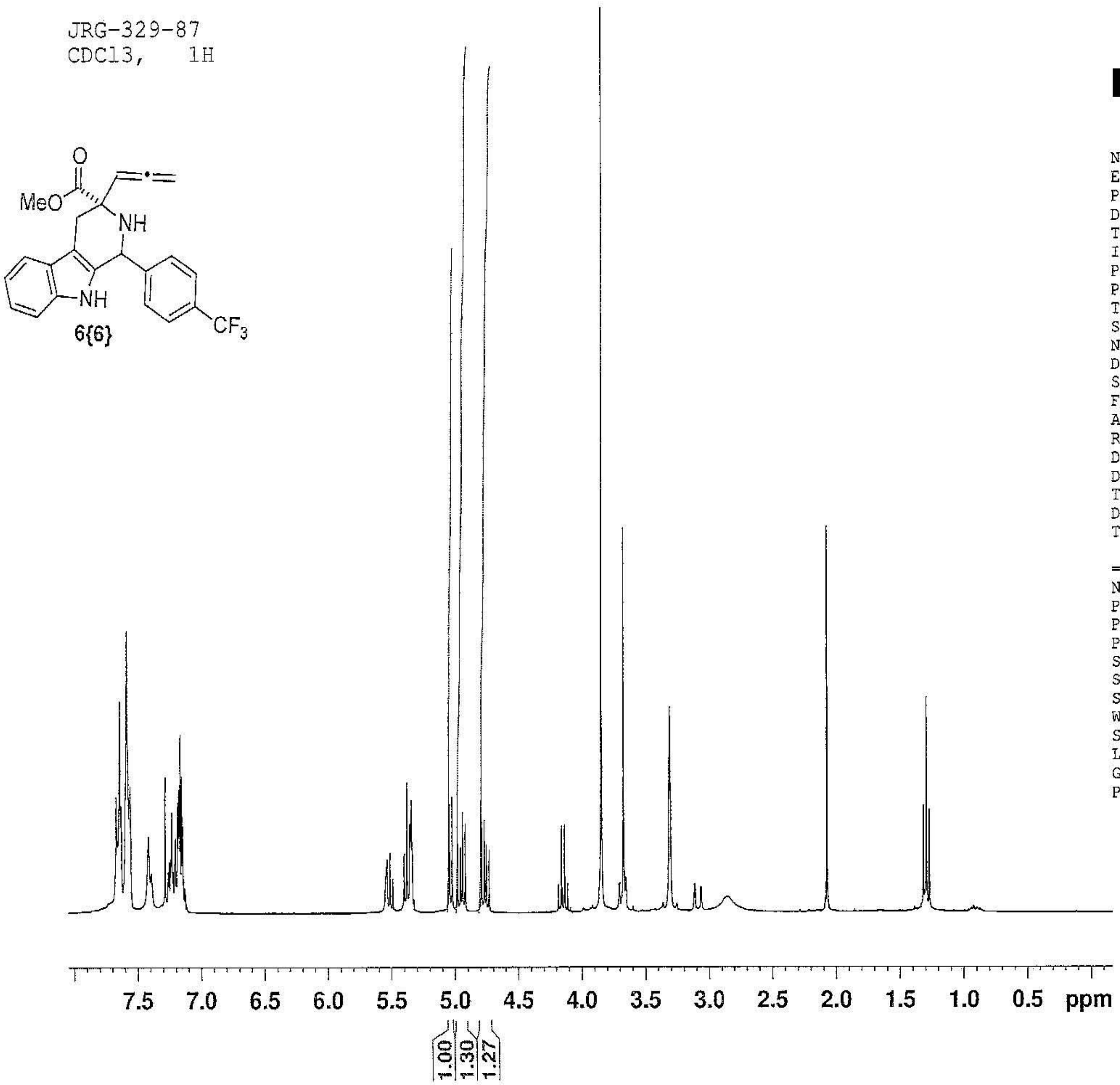

NAME	JRG-329-87	
EXPNO	1	
PROCNO	1	
Date_	20110505	
Time	23.17	
INSTRUM	spect	
PROBHD	5 mm Multinucl	
PULPROG	zg30	
TD	32768	
SOLVENT	CDCl3	
NS	16	
DS	2	
SWH	6188.119	Hz
FIDRES	0.188846	Hz
AQ	2.6477044	sec
RG	114	
DW	80.800	usec
DE	6.50	usec
TE	293.3	K
D1	1.00000000	sec
TD0	1	

======== CHANNEL f1 ========

NUC1	1H	
P1	13.20	usec
PL1	-2.00	dB
PL1W	18.20942116	W
SFO1	300.2318540	MHz
SI	32768	
SF	300.2300000	MHz
WDW	EM	
SSB	0	
LB	0.10	Hz
GB	0	
PC	1.00	

JRG-348-43
CDCl3,   1H

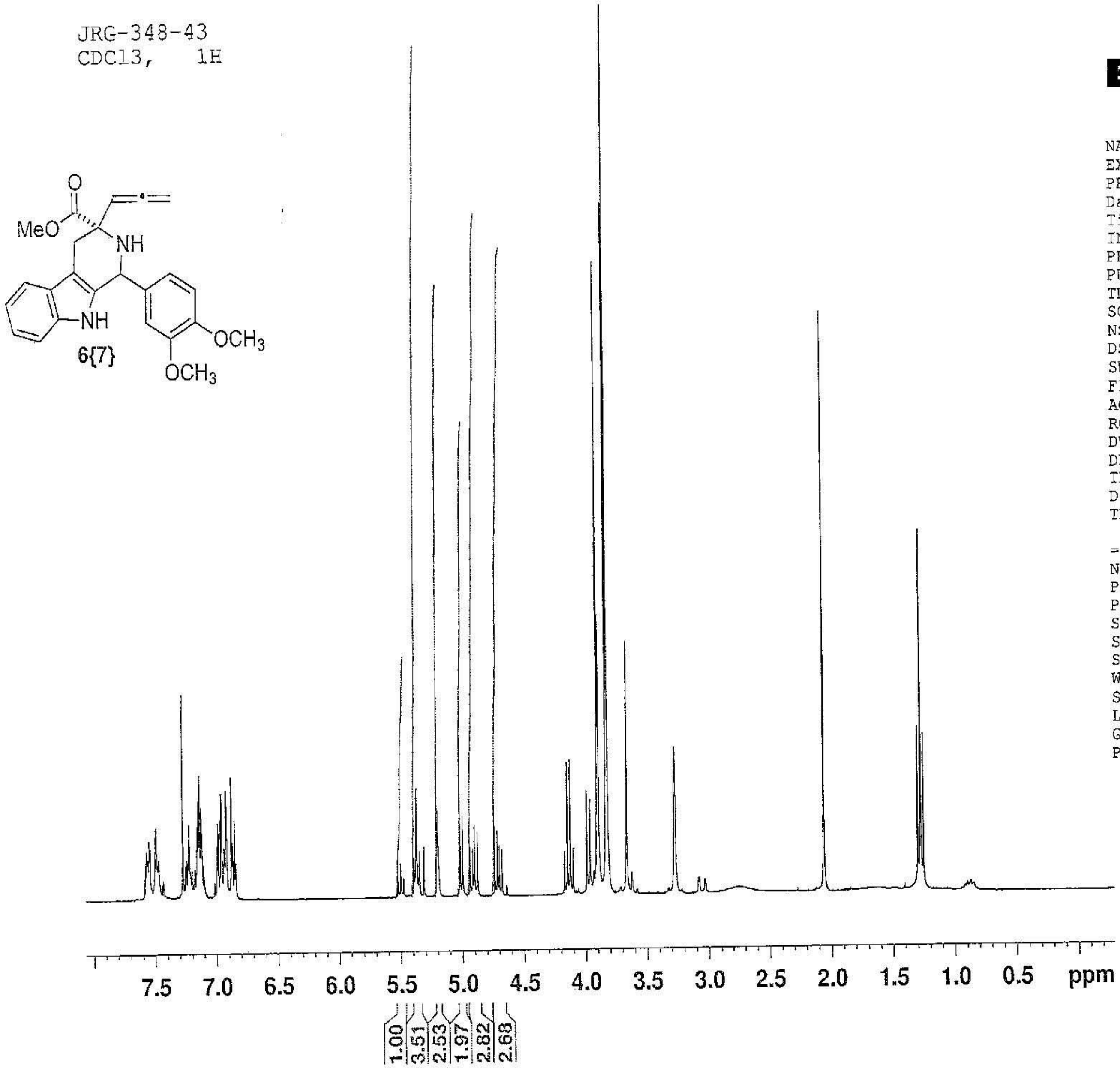

6{7}

NAME	JRG-348-43	
EXPNO	2	
PROCNO	1	
Date_	20110629	
Time	12.23	
INSTRUM	spect	
PROBHD	5 mm Dual 13C/	
PULPROG	zg30	
TD	32768	
SOLVENT	CDCl3	
NS	8	
DS	2	
SWH	6172.839	Hz
FIDRES	0.188380	Hz
AQ	2.6542580	sec
RG	362	
DW	81.000	usec
DE	6.50	usec
TE	295.2	K
D1	1.00000000	sec
TD0	1	

======== CHANNEL f1 ========

NUC1	1H	
P1	6.20	usec
PL1	4.00	dB
SFO1	300.1418535	MHz
SI	32768	
SF	300.1400000	MHz
WDW	EM	
SSB	0	
LB	0.30	Hz
GB	0	
PC	1.00	

JRG-348-41
CDCl13,   1H

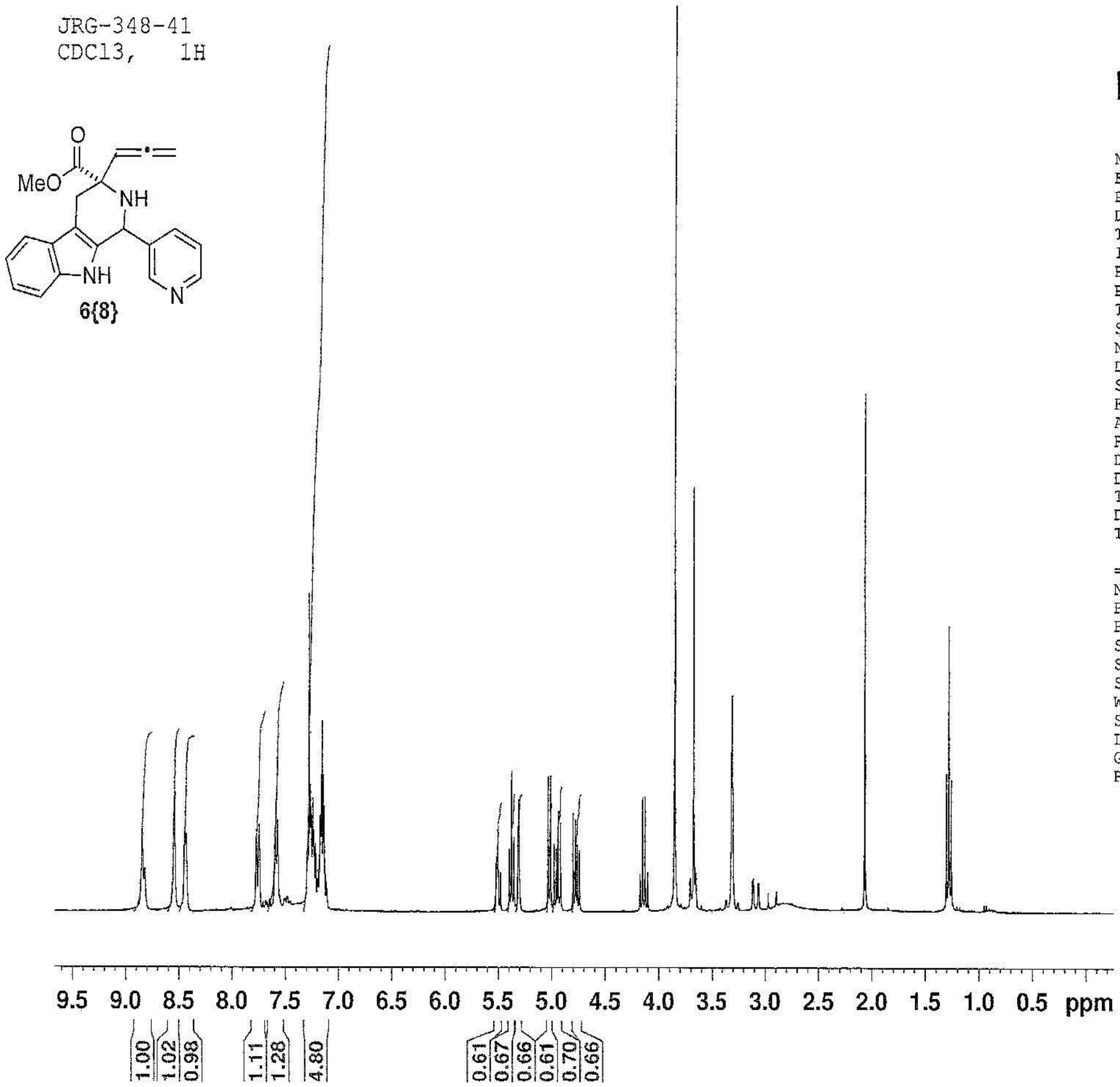

6{8}

NAME	JRG-348-41
EXPNO	1
PROCNO	1
Date_	20110629
Time	8.48
INSTRUM	spect
PROBHD	5 mm Dual 13C/
PULPROG	zg30
TD	32768
SOLVENT	CDCl3
NS	8
DS	2
SWH	6172.839 Hz
FIDRES	0.188380 Hz
AQ	2.6542580 sec
RG	362
DW	81.000 usec
DE	6.50 usec
TE	294.2 K
D1	1.00000000 sec
TD0	1

======== CHANNEL f1 ========

NUC1	1H
P1	6.20 usec
PL1	4.00 dB
SFO1	300.1418535 MHz
SI	32768
SF	300.1400000 MHz
WDW	EM
SSB	0
LB	0.30 Hz
GB	0
PC	1.00

JRG-329-89
CDCl3, 1H

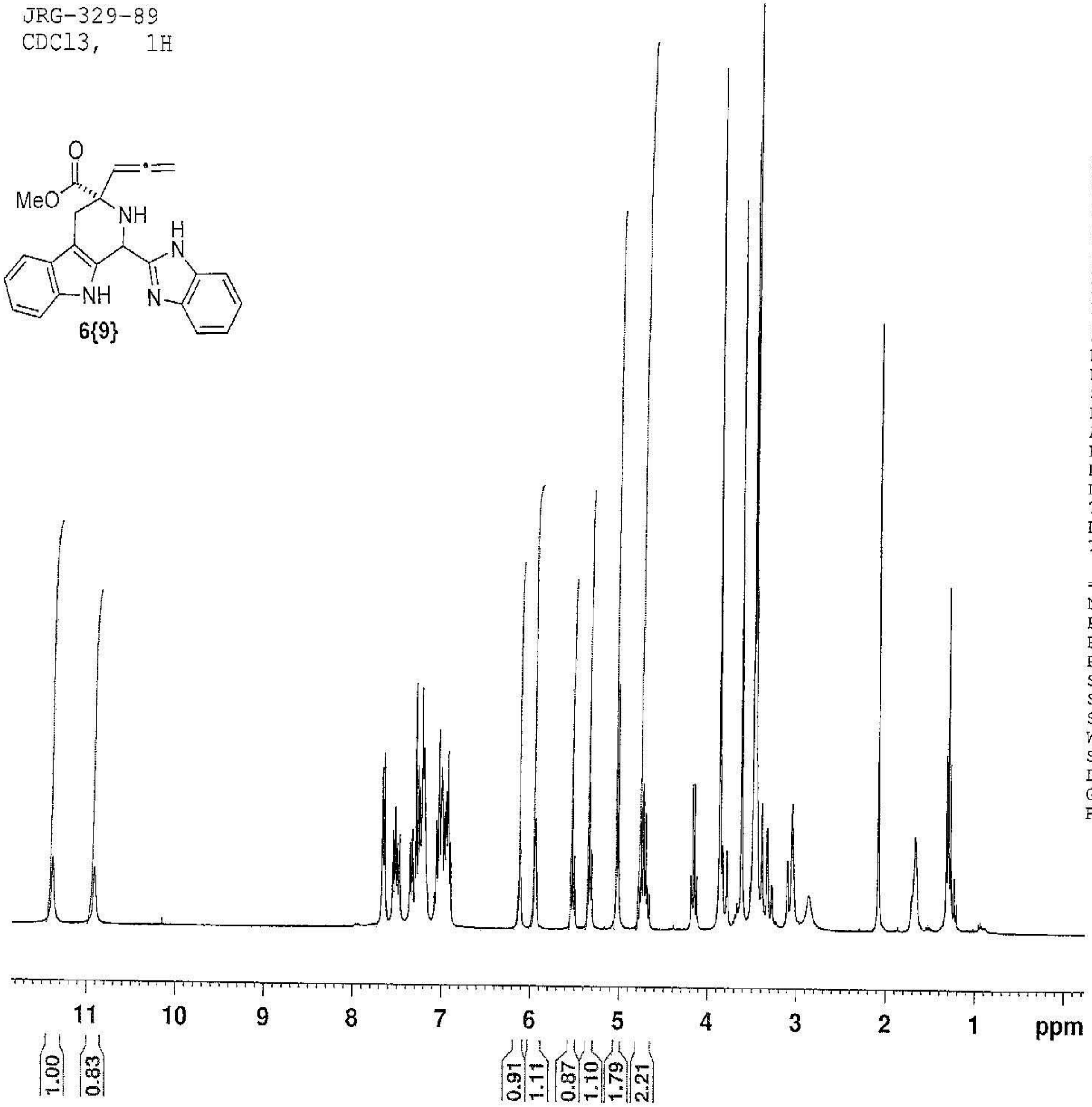

6{9}

BRUKER

NAME	JRG-329-89	
EXPNO	1	
PROCNO	1	
Date_	20110505	
Time	23.30	
INSTRUM	spect	
PROBHD	5 mm Multinucl	
PULPROG	zg30	
TD	32768	
SOLVENT	CDCl3	
NS	16	
DS	2	
SWH	6188.119	Hz
FIDRES	0.188846	Hz
AQ	2.6477044	sec
RG	64	
DW	80.800	usec
DE	6.50	usec
TE	293.3	K
D1	1.00000000	sec
TD0	1	

======== CHANNEL f1 ========

NUC1	1H	
P1	13.20	usec
PL1	-2.00	dB
PL1W	18.20942116	W
SFO1	300.2318540	MHz
SI	32768	
SF	300.2300000	MHz
WDW	EM	
SSB	0	
LB	0.10	Hz
GB	0	
PC	1.00	

JRG-329-90b
CDCl3,   1H

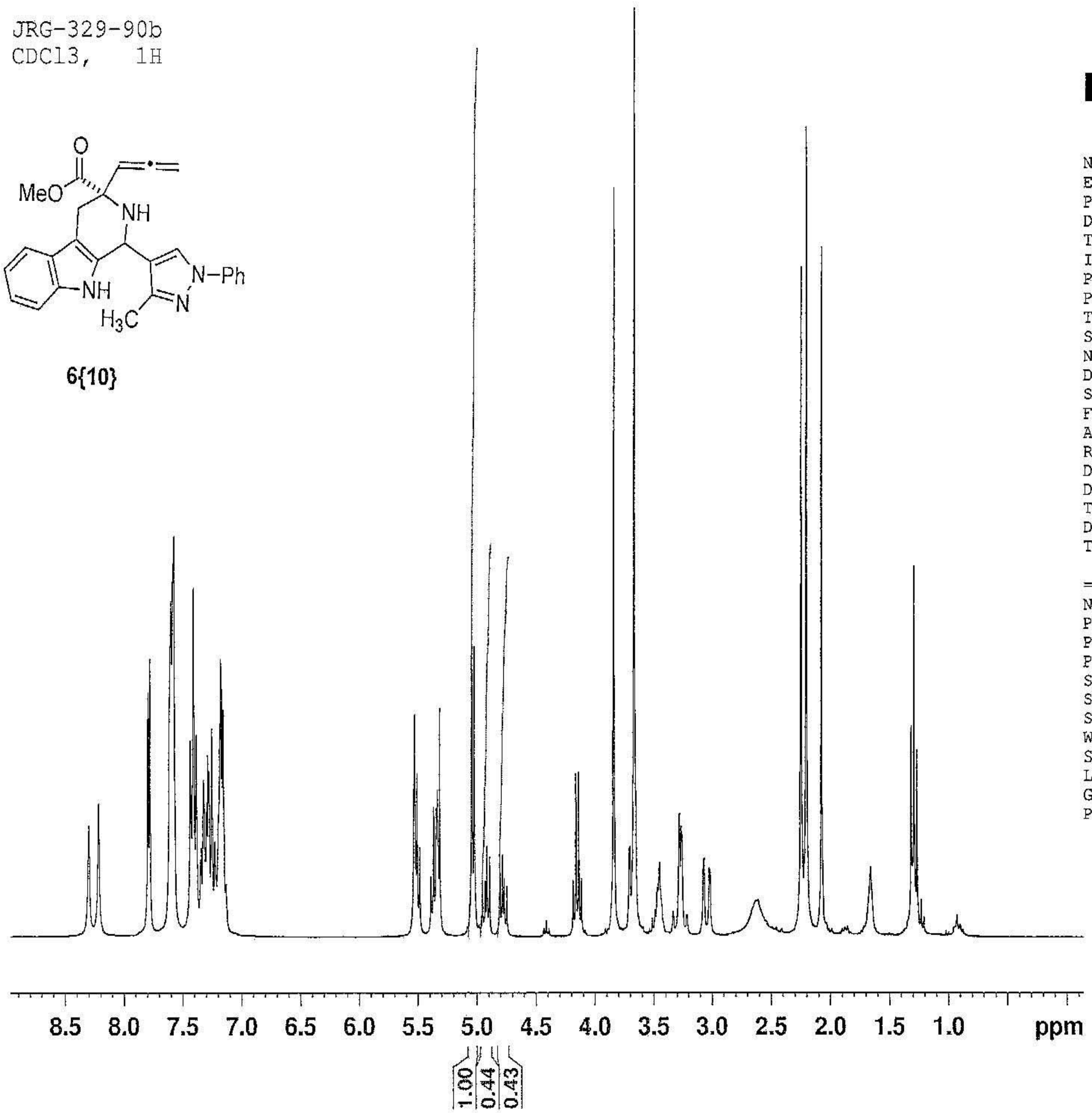

6{10}

BRUKER

NAME	JRG-329-90b
EXPNO	1
PROCNO	1
Date_	20110505
Time	23.10
INSTRUM	spect
PROBHD	5 mm Multinucl
PULPROG	zg30
TD	32768
SOLVENT	CDCl3
NS	16
DS	2
SWH	6188.119 Hz
FIDRES	0.188846 Hz
AQ	2.6477044 sec
RG	50.8
DW	80.800 usec
DE	6.50 usec
TE	293.3 K
D1	1.00000000 sec
TD0	1

======== CHANNEL f1 ========

NUC1	1H
P1	13.20 usec
PL1	-2.00 dB
PL1W	18.20942116 W
SFO1	300.2318540 MHz
SI	32768
SF	300.2300000 MHz
WDW	EM
SSB	0
LB	0.10 Hz
GB	0
PC	1.00

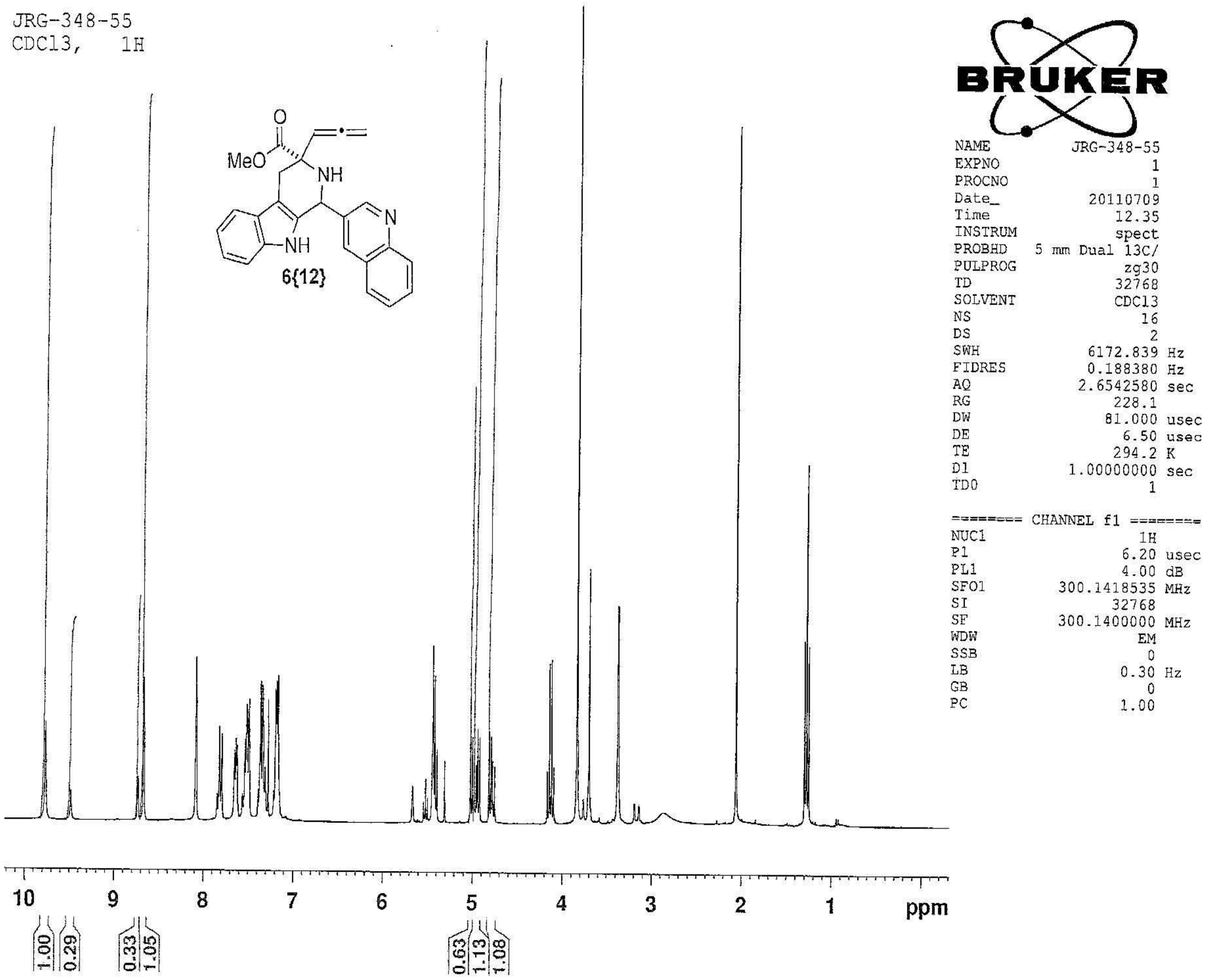
JRG-348-55
CDCl3, 1H
MeO
O
NH
N
NH
6{12}
BRUKER
NAME JRG-348-55
EXPNO 1
PROCNO 1
Date_ 20110709
Time 12.35
INSTRUM spect
PROBHD 5 mm Dual 13C/
PULPROG zg30
TD 32768
SOLVENT CDCl3
NS 16
DS 2
SWH 6172.839 Hz
FIDRES 0.188380 Hz
AQ 2.6542580 sec
RG 228.1
DW 81.000 usec
DE 6.50 usec
TE 294.2 K
D1 1.00000000 sec
TD0 1
======== CHANNEL f1 ========
NUC1 1H
P1 6.20 usec
PL1 4.00 dB
SFO1 300.1418535 MHz
SI 32768
SF 300.1400000 MHz
WDW EM
SSB 0
LB 0.30 Hz
GB 0
PC 1.00
10
9
8
7
6
5
4
3
2
1
ppm
1.00
0.29
0.33
1.05
0.63
1.13
1.08

JRG-348-56
CDCl3, 1H

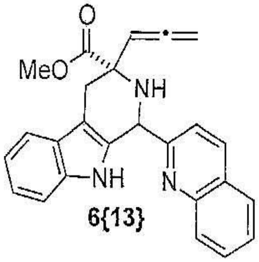

6{13}

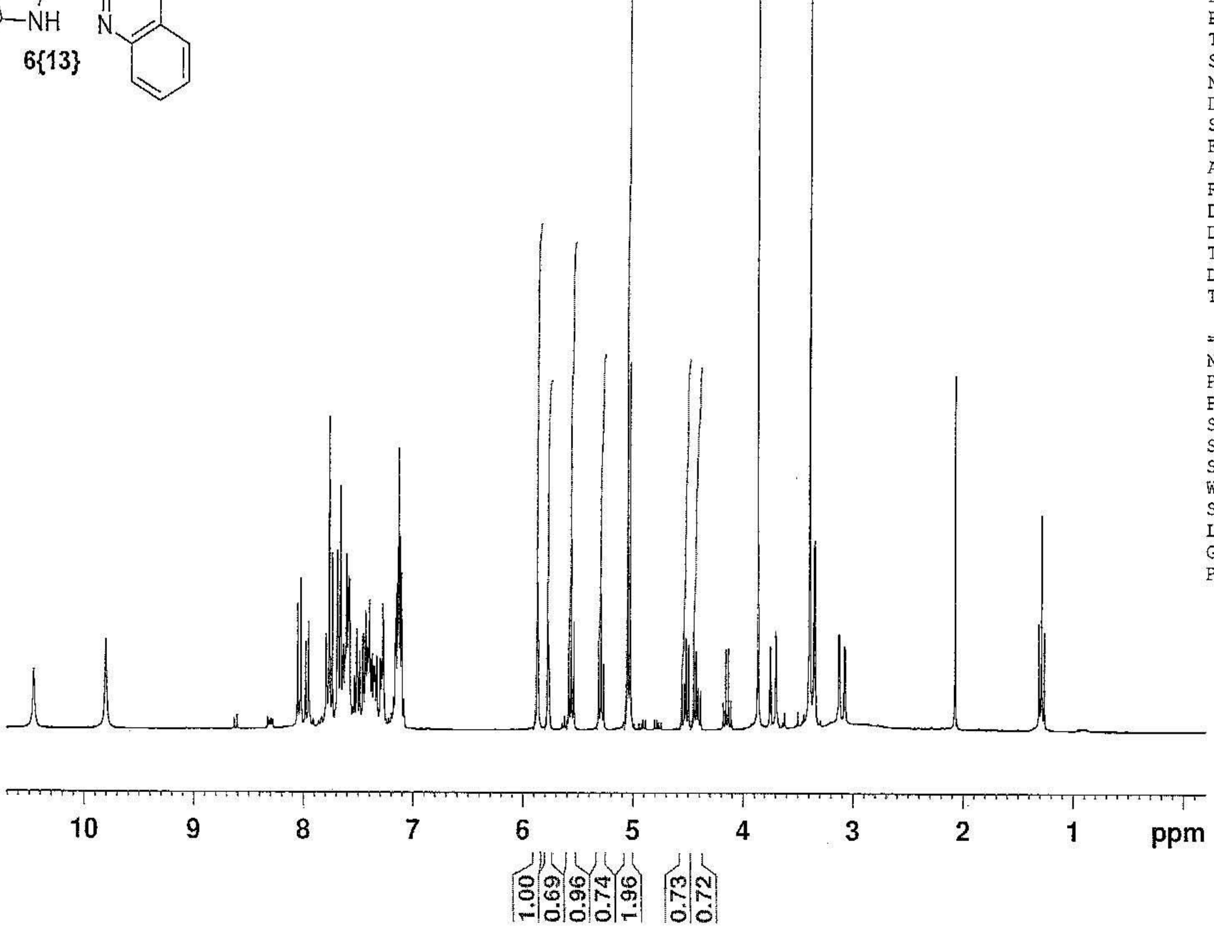

BRUKER

NAME	JRG-348-56
EXPNO	1
PROCNO	1
Date_	20110709
Time	12.42
INSTRUM	spect
PROBHD	5 mm Dual 13C/
PULPROG	zg30
TD	32768
SOLVENT	CDCl3
NS	16
DS	2
SWH	6172.839 Hz
FIDRES	0.188380 Hz
AQ	2.6542580 sec
RG	256
DW	81.000 usec
DE	6.50 usec
TE	294.2 K
D1	1.00000000 sec
TD0	1

======== CHANNEL f1 ========

NUC1	1H
P1	6.20 usec
PL1	4.00 dB
SFO1	300.1418535 MHz
SI	32768
SF	300.1400000 MHz
WDW	EM
SSB	0
LB	0.30 Hz
GB	0
PC	1.00

JRG-348-57
CDCl3, 1H

6{15}

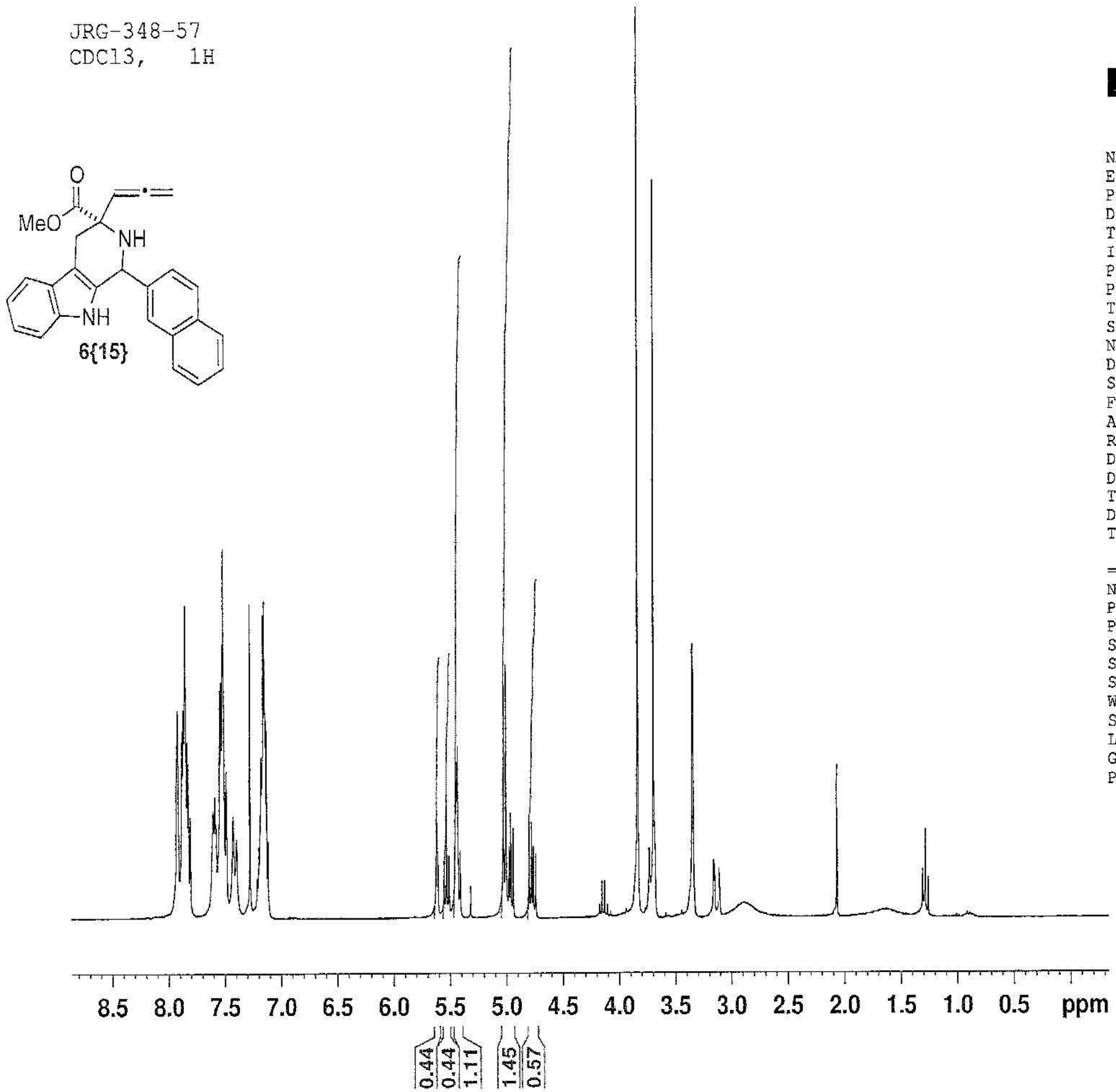

BRUKER

NAME	JRG-348-57
EXPNO	1
PROCNO	1
Date_	20110709
Time	12.53
INSTRUM	spect
PROBHD	5 mm Dual 13C/
PULPROG	zg30
TD	32768
SOLVENT	CDCl3
NS	16
DS	2
SWH	6172.839 Hz
FIDRES	0.188380 Hz
AQ	2.6542580 sec
RG	456.1
DW	81.000 usec
DE	6.50 usec
TE	294.2 K
D1	1.00000000 sec
TD0	1

======== CHANNEL f1 ========

NUC1	1H
P1	6.20 usec
PL1	4.00 dB
SFO1	300.1418535 MHz
SI	32768
SF	300.1400000 MHz
WDW	EM
SSB	0
LB	0.30 Hz
GB	0
PC	1.00

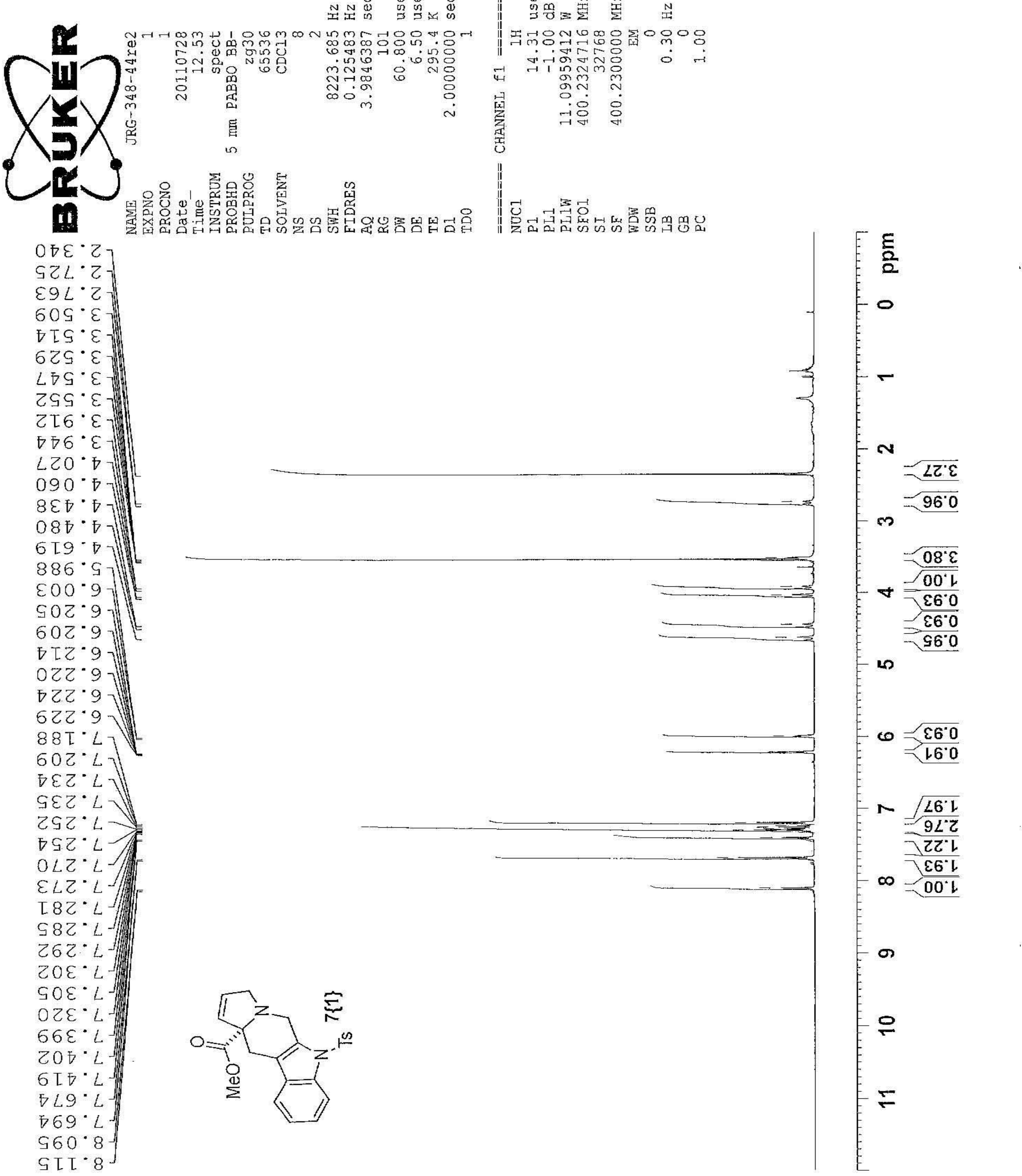
2.340
2.725
2.763
3.509
3.514
3.529
3.547
3.552
3.912
3.944
4.027
4.060
4.438
4.480
4.619
5.988
6.003
6.205
6.209
6.214
6.220
6.224
6.229
7.188
7.209
7.234
7.235
7.252
7.254
7.270
7.273
7.281
7.285
7.292
7.302
7.305
7.320
7.399
7.402
7.419
7.674
7.694
8.095
8.115
NAME JRG-348-44re2
EXPNO 1
PROCNO 1
Date_ 20110728
Time_ 12.53
INSTRUM spect
PROBHD 5 mm PABBO BB-
PULPROG zg30
TD 65536
SOLVENT CDCl3
NS 8
DS 2
SWH 8223.685 Hz
FIDRES 0.125483 Hz
AQ 3.9846387 sec
RG 101
DW 60.800 usec
DE 6.50 usec
TE 295.4 K
D1 2.00000000 sec
TD0 1
======== CHANNEL f1 ========
NUC1 1H
P1 14.31 usec
PL1 -1.00 dB
PL1W 11.09959412 W
SFO1 400.2324716 MHz
SI 32768
SF 400.2300000 MHz
WDW EM
SSB 0
LB 0.30 Hz
GB 0
PC 1.00
MeO
O
N
N
Ts
7{1}
3.27
0.96
3.80
1.00
0.93
0.93
0.95
0.93
0.91
1.97
2.76
1.22
1.93
1.00
ppm
0
1
2
3
4
5
6
7
8
9
10
11

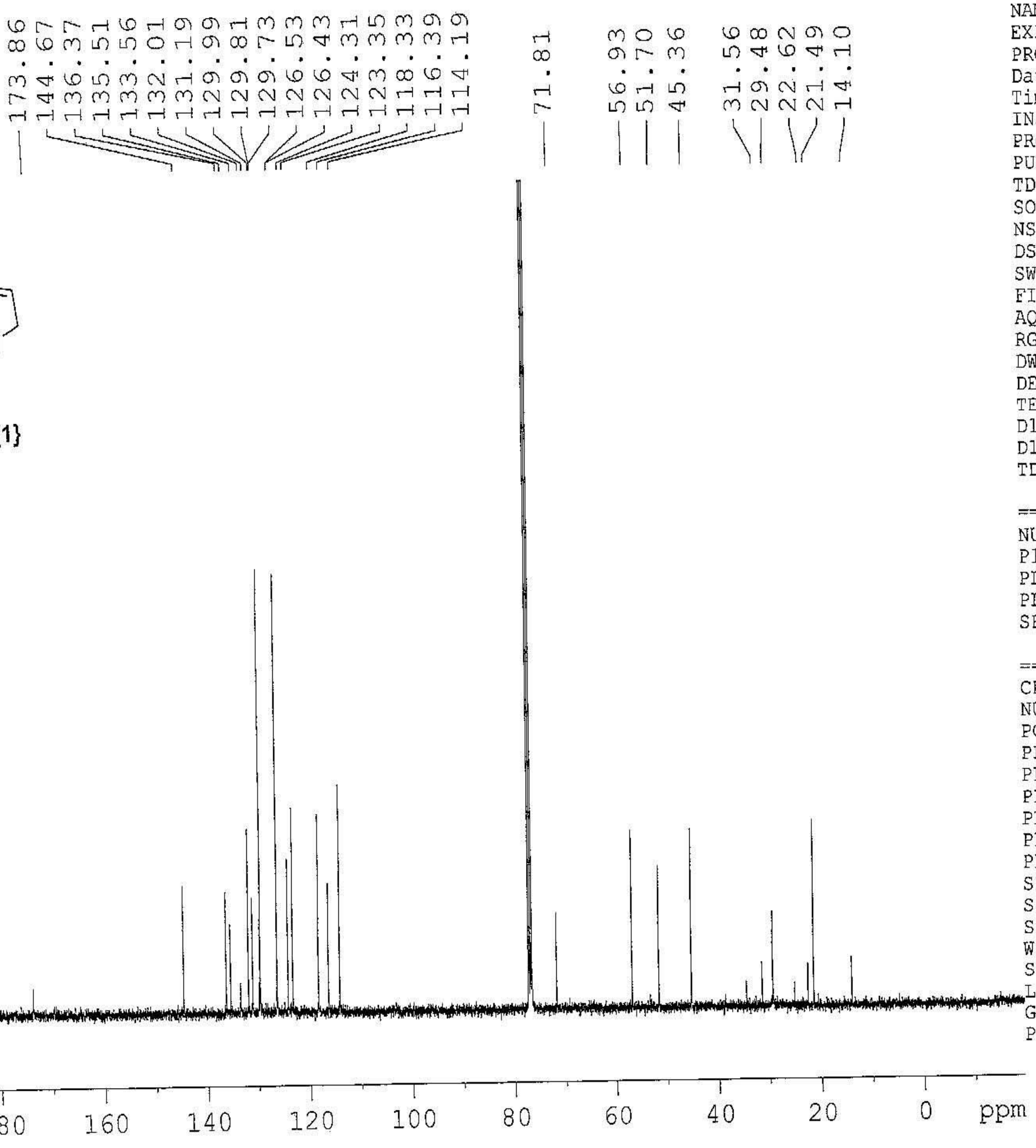

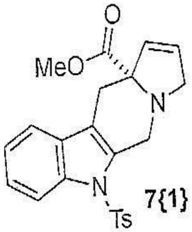

7{1}

BRUKER

NAME	JRG-348-44re2
EXPNO	2
PROCNO	1
Date_	20110728
Time	20.16
INSTRUM	spect
PROBHD	5 mm PABBO BB-
PULPROG	zgpg30
TD	65536
SOLVENT	CDCl3
NS	715
DS	4
SWH	24038.461 Hz
FIDRES	0.366798 Hz
AQ	1.3631988 sec
RG	203
DW	20.800 usec
DE	6.50 usec
TE	295.8 K
D1	3.00000000 sec
D11	0.03000000 sec
TD0	1

======== CHANNEL f1 ========

NUC1	13C
P1	10.00 usec
PL1	-1.59 dB
PL1W	51.07626343 W
SFO1	100.6479773 MHz

======== CHANNEL f2 ========

CPDPRG2	waltz16
NUC2	1H
PCPD2	75.00 usec
PL2	-1.00 dB
PL12	13.39 dB
PL13	20.00 dB
PL2W	11.09959412 W
PL12W	0.40393090 W
PL13W	0.08816721 W
SFO2	400.2316009 MHz
SI	32768
SF	100.6379191 MHz
WDW	EM
SSB	0
LB	1.00 Hz
GB	0
PC	1.40

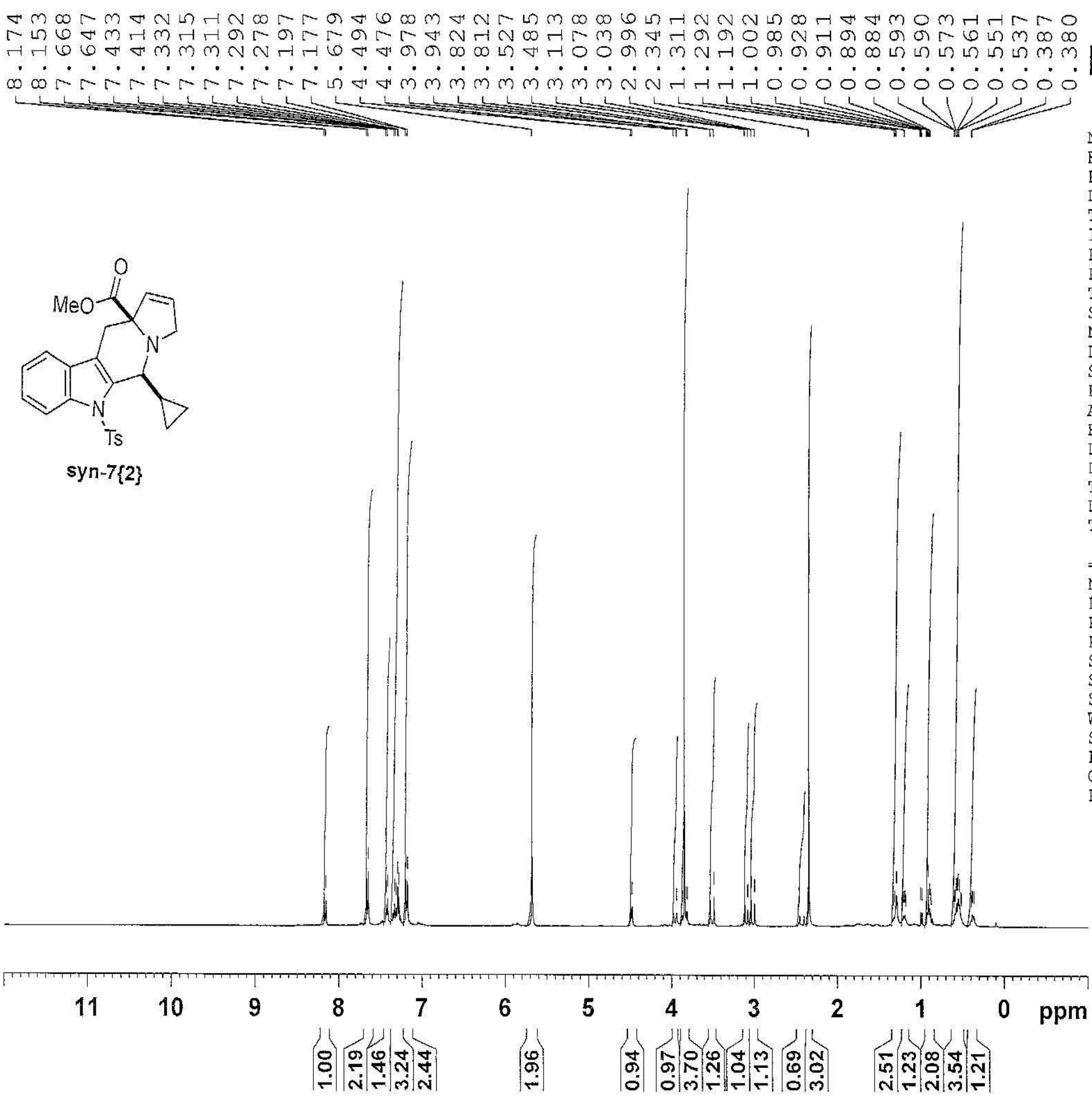

syn-7{2}

BRUKER

NAME	JRG-348-45bre
EXPNO	1
PROCNO	1
Date_	20110728
Time	11.12
INSTRUM	spect
PROBHD	5 mm PABBO BB-
PULPROG	zg30
TD	65536
SOLVENT	CDCl3
NS	3
DS	2
SWH	8223.685 Hz
FIDRES	0.125483 Hz
AQ	3.9846387 sec
RG	80.6
DW	60.800 usec
DE	6.50 usec
TE	294.9 K
D1	2.00000000 sec
TD0	1

======== CHANNEL f1 ========

NUC1	1H
P1	14.31 usec
PL1	-1.00 dB
PL1W	11.09959412 W
SFO1	400.2324716 MHz
SI	32768
SF	400.2300000 MHz
WDW	EM
SSB	0
LB	0.30 Hz
GB	0
PC	1.00

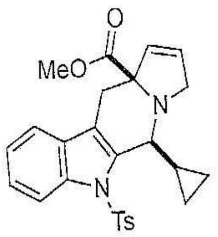

syn-7{2}

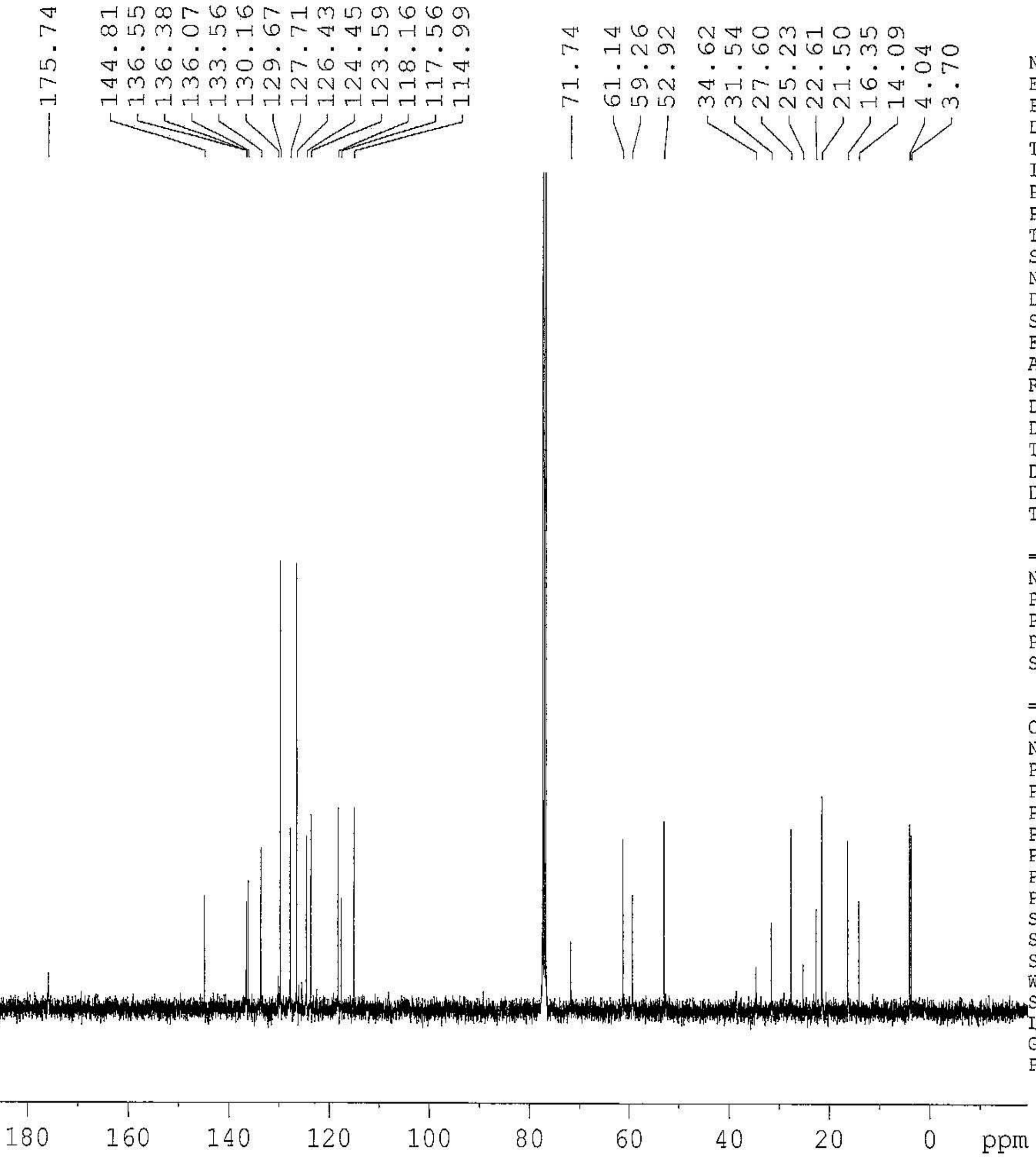

BRUKER

NAME	JRG-348-45bre
EXPNO	2
PROCNO	1
Date_	20110728
Time_	11.26
INSTRUM	spect
PROBHD	5 mm PABBO BB-
PULPROG	zgpg30
TD	65536
SOLVENT	CDCl3
NS	274
DS	4
SWH	24038.461 Hz
FIDRES	0.366798 Hz
AQ	1.3631988 sec
RG	203
DW	20.800 usec
DE	6.50 usec
TE	295.6 K
D1	3.00000000 sec
D11	0.03000000 sec
TD0	1

======== CHANNEL f1 ========

NUC1	13C
P1	10.00 usec
PL1	-1.59 dB
PL1W	51.07626343 W
SFO1	100.6479773 MHz

======== CHANNEL f2 ========

CPDPRG2	waltz16
NUC2	1H
PCPD2	75.00 usec
PL2	-1.00 dB
PL12	13.39 dB
PL13	20.00 dB
PL2W	11.09959412 W
PL12W	0.40393090 W
PL13W	0.08816721 W
SFO2	400.2316009 MHz
SI	32768
SF	100.6379202 MHz
WDW	EM
SSB	0
LB	1.00 Hz
GB	0
PC	1.40

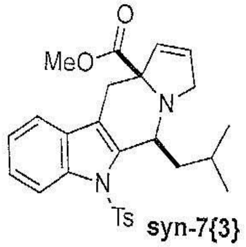

syn-7{3}

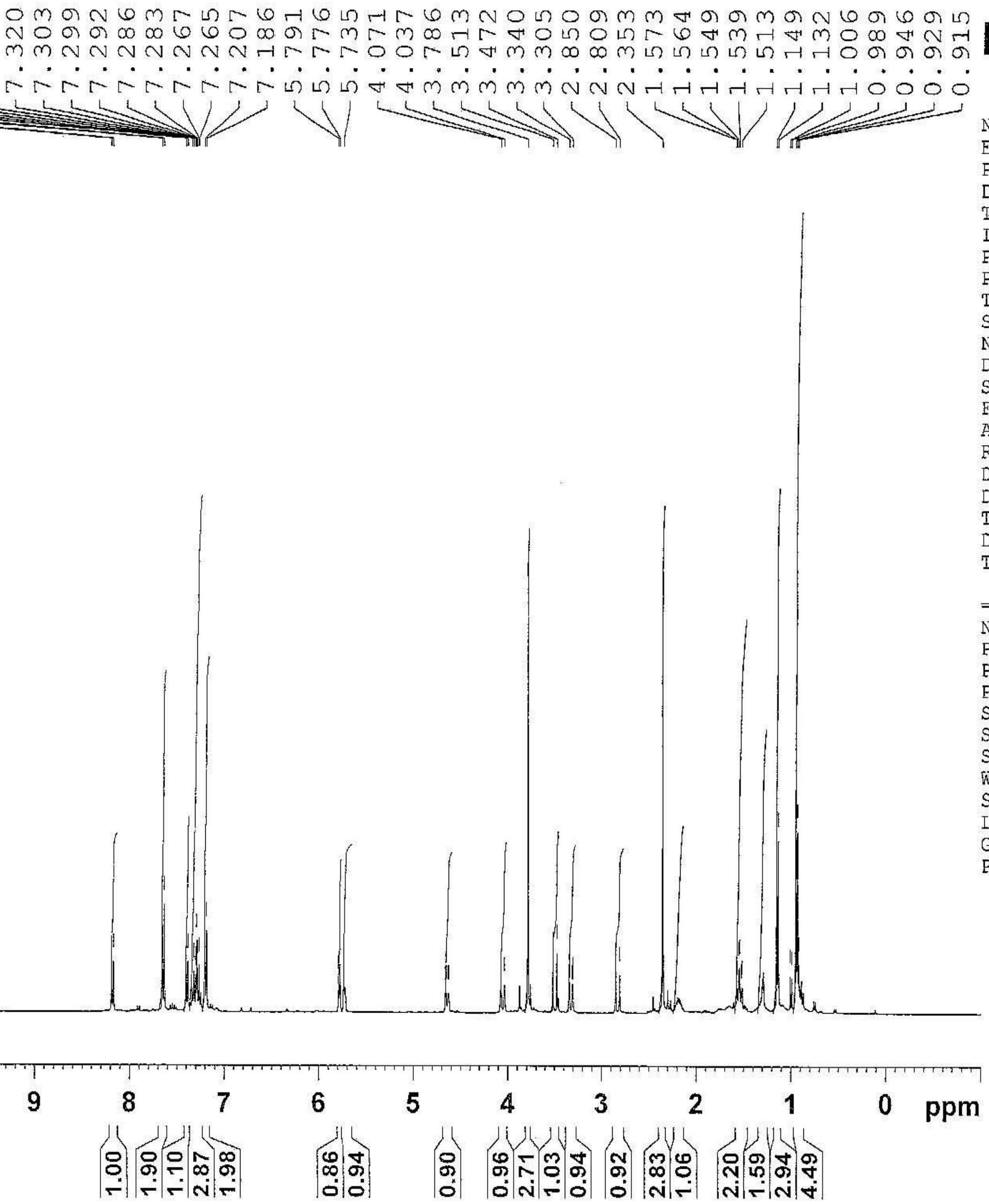

BRUKER

NAME	JRG-348-46bre	
EXPNO	1	
PROCNO	1	
Date_	20110728	
Time_	12.10	
INSTRUM	spect	
PROBHD	5 mm PABBO BB-	
PULPROG	zg30	
TD	65536	
SOLVENT	CDCl3	
NS	4	
DS	2	
SWH	8223.685	Hz
FIDRES	0.125483	Hz
AQ	3.9846387	sec
RG	80.6	
DW	60.800	usec
DE	6.50	usec
TE	295.3	K
D1	2.00000000	sec
TD0	1	
======== CHANNEL f1 ========		
NUC1	1H	
P1	14.31	usec
PL1	-1.00	dB
PL1W	11.09959412	W
SFO1	400.2324716	MHz
SI	32768	
SF	400.2300000	MHz
WDW	EM	
SSB	0	
LB	0.30	Hz
GB	0	
PC	1.00	

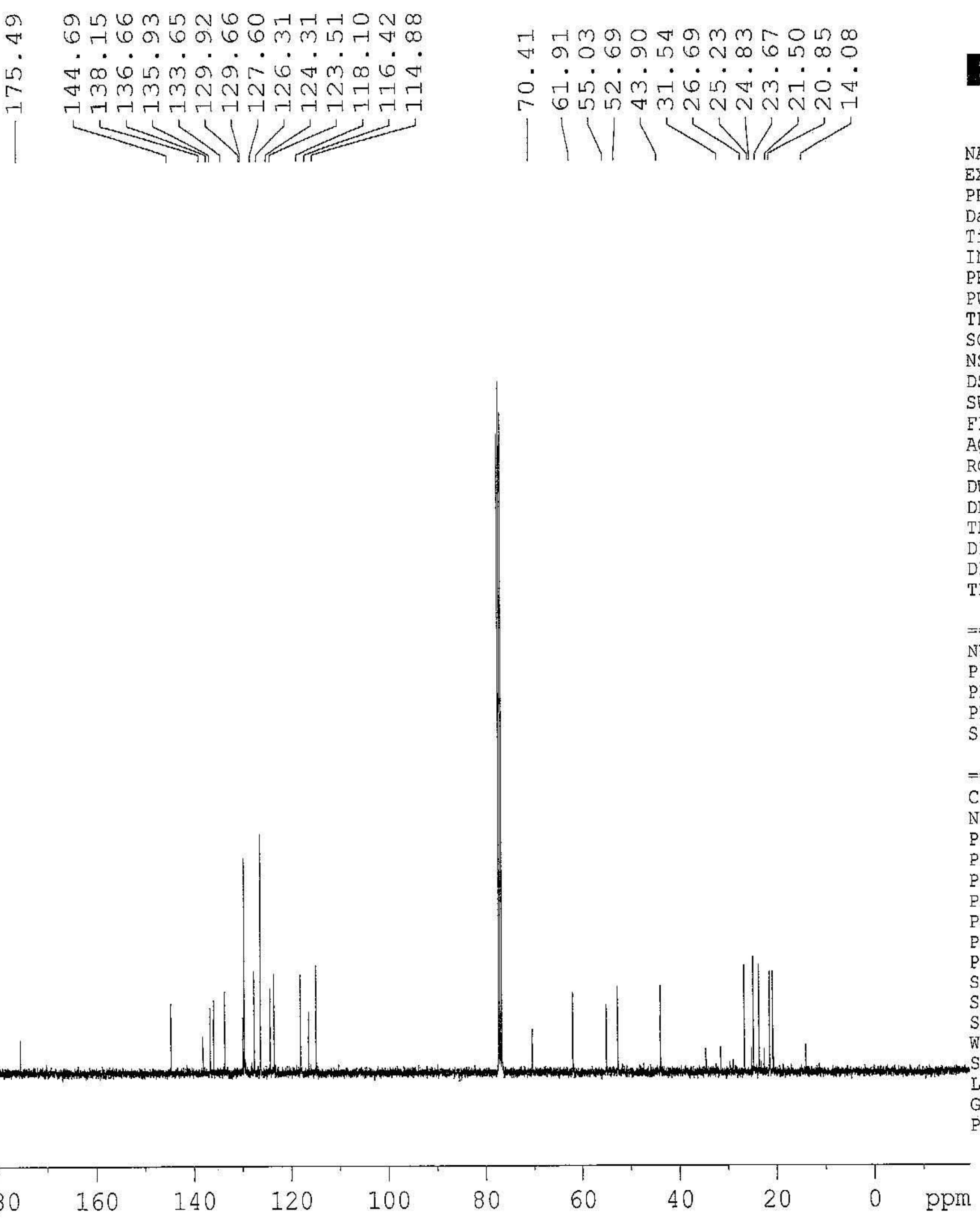

NAME	JRG-348-46bre
EXPNO	2
PROCNO	1
Date_	20110728
Time	12.29
INSTRUM	spect
PROBHD	5 mm PABBO BB-
PULPROG	zgpg30
TD	65536
SOLVENT	CDCl3
NS	239
DS	4
SWH	24038.461 Hz
FIDRES	0.366798 Hz
AQ	1.3631988 sec
RG	203
DW	20.800 usec
DE	6.50 usec
TE	295.8 K
D1	3.00000000 sec
D11	0.03000000 sec
TD0	1

======== CHANNEL f1 ========

NUC1	13C
P1	10.00 usec
PL1	-1.59 dB
PL1W	51.07626343 W
SFO1	100.6479773 MHz

======== CHANNEL f2 ========

CPDPRG2	waltz16
NUC2	1H
PCPD2	75.00 usec
PL2	-1.00 dB
PL12	13.39 dB
PL13	20.00 dB
PL2W	11.09959412 W
PL12W	0.40393090 W
PL13W	0.08816721 W
SFO2	400.2316009 MHz
SI	32768
SF	100.6379208 MHz
WDW	EM
SSB	0
LB	1.00 Hz
GB	0
PC	1.40

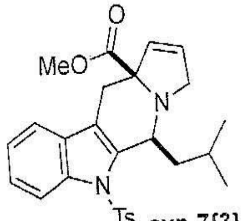

syn-7{3}

A

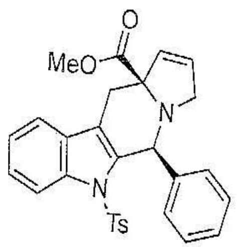

syn-7{4}

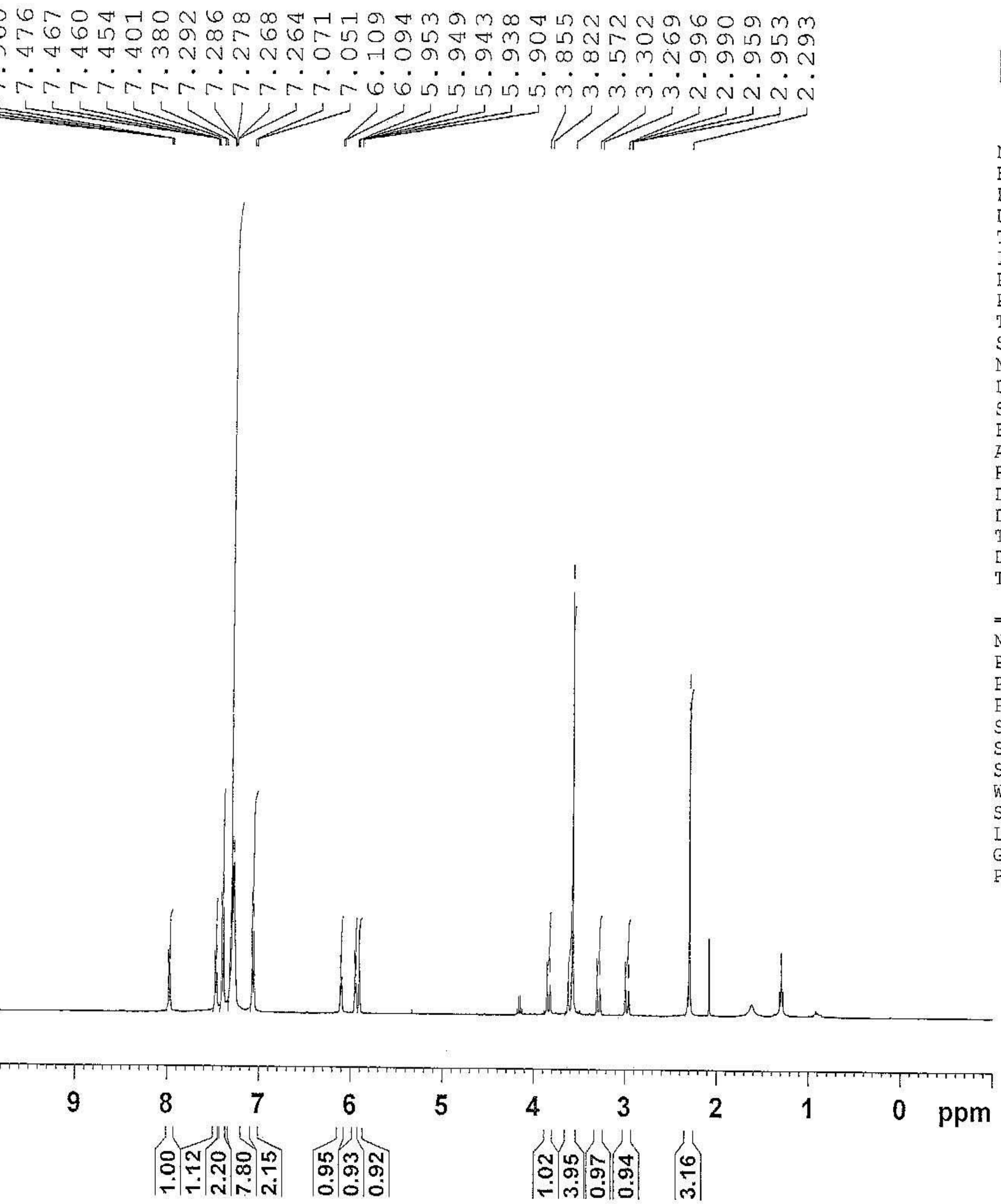

NAME	JRG-348-51re
EXPNO	1
PROCNO	1
Date_	20110727
Time	0.21
INSTRUM	spect
PROBHD	5 mm PABBO BB-
PULPROG	zg30
TD	65536
SOLVENT	CDCl3
NS	10
DS	2
SWH	8223.685 Hz
FIDRES	0.125483 Hz
AQ	3.9846387 sec
RG	144
DW	60.800 usec
DE	6.50 usec
TE	295.0 K
D1	2.00000000 sec
TD0	1

======== CHANNEL f1 ========

NUC1	1H
P1	14.31 usec
PL1	-1.00 dB
PL1W	11.09959412 W
SFO1	400.2324716 MHz
SI	32768
SF	400.2300000 MHz
WDW	EM
SSB	0
LB	0.30 Hz
GB	0
PC	1.00

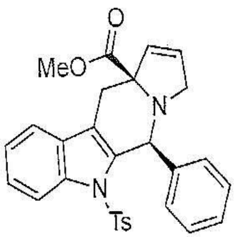

syn-7{4}

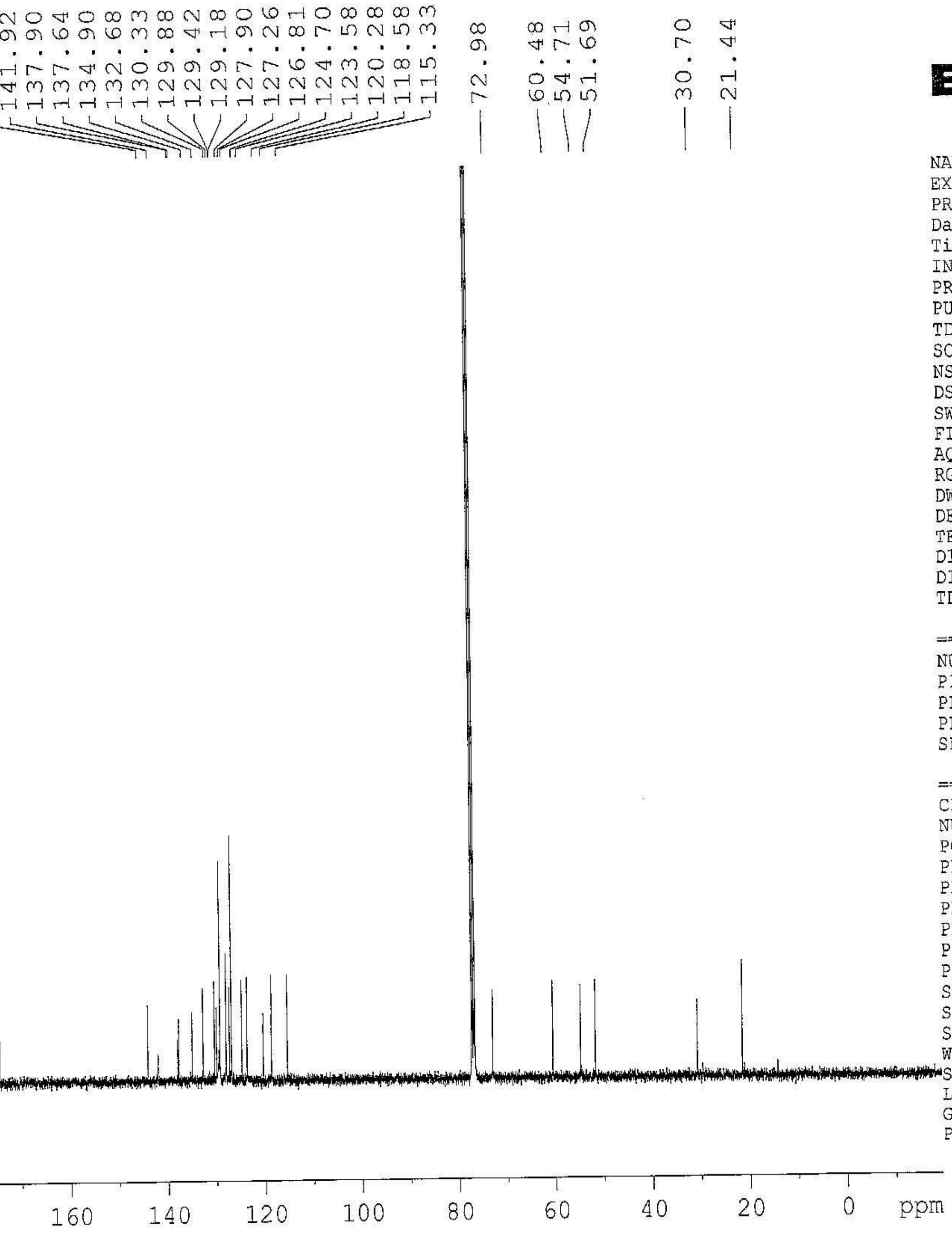

BRUKER

NAME	JRG-348-51re
EXPNO	2
PROCNO	1
Date_	20110727
Time_	1.40
INSTRUM	spect
PROBHD	5 mm PABBO BB-
PULPROG	zgpg30
TD	65536
SOLVENT	CDCl3
NS	1024
DS	4
SWH	24038.461 Hz
FIDRES	0.366798 Hz
AQ	1.3631988 sec
RG	203
DW	20.800 usec
DE	6.50 usec
TE	295.4 K
D1	3.00000000 sec
D11	0.03000000 sec
TD0	1

======== CHANNEL f1 ========

NUC1	13C
P1	10.00 usec
PL1	-1.59 dB
PL1W	51.07626343 W
SFO1	100.6479773 MHz

======== CHANNEL f2 ========

CPDPRG2	waltz16
NUC2	1H
PCPD2	75.00 usec
PL2	-1.00 dB
PL12	13.39 dB
PL13	20.00 dB
PL2W	11.09959412 W
PL12W	0.40393090 W
PL13W	0.08816721 W
SFO2	400.2316009 MHz
SI	32768
SF	100.6379196 MHz
WDW	EM
SSB	0
LB	1.00 Hz
GB	0
PC	1.40

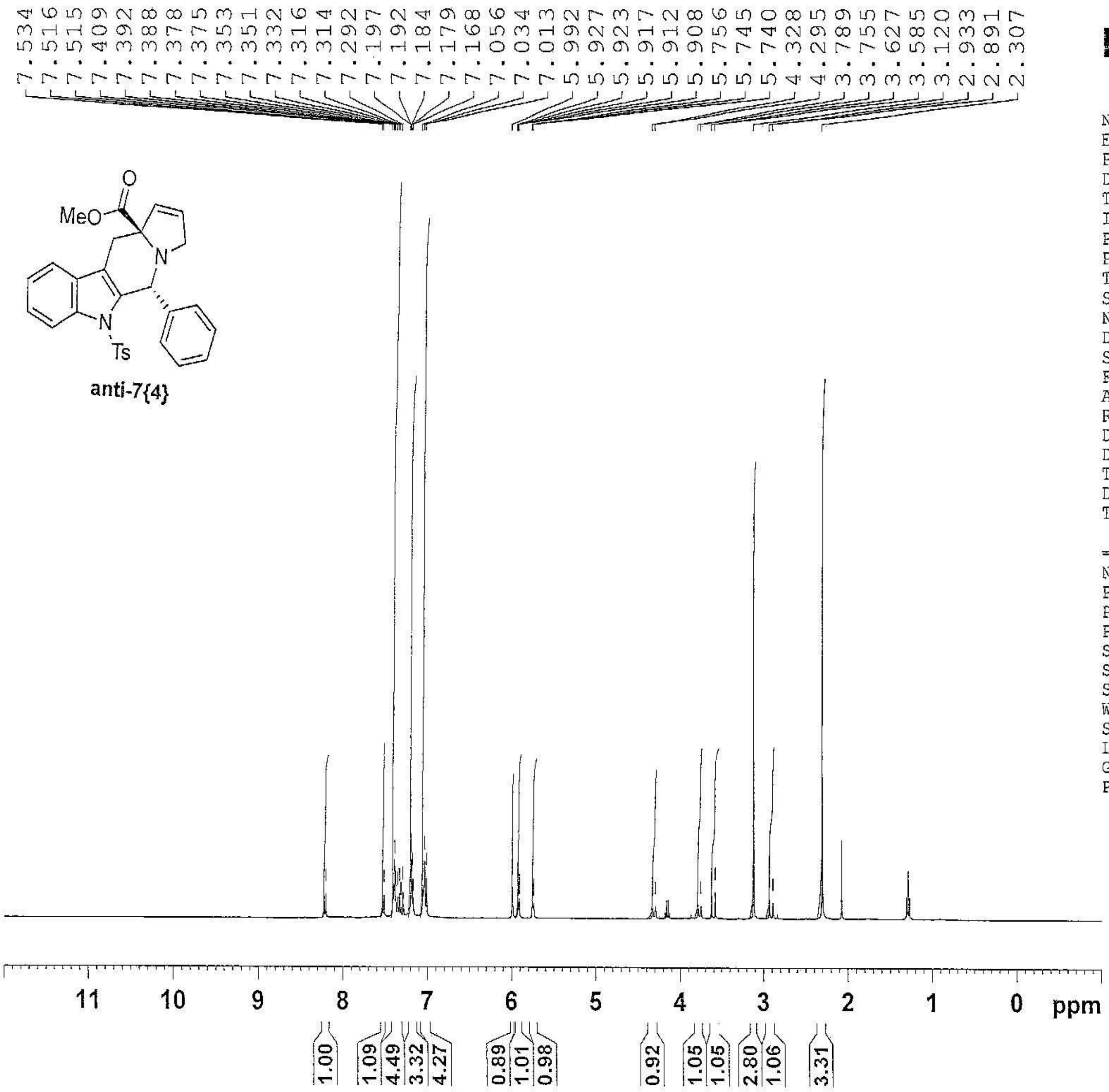

anti-7{4}

NAME	JRG-348-51bre	
EXPNO	1	
PROCNO	1	
Date_	20110727	
Time	3.10	
INSTRUM	spect	
PROBHD	5 mm PABBO BB-	
PULPROG	zg30	
TD	65536	
SOLVENT	CDCl3	
NS	6	
DS	2	
SWH	8223.685	Hz
FIDRES	0.125483	Hz
AQ	3.9846387	sec
RG	90.5	
DW	60.800	usec
DE	6.50	usec
TE	295.0	K
D1	2.00000000	sec
TD0	1	

======== CHANNEL f1 ========

NUC1	1H	
P1	14.31	usec
PL1	-1.00	dB
PL1W	11.09959412	W
SFO1	400.2324716	MHz
SI	32768	
SF	400.2300000	MHz
WDW	EM	
SSB	0	
LB	0.30	Hz
GB	0	
PC	1.00	

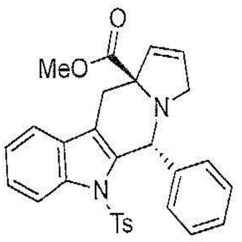

anti-7{4}

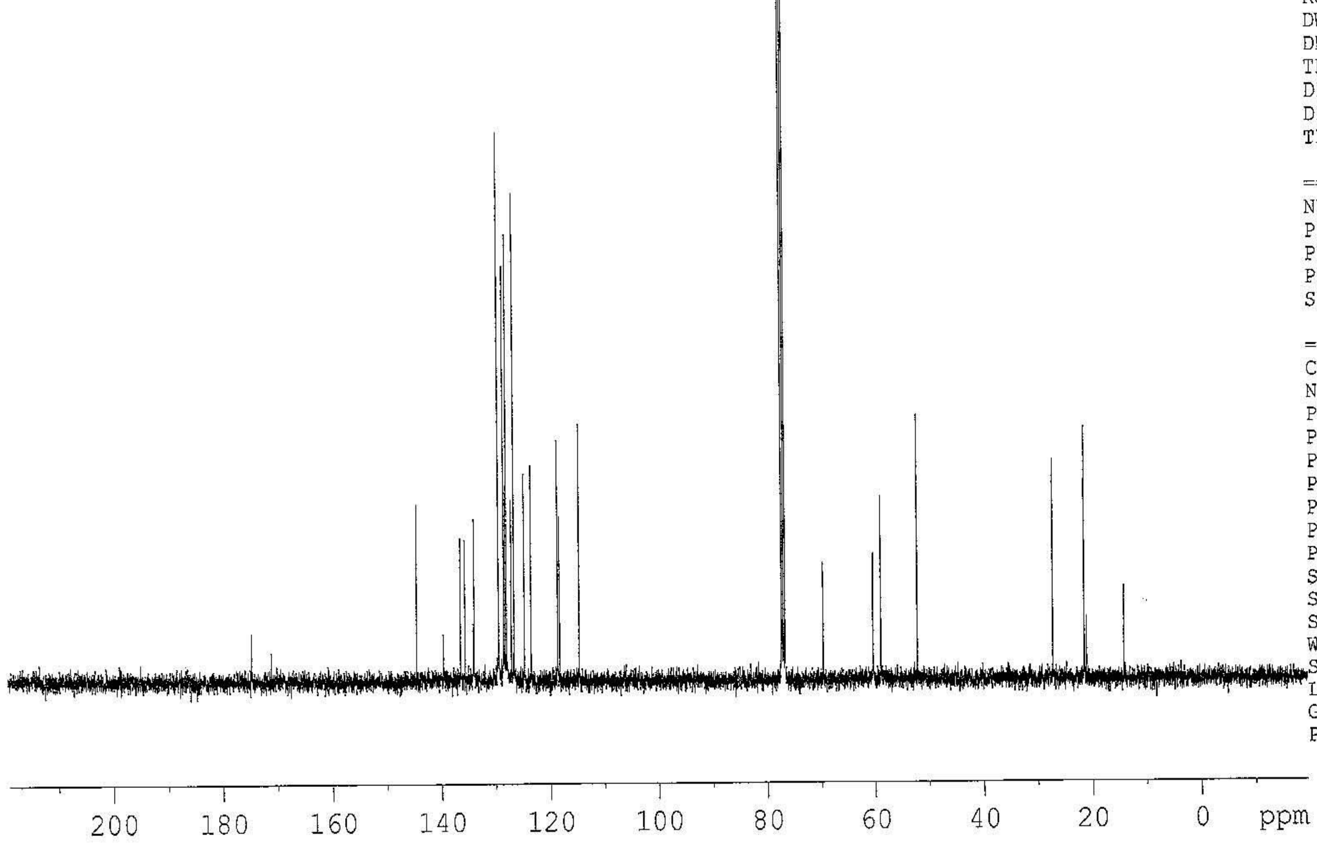

BRUKER

NAME	JRG-348-51bre
EXPNO	2
PROCNO	1
Date_	20110727
Time	3.35
INSTRUM	spect
PROBHD	5 mm PABBO BB-
PULPROG	zgpg30
TD	65536
SOLVENT	CDCl3
NS	300
DS	4
SWH	24038.461 Hz
FIDRES	0.366798 Hz
AQ	1.3631988 sec
RG	203
DW	20.800 usec
DE	6.50 usec
TE	295.5 K
D1	3.00000000 sec
D11	0.03000000 sec
TD0	1

======== CHANNEL f1 ========

NUC1	13C
P1	10.00 usec
PL1	-1.59 dB
PL1W	51.07626343 W
SFO1	100.6479773 MHz

======== CHANNEL f2 ========

CPDPRG2	waltz16
NUC2	1H
PCPD2	75.00 usec
PL2	-1.00 dB
PL12	13.39 dB
PL13	20.00 dB
PL2W	11.09959412 W
PL12W	0.40393090 W
PL13W	0.08816721 W
SFO2	400.2316009 MHz
SI	32768
SF	100.6379200 MHz
WDW	EM
SSB	0
LB	1.00 Hz
GB	0
PC	1.40

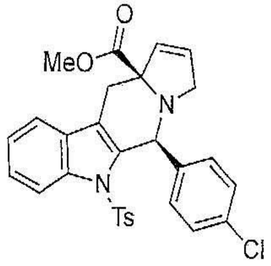

syn-7{5}

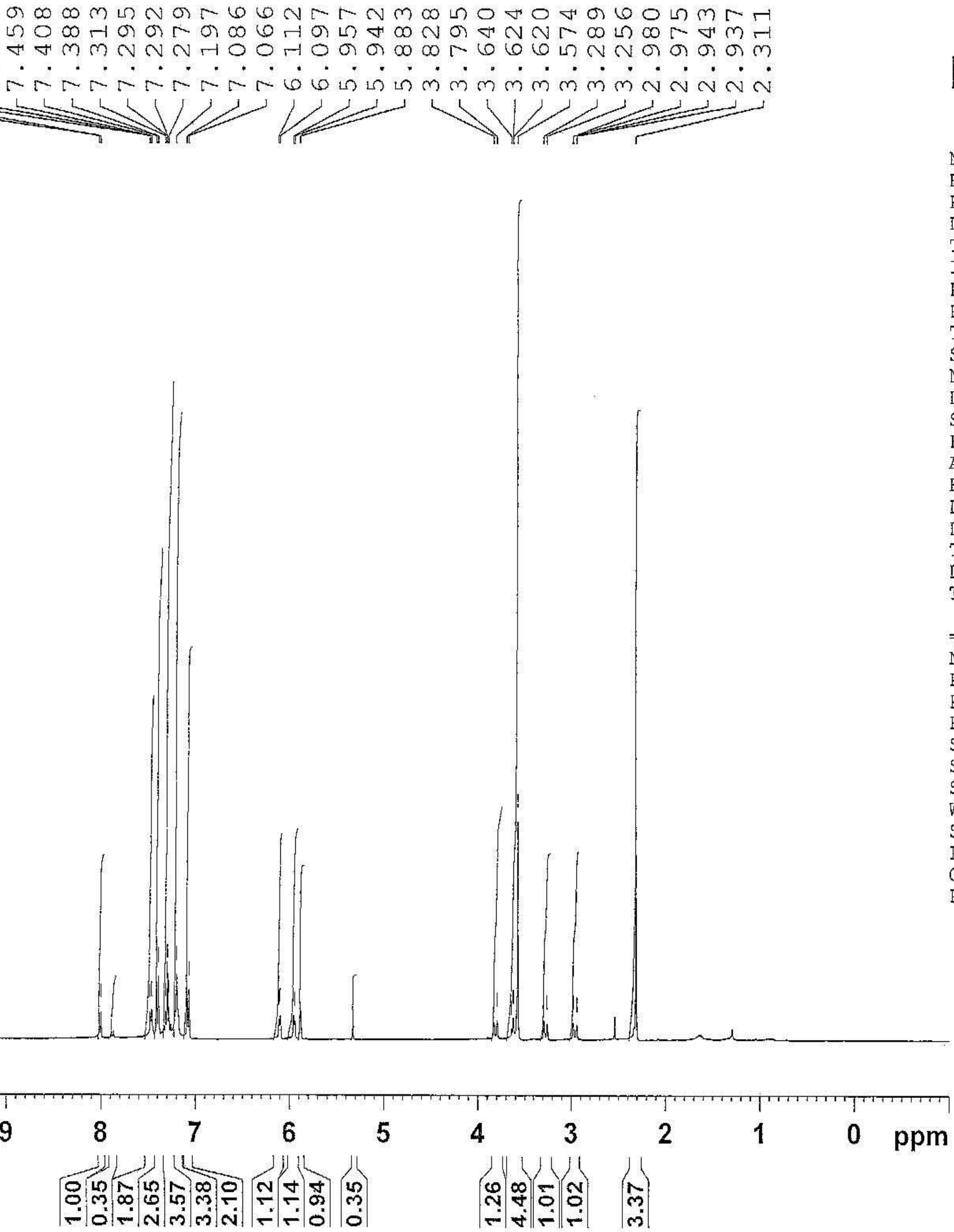

NAME	JRG-348-53are
EXPNO	1
PROCNO	1
Date_	20110727
Time	15.13
INSTRUM	spect
PROBHD	5 mm PABBO BB-
PULPROG	zg30
TD	65536
SOLVENT	CDCl3
NS	8
DS	2
SWH	8223.685 Hz
FIDRES	0.125483 Hz
AQ	3.9846387 sec
RG	114
DW	60.800 usec
DE	6.50 usec
TE	294.9 K
D1	2.00000000 sec
TD0	1

======== CHANNEL f1 ========

NUC1	1H
P1	14.31 usec
PL1	-1.00 dB
PL1W	11.09959412 W
SFO1	400.2324716 MHz
SI	32768
SF	400.2300000 MHz
WDW	EM
SSB	0
LB	0.30 Hz
GB	0
PC	1.00

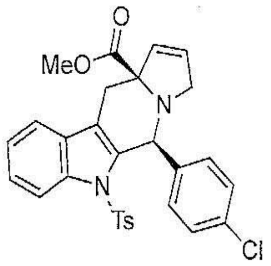

syn-7{5}

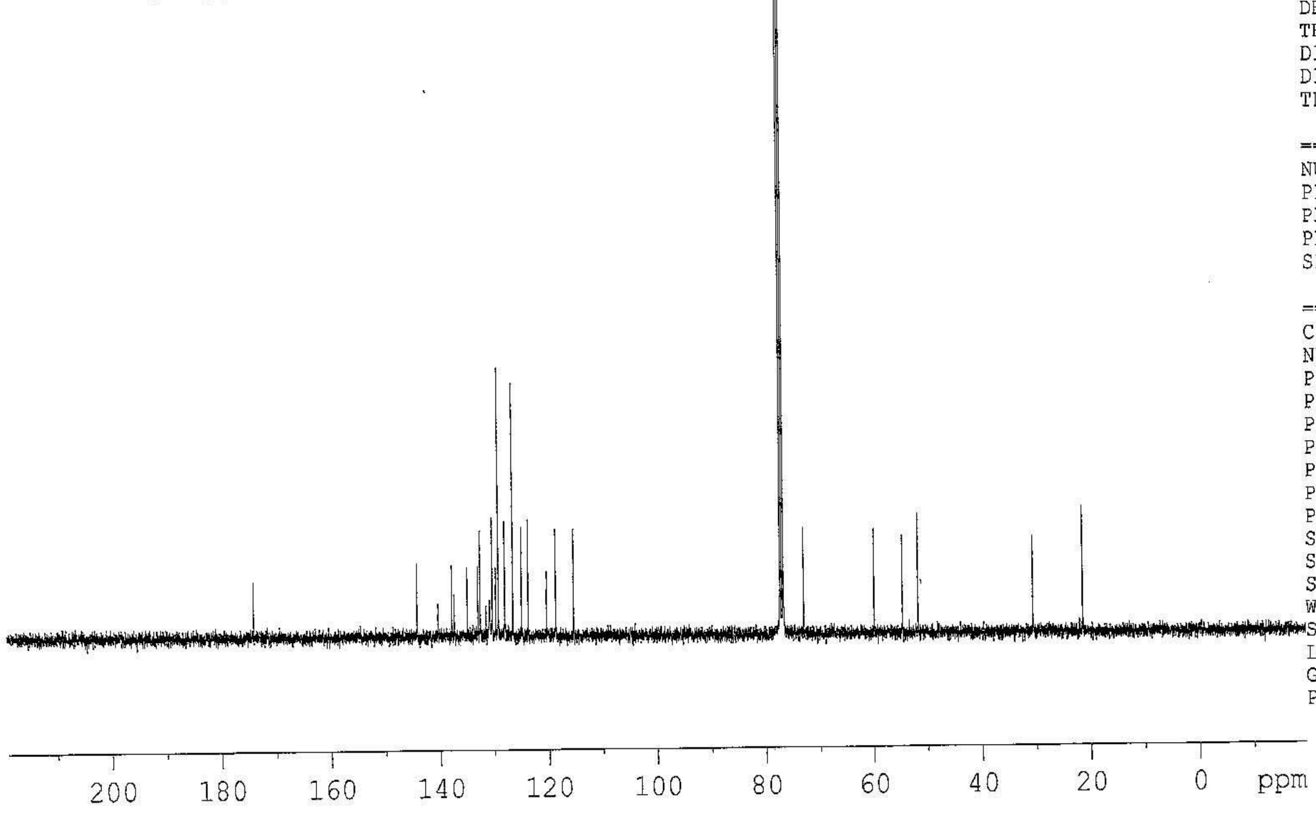

BRUKER

NAME	JRG-348-53are	
EXPNO	2	
PROCNO	1	
Date_	20110727	
Time	15.38	
INSTRUM	spect	
PROBHD	5 mm PABBO BB-	
PULPROG	zgpg30	
TD	65536	
SOLVENT	CDCl3	
NS	318	
DS	4	
SWH	24038.461	Hz
FIDRES	0.366798	Hz
AQ	1.3631988	sec
RG	203	
DW	20.800	usec
DE	6.50	usec
TE	295.3	K
D1	3.00000000	sec
D11	0.03000000	sec
TD0	1	
======== CHANNEL f1 ========		
NUC1	13C	
P1	10.00	usec
PL1	-1.59	dB
PL1W	51.07626343	W
SFO1	100.6479773	MHz
======== CHANNEL f2 ========		
CPDPRG2	waltz16	
NUC2	1H	
PCPD2	75.00	usec
PL2	-1.00	dB
PL12	13.39	dB
PL13	20.00	dB
PL2W	11.09959412	W
PL12W	0.40393090	W
PL13W	0.08816721	W
SFO2	400.2316009	MHz
SI	32768	
SF	100.6379201	MHz
WDW	EM	
SSB	0	
LB	1.00	Hz
GB	0	
PC	1.40	

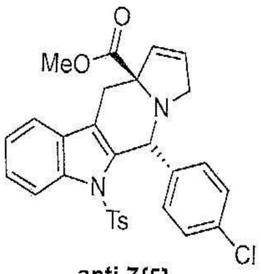

anti-7{5}

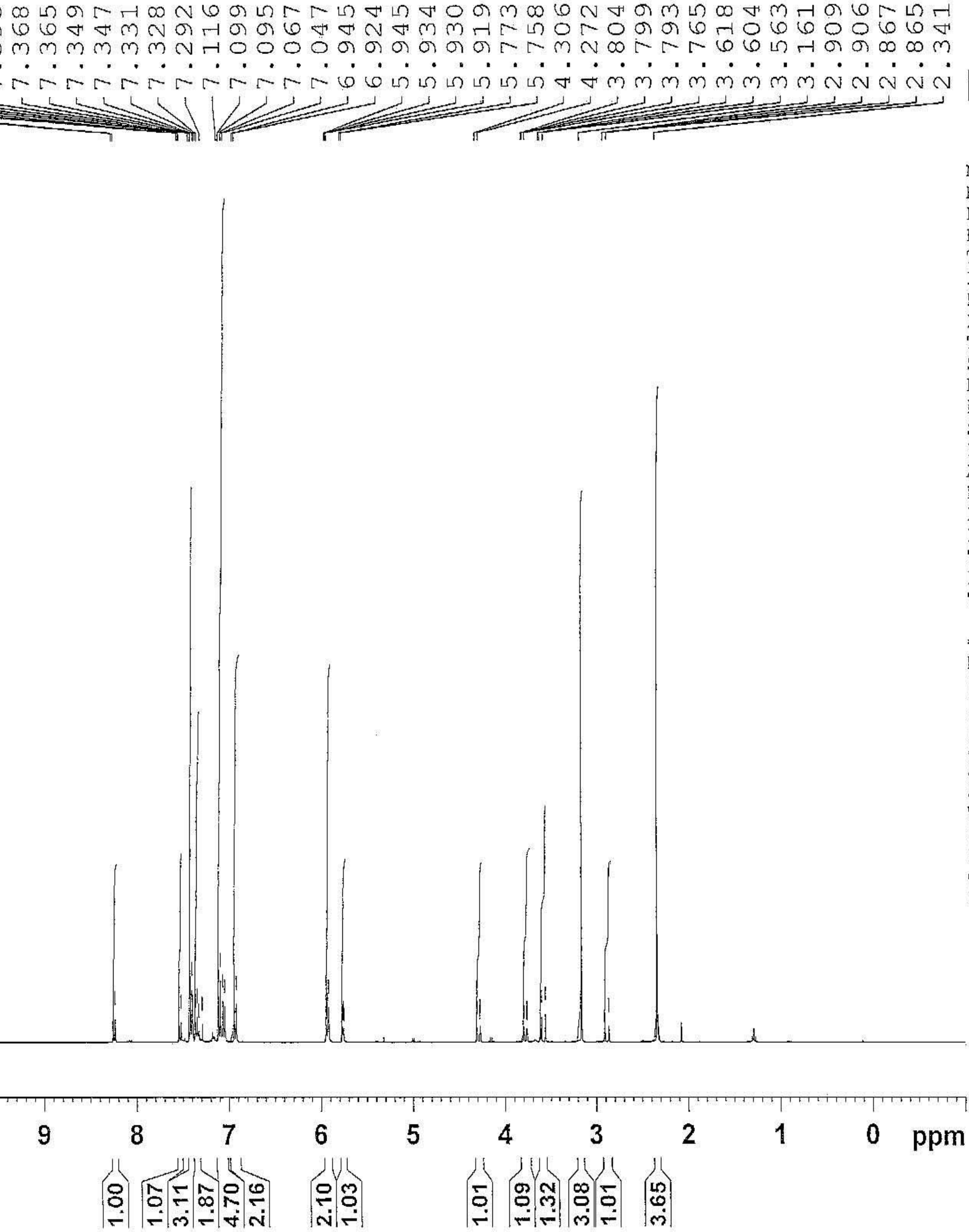

BRUKER

```
NAME          JRG-348-53bre
EXPNO                     1
PROCNO                    1
Date_              20110727
Time                  15.44
INSTRUM               spect
PROBHD      5 mm PABBO BB-
PULPROG                zg30
TD                    65536
SOLVENT               CDCl3
NS                        5
DS                        2
SWH                8223.685 Hz
FIDRES             0.125483 Hz
AQ                3.9846387 sec
RG                     71.8
DW                   60.800 usec
DE                     6.50 usec
TE                    294.9 K
D1               2.00000000 sec
TD0                       1

======== CHANNEL f1 ========
NUC1                     1H
P1                    14.31 usec
PL1                   -1.00 dB
PL1W            11.09959412 W
SFO1            400.2324716 MHz
SI                    32768
SF              400.2300000 MHz
WDW                      EM
SSB                       0
LB                     0.30 Hz
GB                        0
PC                     1.00
```

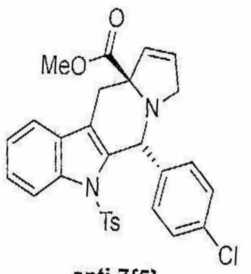

anti-7{5}

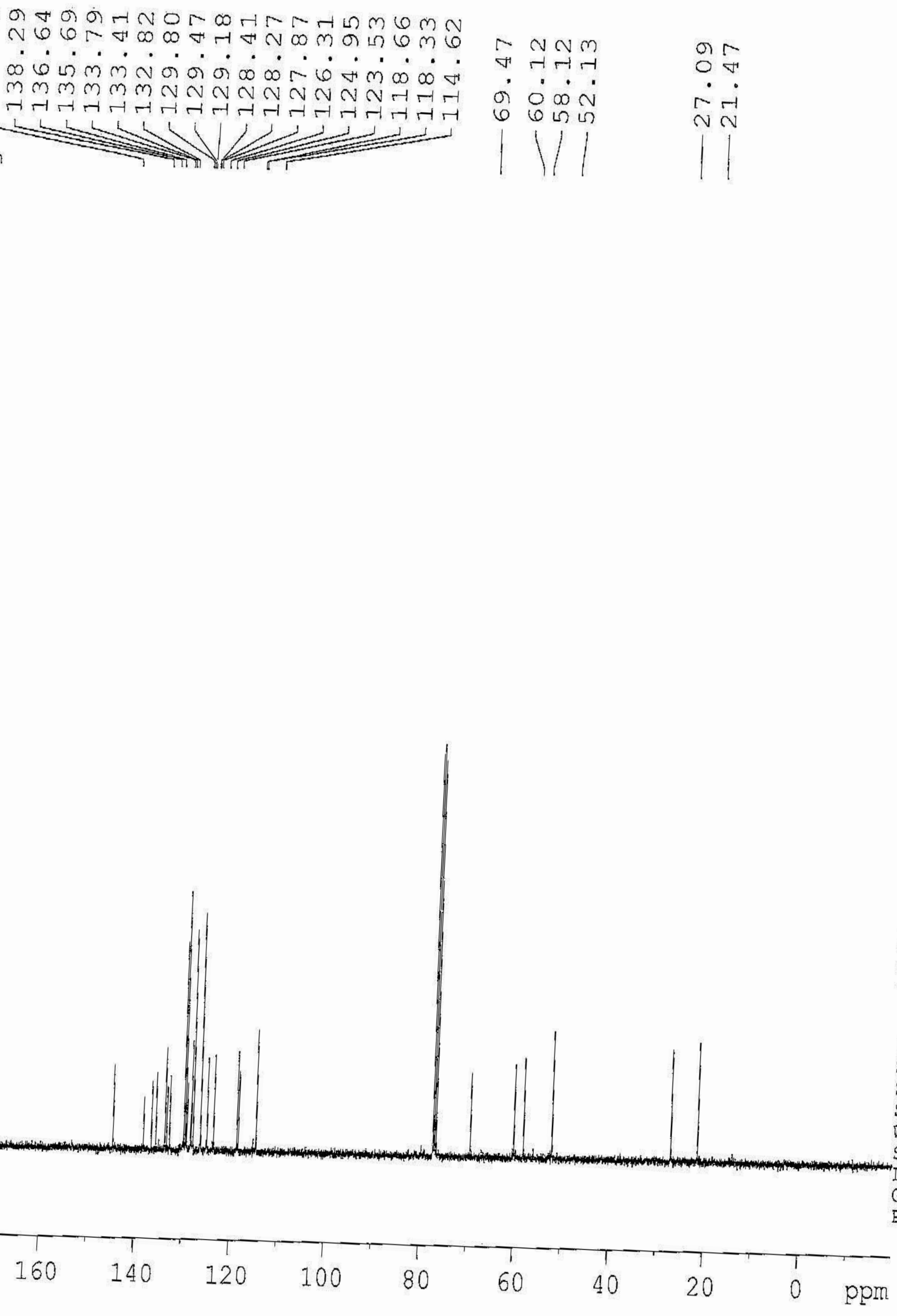

NAME	JRG-348-53bre
EXPNO	2
PROCNO	1
Date_	20110727
Time	15.59
INSTRUM	spect
PROBHD	5 mm PABBO BB-
PULPROG	zgpg30
TD	65536
SOLVENT	CDCl3
NS	177
DS	4
SWH	24038.461 Hz
FIDRES	0.366798 Hz
AQ	1.3631988 sec
RG	203
DW	20.800 usec
DE	6.50 usec
TE	295.4 K
D1	3.00000000 sec
D11	0.03000000 sec
TD0	1

======== CHANNEL f1 ========

NUC1	13C
P1	10.00 usec
PL1	-1.59 dB
PL1W	51.07626343 W
SFO1	100.6479773 MHz

======== CHANNEL f2 ========

CPDPRG2	waltz16
NUC2	1H
PCPD2	75.00 usec
PL2	-1.00 dB
PL12	13.39 dB
PL13	20.00 dB
PL2W	11.09959412 W
PL12W	0.40393090 W
PL13W	0.08816721 W
SFO2	400.2316009 MHz
SI	32768
SF	100.6379203 MHz
WDW	EM
SSB	0
LB	1.00 Hz
GB	0
PC	1.40

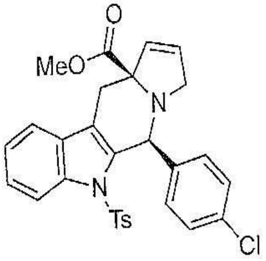

syn-7{5}
Day 0

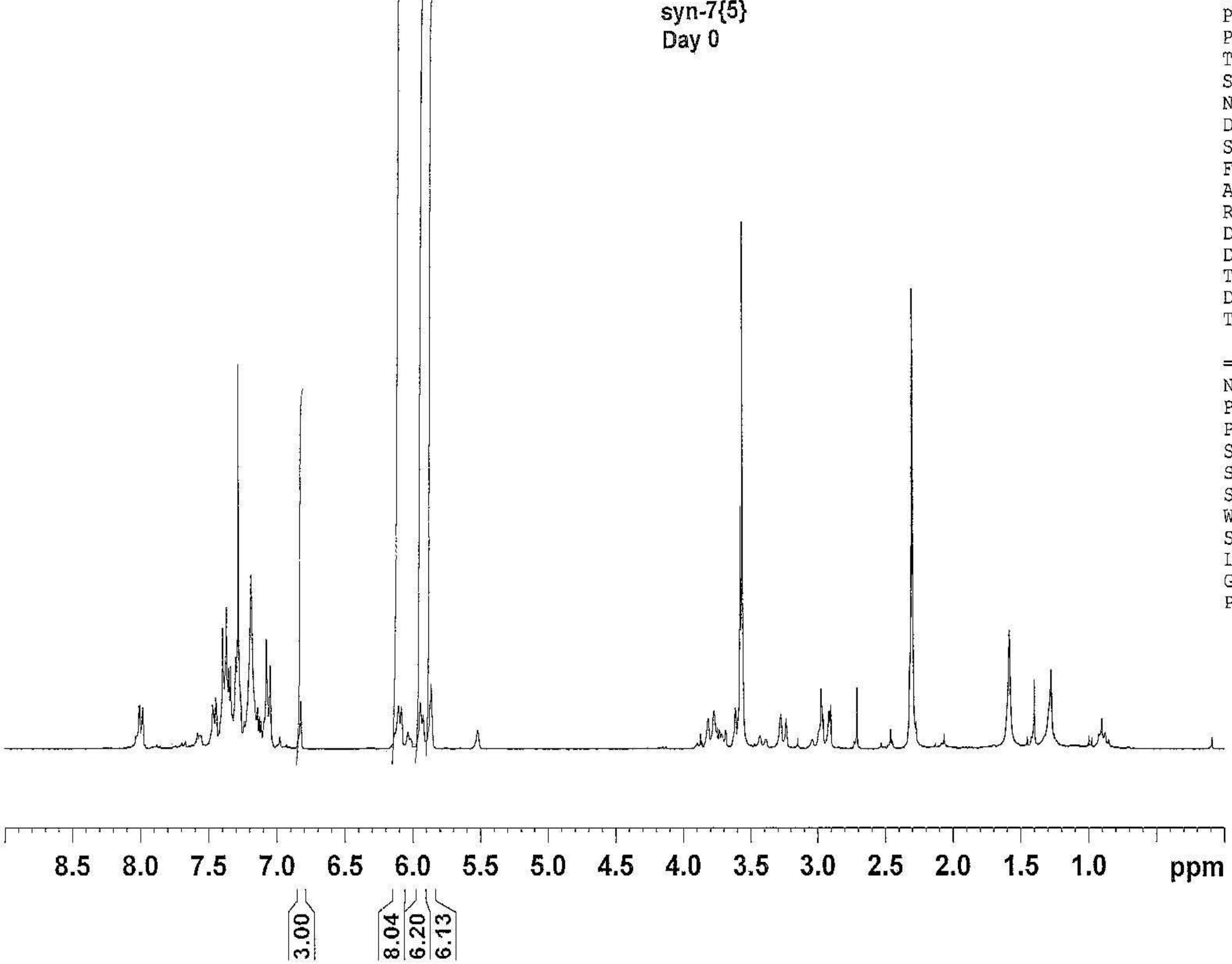

BRUKER

NAME	JRG-329-77a_0day	
EXPNO	1	
PROCNO	1	
Date_	20110427	
Time	15.59	
INSTRUM	spect	
PROBHD	5 mm Dual 13C/	
PULPROG	zg30	
TD	32768	
SOLVENT	CDCl3	
NS	16	
DS	2	
SWH	6172.839	Hz
FIDRES	0.188380	Hz
AQ	2.6542580	sec
RG	645.1	
DW	81.000	usec
DE	6.50	usec
TE	300.0	K
D1	1.00000000	sec
TD0	1	
======== CHANNEL f1 ========		
NUC1	1H	
P1	6.20	usec
PL1	4.00	dB
SFO1	300.1418535	MHz
SI	32768	
SF	300.1400000	MHz
WDW	EM	
SSB	0	
LB	0.30	Hz
GB	0	
PC	1.00	

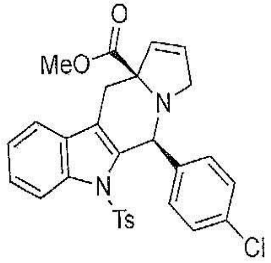

syn-7{5}
Day 14

NAME	JRG-329-77b_air_14day
EXPNO	1
PROCNO	1
Date_	20110512
Time	8.26
INSTRUM	spect
PROBHD	5 mm Multinucl
PULPROG	zg30
TD	32768
SOLVENT	CDCl3
NS	16
DS	2
SWH	6188.119 Hz
FIDRES	0.188846 Hz
AQ	2.6477044 sec
RG	161
DW	80.800 usec
DE	6.50 usec
TE	295.3 K
D1	1.00000000 sec
TD0	1

======== CHANNEL f1 ========

NUC1	1H
P1	13.20 usec
PL1	-2.00 dB
PL1W	18.20942116 W
SFO1	300.2318540 MHz
SI	32768
SF	300.2300000 MHz
WDW	EM
SSB	0
LB	0.10 Hz
GB	0
PC	1.00

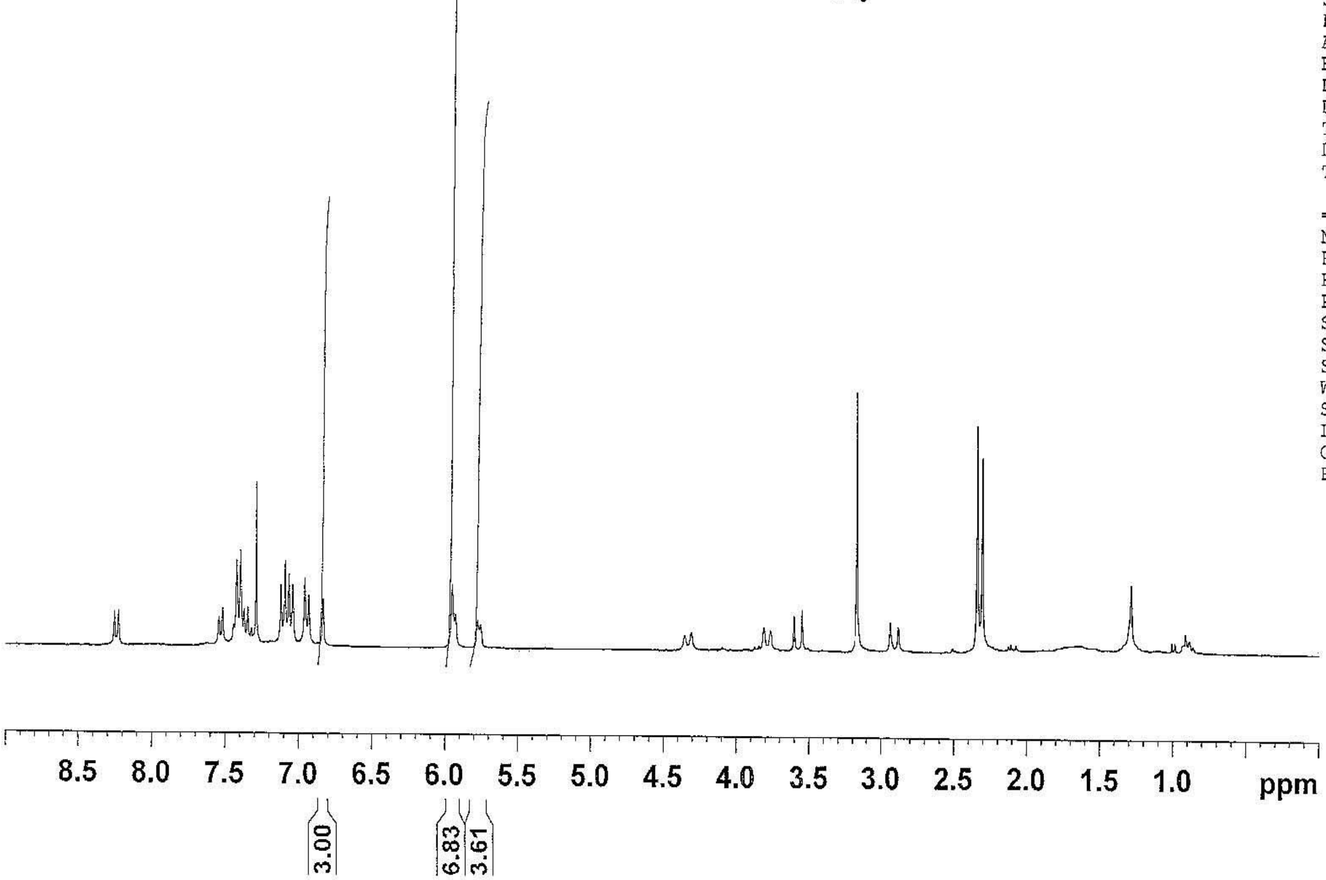

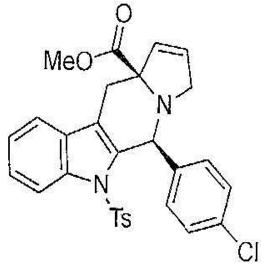

syn-7{5}
Day 21

NAME	JRG-329-77b_air_21day
EXPNO	1
PROCNO	1
Date_	20110519
Time_	7.53
INSTRUM	spect
PROBHD	5 mm Multinucl
PULPROG	zg30
TD	32768
SOLVENT	CDCl3
NS	16
DS	2
SWH	6188.119 Hz
FIDRES	0.188846 Hz
AQ	2.6477044 sec
RG	181
DW	80.800 usec
DE	6.50 usec
TE	294.2 K
D1	1.00000000 sec
TD0	1

======== CHANNEL f1 ========

NUC1	1H
P1	13.20 usec
PL1	-2.00 dB
PL1W	18.20942116 W
SFO1	300.2318540 MHz
SI	32768
SF	300.2300000 MHz
WDW	EM
SSB	0
LB	0.10 Hz
GB	0
PC	1.00

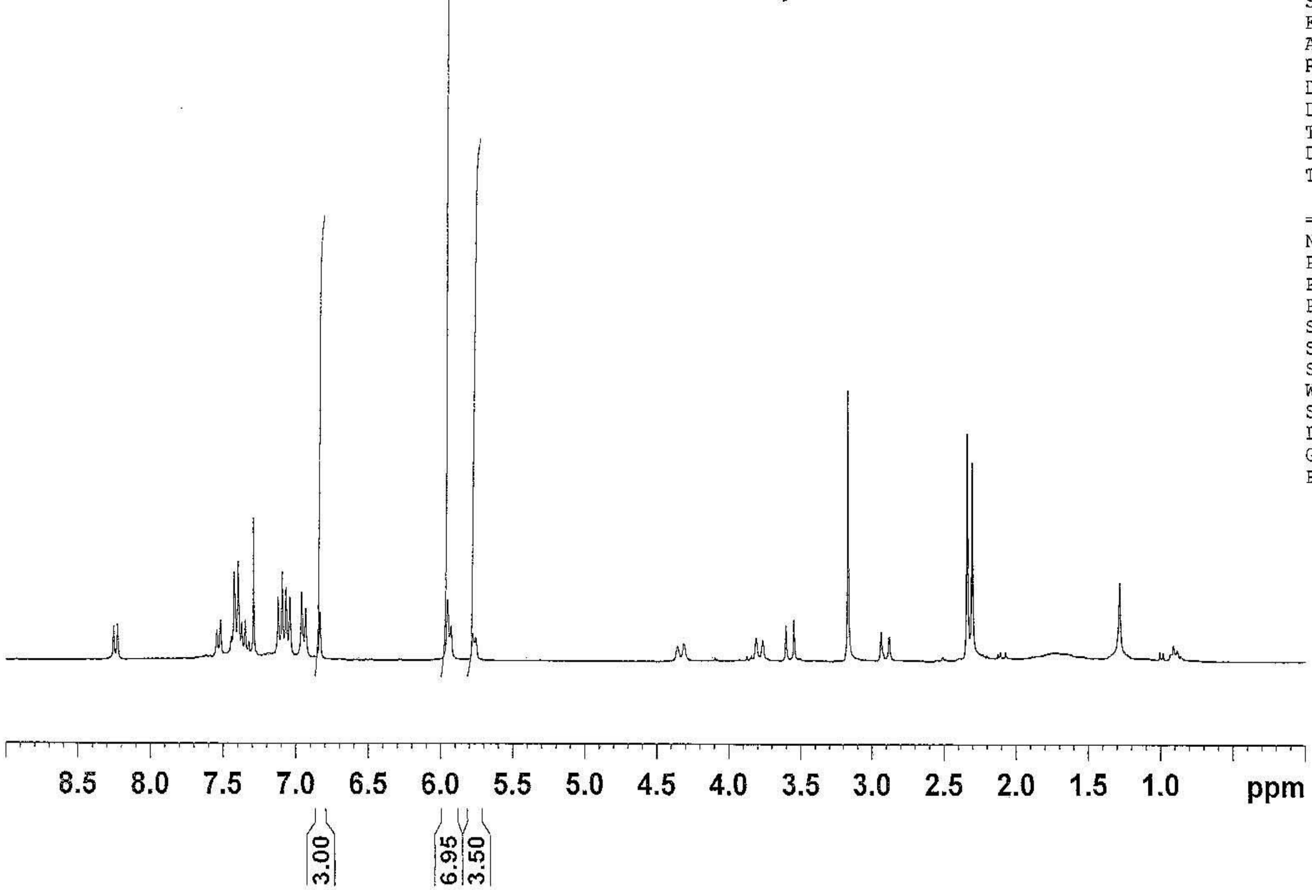

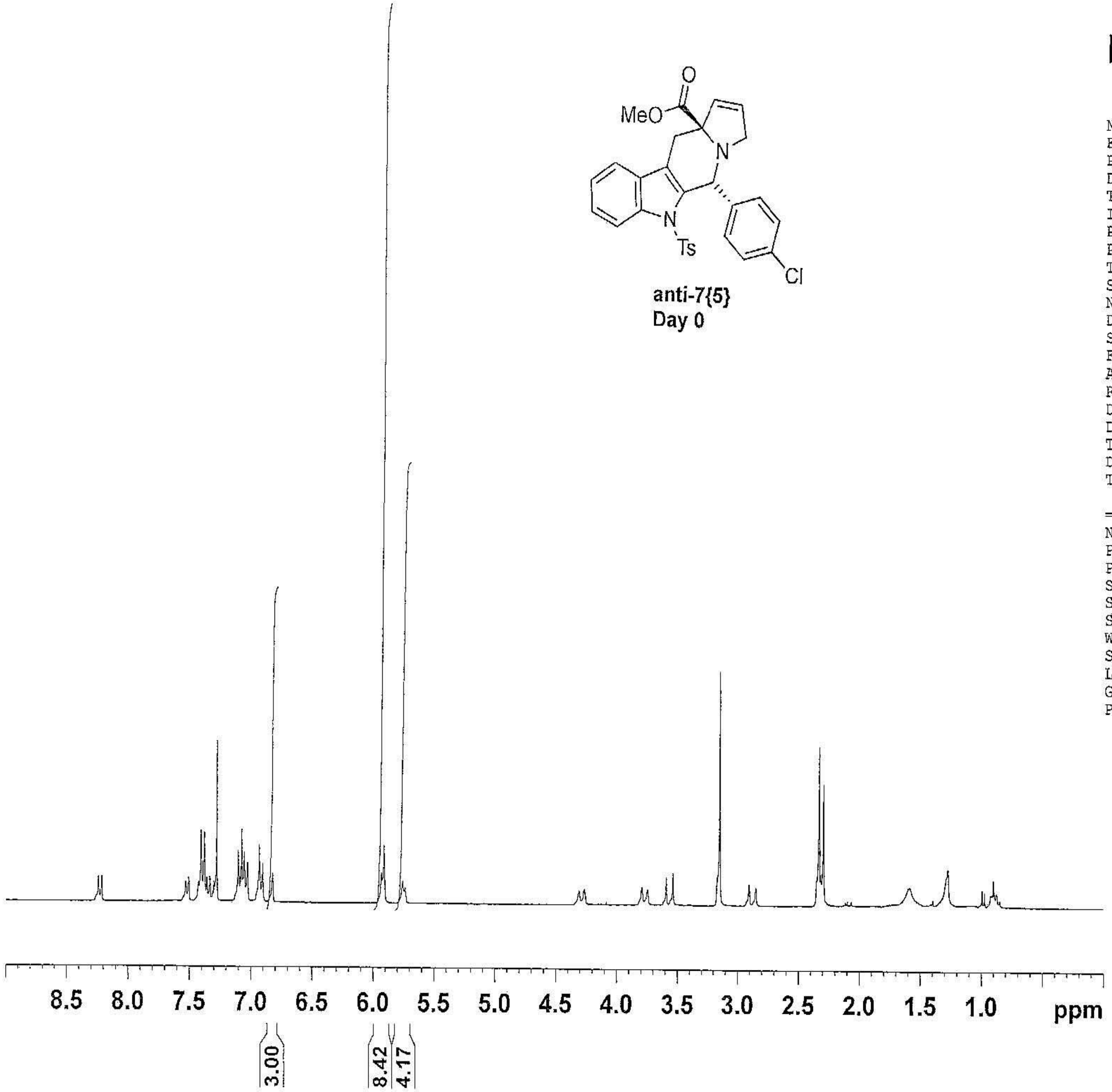

BRUKER

NAME	JRG-329-77b_std_0day_A	
EXPNO	1	
PROCNO	1	
Date_	20110427	
Time	16.07	
INSTRUM	spect	
PROBHD	5 mm Dual 13C/	
PULPROG	zg30	
TD	32768	
SOLVENT	CDCl3	
NS	16	
DS	2	
SWH	6172.839	Hz
FIDRES	0.188380	Hz
AQ	2.6542580	sec
RG	812.7	
DW	81.000	usec
DE	6.50	usec
TE	300.0	K
D1	1.00000000	sec
TD0	1	

======== CHANNEL f1 ========

NUC1	1H	
P1	6.20	usec
PL1	4.00	dB
SFO1	300.1418535	MHz
SI	32768	
SF	300.1400000	MHz
WDW	EM	
SSB	0	
LB	0.30	Hz
GB	0	
PC	1.00	

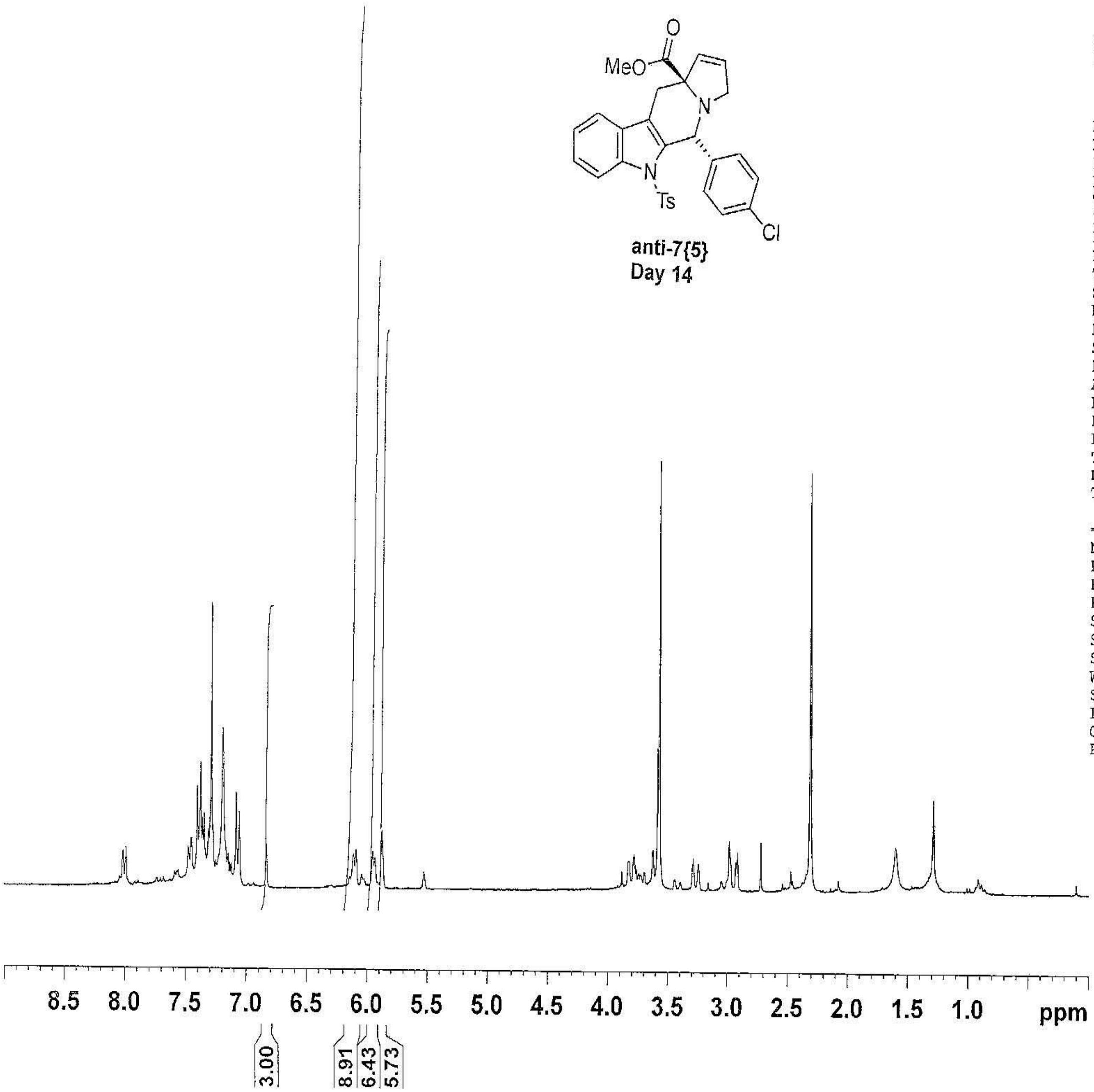

anti-7{5}
Day 14

BRUKER

NAME	JRG-329-77a_air_14day
EXPNO	1
PROCNO	1
Date_	20110512
Time	8.18
INSTRUM	spect
PROBHD	5 mm Multinucl
PULPROG	zg30
TD	32768
SOLVENT	CDCl3
NS	16
DS	2
SWH	6188.119 Hz
FIDRES	0.188846 Hz
AQ	2.6477044 sec
RG	161
DW	80.800 usec
DE	6.50 usec
TE	295.0 K
D1	1.00000000 sec
TD0	1

======== CHANNEL f1 ========

NUC1	1H
P1	13.20 usec
PL1	-2.00 dB
PL1W	18.20942116 W
SFO1	300.2318540 MHz
SI	32768
SF	300.2300000 MHz
WDW	EM
SSB	0
LB	0.10 Hz
GB	0
PC	1.00

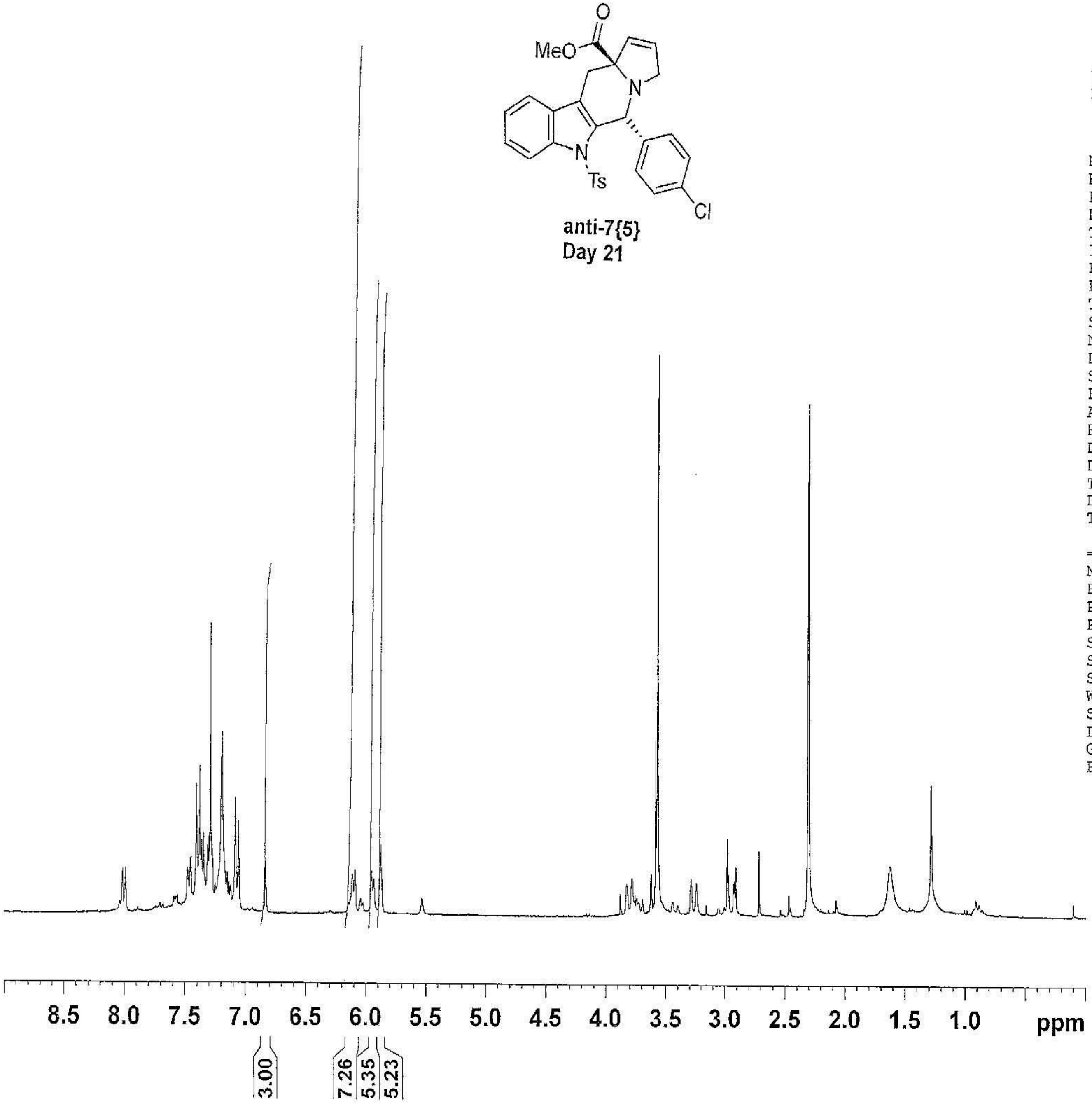

anti-7{5}
Day 21

NAME	JRG-329-77a_air_21day
EXPNO	1
PROCNO	1
Date_	20110519
Time	8.01
INSTRUM	spect
PROBHD	5 mm Multinucl
PULPROG	zg30
TD	32768
SOLVENT	CDCl3
NS	16
DS	2
SWH	6188.119 Hz
FIDRES	0.188846 Hz
AQ	2.6477044 sec
RG	161
DW	80.800 usec
DE	6.50 usec
TE	294.3 K
D1	1.00000000 sec
TD0	1

======== CHANNEL f1 ========

NUC1	1H
P1	13.20 usec
PL1	-2.00 dB
PL1W	18.20942116 W
SFO1	300.2318540 MHz
SI	32768
SF	300.2300000 MHz
WDW	EM
SSB	0
LB	0.10 Hz
GB	0
PC	1.00

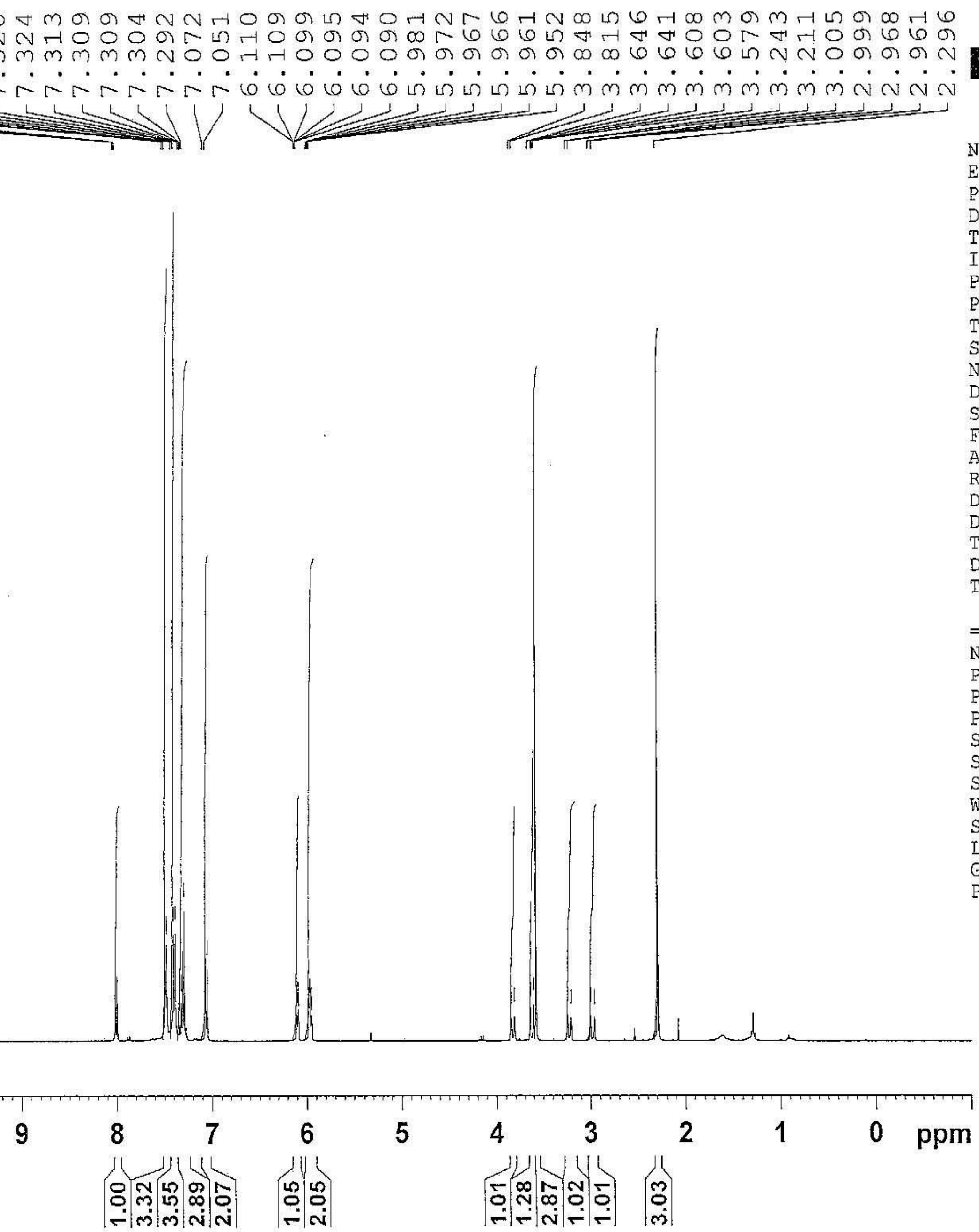

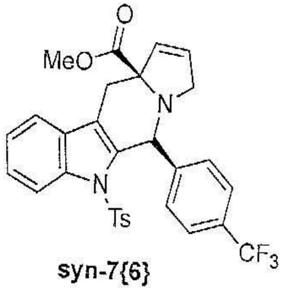

syn-7{6}

BRUKER

NAME	JRG-348-47are
EXPNO	1
PROCNO	1
Date_	20110727
Time_	4.29
INSTRUM	spect
PROBHD	5 mm PABBO BB-
PULPROG	zg30
TD	65536
SOLVENT	CDCl3
NS	7
DS	2
SWH	8223.685 Hz
FIDRES	0.125483 Hz
AQ	3.9846387 sec
RG	128
DW	60.800 usec
DE	6.50 usec
TE	295.2 K
D1	2.00000000 sec
TD0	1

======== CHANNEL f1 ========

NUC1	1H
P1	14.31 usec
PL1	-1.00 dB
PL1W	11.09959412 W
SFO1	400.2324716 MHz
SI	32768
SF	400.2300000 MHz
WDW	EM
SSB	0
LB	0.30 Hz
GB	0
PC	1.00

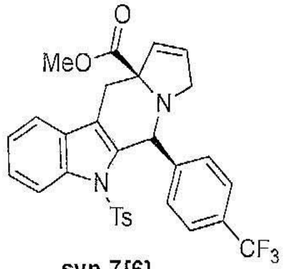

syn-7{6}

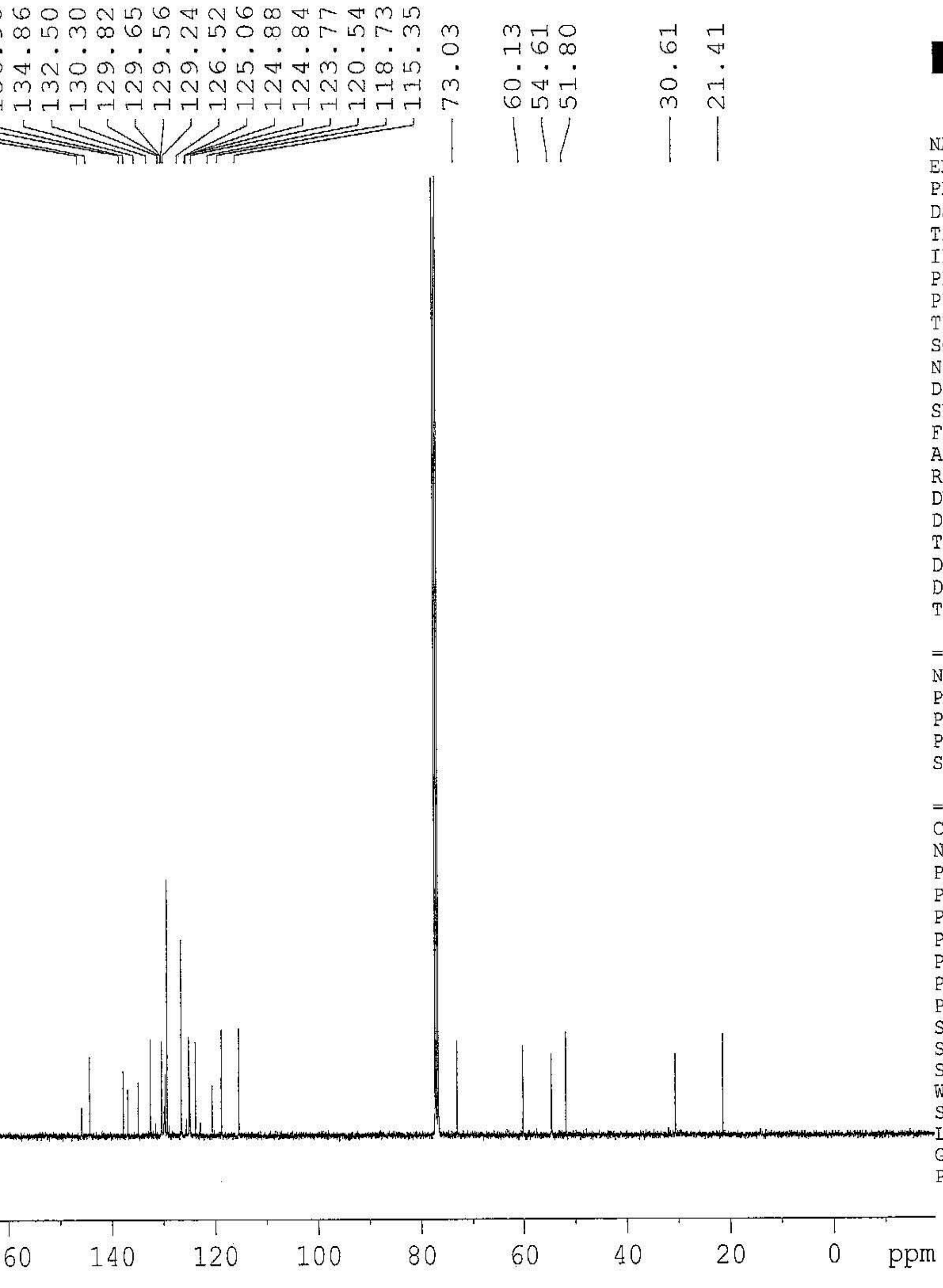

BRUKER

NAME	JRG-348-47are
EXPNO	2
PROCNO	1
Date_	20110727
Time_	5.28
INSTRUM	spect
PROBHD	5 mm PABBO BB-
PULPROG	zgpg30
TD	65536
SOLVENT	CDCl3
NS	775
DS	4
SWH	24038.461 Hz
FIDRES	0.366798 Hz
AQ	1.3631988 sec
RG	203
DW	20.800 usec
DE	6.50 usec
TE	295.5 K
D1	3.00000000 sec
D11	0.03000000 sec
TD0	1

======== CHANNEL f1 ========

NUC1	13C
P1	10.00 usec
PL1	-1.59 dB
PL1W	51.07626343 W
SFO1	100.6479773 MHz

======== CHANNEL f2 ========

CPDPRG2	waltz16
NUC2	1H
PCPD2	75.00 usec
PL2	-1.00 dB
PL12	13.39 dB
PL13	20.00 dB
PL2W	11.09959412 W
PL12W	0.40393090 W
PL13W	0.08816721 W
SFO2	400.2316009 MHz
SI	32768
SF	100.6379189 MHz
WDW	EM
SSB	0
LB	1.00 Hz
GB	0
PC	1.40

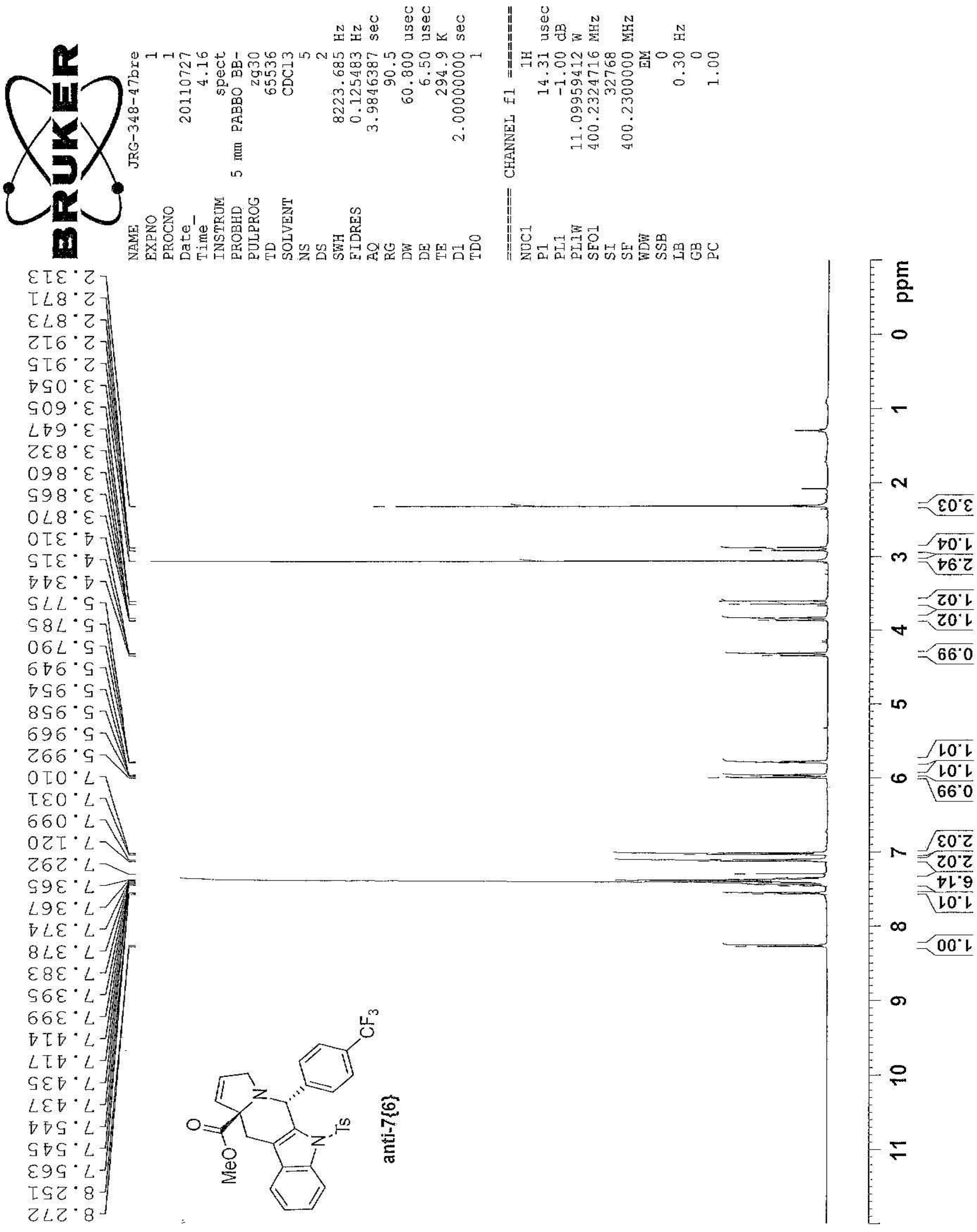
BRUKER
NAME JRG-348-47bre
EXPNO 1
PROCNO 1
Date_ 20110727
Time_ 4.16
INSTRUM spect
PROBHD 5 mm PABBO BB-
PULPROG zg30
TD 65536
SOLVENT CDCl3
NS 5
DS 2
SWH 8223.685 Hz
FIDRES 0.125483 Hz
AQ 3.9846387 sec
RG 90.5
DW 60.800 usec
DE 6.50 usec
TE 294.9 K
D1 2.00000000 sec
TD0 1
======== CHANNEL f1 ========
NUC1 1H
P1 14.31 usec
PL1 -1.00 dB
PL1W 11.09959412 W
SFO1 400.2324716 MHz
SI 32768
SF 400.2300000 MHz
WDW EM
SSB 0
LB 0.30 Hz
GB 0
PC 1.00
2.313 2.871 2.873 2.912 2.915 3.054 3.605 3.647 3.832 3.860 3.865 3.870 4.310 4.315 4.344 5.775 5.785 5.790 5.949 5.954 5.958 5.969 5.992 7.010 7.031 7.099 7.120 7.292 7.365 7.367 7.374 7.378 7.383 7.395 7.399 7.414 7.417 7.435 7.437 7.544 7.545 7.563 8.251 8.272
3.03 1.04 2.94 1.02 1.02 0.99 1.01 1.01 0.99 2.03 2.02 6.14 1.01 1.00
ppm
MeO
O
N
N
Ts
CF3
anti-7{6}

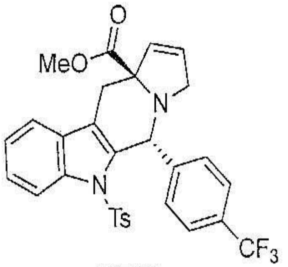

anti-7{6}

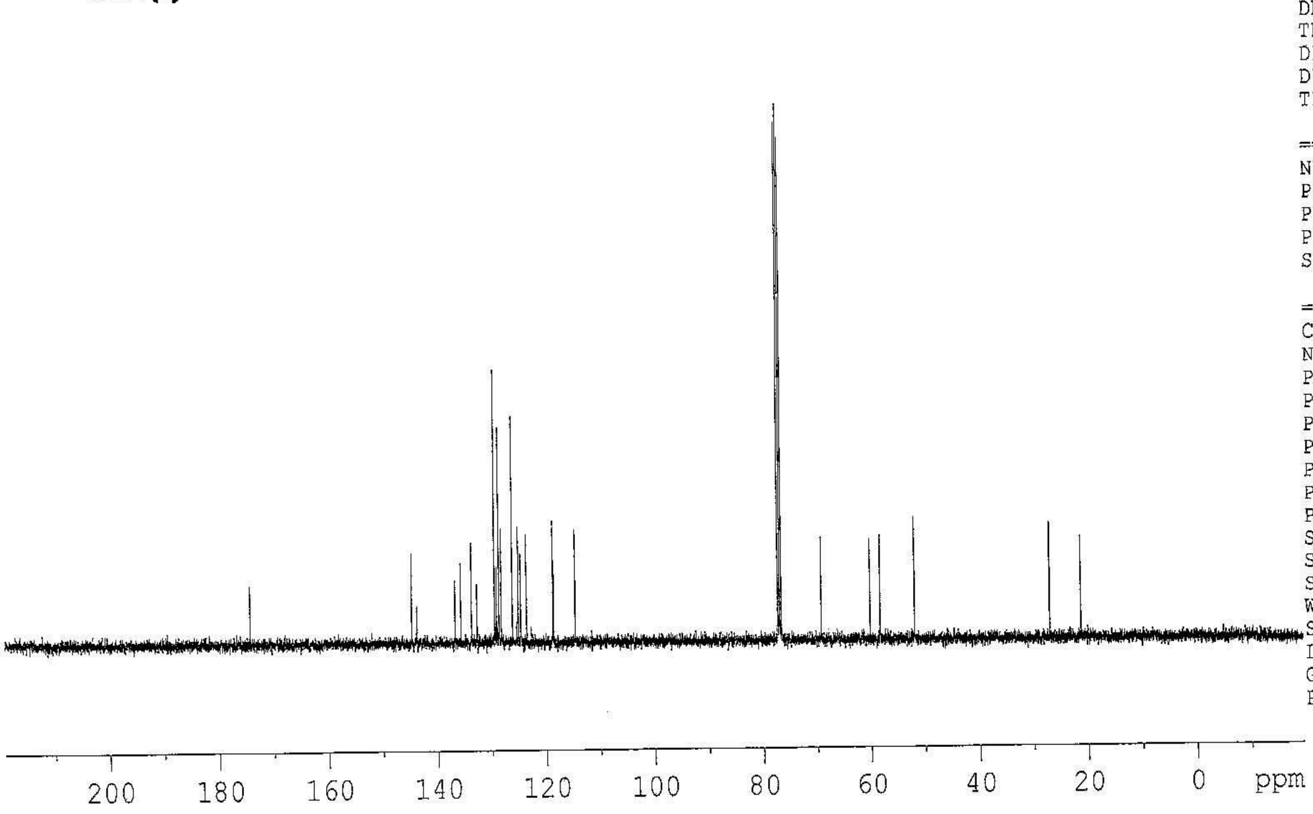

BRUKER

NAME	JRG-348-47bre
EXPNO	2
PROCNO	1
Date_	20110727
Time	4.23
INSTRUM	spect
PROBHD	5 mm PABBO BB-
PULPROG	zgpg30
TD	65536
SOLVENT	CDCl3
NS	67
DS	4
SWH	24038.461 Hz
FIDRES	0.366798 Hz
AQ	1.3631988 sec
RG	203
DW	20.800 usec
DE	6.50 usec
TE	295.5 K
D1	3.00000000 sec
D11	0.03000000 sec
TD0	1

======== CHANNEL f1 ========

NUC1	13C
P1	10.00 usec
PL1	-1.59 dB
PL1W	51.07626343 W
SFO1	100.6479773 MHz

======== CHANNEL f2 ========

CPDPRG2	waltz16
NUC2	1H
PCPD2	75.00 usec
PL2	-1.00 dB
PL12	13.39 dB
PL13	20.00 dB
PL2W	11.09959412 W
PL12W	0.40393090 W
PL13W	0.08816721 W
SFO2	400.2316009 MHz
SI	32768
SF	100.6379199 MHz
WDW	EM
SSB	0
LB	1.00 Hz
GB	0
PC	1.40

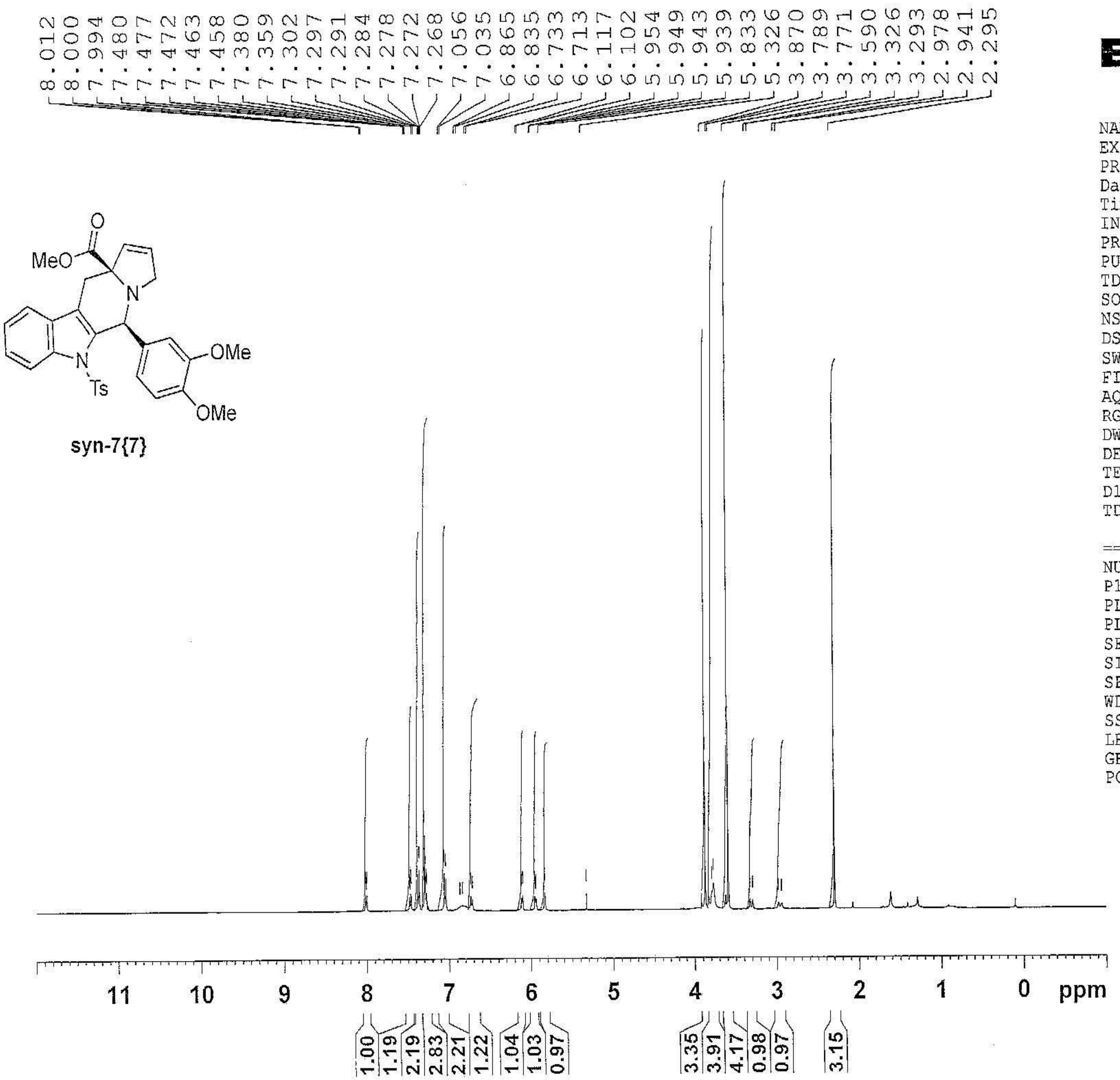

syn-7{7}

BRUKER

NAME	JRG-348-54re
EXPNO	1
PROCNO	1
Date_	20110727
Time	1.46
INSTRUM	spect
PROBHD	5 mm PABBO BB-
PULPROG	zg30
TD	65536
SOLVENT	CDCl3
NS	12
DS	2
SWH	8223.685 Hz
FIDRES	0.125483 Hz
AQ	3.9846387 sec
RG	144
DW	60.800 usec
DE	6.50 usec
TE	295.0 K
D1	2.00000000 sec
TD0	1

======== CHANNEL f1 ========

NUC1	1H
P1	14.31 usec
PL1	-1.00 dB
PL1W	11.09959412 W
SFO1	400.2324716 MHz
SI	32768
SF	400.2300000 MHz
WDW	EM
SSB	0
LB	0.30 Hz
GB	0
PC	1.00

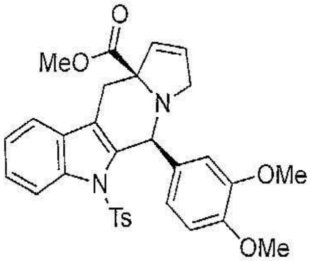

syn-7{7}

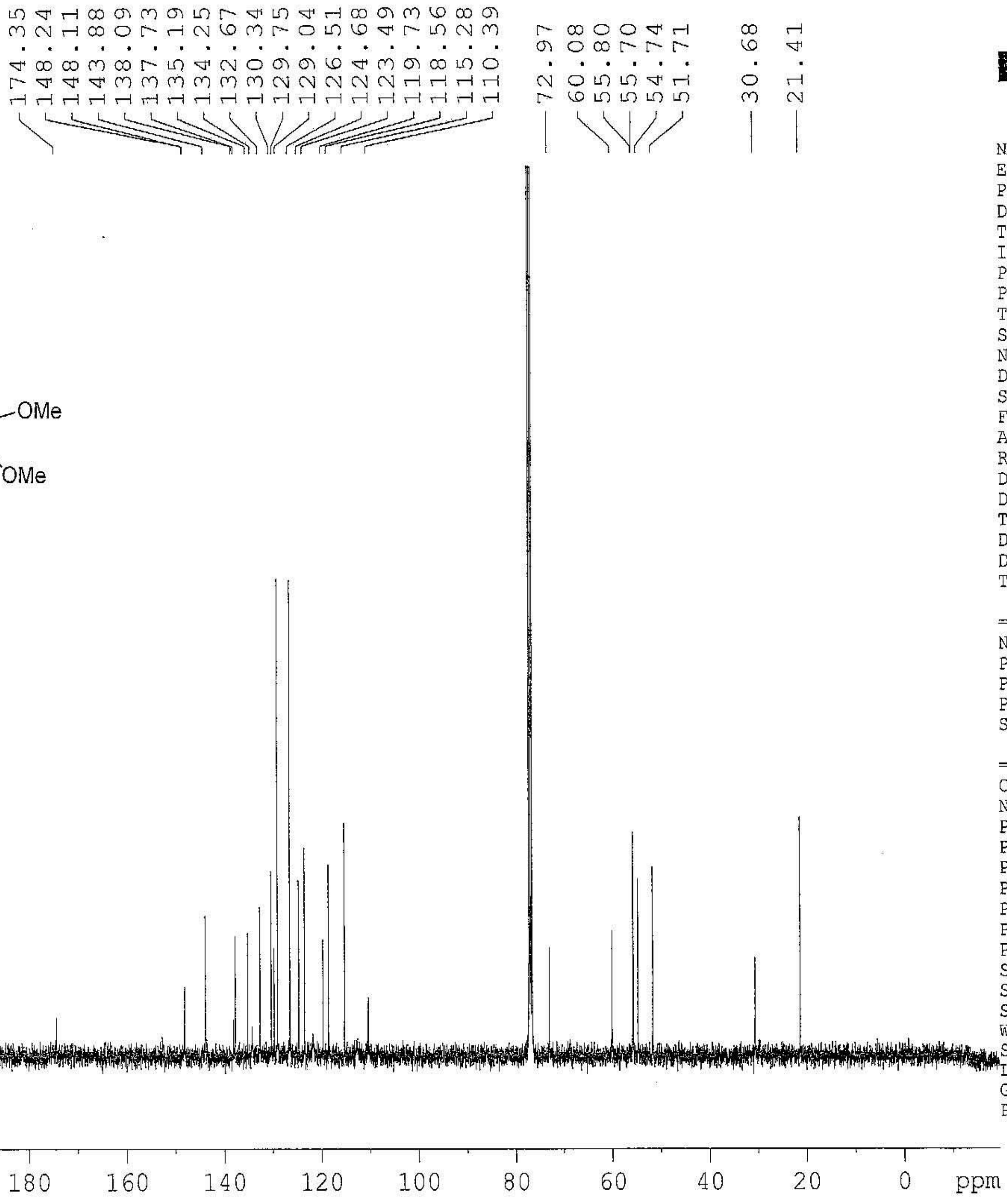

BRUKER

NAME	JRG-348-54re
EXPNO	2
PROCNO	1
Date_	20110727
Time	3.04
INSTRUM	spect
PROBHD	5 mm PABBO BB-
PULPROG	zgpg30
TD	65536
SOLVENT	CDCl3
NS	1024
DS	4
SWH	24038.461 Hz
FIDRES	0.366798 Hz
AQ	1.3631988 sec
RG	203
DW	20.800 usec
DE	6.50 usec
TE	295.5 K
D1	3.00000000 sec
D11	0.03000000 sec
TD0	1

======== CHANNEL f1 ========

NUC1	13C
P1	10.00 usec
PL1	-1.59 dB
PL1W	51.07626343 W
SFO1	100.6479773 MHz

======== CHANNEL f2 ========

CPDPRG2	waltz16
NUC2	1H
PCPD2	75.00 usec
PL2	-1.00 dB
PL12	13.39 dB
PL13	20.00 dB
PL2W	11.09959412 W
PL12W	0.40393090 W
PL13W	0.08816721 W
SFO2	400.2316009 MHz
SI	32768
SF	100.6379206 MHz
WDW	EM
SSB	0
LB	1.00 Hz
GB	0
PC	1.40

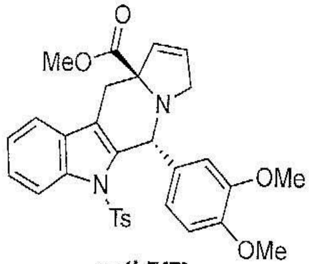

anti-7{7}

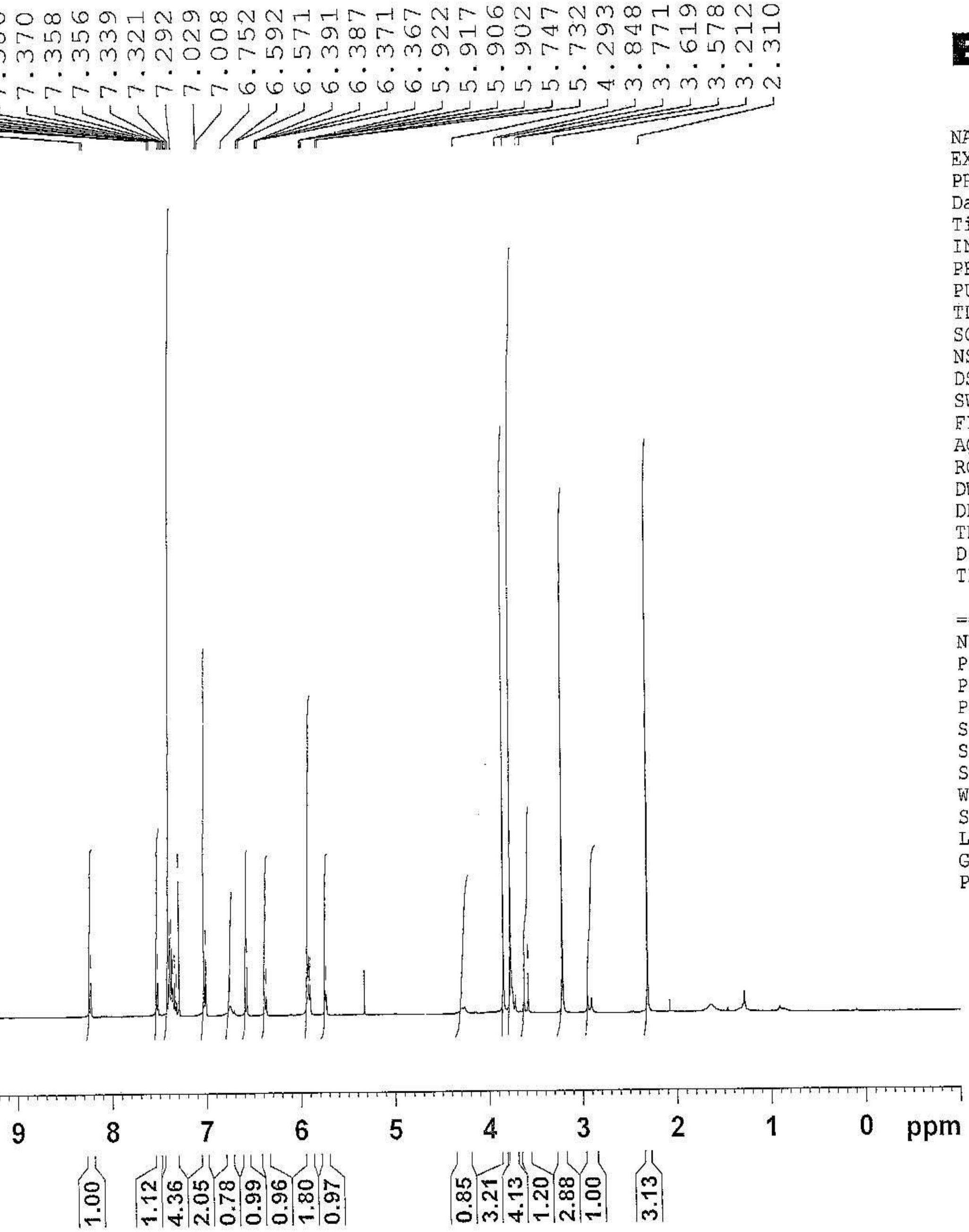

BRUKER

NAME	JRG-348-54are
EXPNO	1
PROCNO	1
Date_	20110727
Time	5.34
INSTRUM	spect
PROBHD	5 mm PABBO BB-
PULPROG	zg30
TD	65536
SOLVENT	CDCl3
NS	4
DS	2
SWH	8223.685 Hz
FIDRES	0.125483 Hz
AQ	3.9846387 sec
RG	144
DW	60.800 usec
DE	6.50 usec
TE	295.2 K
D1	2.00000000 sec
TD0	1

======== CHANNEL f1 ========

NUC1	1H
P1	14.31 usec
PL1	-1.00 dB
PL1W	11.09959412 W
SFO1	400.2324716 MHz
SI	32768
SF	400.2300000 MHz
WDW	EM
SSB	0
LB	0.30 Hz
GB	0
PC	1.00

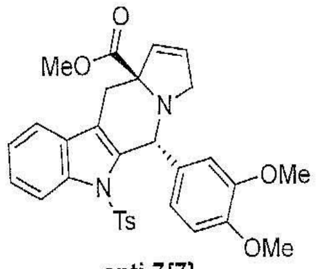

anti-7{7}

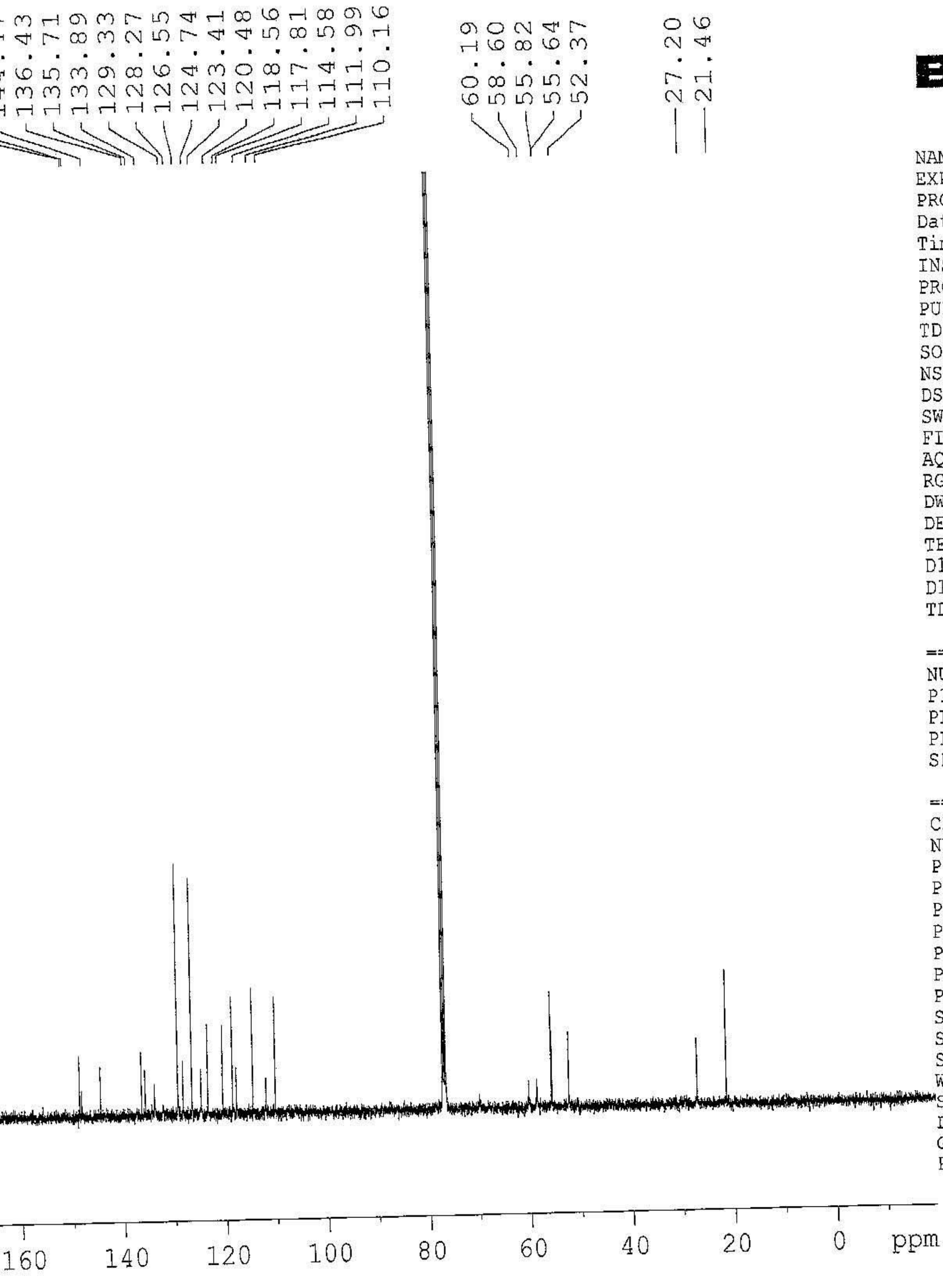

BRUKER

NAME	JRG-348-54are
EXPNO	2
PROCNO	1
Date_	20110727
Time	7.06
INSTRUM	spect
PROBHD	5 mm PABBO BB-
PULPROG	zgpg30
TD	65536
SOLVENT	CDCl3
NS	1200
DS	4
SWH	24038.461 Hz
FIDRES	0.366798 Hz
AQ	1.3631988 sec
RG	203
DW	20.800 usec
DE	6.50 usec
TE	295.6 K
D1	3.00000000 sec
D11	0.03000000 sec
TD0	1

======== CHANNEL f1 ========

NUC1	13C
P1	10.00 usec
PL1	-1.59 dB
PL1W	51.07626343 W
SFO1	100.6479773 MHz

======== CHANNEL f2 ========

CPDPRG2	waltz16
NUC2	1H
PCPD2	75.00 usec
PL2	-1.00 dB
PL12	13.39 dB
PL13	20.00 dB
PL2W	11.09959412 W
PL12W	0.40393090 W
PL13W	0.08816721 W
SFO2	400.2316009 MHz
SI	32768
SF	100.6379182 MHz
WDW	EM
SSB	0
LB	1.00 Hz
GB	0
PC	1.40

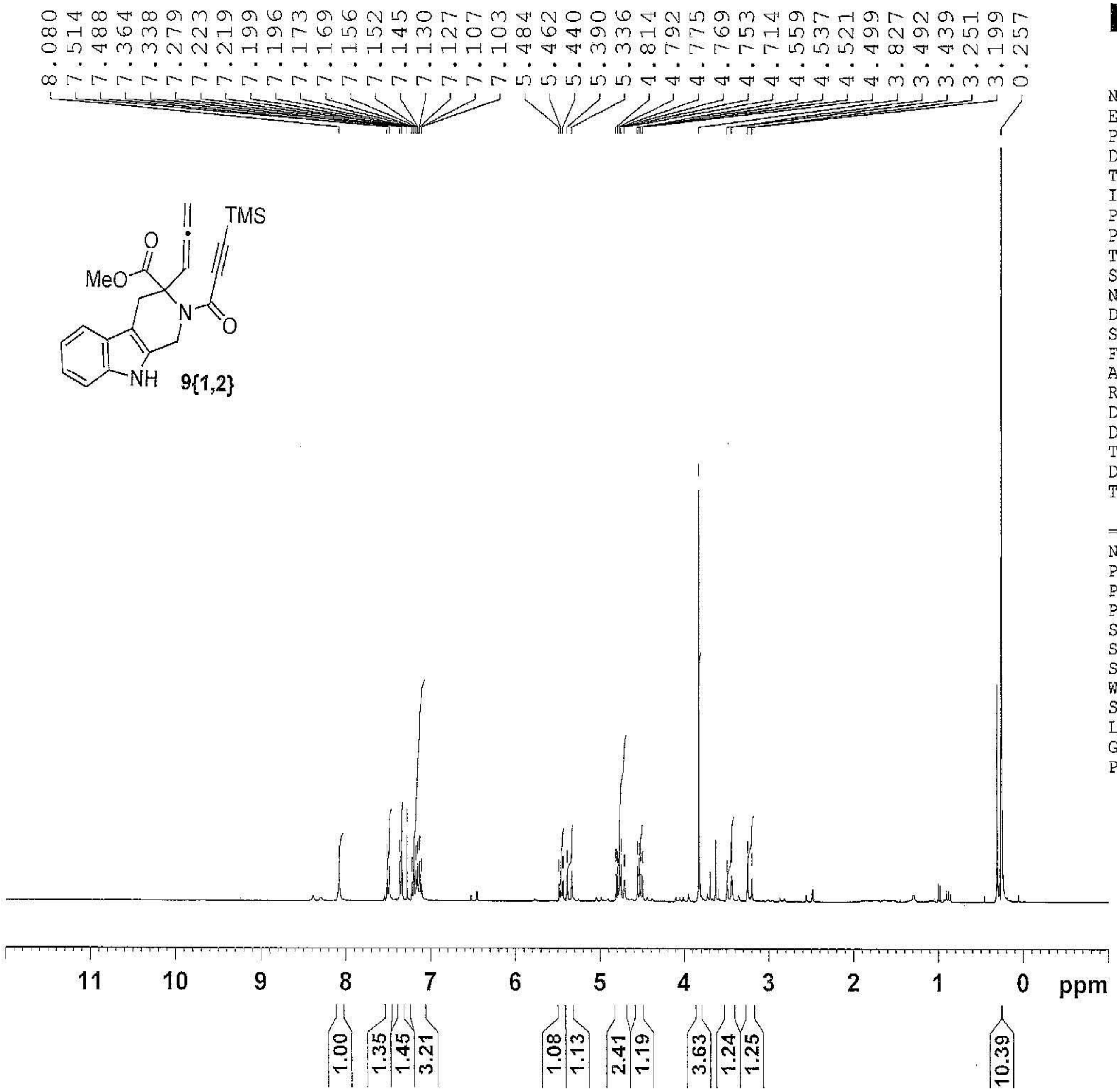

BRUKER

```
NAME          JRG-348-67
EXPNO                  1
PROCNO                 1
Date_           20110718
Time               10.43
INSTRUM            spect
PROBHD   5 mm Dual 13C/
PULPROG             zg30
TD                 32768
SOLVENT            CDCl3
NS                    12
DS                     2
SWH          6172.839 Hz
FIDRES       0.188380 Hz
AQ          2.6542580 sec
RG                   362
DW            81.000 usec
DE              6.50 usec
TE               296.2 K
D1         1.00000000 sec
TD0                    1

======== CHANNEL f1 ========
NUC1                  1H
P1              6.20 usec
PL1               4.00 dB
PL1W        4.57399988 W
SFO1     300.1418535 MHz
SI                 32768
SF       300.1400000 MHz
WDW                   EM
SSB                    0
LB               0.30 Hz
GB                     0
PC                  1.00
```

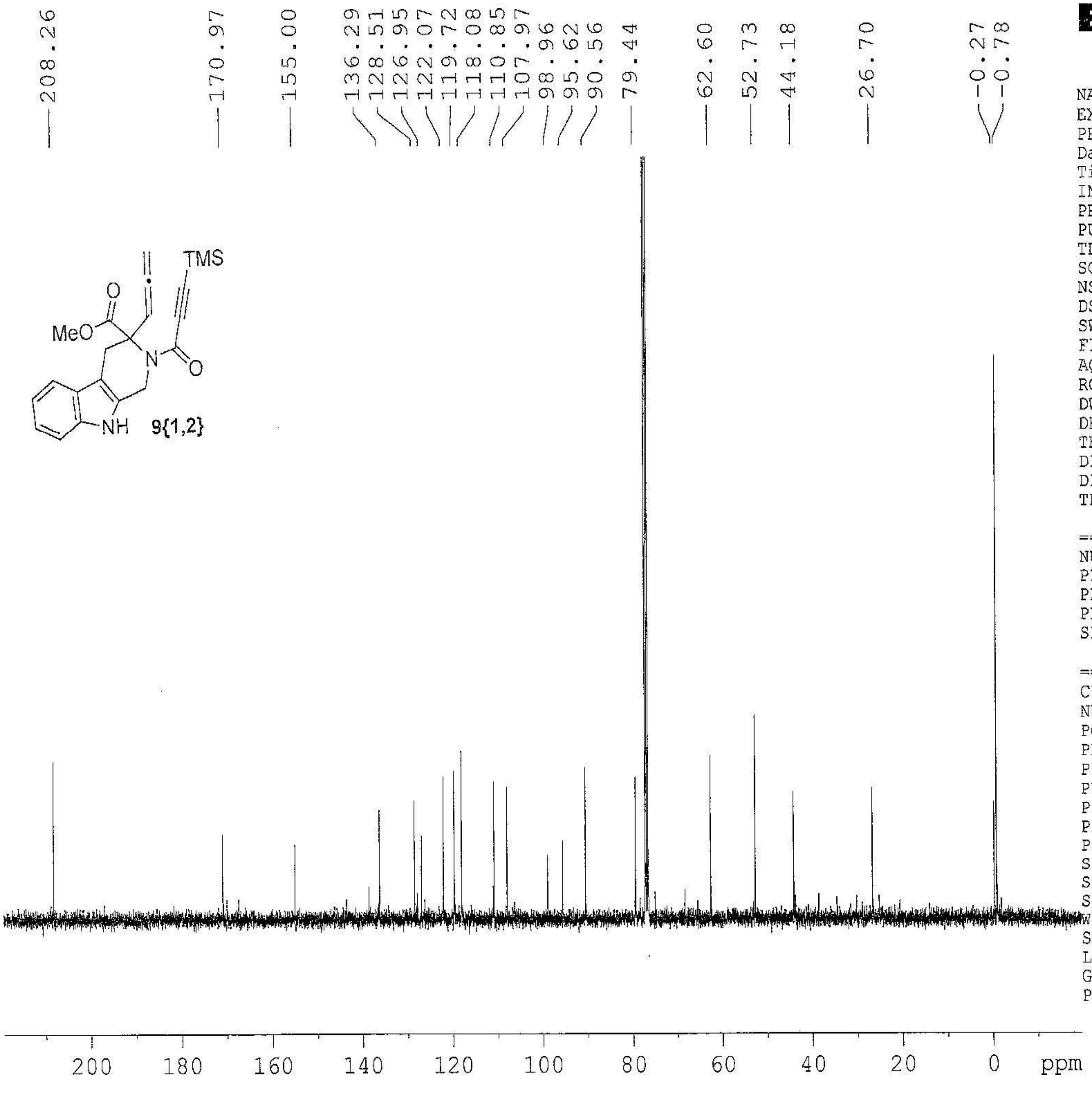

9{1,2}

NAME	JRG-348-67_carbon
EXPNO	1
PROCNO	1
Date_	20110718
Time_	13.47
INSTRUM	spect
PROBHD	5 mm PABBO BB-
PULPROG	zgpg30
TD	65536
SOLVENT	CDCl3
NS	211
DS	4
SWH	24038.461 Hz
FIDRES	0.366798 Hz
AQ	1.3631988 sec
RG	203
DW	20.800 usec
DE	6.50 usec
TE	296.3 K
D1	3.00000000 sec
D11	0.03000000 sec
TD0	1

======== CHANNEL f1 ========

NUC1	13C
P1	10.00 usec
PL1	-1.59 dB
PL1W	51.07626343 W
SFO1	100.6479773 MHz

======== CHANNEL f2 ========

CPDPRG2	waltz16
NUC2	1H
PCPD2	75.00 usec
PL2	-1.00 dB
PL12	13.39 dB
PL13	20.00 dB
PL2W	11.09959412 W
PL12W	0.40393090 W
PL13W	0.08816721 W
SFO2	400.2316009 MHz
SI	32768
SF	100.6379221 MHz
WDW	EM
SSB	0
LB	1.00 Hz
GB	0
PC	1.40

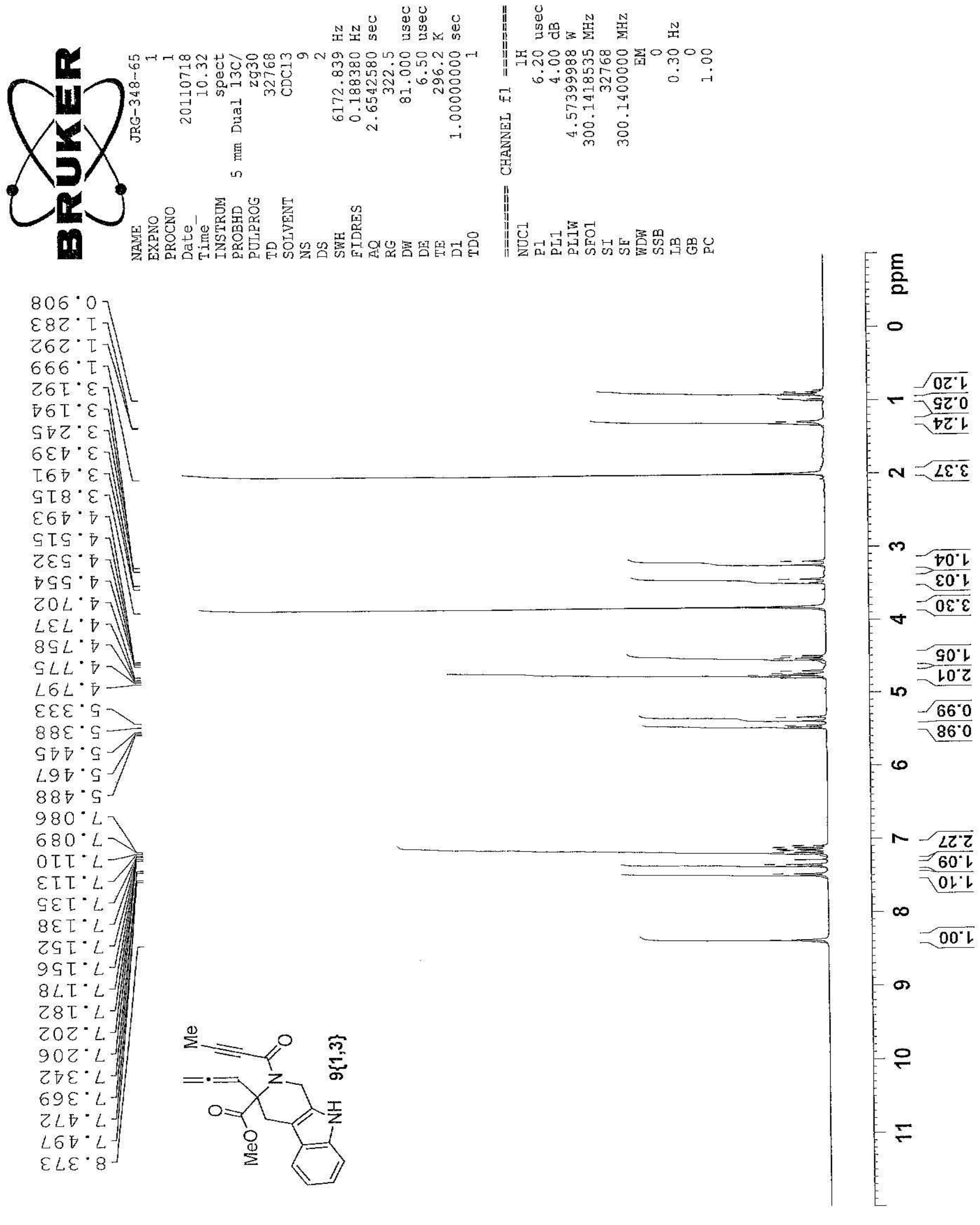

NAME JRG-348-65
EXPNO 1
PROCNO 1
Date_ 20110718
Time 10.32
INSTRUM spect
PROBHD 5 mm Dual 13C/
PULPROG zg30
TD 32768
SOLVENT CDCl3
NS 9
DS 2
SWH 6172.839 Hz
FIDRES 0.188380 Hz
AQ 2.6542580 sec
RG 322.5
DW 81.000 usec
DE 6.50 usec
TE 296.2 K
D1 1.00000000 sec
TD0 1
======== CHANNEL f1 ========
NUC1 1H
P1 6.20 usec
PL1 4.00 dB
PL1W 4.57399988 W
SFO1 300.1418535 MHz
SI 32768
SF 300.1400000 MHz
WDW EM
SSB 0
LB 0.30 Hz
GB 0
PC 1.00
0.908
1.283
1.292
1.999
3.192
3.194
3.245
3.439
3.491
3.815
4.493
4.515
4.532
4.554
4.702
4.737
4.758
4.775
4.797
5.333
5.388
5.445
5.467
5.488
7.086
7.089
7.110
7.113
7.135
7.138
7.152
7.156
7.178
7.182
7.202
7.206
7.342
7.369
7.472
7.497
8.373
1.20
0.25
1.24
3.37
1.04
1.03
3.30
1.05
2.01
0.99
0.98
2.27
1.09
1.10
1.00
ppm
MeO
O
N
O
Me
NH
9{1,3}

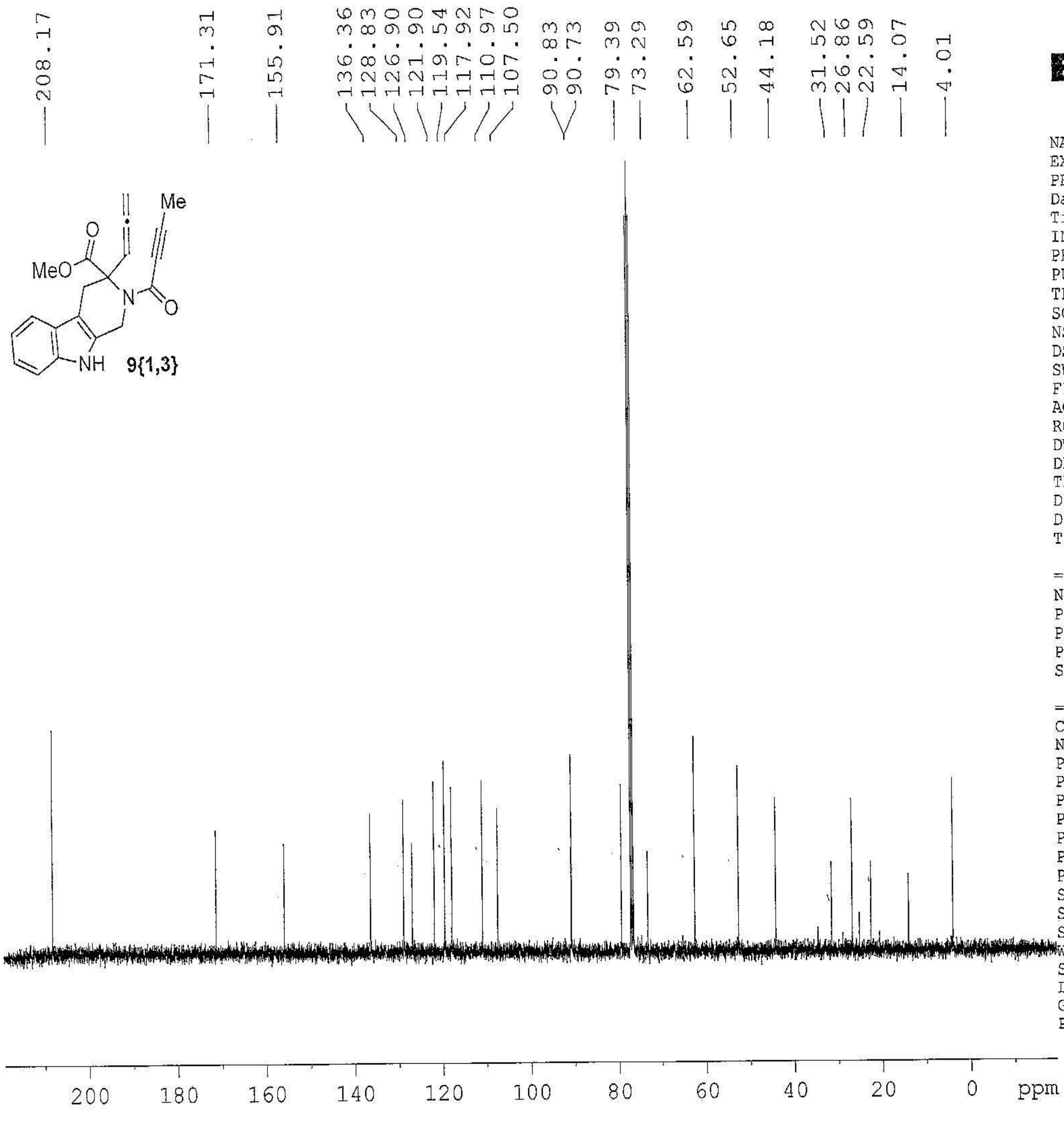

BRUKER

```
NAME       JRG-348-65_carbon
EXPNO                      1
PROCNO                     1
Date_               20110718
Time_                  13.08
INSTRUM                spect
PROBHD      5 mm PABBO BB-
PULPROG               zgpg30
TD                     65536
SOLVENT                CDCl3
NS                        91
DS                         4
SWH               24038.461 Hz
FIDRES             0.366798 Hz
AQ                1.3631988 sec
RG                       203
DW                    20.800 usec
DE                      6.50 usec
TE                     296.4 K
D1                3.00000000 sec
D11               0.03000000 sec
TD0                        1

======== CHANNEL f1 ========
NUC1                     13C
P1                     10.00 usec
PL1                    -1.59 dB
PL1W             51.07626343 W
SFO1             100.6479773 MHz

======== CHANNEL f2 ========
CPDPRG2              waltz16
NUC2                      1H
PCPD2                  75.00 usec
PL2                    -1.00 dB
PL12                   13.39 dB
PL13                   20.00 dB
PL2W             11.09959412 W
PL12W             0.40393090 W
PL13W             0.08816721 W
SFO2             400.2316009 MHz
SI                     32768
SF               100.6379230 MHz
WDW                       EM
SSB                        0
LB                      1.00 Hz
GB                         0
PC                      1.40
```

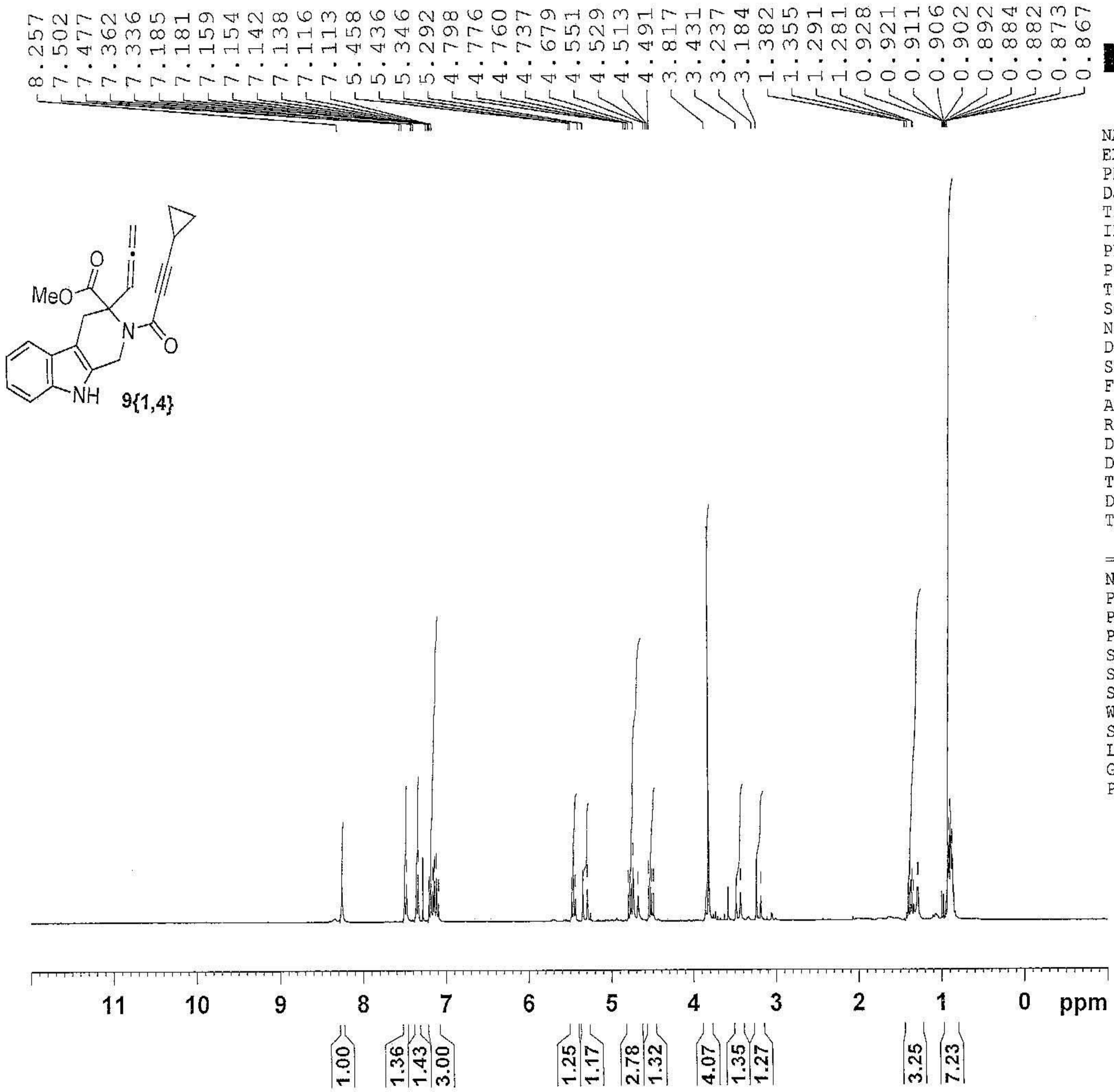

BRUKER

NAME	JRG-348-66	
EXPNO	1	
PROCNO	1	
Date_	20110718	
Time	10.37	
INSTRUM	spect	
PROBHD	5 mm Dual 13C/	
PULPROG	zg30	
TD	32768	
SOLVENT	CDCl3	
NS	11	
DS	2	
SWH	6172.839	Hz
FIDRES	0.188380	Hz
AQ	2.6542580	sec
RG	362	
DW	81.000	usec
DE	6.50	usec
TE	296.2	K
D1	1.00000000	sec
TD0	1	

======== CHANNEL f1 ========

NUC1	1H	
P1	6.20	usec
PL1	4.00	dB
PL1W	4.57399988	W
SFO1	300.1418535	MHz
SI	32768	
SF	300.1400000	MHz
WDW	EM	
SSB	0	
LB	0.30	Hz
GB	0	
PC	1.00	

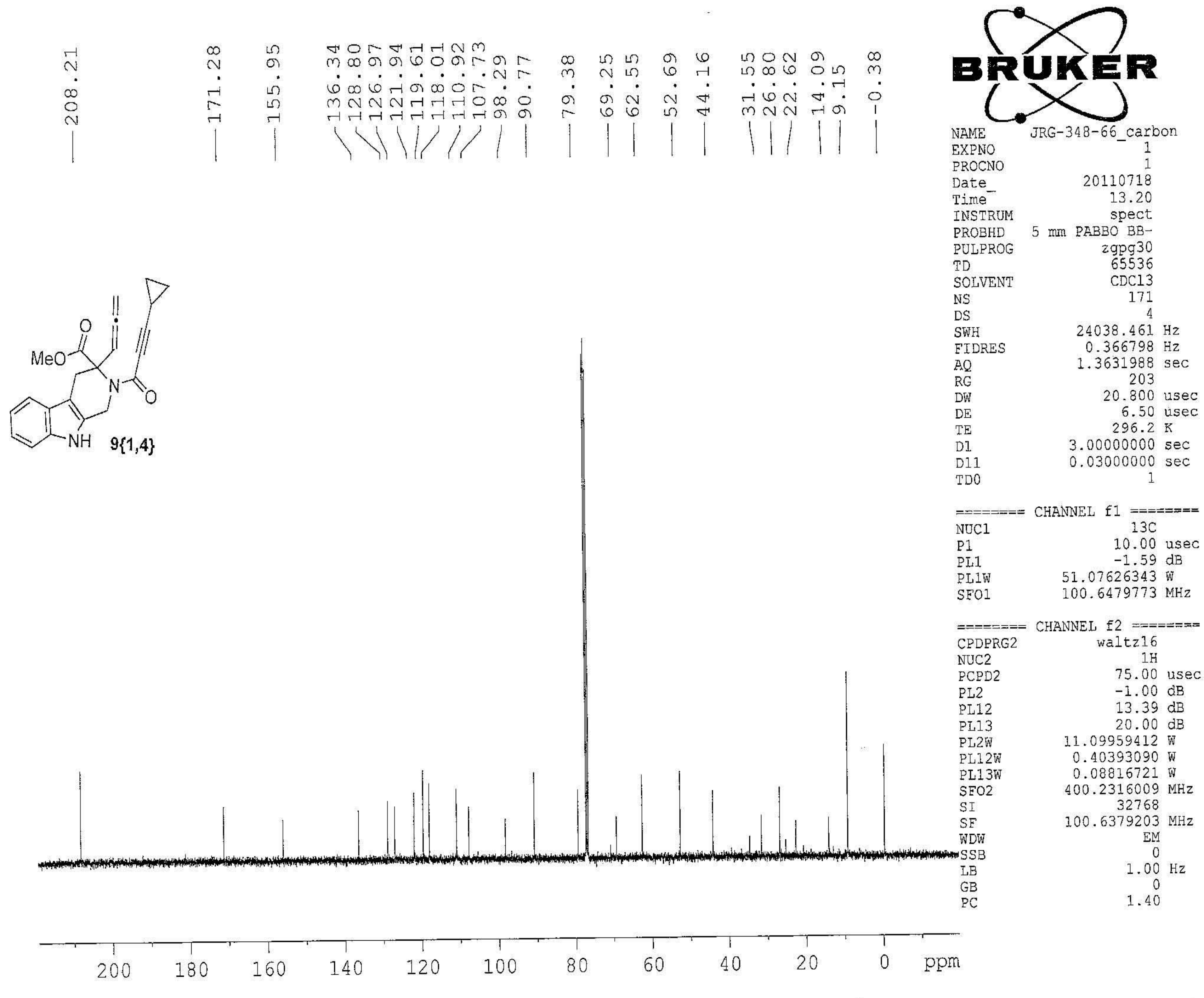
208.21
171.28
155.95
136.34
128.80
126.97
121.94
119.61
118.01
110.92
107.73
98.29
90.77
79.38
69.25
62.55
52.69
44.16
31.55
26.80
22.62
14.09
9.15
-0.38
MeO
NH
9{1,4}
BRUKER
NAME JRG-348-66_carbon
EXPNO 1
PROCNO 1
Date_ 20110718
Time 13.20
INSTRUM spect
PROBHD 5 mm PABBO BB-
PULPROG zgpg30
TD 65536
SOLVENT CDCl3
NS 171
DS 4
SWH 24038.461 Hz
FIDRES 0.366798 Hz
AQ 1.3631988 sec
RG 203
DW 20.800 usec
DE 6.50 usec
TE 296.2 K
D1 3.00000000 sec
D11 0.03000000 sec
TD0 1
======== CHANNEL f1 ========
NUC1 13C
P1 10.00 usec
PL1 -1.59 dB
PL1W 51.07626343 W
SFO1 100.6479773 MHz
======== CHANNEL f2 ========
CPDPRG2 waltz16
NUC2 1H
PCPD2 75.00 usec
PL2 -1.00 dB
PL12 13.39 dB
PL13 20.00 dB
PL2W 11.09959412 W
PL12W 0.40393090 W
PL13W 0.08816721 W
SFO2 400.2316009 MHz
SI 32768
SF 100.6379203 MHz
WDW EM
SSB 0
LB 1.00 Hz
GB 0
PC 1.40
200
180
160
140
120
100
80
60
40
20
0
ppm
